# Identification of 6‑Aryl-7-Deazapurine Ribonucleoside Phosphonates as Inhibitors of Ecto-5′-Nucleotidase (CD73)

**DOI:** 10.1021/acsptsci.5c00180

**Published:** 2025-07-17

**Authors:** Magdalena Šímová, Tereza Ormsby, Ugnė Šinkevičiu̅tė, Lucia Sirotová Veselovská, Kateřina Čermáková, Martin Hadzima, Lenka Bartoň, Jana Staňurová, Anežka Kramná, Pavel Šácha, Michal Tichý, Michal Hocek, Jan Konvalinka, Kristyna Blazkova

**Affiliations:** † Institute of Organic Chemistry and Biochemistry, Czech Academy of Sciences, Flemingovo nam. 2, CZ-16610 Prague 6, Czech Republic; ‡ Department of Organic Chemistry, Faculty of Science, Charles University in Prague, Hlavova 8, CZ-12843 Prague 2, Czech Republic; § First Faculty of Medicine, Charles University in Prague, Kateřinská 32, CZ-12108 Prague 2, Czech Republic

**Keywords:** CD73, monophosphonate, high-throughput screening, DNA-linked probe, ecto-5′-nucleotidase, tumor microenvironment

## Abstract

CD73 is a crucial regulator of adenosine production in the tumor microenvironment and, therefore, represents a valuable target for cancer immunotherapy. While different inhibitors of CD73 have been studied, the progress remains hindered by a lack of high-throughput assays that would allow the screening of large chemical libraries. Establishing a sensitive assay for the detection of CD73 activity could enable additions to the CD73 inhibitor chemical space as well as help facilitate a better understanding of the CD73 reaction mechanism. In this study, we focused on the development and adaptation of DIANA for CD73 high-throughput screening and showed that we can detect enzyme inhibition with high sensitivity. We then used this assay to screen an IOCB library, a proprietary set of chemical compounds with a special focus on nucleotide analogues. We identified several scaffolds that inhibit CD73 and in an SAR study demonstrated fine-tuning of the inhibition properties of monophosphonate analogues. Moreover, using a breast cancer cell line as a model with endogenous CD73 expression, we demonstrated the inhibition of CD73 directly on cells. The establishment of a sensitive assay for the detection of CD73 activity allowed us to develop potent inhibitors of the enzyme with low nanomolar inhibition constants. Our findings further promote the importance of CD73 inhibitors in cancer therapy.

## Graphical abstract

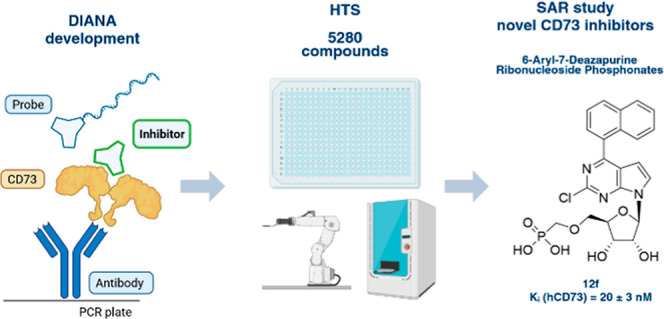

Ecto-5′-nucleotidase CD73 plays a significant role in promoting immunosuppressive conditions within the tumor microenvironment (TME). This enzyme is a critical component of the purinergic signaling pathway, catalyzing the hydrolysis of adenosine monophosphate (AMP) to adenosine (ADO). The resulting adenosine interacts with A2a and A2b receptors on tumor-infiltrating lymphocytes (TILs), inhibiting their functions and thus facilitating tumor immune evasion. Consequently, CD73 inhibition is being explored as a potential therapeutic target to enhance the efficacy of established checkpoint inhibitor therapies.^1^

Currently, there are no FDA-approved drugs targeting CD73 on the market. However, several clinical trials are underway, primarily focusing on the use of monoclonal antibodies, such as MEDI9447 (oleclumab),^2^ which block CD73 function. In parallel, significant efforts are underway to develop small-molecule CD73 inhibitors (Figure 1).^1^ These inhibitors offer several advantages over monoclonal antibodies, including efficient tissue penetration, shorter half-life, easier manufacturing, flexible administration, and lower cost. Moreover, they can be easily combined with current treatments to promote an immune response.

**Figure 1:**
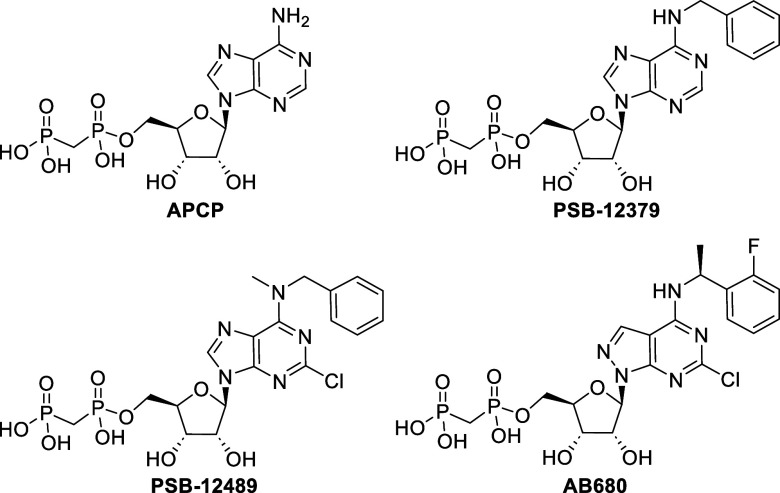
Structures of known small-molecule CD73 inhibitors.

The groundwork for the first generation of potent, selective, and metabolically stable CD73 inhibitors was laid by Müller and co-workers, who developed derivatives of α-β-methylene-ADP, which exhibited a *K*_i_ of 88.4 nM against human CD73.^3,4^ Subsequent structure–activity relationship (SAR) studies focused on enhancing inhibitor potency through targeted modifications. For instance, attachment of a benzyl group to the amine group at C6 significantly improved the inhibitory potential, resulting in PSB-12379 with *K*_i_ = 2.2 nM.^5^ Further optimization at the C2 position revealed that the incorporation of a chlorine atom enhanced potency by an order of magnitude in compound PSB-12489 with *K*_i_ = 0.381 nM.^4^ These findings culminated in the development of AB680 (Quemliclustat), the most potent CD73 inhibitor reported to date with a *K*_i_ of 5 pM.^6^ AB680 is currently the only small-molecule CD73 inhibitor that advanced to phase 2 clinical trials.^7^

The development of effective CD73 inhibitors requires robust and reliable assays to screen new compounds and identify lead structures. Current assays primarily focus on detecting the enzymatic activity of CD73. The malachite green assay and its commercially available version, the PiColorLock Gold assay, detect inorganic phosphate as a chromogenic product. However, these assays are not specific to AMP hydrolysis alone and can detect any phosphate contaminant, necessitating high substrate concentrations and limiting their suitability for high-throughput screening (HTS). In addition, the protocol requires precise timing for incubation and the addition of the developing reagents, making it challenging to implement in a high-throughput setup. The luciferase-based luminescence assay indirectly measures CD73 activity as AMP, the substrate, inhibits luciferase.^8^ While adapted for high throughput, this assay does not measure CD73 activity directly. The Transcreener ADO CD73 assay utilizes the immunodetection of nucleotides and fluorescence polarization readout.^9^ CD73 activity is determined indirectly via a coupling enzyme, ADO kinase, which converts adenosine to ATP and ADP, with ADP subsequently detected by an anti-ADP antibody coupled to a tracer. While this facilitates high-throughput testing, optimization is complex, and the assay suffers from a narrow dynamic range due to ADP being a weak CD73 inhibitor.^3,5^ Additionally, it may identify compounds inhibiting the ADO kinase. Multiplexed RapidFire-tandem mass spectrometry (RF-MS/MS) measures CD73 activity using four plates, each containing different isotopically labeled AMP derivatives as substrates.^10^ After reaction quenching, the plates are pooled for analysis using RF-MS/MS. This assay is highly sensitive and allows for the screening of large chemical libraries thanks to multiplexing. The isotopically labeled AMP derivatives are used at concentrations near their *K*_m_ values, enabling the identification of inhibitors with various modalities. However, the synthesis of these substrates is complex and costly, and the assay requires sophisticated instruments and data analysis, making its establishment challenging.

In this study, we establish and optimize the DNA-linked Inhibitor ANtibody Assay (DIANA) as a simple, reliable, and sensitive method to directly measure the active site competition of CD73. By combining sandwich ELISA with quantitative PCR (qPCR) detection, this technique can identify new inhibitors in the competitive format.^11–13^ The procedure involves immobilizing the target enzyme in a multiwell plate using a specific antibody. The enzyme is then detected with a synthetic probe consisting of a DNA oligonucleotide covalently attached to a known competitive inhibitor (the competitive inhibitor could alternatively be replaced with an allosteric inhibitor or noninhibitory ligand if so desired). The amount of the bound probe is then quantified using qPCR (Figure 2A). For inhibitor screening, compounds are evaluated based on their ability to outcompete the detection probe from a single-well experiment. Thanks to the fully automated format,^13^ DIANA can be easily used to perform high-throughput screening of extensive chemical libraries in just a few hours.

**Figure 2:**
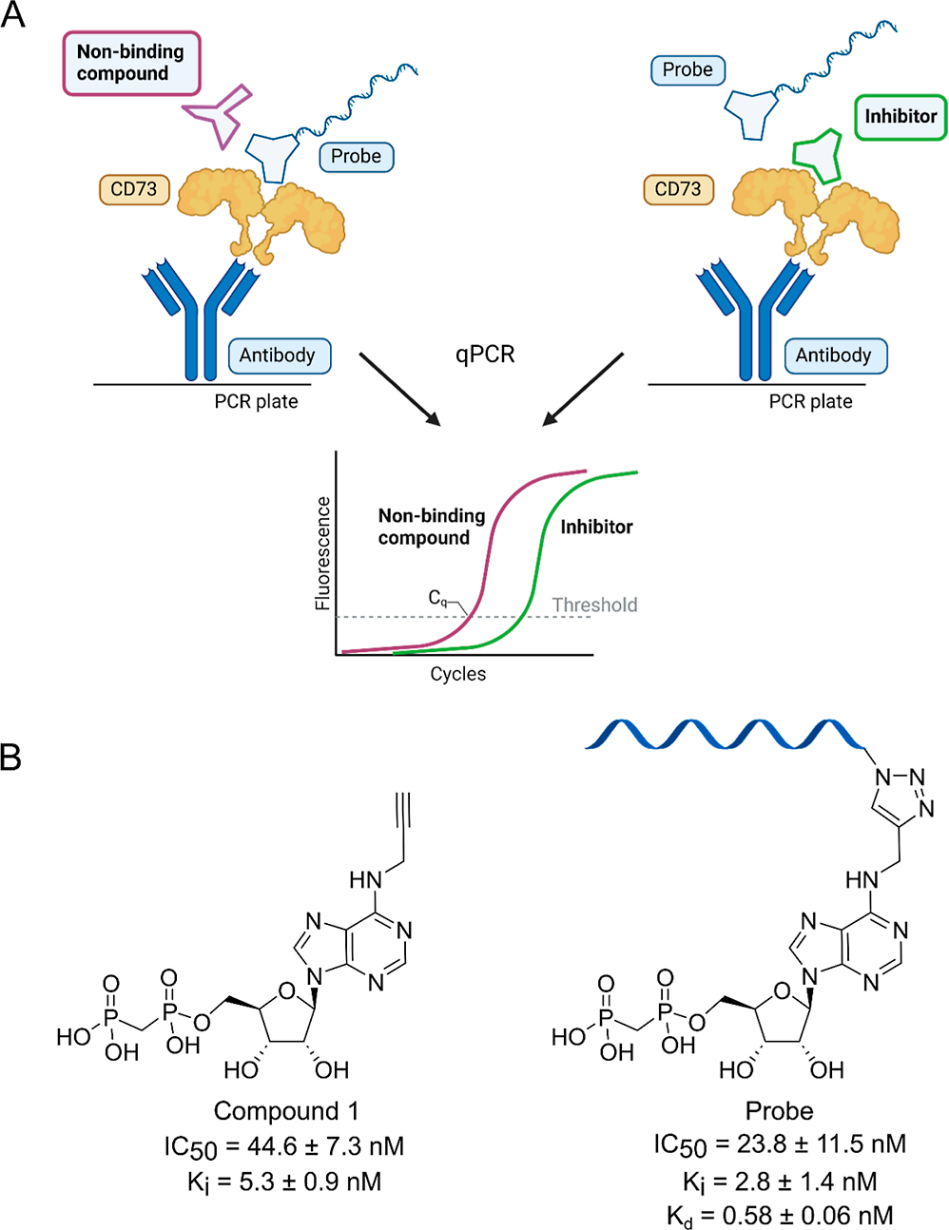
CD73 DIANA. (A) Representation of the CD73 DIANA assay—recombinant human CD73 (rhCD73) is immobilized through an anti-His antibody, and a mixture of the tested compounds with a CD73-specific probe is added. The bound probe is detected by qPCR. (B) Structure, inhibitory properties, and binding of the probe precursor (compound **1**) and the probe (compound **2**). IC_50_ and *K*_i_ values were measured using rhCD73 in an activity assay. Data were analyzed using nonlinear regression using GraphPad Prism, and *K*_i_ values were calculated with the Cheng–Prusoff equation. Displayed values represent the mean ± standard deviation (s.d.) of two experiments. *K*_d_ value of the probe was determined using DIANA; the probe was added to rhCD73 in a dilution series and the amount of bound probe was monitored by qPCR. Created in BioRender. Stanurova, J. (2025) https://BioRender.com/apitgfi.

## Results and Discussion

### Development of DIANA for CD73

To first establish a sensitive assay, we adapted DIANA for CD73 (Figure 2A). The key aspect of DIANA is probe preparation, which requires modifying an inhibitor with a DNA oligo to allow qPCR readout. Several structural studies had already been published and allowed us to rationally design a new small-molecule inhibitor with a click-reactive propargyl handle attached at the 6-amino position of the adenine moiety (compound **1**, Figure 2B and Scheme S1). The position for handle attachment was derived from available structural information as well as the published position of fluorophore attachment.^14^ First, acetonide-protected *N* ^6^-propargyl-adenosine (**S1**) was prepared according to a published procedure.^15^ This intermediate was then phosphonylated using methylenebis­(phosphonic dichloride), and the acetonide-protecting group was removed to yield compound **1** (probe precursor, Scheme S1). Using click chemistry, compound **1** was conjugated with an oligonucleotide to generate the probe (compound **2**; Figure 2B). Following the click reaction, the inhibitor featured an aromatic ring at the 6-amino position, analogous to the well-described CD73 inhibitor PSB-12379.

To verify that the binding of the probe to CD73 was not compromised by the attachment of a linker and conjugation to an oligonucleotide, we evaluated the inhibitory capacity of **1** as well as the probe using an enzymatic assay. Results showed that binding and inhibition properties were retained with **1** inhibiting CD73 with IC_50_ 44.6 ± 7.3 nM and *K*_i_ 5.3 ± 0.9 nM and the probe inhibiting CD73 with IC_50_ 23.8 ± 11.5 nM and *K*_i_ 2.8 ± 1.4 nM (Figure 2B). This design of the probe allowed effective binding to CD73 through the inhibitor and easy detection through qPCR using the oligonucleotide of the probe as a template. We then determined the equilibrium dissociation constant (*K*_d_) of the probe in DIANA to be 0.58 ± 0.06 nM (Figure 2B).^11^

### CD73 DIANA Optimization and Validation

High-throughput screening requires the use of chemical libraries that are composed of DMSO stock solutions. To assess the impact of DMSO on the enzyme, we tested a range of DMSO concentrations up to 10% in DIANA and up to 25% in an orthogonal assay (Figure 3A, Table S1). In the activity assay, we did not observe any effect on CD73 activity in concentrations up to 3% DMSO, but even concentrations up to 6% still showed that CD73 retained over 90% activity. In DIANA, DMSO concentrations tested had little impact on the *C*_q_ values measured. We also optimized the assay buffer conditions (data not shown), adapted the assay to 384-well plates,^12^ and determined the amount of the enzyme to allow high sensitivity (Figure 3B).

**Figure 3:**
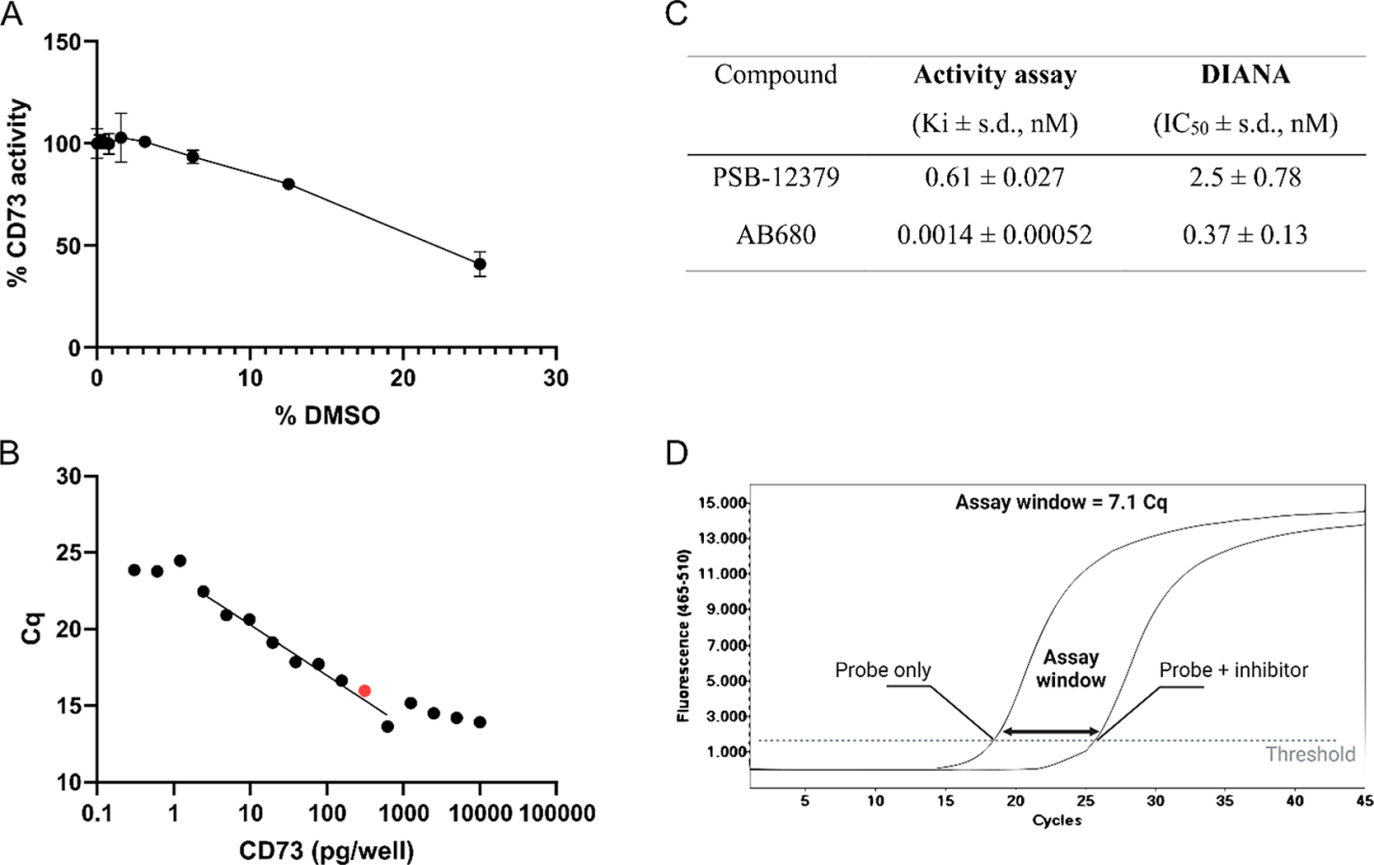
Optimization of DIANA conditions. (A) Effect of DMSO on CD73 activity. rhCD73 was incubated in various concentrations of DMSO, and CD73 activity was monitored by the activity assay. Displayed values represent duplicates from a single measurement. (B) Optimization of enzyme concentration for DIANA. The effect of the rhCD73 concentration on DIANA was determined by qPCR. The red dot indicates the concentration selected for further work. (C) Determination of the inhibitory activity of inhibitors PSB-12379 and AB680 in the activity assay and in DIANA. Data from the activity assay were analyzed using nonlinear regression using GraphPad Prism, and *K*_i_ values were calculated using the Morrison equation. The reported values represent the mean of three experiments, along with the standard deviation (s.d.). For DIANA, IC_50_ was calculated from % of probe binding inhibition using nonlinear regression in GraphPad Prism. Displayed values represent the mean of three experiments; s.d., standard deviation. (D) Schematic representation of the assay window. The assay window was calculated as a difference of the mean *C*_q_ value of the inhibited reaction (probe + 1 μM PSB12379) and the mean *C*_q_ value of the noninhibited reaction (probe only).

To further validate the DIANA setup, we used published inhibitors of CD73—PSB-12379 ^5^ and AB680 ^6^ (Figure 1)—in both DIANA and the activity assay. We observed that in both assays, CD73 was inhibited by the molecules (Figure 3C). While small differences in *K*_i_ values were observed compared to those in the literature, these can be attributed to variations in assay formats, protein constructs, and data analysis methods. This likely reflects inherent differences in assay principles: DIANA estimates binding, while the enzymatic assay captures the inhibition of catalytic turnover. Additional factors such as buffer composition, probe competition, and protein immobilization may also contribute. Despite these differences, both methods produced consistent inhibitor ranking, supporting the reliability of DIANA for hit evaluation. This experiment validated that DIANA was indeed able to detect inhibitors of CD73. We, therefore, determined the dynamic range of DIANA, referred to as the assay window. To that end, we measured the difference between *C*_q_ values of uninhibited binding of the probe to CD73 and maximum inhibition achieved through PSB-12379. The observed assay window reached 7.1 cycles (Figure 3D), which was sufficient to proceed to HTS.

### Screening of the IOCB Compound Library

The IOCB compound library contains over five thousand original compounds, including a number of nucleotide analogues synthesized at IOCB. We hypothesized that this library was likely to contain new binders of CD73 since published inhibitors of this enzyme are often nucleotide analogues themselves. DIANA allows HTS in a 384-well format as well as screening of several compounds pooled into a single well due to its high sensitivity,^11–13^ which would allow us to screen the library with high efficiency.

The screen was performed with a total of 5,280 IOCB library compounds in a fully automated workflow. The compounds were pooled, so that 11 compounds were present in a single well. Pooled compound screening compressed the screen on less than two 384-well plates, minimizing the resources needed. PSB-12379, a known CD73 inhibitor, served as an interplate control in HTS. The screening strategy (Figure 4A) and the screen parameters (Figure 4B) are described for clarity. Wells where Δ*C*_q_ (difference between the uninhibited reaction and reaction with an inhibitor) exceeded the calculated threshold value were classified as primary hits (Figure 4C).

**Figure 4:**
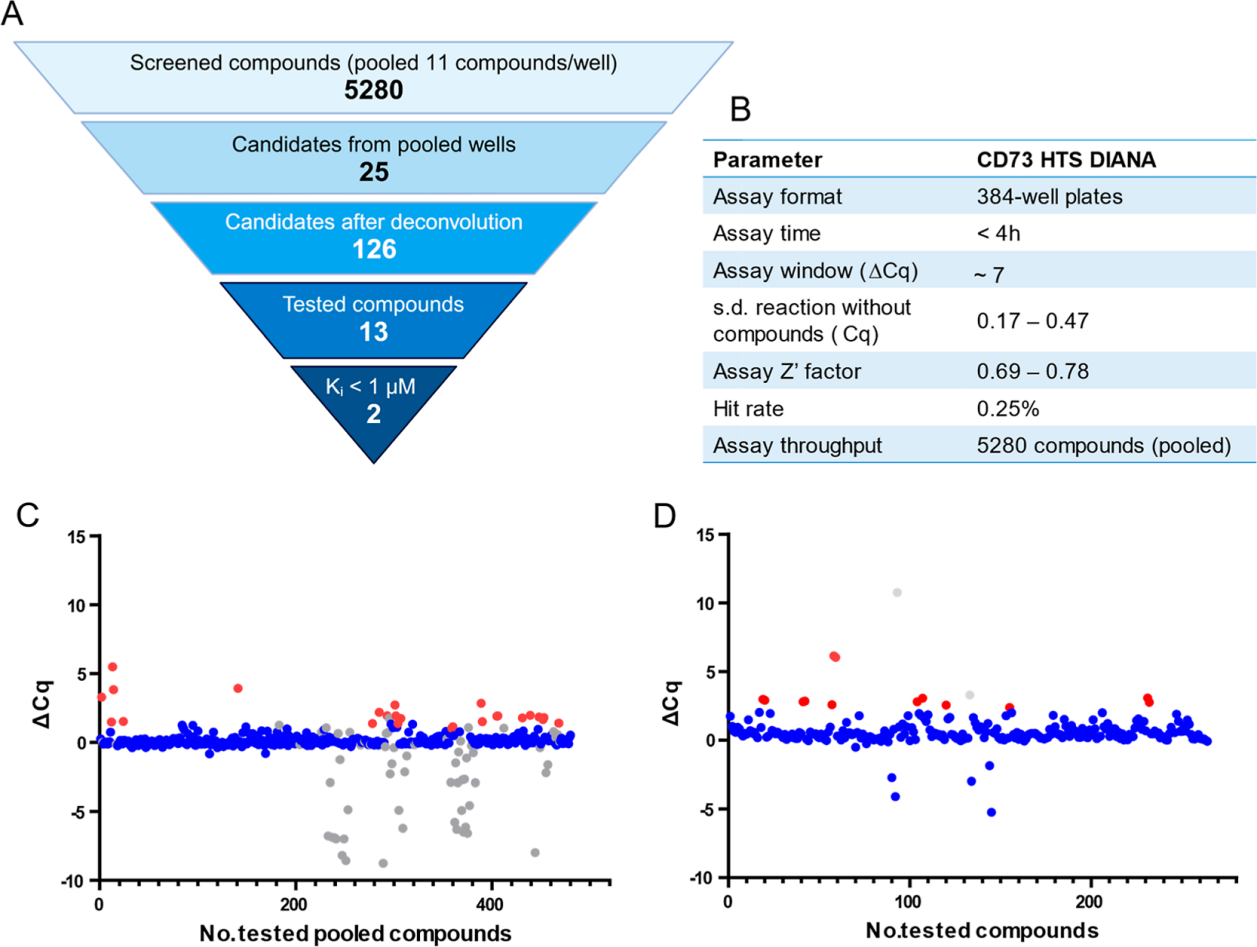
Library screening using DIANA. (A) Workflow of CD73 HTS DIANA. The IOCB library of 5,280 compounds was screened in pools of 11 compounds per well. The primary screen identified 25 pooled hits, and subsequent deconvolution yielded 126 candidate compounds. Thirteen hits with Δ*C*_q_ > 2.2 were tested in the activity assay, confirming five CD73 inhibitors as true hits. Two hits with *K*_i_ below 1 μM were used in further experiments. (B) Parameters of CD73 HTS using DIANA. (C) Primary screening results showing Δ*C*_q_ values obtained in pooled wells. Each dot represents a well of the screen: blue (nonbinding compounds), gray (interfering compounds), and red (hits). Two wells with Δ*C*_q_ > 20 were evaluated as interference and omitted for clarity. (D) Deconvolution of the CD73 screen. Gray dots indicate interfering compounds; blue dots represent individual compounds. Red dots denote hits with Δ*C*_q_ > 2.2. Created in BioRender. Stanurova, J. (2025) https://BioRender.com/cmufrk5.

To exclude wells where compounds interfered with the qPCR readout or the inhibition was not competitive, a counter-screen was conducted. In the counter-screen, a known CD73 inhibitor, PSB-12379, was added to each well at 200 nM and changes in *C*_q_ values were monitored. Two criteria were used to eliminate interferences: wells were labeled as interfering if (a) the *C*_q_ value in the counter-screen was significantly lower than the *C*_q_ of the uninhibited reaction or significantly higher than the background signal, indicating interference with the assay or the PCR readout, or (b) the addition of PSB-12379 did not further increase *C*_q_ compared to primary screen to at least the level of 200 nM PSB-12379 inhibition. The readouts from the interfering wells were excluded from further analysis.

We assessed the HTS parameters for both the primary screen and the counter-screen. The average *C*_q_ values of 32 uninhibited wells were 16.74 ± 0.36 in the pooled screen and 16.73 ± 0.23 in the counter-screen. The most inhibited wells were used to determine the assay window. The average *C*_q_ values from 6 wells in these most inhibited wells were 23.73 ± 0.29 in the pooled screen and 23.53 ± 0.34 in the counter-screen. These *C*_q_ values resulted in an assay window of 7.0 for the pooled screen and 6.8 for the counter-screen. The screening parameters and assay window demonstrated sufficient reproducibility, which corresponded well with prior assay optimization (Figures 3D and 4B).

Additionally, the quality of our HTS was assessed using the *Z*′ factor, which is commonly used to describe assay quality. The *Z*′ factor for the pooled IOCB library was 0.72 and 0.74 for the counter-screen. *Z*′ factors well above 0.5 indicate an excellent assay quality, with a clear separation band between positive and negative signals for HTS. The values observed for screen and counter-screen were above 0.7, which represented highly reliable results. The combination of screening and counter-screening ultimately identified 25 pooled well hits.

### Hit Deconvolution and Validation

To identify which compounds in the pooled wells were responsible for the inhibition of CD73, we deconvoluted the hits by testing individual compounds from the pooled wells using DIANA (Figure 4D). The deconvolution experiment had similar assay parameters to the primary screen and counterscreen (average *C*_q_ values for the uninhibited wells 17.01 ± 0.20 and PSB-12379-inhibited wells 23.82 ± 0.28, assay window = 6.74, *Z*′ = 0.79). This process confirmed that 126 compounds inhibited CD73 in the 10 μM concentration used in the screen. Out of these, candidates with Δ*C*_q_ > 2.2 were subjected to further analysis except for two that were excluded as interferences. This resulted in 13 hits and a hit rate of 0.25%. Testing for dose-dependent effects in the activity assay confirmed five of them as true CD73 inhibitors (Figure 5A). Two compounds (Figure 5B) with the highest CD73 inhibition were structurally similar, both belonging to the class of nucleoside analogues and bearing resemblance to compounds reported by Sharif et al.^16^ Their *K*_i_ values lie below 1 μM, which aligns well with their similarity to published CD73 inhibitors.

**Figure 5:**
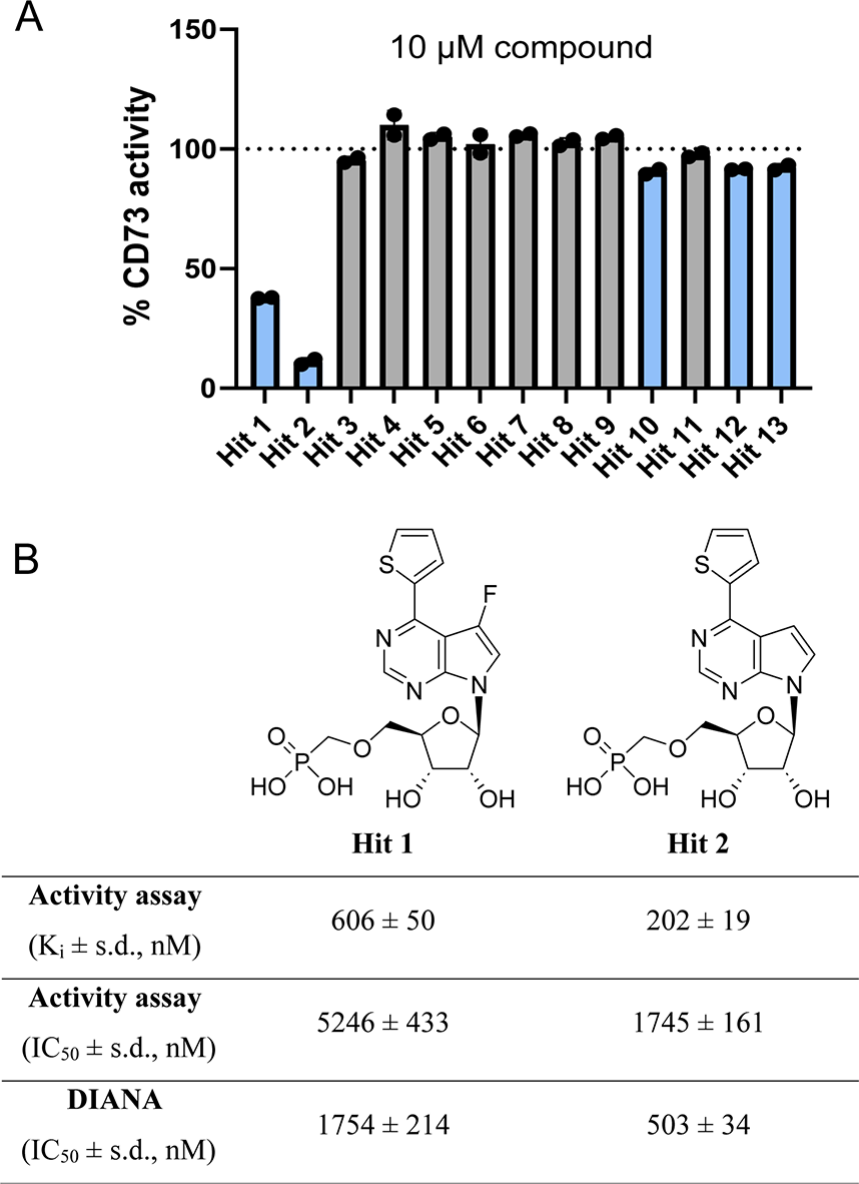
Identification of CD73 inhibitors. (A) Evaluation of hits using an orthogonal activity assay. rhCD73 was incubated with 13 hits at 10 μM and CD73 activity was monitored using the activity assay. Data are normalized as the percentage of CD73 activity. Blue bars represent inhibiting compounds; gray bars depict noninhibiting compounds. (B) Structures of Hit 1 and Hit 2 and their evaluation in orthogonal assay and dose–response DIANA. rhCD73 was incubated with a dilution series of compounds, and CD73 activity was monitored using the activity assay. Data were analyzed using nonlinear regression using GraphPad Prism and *K*_i_ values were calculated with the Cheng–Prusoff equation. Displayed values represent the mean of three experiments; s.d., standard deviation. For DIANA, IC_50_ was calculated from % of probe binding inhibition using nonlinear regression in GraphPad Prism. Displayed values represent the mean of two experiments; s.d., standard deviation.

Hits 1 and 2 share a common scaffold of 7-deazapurine ribonucleoside 5′-*O*-phosphonomethyl ethers, each bearing thiophen-2-yl group in position 6.^17^ The only structural difference is the presence of a fluorine atom at position 7 on the nucleobase in Hit 1, which worsened the inhibitory property of this compound (Figure 5B). We, therefore, investigated the impact of structural variations of Hit 2 on the inhibitory properties and to that end performed a structure–activity relationship study (SAR). The study focused on 7-deazapurine ribonucleoside 5-*O*-phosphonomethyl ethers, specifically examining the binding affinity of (het)­aryl substituents of different sizes and orientations in position 6.

### SAR Study

To prepare the desired set of compounds, our initial synthetic strategy toward the 6-hetaryl 7-deazapurine ribonucleoside 5-*Ó*-phosphonomethyl ethers **7** was based on the published synthesis of hits 1 and 2.^17^ Hetaryl groups were introduced first into position 6 by Suzuki coupling. Following deprotection of the *tert*-butyldimethylsilyl (TBDMS) group, the 5′-OH group was alkylated by phosphonomethyl tosylate to the desired protected phosphonates **5a–c**. The final two-step deprotection resulted in target phosphonomethyl ethers **7a–c** (Scheme 1, approach A). However, introducing variability at the beginning of a 4-step synthesis was not practical for the synthesis of a larger series of compounds. To reduce the number of synthetic steps and to introduce the desired aryl groups into position 6 as late as possible, we tried an alkylation with phosphonomethyl tosylate on 6-chloro derivative **8**. However, all attempts at this reaction were unsuccessful and led only to decomposition of the starting material. The same outcome was previously observed on a related 6-chloropurine nucleoside.^18^ Therefore, we switched the first two steps and started with deprotection of the 5′-OTBDMS group in nucleoside **2** using tetra-*n*-butylammonium fluoride (TBAF), followed by Suzuki coupling, phosphonomethylation, and final deprotection to the target phosphonomethyl ethers **7d–j** (Scheme 1, approach A). The same synthetic strategy was then successfully used for the synthesis of 6-substituted 2-chloro-7-deazapurine analogues **12b**,**d**,**f**,**g**, which were isolated in poor yields due to laborious purification (Scheme 1, approach B). Compound purity was analyzed using HPLC and UPLC (Table S4). Yields of all reaction steps are summarized in the Supporting Information (Table S5).

**Scheme 1:**
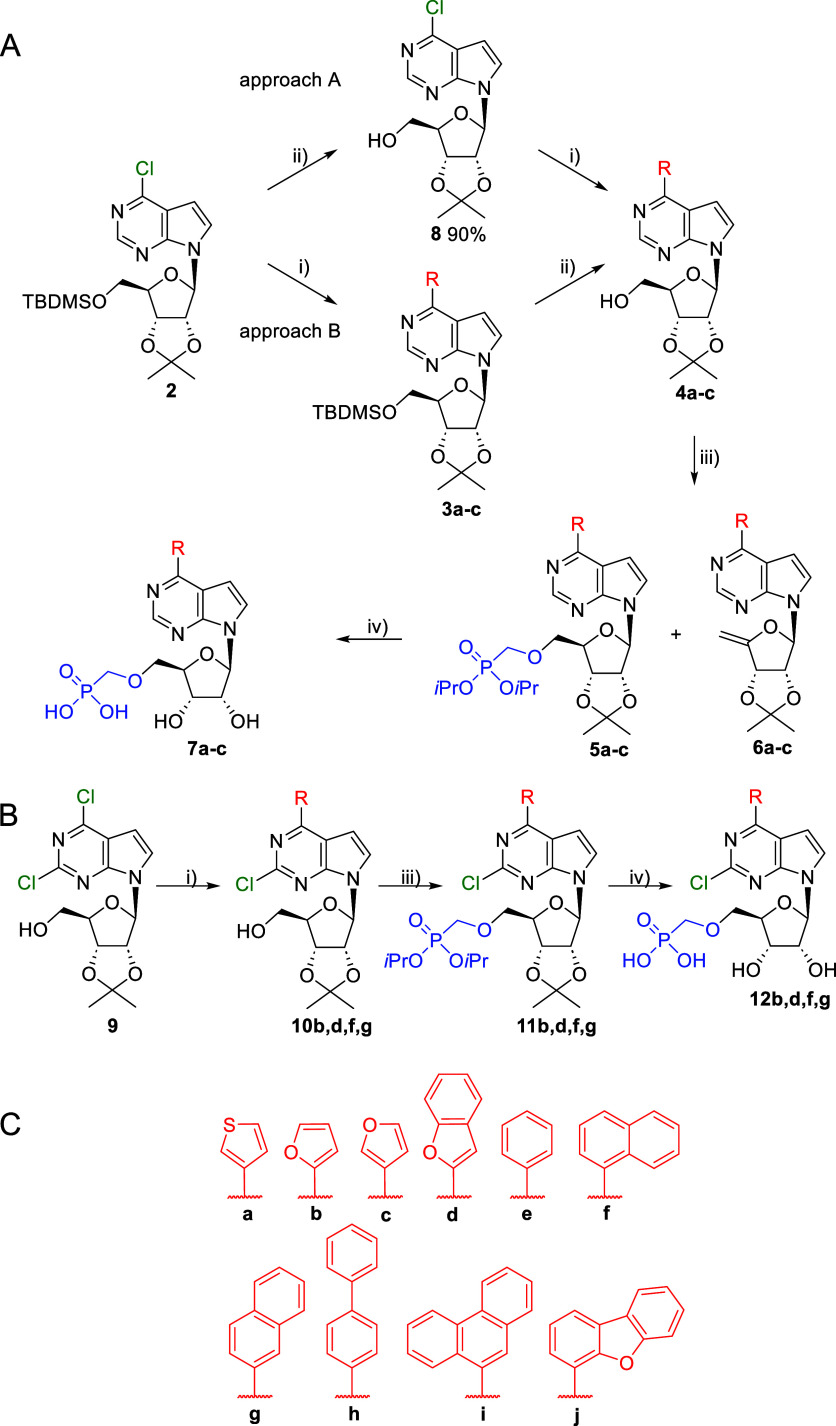
(A) Synthetic Scheme for the Production of 6-Substituted 7-Deazapurine Analogues; (B) Synthetic Scheme for the Production of 6-Substituted 2-Chloro-7-Deazapurine Analogues; (C) Structures of Substituents in Position 6 Synthesized in This Study^a^

a Conditions: (i) R–B­(OH)_2_, K_2_CO_3_, Pd­(PPh_3_)_4_, DMF, 100 °C, 16 h; (ii) TBAF 1 m solution in THF, 22 °C, 20 min; (iii) phosphonomethyl tosylate, NaH, DMF, −20 to 22 °C, 1–1.5 h; (iv) (1) TMSBr, 2,6-lutidine, CH_3_CN, 22 °C, 1–2 days; (2) TFA, 22 °C, 40 min.

We tested all of the final phosphonomethyl ethers **7a–j** and **12b**,**d**,**f**,**g** for their inhibitory activity against both mouse and human CD73 enzymes using the activity assay. Results are summarized in Table 1.

**Table 1:**
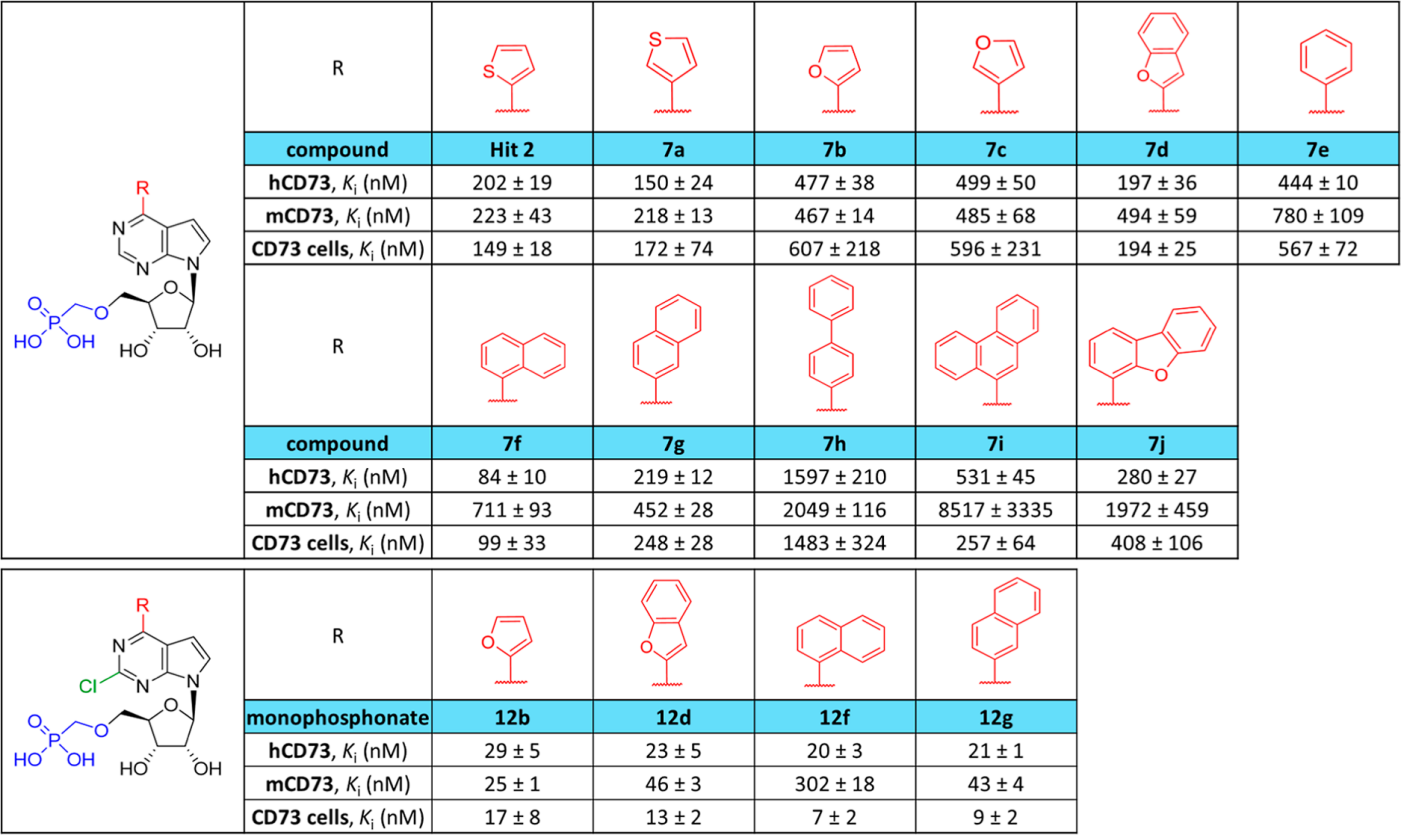
Inhibition of CD73 by Target Compounds^a^

a Human rhCD73, mouse recombinant CD73 (rmCD73), and CD73-expressing human breast cancer cell line MDA-MB-231 cells were incubated with a dilution series of compounds, and CD73 activity was monitored using the activity assay. Data were analyzed using nonlinear regression using GraphPad Prism and *K*_i_ values were calculated using the Cheng–Prusoff equation. Displayed values represent the mean of three experiments; s.d., standard deviation.

In general, all compounds inhibited both mouse and human enzymes and were more potent against human CD73 (the only exceptions are both 2-furyl derivatives **7b** and **12b**). In the first series of phosphonomethyl ethers **7**, the most potent inhibitor was 1-naphthyl derivative **7f** with *K*_i_ = 84 nM. Its 2-naphthyl isomer **7g** was 2.5-fold less active, suggesting that the orientation of the aryl substituent was more important than just its size, which is supported by the fact that the dibenzofuranyl derivative **7j** was twice as active as the phenanthrenyl derivative **7i**. On the other hand, all derivatives bearing a monocyclic aryl group (**7a–c**, **e**) were less potent than the 1-naphthyl derivative **7f**, which meant that the aryl group was involved in interaction with CD73 and was important for binding.

Unlike (some of) the parent nucleosides,^19^ which were found to be potent cytostatics, the original screening hits 1 and 2 ^17^ and the synthesized compounds showed little to no cytotoxicity against four cancer cell lines (CCRF-CEM, HepG2, HeLa S3, HL-60) and nonmalignant human dermal fibroblasts (NHDF) at the tested concentration (10 μM) (Table S2). Lack of cytotoxic activity of the target compounds is very likely caused by the presence of the phosphonate group, which is charged under physiological conditions and hampers cellular uptake.

### Mouse CD73 Possesses Different Structural Requirements Than Human CD73

Results from the mouse CD73 inhibition assay show that the mouse enzyme was more sensitive to the size of the aryl group at position 6. Derivatives with bulky aryl groups were much less potent against mCD73 than against hCD73 (16-fold for **7i**, 7-fold for **7j**), whereas in the case of derivatives with small aryl groups like furanyl **7c**, thiophenyl **7a**, and phenyl **7e**, the drop of activity going from human to mouse CD73 was only 1.5- to 2.5-fold.

Introduction of a chlorine atom into position 2 on the nucleobase improved the potency of all four derivatives against both human and mouse enzymes **12b**,**d**,**f**,**g** with *K*_i_ in the range from 20 to 29 nM. The aryl groups in these 2-chloro derivatives had almost no impact on CD73 inhibition, suggesting either a different binding mode from the 2-unsubstituted analogues or that the presence of a chlorine atom outweighs the impact of aryl groups in position 6. This fact could be used for fine-tuning of compound properties by small modifications of the aryl group.

### Selectivity of Monophosphonates Against CD39

The purinergic pathway utilized by cancer cells to produce immunosuppressive adenosine is composed of several steps. The CD73 activity is preceded by CD39 activity where extracellular adenosine tri- and diphosphate is hydrolyzed to AMP, which then acts as a substrate for CD73. Due to high structural similarity of the substrates in the purinergic pathway, we set out to verify that our molecules are selective for CD73; therefore, we have also tested them against CD39. We first established a stable clonal CD39 cell line (Figure S1) and then used it to test the activity of the synthesized compounds against CD39 in a cell-based assay. All inhibitors studied in our SAR were highly selective for CD73 over CD39 with CD39 IC_50_ > 10 μM (Table S3).

### Monophosphonates Inhibit Adenosine Production in a Breast Cancer Cell Line MDA-MB-231

To establish whether the developed inhibitors of CD73 could block adenosine production in cells, we used a human breast cancer cell line MDA-MB-231, which expresses CD73.^20^ We first verified that we detected adenosine production using an activity assay and optimized assay conditions for inhibition measurement. We were able to confirm that similarly to biochemical experiments, the developed compounds were able to inhibit adenosine production from AMP by blocking CD73 on cells (Table 1). The *K*_i_ obtained from the cell-based assay correlated well with values measured with the purified recombinant enzyme. The most potent compound **12f** had the lowest *K*_i_ in both biochemical and cell-based testing (20 ± 4 nM for rhCD73 and 7 ± 2 nM *K*_i_ in CD73-expressing cells).

In conclusion, we developed a new DIANA assay for high-throughput screening of CD73 inhibition and used it to identify inhibitors in the IOCB chemical library. Sensitive detection through qPCR allowed us to screen the IOCB library in a pooled format with eleven compounds per well followed by subsequent deconvolution of the hits. The screening and deconvolution of hits fit on three plates (five with counterscreen) instead of eleven+ that would usually be required to screen a library of this size, allowing us to easily screen 5,280 compounds. The DIANA assay has the potential for further high-throughput testing of other compound libraries and identification of new ligands. Two of the obtained hits had a *K*_i_ in hundreds of nanomolar and were structurally close; therefore, we used this scaffold as a lead compound for a SAR study. We developed a set of potent CD73 inhibitors that inhibited not only human and mouse CD73 but also blocked adenosine production by a human breast cancer cell line.

## Materials and Methods

### Preparation of the DIANA Probe

Compound **1** (see SI, Scheme S1 and Supporting Methods) was derived from the APCP inhibitor,^3,5^ where the amino group attached to the C6 was modified with an alkyne to enable binding to the DNA oligonucleotide^11^ using click chemistry. The DNA-oligonucleotide-azide was prepared by coupling an amine-bearing DNA-oligonucleotide with azido-PEG4-NHS-ester. After purification using Amicon Ultra centrifugal filter units with a 10K molecular weight cutoff (Merck), the DIANA detection probe was prepared via a copper-catalyzed azide–alkyne cycloaddition (CuAAC) between compound **1** and the azide-bearing oligonucleotide. The conjugate was purified again using Amicon centrifugation filters.

### Cloning, Expression, and Purification of Recombinant CD73 Extracellular Domain

DNA sequence encoding the enzymatic domain of human CD73 (residues 27–549) was amplified by PCR using primers 5′-AAAGGATCCGGAAAACTTGATCCGACCTTC-3′ (reverse) and 5′-AAAGCTAGCTGGGAGCTTACGATTTTGCAC-3′ (forward) from pCMV-N-Flag-NT5E (SinoBiological). DNA sequence encoding the enzymatic domain of mouse CD73 was amplified by PCR using primers 5′-CTTCAGCTGAGTCTTGGAATTGCTAGCTGGGAGCTCACGATCCTGCAC-3′ (forward) and 5′-GGTGATGGTGACCGGTGCTGGATCCCTGGAAGTACAGGTTCTCAGAGAACTTGATCCGCCCTT-3′ (reverse) from pCMV3-*mNT5E* (SinoBiological). Both PCR fragments were then subcloned into plasmid pTT28 (National Research Council of Canada) between the *Bam*HI and NheI restriction sites. The DNA sequence of the human and mouse CD73 can be found in Figure S2. The pTT28-rhCD73 and pTT28-rmCD73 vectors include an N-terminal secretion leader sequence and a C-terminal 6xHisTag sequence, facilitating high-yield protein expression and secretion in Expi293 cells. Briefly, Expi293F (Thermo Fisher Scientific) in 100 mL of Expi293 Met (-) medium was transiently transfected with 100 μg of pTT28-rhCD73 or pTT28-rmCD73 using the ExpiFectamine^TM^ 293 Transfection Kit (Thermo Fisher Scientific) according to the manufacturer’s instructions. After 6 days of incubation (37 °C, 8% CO_2_, 120 rpm), the medium was collected, and the proteins were purified in two steps. First, enzymes were purified by affinity chromatography using cOmplete His-Tag Purification Resin (Sigma-Aldrich). CD73 bound to the chelator with Ni^2+^ was eluted by 300 mM imidazole in Tris buffer (300 mM NaCl, 50 mM Tris, pH 7.0). Half of the protein was dialyzed against TBS (200 mM NaCl, 50 mM Tris, 4 mM CaCl_2_, pH 7.5) and further purified using a HiLoad Superdex 200 prep grade column (GE Healthcare Life Sciences). The purified proteins were dialyzed against storage buffer (120 mM NaCl, 20 mM Tris, 4 mM CaCl_2_, 20% glycerol, pH = 7.5) and stored at −80 °C until use. SDS-PAGE was performed to assess protein purity, and the final concentrations of the purified proteins were determined by the Bradford assay, resulting in a total yield of 3.2 mg of rhCD73-6xHis and 3.7 mg of rmCD73-6xHis.

### DIANA

The general DIANA protocol for CD73 was adapted from Navratil et al.^11^ with the following modifications. For the immobilization, 50 ng of 6x-His Tag Monoclonal Antibody (cat. no. MA1135, Thermo Fisher Scientific, USA) was captured on the surface of a 96-well qPCR plate by incubation in TBS (Tris-buffered saline, 20 mM Tris–HCl, 150 mM NaCl; pH 7.5) for 1 h. The antibody-coated wells were then blocked with a casein blocker in TBS overnight. After washing, 300 pg of His-tagged rhCD73 was added in TBST′Mg (TBST′ = TBS + 0.1% Tween, with 5 μM MgCl_2_) and incubated for 1 h to allow binding to the immobilized antibody. Excess rhCD73 was washed away. Next, 1 nM of the probe (together with a tested compound in the case of inhibitors testing) was added in TBST′Mg supplemented with 1,000× diluted Casein Buffer (20X-4X concentrate; 5.5%) and incubated for 1 h. The equilibrium dissociation constant (*K*_d_) of the DIANA detection probe was determined as previously described.^11^ Briefly, *K*_d_, the probe was added to immobilized rhCD73 at varying concentrations ranging from 5 nM to 2 pM. In the end, the unbound probe was washed out, and the qPCR mixture was added to the wells, followed by qPCR analysis.

### DIANA High-Throughput Screening of a Compound Library

Based on Tykvart et al.,^12^ we modified the 96-well plate protocol described above to the HTS format by using 384-well plates in the same way. We tested the IOCB compound library under the same conditions as mentioned above in this new format. As was reported in Tykvart,^12^ we screened the pooled IOCB library, which is still growing and nowadays consists of >5,000 compounds. The pooled library was prepared in the same manner as was described previously: 11 compounds were pooled together into a well of the pooled library (the concentration of a compound in every pooled well is 9.1 μM).

Simultaneously, the counter screen^21^ with the pooled library was tested. The known inhibitor PSB-12379 was added into every well of the pooled library in the concentration of 200 nM. Two criteria were used to eliminate interferences: wells were labeled as interfering if (a) the *C*_q_ value in the counter-screen was significantly lower than the *C*_q_ of the uninhibited reaction or significantly higher than the background signal, indicating interference with the assay or the PCR readout or (b) the addition of PSB-12379 did not further increase *C*_q_ compared to primary screen to at least the level of 200 nM PSB-12379 inhibition. Interferences were excluded from further analysis. The threshold Δ*C*_q_ value for hits was established based on standard deviation (s.d.) of controls (wells without inhibitor). We had 16 wells of controls in each plate. Due to one outlier, the standard deviation was 0.47 in the first plate and the statistical threshold for hits calculated by the formula 2 × (2^0.5^) × s.d. was 1.34 cycles. In the second plate, the s.d. was 0.17, and the calculated statistical threshold was 0.47 cycle. To avoid selecting low potent compounds, we applied a stricter empirical threshold of 1 cycle in the second plate. We found 25 positive pooled wells, and they were tested compound-by-compound (275 compounds) afterward under the same condition. We identified 15 hits with Δ*C*_q_ with significant CD73 inhibition (higher than 2.2 cycles). Of these, two were previously identified as interfering in an unrelated screen, so they were excluded.

As controls for the DIANA assay in the case of the IOCB library screening, we used the PSB-12379 published inhibitor as positive controls and only DMSO as negative controls. The positive control was set up as published previously^12^—the serial dilution of the inhibitor from 0.2 pM to 200 nM concentration in duplicates. The determination of *K*_i_ values was performed as published.^12^

### Testing of CD73 Activity

The PiColorLock Gold kit (Novus Biologicals) was used to determine the inhibition constants of the tested compounds. We followed the standard protocol with specific conditions as follows: 1.25 ng of rhCD73 or 0.8 ng of rmCD73 in 80 μL of TBST (TBS + 0.05% Tween) was mixed with a dilution series of the tested compound in 10 μL of TBST. The reaction was initiated by adding 10 μL of 2 mM AMP. After 30 min at room temperature, 25 μL of PiColorLock Gold was added, followed by 10 μL of Stabilizer after 5 min. After a further 30 min, the absorbance was measured at 635 nm using an Infinite M1000 plate reader (Tecan). The phosphate calibration curve was used to calculate the conversion rate for each well. The percentage of activity was calculated as the ratio of the sample well to the average of the uninhibited reaction (the maximum conversion did not exceed 20%). The resulting values were plotted, and IC_50_ values were calculated using GraphPad Prism 10.2.1. *K*_i_ values were calculated using the Cheng–Prusoff equation or the Morrison equation. To determine the *K*_M_ and *V*_max_ values, 1.25 ng of rhCD73 and 0.8 ng of rmCD73 were incubated with a dilution series of AMP (2 μM–1,000 μM) in Tris buffer (20 mM Tris, 150 mM NaCl, pH 7.5) for 30 min at RT. The activity of the enzymes was monitored using the PiColorLock Gold kit. *K*_M_ and *V*_max_ values were calculated using the Michaelis–Menten fit in GraphPad Prism 10.2.1 (Figure S3).

### Cell Culture

All cell lines were grown and maintained at 37 °C, 5% CO_2_, >95% humidity, and ambient oxygen levels in a cell culture incubator. All media were supplemented with 10% fetal bovine serum (FBS-Gibco). Cells lines were obtained and cultured as follows: MDA-MB-231 (kindly provided by Dr. Cyril Bařinka) was grown in full RPMI 1640 (Biosera, Biotech), Expi293F (Thermo Fisher Scientific) was grown in Expi293 Expression medium (Thermo Fisher Scientific), and HEK293T (kindly provided by Dr. Kvido Stříšovský) was grown in DMEM (Sigma-Aldrich).

### Preparation of the CD39-Transfected HEK293T Cell Line

HEK293T cells were seeded in a 24-well plate to achieve 50–60% confluency on the day of transfection. A mixture of 0.5 μg of CD39 plasmid (pCMV3-HA-ENTPD1, SinoBiological) in 25 μL of OptiMEM (Gibco) and 1.5 μL of Lipofectamine (Invitrogen) was incubated at room temperature for 15 min and then gently added dropwise to the cells. The next day, the cells were transferred to a 100 mm^2^ Petri dish with 200 μg/mL hygromycin B (Roche) and 400 μg/mL Geneticin (G418, Sigma-Aldrich). The cells were allowed to grow, and the media with antibiotics were replaced every fourth day. Developed colonies were transferred using cloning cylinders to a 24-well plate. CD39 expression was tested using an APC-conjugated anti-CD39 antibody (Exbio) and analyzed by flow cytometry with a BD LSRFortessa flow cytometer, gating for single cells. Data were analyzed with FlowJo 10.4.1. For activity experiments, we selected clonal line 293T-CD39-27 (SI, Figure S1).

### Cellular Assay for Testing CD73 and CD39 Activity or Specificity

A modified PiColorLock Gold protocol was used to determine the inhibition constants of the tested compounds on a CD73 endogenously expressing cell line (MDA-MB-231) and to test the selectivity of the tested compounds against CD39 on transfected HEK cells (293T-CD39-27). On the day of the experiment, an aliquot of cells stored at −80 °C was thawed at 37 °C and immediately diluted into 10 mL of assay buffer (20 mM HEPES, 137 mM NaCl, 5.4 mM KCl, 1.3 mM CaCl_2_, 4.2 mM NaHCO_3_, 0.1% glucose). The cells were then washed twice in 1 mL of the same buffer. Afterward, cells were counted using an automatic cell counter. 6,000 MDA-MB-231 cells or 500 293T-CD39-27 cells in 70 μL of assay buffer were mixed with a dilution series of the tested compound in 10 μL of assay buffer. After 1 h of incubation at 37 °C, the reaction was initiated by adding 20 μL of 1 mM AMP for MDA-MB-231 cells or 20 μL of 250 μM ATP for 293T-CD39-27 cells. After 30 min at room temperature, the plate with cells was spun down and 80 μL of the supernatant was mixed with 20 μL of PiColorLock Gold, followed by the addition of 8 μL of Stabilizer after 5 min. The subsequent procedure was the same as that for the recombinant protein.

### Cytotoxicity

The cytotoxicity of all compounds synthesized in this manuscript was assessed as described previously.^22^ Briefly, four cancer cell lines (CCRF-CEM, HepG2, HeLa S3, HL-60) and nonmalignant human dermal fibroblasts (NHDF), all from American Tissue Culture Collection (ATCC), were grown as recommended by ATCC. For cytotoxicity determination, cells were plated at 2,000–50,000 cells/well in a white 384-well plate and incubated overnight. Compounds were added at 10 μM (final concentration) and incubated with cells for 72 h in a cell incubator. Cell viability was evaluated using CellTiter-Glo 2.0 (Promega) reagent with a luminescence readout. Untreated control wells were set as 100% viability, and the signal in sample wells was calculated as a percentage of the maximum.

## Supplementary Material



## Notes

Additional biochemical characterizations of reagents used in the manuscript as well as the methods section and characterizations of chemical compounds developed in the present work (PDF)
