# Supplementary material for: Identification of 6‑Aryl-7-Deazapurine Ribonucleoside Phosphonates as Inhibitors of Ecto-5′-Nucleotidase (CD73)
Source: ACS Pharmacol Transl Sci. 2025 Jul 17;8(8):2575–85. doi: 10.1021/acsptsci.5c00180 (PMC12340617; doi:10.1021/acsptsci.5c00180)
Supplement: Supplementary file 1 [file pt5c00180_si_001.pdf]

Supporting Information for:

Identification of 6-Aryl-7-Deazapurine Ribonucleoside Phosphonates as Inhibitors of Ecto-5'-Nucleotidase (CD73)

Magdalena Šímová#[a], Tereza Ormsby#[a], Ugnė Šinkevičiūtė#[a,b], Lucia Sirotová Veselovská[a], Kateřina Čermáková[a,c], Martin Hadzima[a,b], Lenka Bartoň[a], Jana Staňurová[a], Anežka Kramná[a], Pavel Šácha[a], Michal Tichý[a], Michal Hocek [a,b] , Jan Konvalinka*[a] and Kristyna Blazkova*[a]

[a] *Institute of Organic Chemistry and Biochemistry, Czech Academy of Sciences, Flemingovo nam. 2, CZ-16610 Prague 6, Czech Republic.*

[b] *Department of Organic Chemistry, Faculty of Science, Charles University in Prague, Hlavova 8, CZ-12843 Prague 2, Czech Republic.*

[c] *First Faculty of Medicine, Charles University in Prague, Kateřinská 32, CZ-12108 Prague 2, Czech Republic*

#these authors contributed equally

*corresponding authors

Contact information for corresponding authors:

jan.konvalinka@uochb.cas.cz

blazkova@stanford.edu

Table of Contents

SUPPORTING DATA – BIOCHEMISTRY 3

Figure S1. Preparation of CD39-expressing stable cell line in HEK 293. 3

Table S1. DMSO effect in CD73 DIANA. 3

Table S2. % of surviving cells at 10 µM compound concentration 4

Table S3. CD39 inhibition by the developed compounds. 5

Figure S2. DNA sequences of proteins expressed for this study 5

Figure S3. K_M and Vmax measurement of rhCD73 and rmCD73. 7

SUPPORTING METHODS AND DATA - CHEMISTRY 8

Synthetic Experimental Part 8

Synthesis of DIANA probe precursor compound 1 9

Scheme S1. Synthesis of DIANA probe precursor compound 1. 10

Synthesis of compounds for structure-activity relationship study 12

Table S4. HPLC and UPLC purity of compounds 1, 7a-j and 12b,d,f,g. 54

Table S5: Yields of compounds 3-7, 10, 12. 56

NMR spectra of reported compounds 57

Supporting data – biochemistry

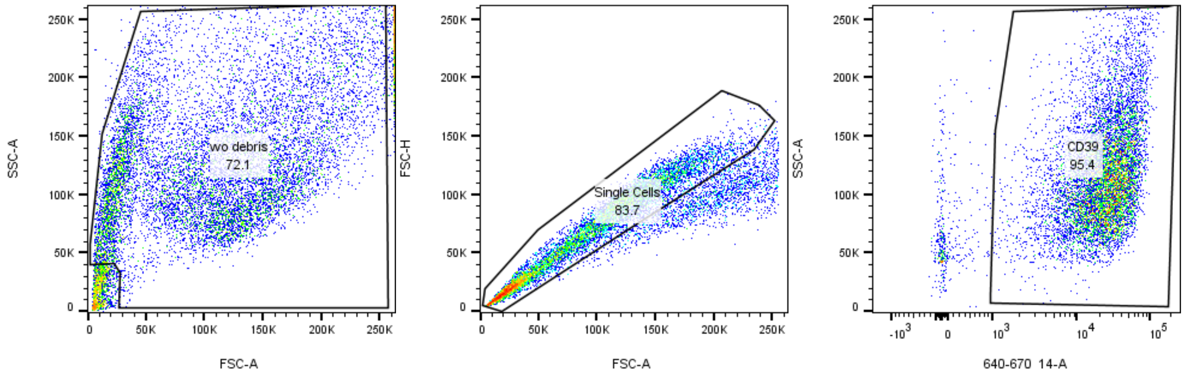

Figure S1. Preparation of a CD39-expressing stable cell line in HEK 293. Flow cytometry was used to evaluate the expression of CD39 in a CD39-transfected HEK293 clonal line using a specific antibody. Detached cells were stained with 10× diluted APC-conjugated anti-CD39 antibody (EXBIO, 1A-184-T100). CD39 expression was detected by flow cytometry, and data were analyzed with FlowJo 10.4.1.

Table S1. DMSO effect in CD73 DIANA. Immobilized rhCD73 was incubated with the probe in various concentrations of DMSO. Binding was monitored by qPCR.

DMSO (%)	Cq	s.d.
0	18.1	0.4
1	18.3	0.2
5	18.4	0.2
10	18.2	0.2

Table S2. % of surviving cells at 10 μM compound concentration

Compound	CEM	HeLa	HepG2	HL60	NHDF
**7a**	95±6	94±5	86±8	94±5	97±6
**7b**	77±3	84±4	77±3	92±3	85±2
**7c**	82±7	89±6	88±6	89±7	90±3
**7d**	87±4	85±3	90±5	93±3	89±4
**7e**	84±3	85±5	87±5	89±3	87±3
**7f**	88±6	93±3	96±5	98±4	91±3
**7g**	96±1	97±5	96±6	100±5	96±4
**7h**	90±10	86±4	81±4	90±2	93±4
**7i**	88±4	100±2	93±2	93±4	72±6
**7j**	84±5	87±5	88±4	88±4	82±9
**12b**	120±3	101±5	128±6	95±11	107±0
**12d**	97±2	88±8	105±9	99±8	109±3
**12f**	115±6	88±6	104±6	95±10	98±1
**12g**	114±5	103±1	117±7	102±10	114±1

Table S3. CD39 inhibition by the developed compounds. HEK cells transfected with CD39 (293T-CD39-27) were incubated with dilution series of the tested compounds, and CD39 activity was monitored using the PiColorLock Gold assay. Data were analyzed using nonlinear regression in GraphPad Prism.

Compound	IC_{50} (293T-CD39-27) (µM)
Hit 1	>10
7a	>10
7b	>10
7c	>10
7d	>10
7e	>10
7f	>10
7g	>10
7h	>10
7i	>10
7j	>10
12b	>10
12d	>10
12f	>10
12g	>10

Figure S2. DNA sequences of proteins expressed for this study. Underlined parts depict the catalytic domains.

Human CD73

ATGGGAGAACTGCTGCTGCTCCTTCTGCTGGGGCTGCGGCTTCAGCTGAGTCTTGGAATTGCTAGCT
GGGAGCTTACGATTTTGCACACCAACGACGTGCACAGCCGGCTGGAGCAGACCAGCGAGGACTCCA
GCAAGTGCGTCAACGCCAGCCGCTGCATGGGTGGCGTGGCTCGGCTCTTCACCAAGGTTCAGCAGA
TCCGCCGCGCCGAACCCAACGTGCTGCTGCTGGACGCCGGCGACCAGTACCAGGGCACTATCTGGTT
CACCGTGTACAAGGGCGCCGAGGTGGCGCACTTCATGAACGCCCTGCGCTACGATGCCATGGCACT
GGGAAATCATGAATTTGATAATGGTGTGGAAGGACTGATCGAGCCACTCCTCAAAGAGGCCAAATTT

CCAATTCTGAGTGCAAACATTAAAGCAAAGGGGCCACTAGCATCTCAAATATCAGGACTTTATTTGCC
ATATAAAGTTCTTCCTGTTGGTGATGAAGTTGTGGGAATCGTTGGATACACTTCCAAAGAAACCCCTT
TTCTCTCAAATCCAGGGACAAATTTAGTGTTTGAAGATGAAATCACTGCATTACAACCTGAAGTAGAT
AAGTTAAAAACTCTAAATGTGAACAAAATTATTGCACTGGGACATTCGGGTTTTGAAATGGATAAAC
TCATCGCTCAGAAAGTGAGGGGTGTGGACGTCGTGGTGGGAGGACACTCCAACACATTTCTTTACAC
AGGCAATCCACCTTCCAAAGAGGTGCCTGCTGGGAAGTACCCATTCATAGTCACTTCTGATGATGGG
CGGAAGGTTCCTGTAGTCCAGGCCTATGCTTTTGGCAAATACCTAGGCTATCTGAAGATCGAGTTTG
ATGAAAGAGGAAACGTCATCTCTTCCCATGGAAATCCCATTCTTCTAAACAGCAGCATTCCTGAAGAT
CCAAGCATAAAAGCAGACATTAACAAATGGAGGATAAAATTGGATAATTATTCTACCCAGGAATTAG
GGAAAACAATTGTCTATCTGGATGGCTCCTCTCAATCATGCCGCTTTAGAGAATGCAACATGGGCAA
CCTGATTTGTGATGCAATGATTAACAACAACCTGAGACACACGGATGAAATGTTCTGGAACCACGTA
TCCATGTGCATTTTAAATGGAGGTGGTATCCGGTCGCCCATTGATGAACGCAACAATGGCACAATTA
CCTGGGAGAACCTGGCTGCTGTATTGCCCTTTGGAGGCACATTTGACCTAGTCCAGTTAAAAGGTTCC
ACCCTGAAGAAGGCCTTTGAGCATAGCGTGCACCGCTACGGCCAGTCCACTGGAGAGTTCCTGCAG
GTGGGCGGAATCCATGTGGTGTATGATCTTTCCCGAAAACCTGGAGACAGAGTAGTCAAATTAGATG
TTCTTTGCACCAAGTGTCGAGTGCCCAGTTATGACCCTCTCAAAATGGACGAGGTATATAAGGTGATC
CTCCCAAACTTCCTGGCCAATGGTGGAGATGGGTTCCAGATGATAAAAGATGAATTATTAAGACATG
ACTCTGGTGACCAAGATATCAACGTGGTTTCTACATATATCTCCAAAATGAAAGTAATTTATCCAGCA
GTTGAAGGTCGGATCAAGTTTTCCGGATCCAGCACCGGTCACCATCACCACCATCACCACCACGGGT
GA

Mouse CD73

ATGGGAGAACTGCTGCTGCTCCTTCTGCTGGGGCTGCGGCTTCAGCTGAGTCTTGGAATTGCTAGCT
GGGAGCTCACGATCCTGCACACAAACGACGTGCACAGCCGGCTAGAGCAGACCAGCGATGACTCCA
CCAAGTGCCTCAACGCCAGCCTGTGTGTGGGCGGCGTGGCCCGGCTCTTTACCAAGGTGCAGCAGAT
CCGCAAGGAAGAACCCAACGTGCTGTTTTTGGATGCCGGAGACCAGTACCAGGGCACCATCTGGTTC
ACCGTTTACAAAGGCCTTGAAGTGGCACACTTCATGAACATCCTGGGCTACGATGCTATGGCACTGG
GAAATCATGAATTTGATAACGGTGTGGAAGGACTGATTGATCCCCTCCTCAGAAACGTTAAATTTCCA
ATTCTGAGCGCAAACATTAAGGCACGGGGGCCTCTAGCACATCAGATATCTGGACTTTTTCTGCCATC
TAAAGTTCTCTCTGTTGGCGGTGAGGTTGTGGGGATTGTTGGATATACTTCAAAGGAAACCCCTTTCC
TCTCAAATCCAGGGACAAATTTAGTCTTTGAAGATGAAATCTCTGCATTGCAGCCTGAAGTAGATAAA
CTAAAGACGCTAAATGTGAATAAGATCATCGCCCTGGGGCACTCTGGTTTTGAGATGGACAAACTTA

TCGCTCAGAAAGTTCGAGGTGTGGACATCGTGGTGGGAGGACACTCCAACACCTTTCTCTACACAGG
AAATCCACCTTCCAAAGAAGTGCCTGCGGGGAAGTACCCATTCATAGTCACCGCAGATGATGGACGG
CAGGTGCCTGTGGTCCAGGCCTATGCCTTTGGCAAATACCTGGGCTACCTGAAGGTTGAGTTTGATG
ATAAAGGCAATGTTATCACTTCCTATGGAAATCCCATTCTTCTCAACAGCAGCATTCCTGAAGATGCG
ACCATCAAAGCAGACATTAACCAATGGAGGATAAAATTAGATAATTATTCTACCCAGGAACTCGGGA
GAACGATCGTCTACCTGGATGGCTCCACTCAGACGTGCCGCTTCAGGGAATGCAACATGGGAAACCT
GATCTGTGATGCCATGATTAACAACAACCTCAGACACCCAGATGAAATGTTTTGGAACCACGTGTCCA
TGTGCATTGTAAACGGAGGTGGCATCCGGTCCCCCATTGATGAGAAGAACAATGGTACCATCACCTG
GGAGAACCTGGCTGCTGTGCTGCCCTTTGGAGGGACATTTGACCTCGTCCAATTAAAAGGGTCCACC
CTGAAGAAGGCTTTTGAGCACAGCGTGCATCGCTATGGCCAGTCCACCGGAGAGTTCCTGCAAGTG
GGTGGAATCCATGTGGTGTACGATATTAACCGAAAGCCCTGGAACAGAGTGGTCCAATTAGAAGTTC
TCTGCACCAAGTGTCGAGTGCCCATCTATGAGCCTCTTGAAATGGATAAAGTGTATAAAGTGACCCTC
CCAAGCTATCTGGCCAACGGTGGAGATGGATTCCAGATGATAAAAGATGAATTACTAAAGCATGACT
CTGGTGATCAAGATATCAGCGTGGTTTCTGAATACATCTCAAAAATGAAAGTAGTTTACCCAGCCGTT
GAAGGGCGGATCAAGTTCTCTGAGAACCTGTACTTCCAGGGATCCAGCACCGGTCACCATCACCACC
ATCACCACCACGGGTG

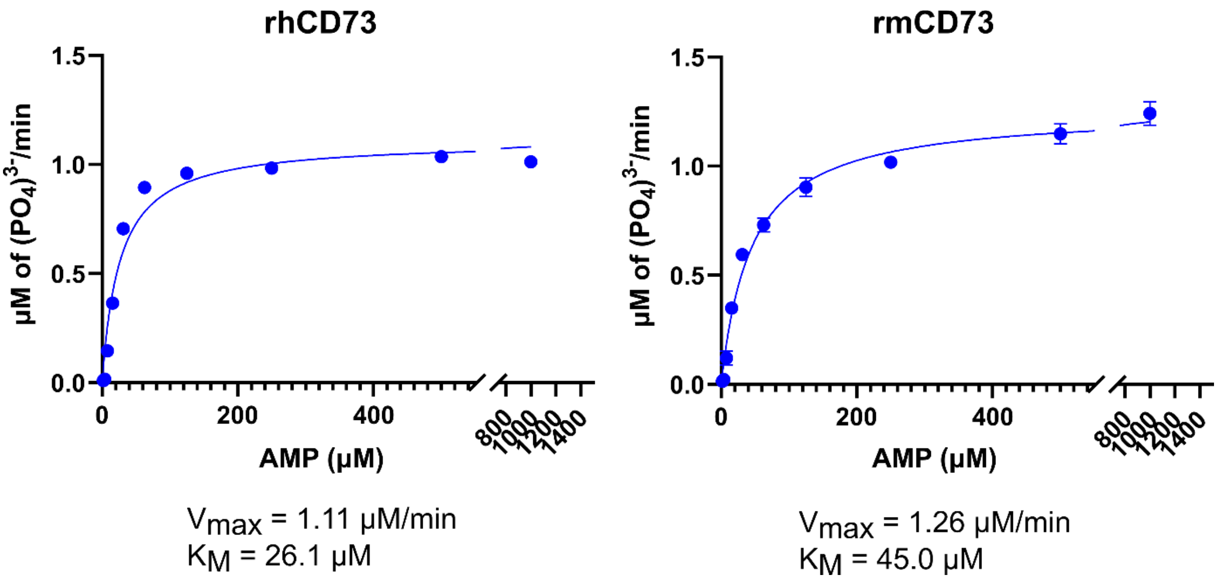

Figure S3. K_M and Vmax measurement of rhCD73 and rmCD73. rhCD73 and rmCD73 were incubated with a dilution series of AMP (2-1000 µM), and the enzymes’ activity was monitored by the activity assay. K_M and V_{max} values were calculated using the Michaelis-Menten fit.

Supporting methods and data - chemistry

Synthetic Experimental Part

General remarks: All solvents and reagents were purchased from commercial suppliers and used as received. Reactions were monitored by thin layer chromatography (TLC) on TLC Silica gel 60 F_{254} (Merck) and detected by UV (254 nm) and by Advion Expression Compact Mass Spectrometer connected to a Plate Express® TLC Plate Reader using electrospray ionization (ESI). NMR spectra were measured on Bruker Avance 400 MHz spectrometer (400.1 MHz for ^{1}H and 100.6 MHz for ^{13}C), Bruker Avance 500 MHz spectrometer (499.8 MHz for ^{1}H, 125.7 MHz for ^{13}C and ^{31}P at 202.3 MHz) or Bruker Avance 600 MHz spectrometer (600.1 MHz for ^{1}H and 150.9 MHz for ^{13}C) in DMSO-*d6* (referenced to the residual solvent signal, [δ (^{1}H) = 2.50 ppm, δ (^{13}C) = 39.7 ppm]), in D_2O (dioxane used as external standard, [δ (^{1}H) = 3.75 ppm, δ (^{13}C) = 67.19 ppm]) or in $CDCl_3$ (referenced to the residual solvent signal, [δ (^{1}H) = 7.26 ppm, δ (^{13}C) = 77.0 ppm]). Chemical shifts are given in ppm (δ-scale), coupling constants (*J*) in Hz. Complete assignments of all NMR signals was performed using a combination of H,H-COSY, H,H-ROESY, H,C-HSQC and H,C-HMBC experiments. Low-resolution mass spectra were measured on an LCQ Fleet (Thermo Fisher Scientific) using electrospray ionization (ESI). High-resolution mass spectra were measured on an LTQ Orbitrap XL (Thermo Fisher Scientific). All mass spectra were acquired by the MS service at IOCB. High-performance flash chromatography (HPFC) was performed with ISCO Combiflash Rf system on RediSep Rf Gold Silica Gel Disposable columns or Reverse Phase (C18) RediSep Rf column. When needed, purification of final free phosphonates was performed using HPLC (Waters modular HPLC system) on a column packed with 10 µm C18 reversed phase (Phenomenex, Luna C18 (2) 100 Å). Purity of all final compounds (>95%) was determined by analytical HPLC and by clean NMR spectra. HPLC analysis was performed on a Waters 600 HPLC system (Waters 600 Controller, Waters 2996 Photodiode Array Detector), Column: Gemini 5µ C18 110A (250 × 4.60 mm, 5 micron), Flow: 1 mL/min. UPLC-MS analysis was performed on an *Agilent 1260 Infinity II LC* system with an *Agilent 1260 Photodiode Array Detector*, Column: *Kinetex EVO C18 100 Å 1.7 µm* (2.1 × 150 mm), Flow: 0.2-0.25 mL/min.

General Procedure A: Suzuki cross-coupling reaction

DMF or H_2O/MeCN (1:1) was added through a septum to an argon purged vial containing protected nucleoside intermediate (1 equiv), corresponding boronic acid (1.5 or 2 equiv), K_2CO_3 (2 equiv), $Pd(PPh_3)_4$ (0.05 equiv) or Na_2CO_3 (1.5 equiv.), TPPTS (0.06 equiv.) and $Pd(OAc)_2$ (0.025 equiv.). The mixture was heated to 100 °C from 10 min to 16 h. Solvent was co-evaporated with toluene and the crude mixture was purified by HPFC.

General Procedure B: Alkylation with phosphonomethyl tosylate

A solution of compounds **4a**–**j** and **10b,d,f,g** (1 equiv) in dry DMF at –20 °C was treated with phosphonomethyltosylate (1.7 equiv) and sodium hydride (60% in mineral oil, 4 equiv). The mixture was then let to warm to 22 °C for 1 or 2 h. Volatiles were removed in vacuo by co-distillation with toluene, and the crude mixture was purified by HPFC.

General Procedure C: Deprotection

A solution of compounds **5a–j** and **11b.d.f.g** (1 equiv) in dry acetonitrile at 22 °C was treated with 2,6-lutidin (10 equiv) and trimethylsilyl bromide (5 equiv) dropwise. After 1 day, MeOH/H_2O (8:2, 1 mL) mixture was added, and the mixture was stirred for an additional 30 min. Volatiles were removed in vacuo. The obtained oil was then treated with TFA (2 mL) at 22 °C for 40 min. The mixture was diluted with toluene, volatiles were removed in vacuo, and the residue was purified by RP-HPFC (C-18, H_2O/MeOH 0 $\rightarrow$100 %) and lyophilized (^{t}BuOH/H_2O).

Synthesis of DIANA probe precursor compound 1

First, acetonide-protected N^6-propargyl-adenosine (**S1**) was prepared according to a published procedure.[1] This intermediate was then phopshonylated using methylenebis(phosphonic dichloride) and the acetonide protecting group was removed to yield compound **1** as a probe precursor (Scheme S1).

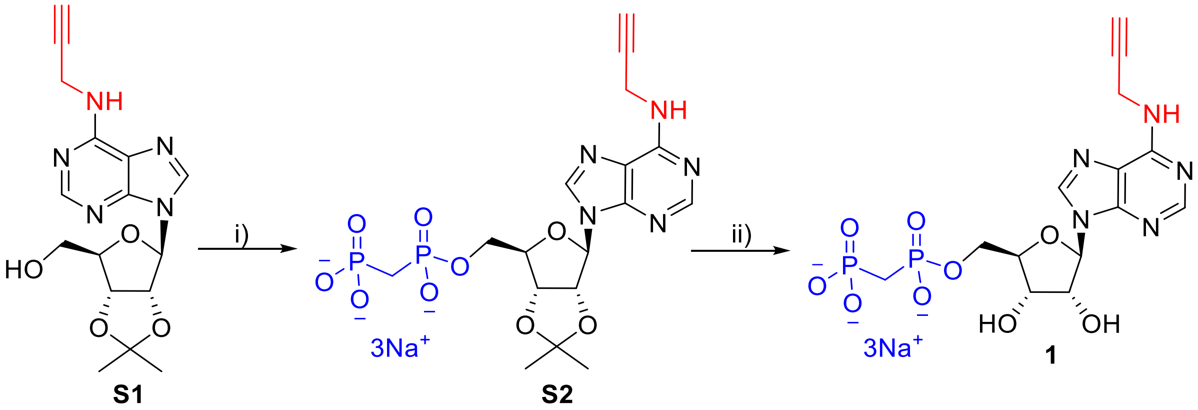

Scheme S1. Synthesis of DIANA probe precursor compound 1. i) Methylenebis(phosphonic dichloride), $PO(OMe)_3$, 0 °C, 40 h; 2) 2 M TEAB, 22 °C; 3) DOWEX 50; ii) 1) HCOOH:H_20 (10:1), 2.5 h, 22 °C; 2) DOWEX 50.

6-(6-(Prop-2-yn-1-ylamino)-7-(2,3-*O*-isopropylidene-*β*-D-ribofuranosyl)- 9*H*-purin-9-yl (S1)

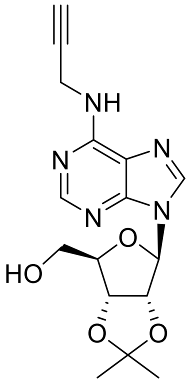

S1 was prepared according to a published procedure.[1] NMR spectra were in agreement with the literature.[1]

[(5-{[6-(Prop-2-yn-1-ylamino)-7-(2,3-*O*-isopropylidene-*β*-D-ribofuranosyl)-9*H*-purin-9-yl]-*β*-D-ribofuranosyl}oxy)phosphonomethyl]phosphonate trisodium salt (S2)

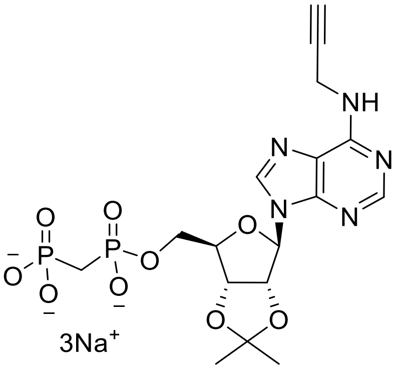

Methylenebis(phosphonic dichloride) (63 mg, 0.25 mmol) was dissolved in anhydrous $PO(OMe)_3$ (3 mL), and solution of **S1** (25 mg, 0.07 mmol,) in anhydrous $PO(OMe)_3$ (1 mL) was added over 16 h via a syringe pump. The resulting solution was stirred at room temperature for additional 24 hours. It was then treated with 2 M TEAB solution, diluted with water and lyophilized. The residue was dissolved in 0.1 M TEAB buffer and purified by RP-HPLC (gradient 2 $\rightarrow$ 40 % MeCN in 0.1M TEAB). Fractions containing the product were collected and lyophilized to obtain the desired bisphosphonate as a TEAB salt. It was then dissolved in Milli-Q H_2O and converted to Na^+ salt by passing through a column of DOWEX 50 in the Na^+ cycle. The collected product-containing fractions were lyophilized to yield **S2** as a white solid (10 mg, 24%). ^{1}H NMR (400 MHz, D_2O) δ 1.46 (s, 3H), 1.68 (s, 3H), 2.01 (t, 2H, *J* = 19.6 Hz), 2.62 (t, 1H, *J* = 2.4 Hz), 4.01 – 4.16 (m, 2H), 4.34 (s, 2H), 4.61 – 4.67 (m, 1H), 5.23 (dd, 1H, *J* = 6.2, 2.4 Hz), 5.42 (dd, 1H, *J* = 6.2, 3.4 Hz), 6.26 (d, 1H, *J* = 3.4 Hz), 8.29 (s, 1H), 8.45 (s, 1H); ^{13}C NMR (100 MHz, D_2O) δ 24.3, 26.1, 27.6 (d, *J* = 120 Hz), 28.8 (d, *J* = 120 Hz), 30.1, 63.8 (d, *J* = 5.2 Hz), 71.6, 79.9, 81.3, 83.8, 84.9 (d, *J* = 7.9 Hz), 89.9, 115.0, 119.2, 140.0, 148.2, 152.7, 153.9; ^{31}P NMR {^{1}H} (162 MHz, D_2O) δ 15.1 (d, *J* = 8.6 Hz), 23.8 (d, *J* = 8.6 Hz). HR-ESI-MS: *found*: 502.08970 ([M-H]$^-$, calcd for $C_{17}H_{22}N_5O_9P_2^-$: 502.08982).

[(5-{[6-(Prop-2-yn-1-ylamino)-9*H*-purin-9-yl]-*β*-D-ribofuranosyl}oxy)phosphonomethyl]phosphonate trisodium salt (1)

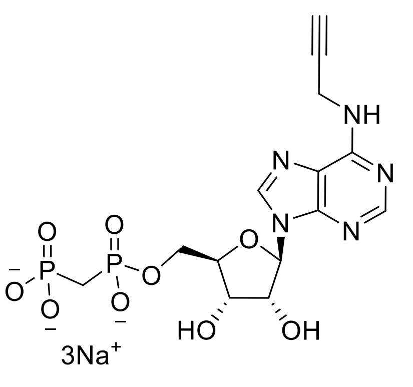

Protected nucleotide **S2** (10 mg, 19 μmol) was dissolved in HCOOH:H_2O / 10:1 and stirred at room temperature for 2.5 hours. The reaction mixture was then diluted with water and lyophilized. The residue was dissolved in 0.1 M TEAB buffer and purified by RP-HPLC (gradient 2 $\rightarrow$ 20 % MeCN in 0.1 M TEAB). Fractions containing the product were collected and

lyophilized to obtain the desired bisphosphonate as a TEAB salt. It was then dissolved in Milli-Q H_2O and converted to Na^+ salt by passing through a column of DOWEX 50 in Na^+ cycle. The collected product-containing fractions were lyophilized to yield **1** as white solid (7 mg, 75%). ^{1}H NMR (400 MHz, D_2O) δ 2.10 (t, 2H, *J* = 19.6 Hz), 2.62 (t, 1H, *J* = 2.4 Hz), 4.12 – 4.24 (m, 2H), 4.32 – 4.39 (m, 3H), 4.58 (t, 1H, *J* = 4.8 Hz), 6.13 (d, 1H, *J* = 5.2 Hz), 8.30 (s, 1H), 8.52 (s, 1H); ^{13}C NMR (100 MHz, D_2O) δ 27.7 (d, *J* = 120 Hz), 28.9 (d, *J* = 120 Hz), 30.1, 63.2 (d, *J* = 5.1 Hz), 70.0 71.5, 74.2, 80.0, 83.8 (d, *J* = 8.2 Hz), 87.0, 119.2, 139.8, 148.4, 152.7, 154.0; ^{31}P {^{1}H} NMR (162 MHz, D_2O) δ 15.0 (d, *J* = 9.5 Hz), 23.9 (d, *J* = 9.5 Hz). HR-ESI-MS: *found*: 462.05871([M-H] $^-$, calcd for $C_{14}H_{18}N_5O_9P_2^-$ 462.05852.

Synthesis of compounds for structure-activity relationship study

4-(Thiophen-3-yl)-7-(2′,3′-*O*-isopropylidene-5-*O*-tert-butyldimethylsilyl-*β*-D-ribofuranosyl)-7*H*-pyrrolo[2,3-*d*]pyrimidine (3a)

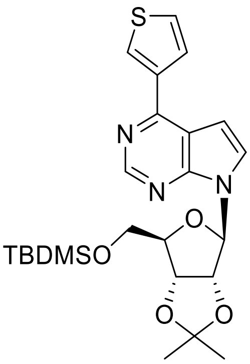

Nucleoside **3a** was prepared according to the general procedure A, from S**2** (300 mg, 0.68 mmol) and thiophene-3-boronic acid (131 mg, 1.02 mmol) in DMF (5 mL). HPFC (SiO_2, cyclohexane/EtOAc 0 → 50%) gave **3a** (316 mg, 95%) as a yellow oil. ^{1}H NMR (500.0 MHz, $CDCl_3$): 0.056, 0.061 (2 × s, 2 × 3H, CH_3Si); 0.90 (s, 9H, $(CH_3)_3C$); 1.39, 1.66 (2 × s, 2 × 3H, $(CH_3)_2C$); 3.80 (dd, 1H, J_{gem} = 11.2, $J_{5'b,4'}$ = 3.9, H-5′b); 3.89 (dd, 1H, J_{gem} = 11.2, $J_{5'a,4'}$ = 3.7, H-5′a); 4.35 (ddd, 1H, $J_{4',5'}$ = 3.9, 3.7, $J_{4',3'}$ = 3.1, H-4′); 4.99 (dd, 1H, $J_{3',2'}$ = 6.3, $J_{3',4'}$ = 3.1, H-3′); 5.14 (dd, 1H, $J_{2',3'}$ = 6.3, $J_{2',1'}$ = 3.0, H-2′); 6.47 (d, 1H, $J_{1',2'}$ = 3.0, H-1′); 6.83 (d, 1H, $J_{5,6}$ = 3.8, H-5); 7.47 (dd, 1H, $J_{5,4}$ = 5.1, $J_{5,2}$ = 3.0, H-5-thienyl); 7.55 (d, 1H, $J_{6,5}$ = 3.8, H-6); 7.87 (dd, 1H, $J_{4,5}$ = 5.1, $J_{4,2}$ = 1.3, H-4-thienyl); 8.16 (dd, 1H, $J_{2,5}$ = 3.0, $J_{2,4}$ = 1.3, H-2-thienyl); 8.91 (s, 1H, H-2). ^{13}C NMR (125.7 MHz, $CDCl_3$): –5.50, –5.38 (CH_3Si); 18.37 ($(CH_3)_3$**C**); 25.47 ((**C**$H_3)_2C$); 25.90 ((**C**$H_3)_3C$); 27.34 ((**C**$H_3)_2C$); 63.38(CH_2-5′); 80.92 (CH-3′); 84.94 (CH-2′); 86.05 (CH-4′); 90.24 (CH-1′); 101.12 (CH-5); 114.13 ($(CH_3)_2$**C**); 115.54 (C-4a); 126.29 (CH-5-thienyl); 126.75 (CH-6); 127.32

(CH-2-thienyl); 127.45 (CH-4-thienyl); 140.35 (C-3-thienyl); 151.55 (CH-2); 151.73 (C-7a); 152.52 (C-4). HR-ESI-MS: *found*: 488.2034 ([M + H]$^+$, calcd for $C_{24}H_{34}O_4N_3SSi^+$: 488.2033); HR-ESI-MS: *found*: 510.1852 ([M + Na]$^+$, calcd for $C_{24}H_{33}O_4N_3NaSSi^+$: 510.1853).

4-(Furan-2-yl)-7-(2′,3′-*O*-isopropylidene-5-*O*-tert-butyldimethylsilyl-*β*-D-ribofuranosyl)-7*H*-pyrrolo[2,3-*d*]pyrimidine (3b)

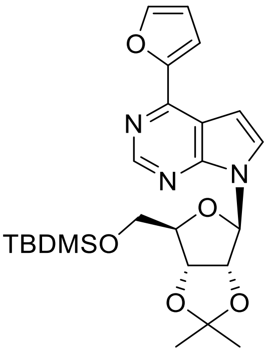

Nucleoside **3b** was prepared according to the general procedure A, from **2** (350 mg, 0.79 mmol) and furan-2-boronic acid (135 mg, 1.2 mmol) in DMF (6 mL). HPFC (SiO_2, cyclohexane/EtOAc 0 → 50%) gave **3b** (303 mg, 80%) as a yellow oil. ^{1}H NMR (500.0 MHz, $CDCl_3$): 0.061, 0.065 (2 × s, 2 × 3H, CH_3Si); 0.91 (s, 9H, $(CH_3)_3C$); 1.39, 1.66 (2 × s, 2 × 3H, $(CH_3)_2C$); 3.81, 3.89 (2 × dd, 2 × 1H, J_{gem} = 11.2, $J_{5',4'}$ = 3.8, H-5′); 4.34 (td, 1H, $J_{4',5'}$ = 3.8, $J_{4',3'}$ = 3.1, H-4′); 4.98 (dd, 1H, $J_{3',2'}$ = 6.3, $J_{3',4'}$ = 3.1, H-3′); 5.12 (dd, 1H, $J_{2',3'}$ = 6.3, $J_{2',1'}$ = 3.0, H-2′); 6.45 (d, 1H, $J_{1',2'}$ = 3.0, H-1′); 6.64 (dd, 1H, $J_{4,3}$ = 3.5, $J_{4,5}$ = 1.7, H-4-furyl); 7.05 (d, 1H, $J_{5,6}$ = 3.7, H-5); 7.44 (bs, 1H, H-3-furyl); 7.55 (d, 1H, $J_{6,5}$ = 3.7, H-6); 7.87 (dd, 1H, $J_{5,4}$ = 1.7, $J_{5,3}$ = 0.8, H-5-furyl); 8.86 (s, 1H, H-2). ^{13}C NMR (125.7 MHz, $CDCl_3$): –5.48, –5.36 (CH_3Si); 18.40 ($(CH_3)_3\mathbf{C}$); 25.49 ($(\mathbf{C}H_3)_2C$); 25.92 ($(\mathbf{C}H_3)_3C$); 27.36 ($(\mathbf{C}H_3)_2C$); 63.40 (CH_2-5′); 80.93 (CH-3′); 84.95 (CH-2′); 86.04 (CH-4′); 90.16 (CH-1′); 102.16 (CH-5); 112.39 (CH-4-furyl); 113.11 (b, CH-3-furyl); 113.57 (C-4a); 114.17 ($(CH_3)_2\mathbf{C}$); 126.92 (CH-6); 145.17 (CH-5-furyl); 147.04 (b, C-4); 151.32 (b, CH-2); 151.89 (C-7a); 152.95 (b, C-2-furyl). HR-ESI-MS: *found*: 472.2263 ([M + H]$^+$, calcd for $C_{24}H_{34}O_5N_3Si^+$: 472.2262); HR-ESI-MS: *found*: 494.2083 ([M + Na]$^+$, calcd for $C_{24}H_{33}O_5N_3NaSi^+$: 494.2081).

4-(Furan-3-yl)-7-(2′,3′-*O*-isopropylidene-5-*O*-tert-butyldimethylsilyl-*β*-D-ribofuranosyl)-7*H*-pyrrolo[2,3-*d*]pyrimidine (3c)

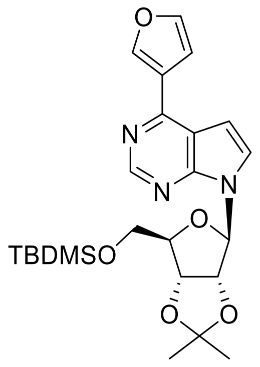

Nucleoside **3c** was prepared according to the general procedure A, from **2** (350 mg, 0.79 mmol) and furan-3-boronic acid (135 mg, 1.2 mmol) in DMF (6 mL). HPFC (SiO_2, cyclohexane/EtOAc 0 → 50%) gave **3c** (370 mg, 96%) as a yellow oil. ^{1}H NMR (500.2 MHz, $CDCl_3$, T = 60 °C): 0.068, 0.070 (2 × s, 2 × 3H, CH_3Si); 0.92 (s, 9H, $(CH_3)_3C$); 1.40, 1.66 (2 × s, 2 × 3H, $(CH_3)_2C$); 3.81, 3.89 (2 × dd, 2 × 1H, J_{gem} = 11.1, $J_{5',4'}$ = 4.1, H-5′); 4.33 (td, 1H, $J_{4',5'}$ = 4.1, $J_{4',3'}$ = 3.2, H-4′); 4.99 (dd, 1H, $J_{3',2'}$ = 6.3, $J_{3',4'}$ = 3.2, H-3′); 5.17 (dd, 1H, $J_{2',3'}$ = 6.3, $J_{2',1'}$ = 3.0, H-2′); 6.44 (d, 1H, $J_{1',2'}$ = 3.0, H-1′); 6.73 (d, 1H, $J_{5,6}$ = 3.8, H-5); 7.18 (d, 1H, $J_{4,5}$ = 1.8, H-4-furyl); 7.53 (d, 1H, $J_{6,5}$ = 3.8, H-6); 7.58 (t, 1H, $J_{5,2}$ = $J_{5,4}$ = 1.8, H-5-furyl); 8.31 (bs, 1H, H-2 furyl); 8.89 (s, 1H, H-2). ^{13}C NMR (125.8 MHz, $CDCl_3$, T = 60 °C): –5.45, –5.36 (CH_3Si); 18.40 ($(CH_3)_3\mathbf{C}$); 25.57 ($(\mathbf{C}H_3)_2C$); 25.94 ($(\mathbf{C}H_3)_3C$); 27.41 ($(\mathbf{C}H_3)_2C$); 63.52 (CH_2-5′); 81.18 (CH-3′); 85.02 (CH-2′); 86.36 (CH-4′); 90.59 (CH-1′); 100.89 (CH-5); 109.63 (CH-4-furyl); 114.30 ($(CH_3)_2\mathbf{C}$); 115.53 (C-4a); 125.05 (b, C-3-furyl); 127.07 (CH-6); 143.99 (CH-5-furyl); 144.21 (b, CH-2-furyl); 150.84 (b, C-4); 151.27 (b, CH-2); 151.60 (C-7a). HR-ESI-MS: *found*: 472.2270 ($[M + H]^+$, calcd for $C_{24}H_{34}O_5N_3Si^+$: 472.2262); HR-ESI-MS: *found*: 494.2088 ($[M + Na]^+$, calcd for $C_{24}H_{33}O_5N_3NaSi^+$: 494.2081).

4-(Thiophen-3-yl)-7-(2′,3′-*O*-isopropylidene-*β*-D-ribofuranosyl)-7*H*-pyrrolo[2,3-*d*]pyrimidine (4a)

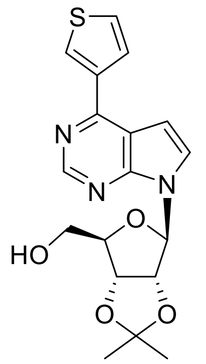

Compound **4a** was prepared according to the literature protocol.[2] **3a** (316 mg, 0.65 mmol) was treated with TBAF (1 M solution in THF, 1.1 mL, 1.04 mmol) at 22 °C for 20 min. The mixture was then diluted with EtOAc, washed with H_2O and the organic phase was dried over $MgSO_4$, filtered and concentrated in vacuo. HPFC (SiO_2, cyclohexane/EtOAc 0 → 50%) gave **4a** (210 mg, 86%) as a white foam. ^{1}H NMR (500.0 MHz, $CDCl_3$, T = 60 °C): 1.40, 1.66 (2 × s, 2 × 3H, $(CH_3)_2C$); 3.83 (dd, 1H, J_{gem} = 12.4, $J_{5'b,4'}$ = 2.4, H-5′b); 4.00 (dd, 1H, J_{gem} = 12.4, $J_{5'a,4'}$ = 2.0, H-5′a); 4.49 (ddd, 1H, $J_{4',5'}$ = 2.4, 2.0, $J_{4',3'}$ = 2.1, H-4′); 5.16 (dd, 1H, $J_{3',2'}$ = 6.2, $J_{3',4'}$ = 2.1, H-3′); 5.32 (dd, 1H, $J_{2',3'}$ = 6.2, $J_{2',1'}$ = 4.6, H-2′); 5.97 (d, 1H, $J_{1',2'}$ = 4.6, H-1′); 6.85 (d, 1H, $J_{5,6}$ = 3.7, H-5); 7.36 (d, 1H, $J_{6,5}$ = 3.7, H-6); 7.49 (dd, 1H, $J_{5,4}$ = 5.1, $J_{5,2}$ = 3.0, H-5-thienyl); 7.90 (dd, 1H, $J_{4,5}$ = 5.1, $J_{4,2}$ = 1.3, H-4-thienyl); 8.27 (bdd, 1H, $J_{2,5}$ = 3.0, $J_{2,4}$ = 1.3, H-2-thienyl); 8.88 (s, 1H, H-2). ^{13}C NMR (125.7 MHz, $CDCl_3$, T = 60 °C): 25.39, 27.66 ((**C**$H_3)_2C$); 63.42 (CH_2-5′); 81.55 (CH-3′); 83.37 (CH-2′); 85.93 (CH-4′); 95.41 (CH-1′); 101.08 (CH-5); 114.24 ($(CH_3)_2$**C**); 116.96 (C-4a); 126.59 (CH-5-thienyl); 127.59 (CH-4-thienyl); 128.49 (CH-2-thienyl); 129.41 (CH-6); 139.31 (C-3-thienyl); 150.60 (CH-2); 150.83 (C-7a); 153.18 (C-4). HR-ESI-MS: *found*: 374.1174 ($[M + H]^+$, calcd for $C_{18}H_{20}O_4N_3S^+$: 374.1169); HR-ESI-MS: *found*: 396.0993 ($[M + Na]^+$, calcd for $C_{18}H_{19}O_4N_3NaS^+$: 396.0988).

4-(Furan-2-yl)-7-(2′,3′-*O*-isopropylidene-*β*-D-ribofuranosyl)-7*H*-pyrrolo[2,3-*d*]pyrimidine (4b)

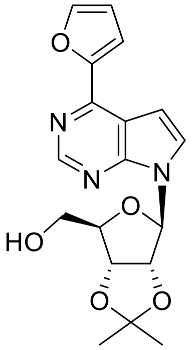

Nucleoside **4b** was prepared in the same manner as **4a** from corresponding **3b** (350 mg, 0.74 mmol) and TBAF (1 M solution in THF, 1.2 mL, 1.2 mmol). HPFC (SiO_2, cyclohexane/EtOAc 0 → 50%) gave **4b** (179 mg, 51%) as a yellowish oil. ^{1}H NMR (500.0 MHz, $CDCl_3$, T = 60 °C): 1.39, 1.66 (2 × s, 2 × 3H, $(CH_3)_2C$); 3.82 (dd, 1H, J_{gem} = 12.4, $J_{5'b,4'}$ = 2.4, H-5′b); 3.99 (dd, 1H, J_{gem} = 12.4, $J_{5'a,4'}$ = 2.0, H-5′a); 4.48 (ddd, 1H, $J_{4',5'}$ = 2.4, 2.0, $J_{4',3'}$ = 2.1, H-4′); 5.15 (dd, 1H, $J_{3',2'}$ = 6.1, $J_{3',4'}$ = 2.1, H-3′); 5.31 (dd, 1H, $J_{2',3'}$ = 6.1, $J_{2',1'}$ = 4.6, H-2′); 5.95 (d, 1H, $J_{1',2'}$ = 4.6, H-1′); 6.65 (dd,

1H, $J_{4,3}$ = 3.5, $J_{4,5}$ = 1.7, H-4-furyl); 7.06 (d, 1H, $J_{5,6}$ = 3.7, H-5); 7.32 (d, 1H, $J_{6,5}$ = 3.7, H-6); 7.44 (bd, 1H, $J_{3,4}$ = 3.5, H-3-furyl); 7.72 (dd, 1H, $J_{5,4}$ = 1.7, $J_{5,3}$ = 0.5, H-5-furyl); 8.81 (s, 1H, H-2). ^{13}C NMR (125.7 MHz, CDCl$_3$, T = 60 °C): 25.39, 27.86 ((**C**H$_3$)$_2$C); 63.42 (CH$_2$-5′); 81.50 (CH-3′); 83.36 (CH-2′); 85.88 (CH-4′); 95.41 (CH-1′); 102.00 (CH-5); 112.57 (CH-4-furyl); 114.18 (CH-3-furyl); 114.17 ((CH$_3$)$_2$**C**); 115.08 (C-4a); 129.33 (CH-6); 145.64 (CH-5-furyl); 147.91 (C-4); 150.64 (CH-2); 150.93 (C-7a); 152.81 (C-2-furyl). HR-ESI-MS: *found*: 358.1395 ([M + H]$^+$, calcd for $C_{18}H_{20}O_5N_3^+$: 358.1397).

4-(Furan-3-yl)-7-(2′,3′-*O*-isopropylidene-*β*-D-ribofuranosyl)-7*H*-pyrrolo[2,3-*d*]pyrimidine (4c)

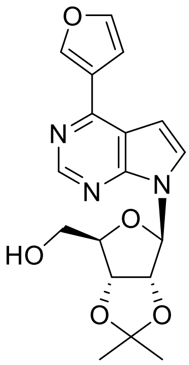

Nucleoside **4c** was prepared in the same manner as **4a** from corresponding **3c** (427 mg, 0.91 mmol) and TBAF (1 M solution in THF, 1.5 mL, 1.5 mmol). HPFC (SiO$_2$, cyclohexane/EtOAc 0 → 50%) gave **4c** (250 mg, 71%) as a yellowish oil. ^{1}H NMR (500.2 MHz, CDCl$_3$, T = 60 °C): 1.39, 1.66 (2 × s, 2 × 3H, (CH$_3$)$_2$C); 3.82 (dd, 1H, J_{gem} = 12.4, $J_{5'b,4'}$ = 2.5, H-5′b); 3.99 (dd, 1H, J_{gem} = 12.4, $J_{5'a,4'}$ = 2.1, H-5′a); 4.48 (dt, 1H, $J_{4',5'}$ = 2.5, 2.1, $J_{4',3'}$ = 2.1, H-4′); 5.15 (dd, 1H, $J_{3',2'}$ = 6.1, $J_{3',4'}$ = 2.1, H-3′); 5.32 (dd, 1H, $J_{2',3'}$ = 6.1, $J_{2',1'}$ = 4.6, H-2′); 5.93 (d, 1H, $J_{1',2'}$ = 4.6, H-1′); 6.71 (d, 1H, $J_{5,6}$ = 3.7, H-5); 7.15 (dd, 1H, $J_{4,5}$ = 2.0, $J_{4,2}$ = 0.8, H-4-furyl); 7.30 (d, 1H, $J_{6,5}$ = 3.7, H-6); 7.58 (dd, 1H, $J_{5,4}$ = 2.0, $J_{5,2}$ = 1.4, H-5-furyl); 8.26 (dd, 1H, $J_{2,5}$ = 1.4, $J_{2,4}$ = 0.8, H-2 furyl); 8.82 (s, 1H, H-2). ^{13}C NMR (125.8 MHz, CDCl$_3$, T = 60 °C): 25.39, 27.67 ((**C**H$_3$)$_2$C); 63.44 (CH$_2$-5′); 81.53 (CH-3′); 83.34 (CH-2′); 85.93 (CH-4′); 95.57 (CH-1′); 100.41 (CH-5); 109.61 (CH-4-furyl); 114.21 ((CH$_3$)$_2$**C**); 117.02 (C-4a); 125.30 (b, C-3-furyl); 129.02 (CH-6); 144.08 (CH-5-furyl); 144.28 (CH-2-furyl); 150.45 (C-4); 151.05 (CH-2); 152.19 (C-7a). HR-ESI-MS: *found*: 358.1395 ([M + H]$^+$, calcd for $C_{18}H_{20}O_5N_3^+$: 358.1397).

4-Chloro-7-(2′,3′-*O*-isopropylidene-*β*-D-ribofuranosyl)-7*H*-pyrrolo[2,3-*d*]pyrimidine (8)

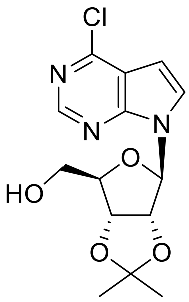

Compound **8** was prepared in the same manner as **4a** from fully protected nucleoside **2** (1.0 g, 2.27 mmol) and TBAF (1 M solution in THF, 3.7 mL, 3.67 mmol). HPFC (SiO_2, cyclohexane/EtOAc 0 → 40%) gave **8** (663 mg, 90%) as a colorless oil. ^{1}H NMR (500.0 MHz, $CDCl_3$): 1.37, 1.64 (2 × s, 2 × 3H, $(CH_3)_2C$); 3.81 (dd, 1H, J_{gem} = 12.6, $J_{5'b,4'}$ = 2.1, H-5′b); 3.97 (dd, 1H, J_{gem} = 12.6, $J_{5'a,4'}$ = 1.8, H-5′a); 4.49 (dt, 1H, $J_{4',5'}$ = 2.1, 1.8, $J_{4',3'}$ = 1.8, H-4′); 5.12 (dd, 1H, $J_{3',2'}$ = 6.1, $J_{3',4'}$ = 1.8, H-3′); 5.24 (dd, 1H, $J_{2',3'}$ = 6.1, $J_{2',1'}$ = 4.9, H-2′); 5.87 (d, 1H, $J_{1',2'}$ = 4.9, H-1′); 6.63 (d, 1H, $J_{5,6}$ = 3.7, H-5); 7.33 (d, 1H, $J_{6,5}$ = 3.7, H-6); 8.64 (s, 1H, H-2). ^{13}C NMR (125.7 MHz, $CDCl_3$): 25.25, 27.59 (($\mathbf{C}H_3)_2C$); 63.31 (CH_2-5′); 81.34 (CH-3′); 82.98 (CH-2′); 85.54 (CH-4′); 95.72 (CH-1′); 100.16 (CH-5); 114.23 ($(CH_3)_2\mathbf{C}$); 119.93 (C-4a); 129.66 (CH-6); 149.58 (C-7a); 150.34 (CH-2); 153.31 (C-4). HR-ESI-MS: *found*: 326.0899 ($[M + H]^+$, calcd for $C_{14}H_{17}O_4N_3Cl^+$: 326.0902); HR-ESI-MS: *found*: 348.0719899 ($[M + Na]^+$, calcd for $C_{14}H_{16}O_4N_3ClNa^+$: 348.0721).

4-(Benzofuran-2-yl)-7-(2′,3′-*O*-isopropylidene-*β*-D-ribofuranosyl)-7*H*-pyrrolo[2,3-*d*]pyrimidine (4d)

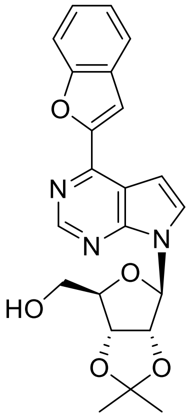

Nucleoside **4d** was prepared according to the general procedure A, from **8** (300 mg, 0.92 mmol) and benzofuran-2-boronic acid (224 mg, 1.38 mmol) in DMF (5 mL). HPFC (SiO_2, cyclohexane/EtOAc 0 → 50%) gave **4d** (331 mg, 88%) as a yellowish foam. ^{1}H NMR (500.2 MHz, $CDCl_3$): 1.39, 1.67 (2 × s, 2 × 3H, $(CH_3)_2C$); 3.84 (dd, 1H, J_{gem} = 12.6, $J_{5'b,4'}$ = 2.0, H-5′b); 4.02 (dd,

1H, J_{gem} = 12.6, $J_{5'a,4'}$ = 1.8, H-5′a); 4.53 (q, 1H, $J_{4',5'}$ = $J_{4',3'}$ = 1.8, H-4′); 5.16 (dd, 1H, $J_{3',2'}$ = 6.1, $J_{3',4'}$ = 1.8, H-3′); 5.33 (dd, 1H, $J_{2',3'}$ = 6.1, $J_{2',1'}$ = 4.9, H-2′); 5.93 (d, 1H, $J_{1',2'}$ = 4.9, H-1′); 7.22 (d, 1H, $J_{5,6}$ = 3.7, H-5); 7.32 (ddd, 1H, $J_{5,4}$ = 7.8, $J_{5,6}$ = 7.3, $J_{5,7}$ = 1.1, H-5-benzofuryl); 7.40 (bd, 1H, $J_{6,5}$ = 3.7, H-6); 7.43 (ddd, 1H, $J_{6,7}$ = 8.3, $J_{6,5}$ = 7.2, $J_{6,4}$ = 1.3, H-6-benzofuryl); 7.63 (bdq, 1H, $J_{7,6}$ = 8.3, $J_{7,5}$ = $J_{7,4}$ = $J_{7,3}$ = 0.8, H-7-benzofuryl); 7.73 (bdt, 1H, $J_{4,5}$ = 7.8, $J_{4,6}$ = $J_{4,7}$ = 1.0, H-4-benzofuryl); 7.85 (bs, 1H, H-3-benzofuryl); 8.88 (s, 1H, H-2). ^{13}C NMR (125.8 MHz, $CDCl_3$): 25.29, 27.64 ((**C**H_3)$_2$C); 63.42 (CH_2-5′); 81.48 (CH-3′); 82.94 (CH-2′); 85.58 (CH-4′); 95.73 (CH-1′); 102.02 (CH-5); 110.06 (CH-3-benzofuryl); 111.84 (CH-7-benzofuryl); 114.10 ((CH_3)$_2$**C**); 116.21 (C-4a); 122.43 (CH-4-benzofuryl); 123.67 (CH-5-benzofuryl); 126.59 (CH-6-benzofuryl); 128.03 (C-3a-benzofuryl); 130.06 (CH-6); 148.18 (C-4); 150.54 (CH-2); 150.82 (C-7a); 156.10 (C-7a-benzofuryl). *Carbon* C-2-benzofuryl *was not found.* HR-ESI-MS: *found*: 408.1552 ([M + H]$^+$, calcd for $C_{22}H_{22}O_5N_3^+$: 408.1554); HR-ESI-MS: *found*: 430.1371 ([M + Na]$^+$, calcd for $C_{22}H_{21}O_5N_3Na^+$: 430.1373).

4-Phenyl-7-(2′,3′-*O*-isopropylidene-*β*-D-ribofuranosyl)-7*H*-pyrrolo[2,3-*d*]pyrimidine (4e)

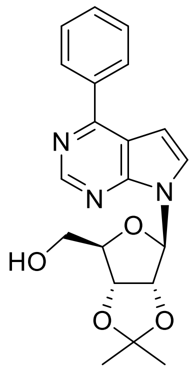

Nucleoside **4e** was prepared according to the general procedure A, from **8** (300 mg, 0.92 mmol) and phenylboronic acid (170 mg, 1.38 mmol) in DMF (5 mL). HPFC (SiO_2, cyclohexane/EtOAc 0 → 50%) gave **4e** (324 mg, 96%) as a white foam. ^{1}H NMR (500.2 MHz, $CDCl_3$): 1.39, 1.66 (2 × s, 2 × 3H, (CH_3)$_2$C); 3.86 (dd, 1H, J_{gem} = 12.6, $J_{5'b,4'}$ = 1.8, H-5′b); 4.01 (dd, 1H, J_{gem} = 12.6, $J_{5'a,4'}$ = 1.8, H-5′a); 4.53 (q, 1H, $J_{4',5'}$ = $J_{4',3'}$ = 1.8, H-4′); 5.17 (dd, 1H, $J_{3',2'}$ = 6.1, $J_{3',4'}$ = 1.7, H-3′); 5.35 (dd, 1H, $J_{2',3'}$ = 6.1, $J_{2',1'}$ = 4.9, H-2′); 5.91 (d, 1H, $J_{1',2'}$ = 4.9, H-1′); 6.83 (d, 1H, $J_{5,6}$ = 3.7, H-5); 7.35 (d, 1H, $J_{6,5}$ = 3.7, H-6); 7.50 – 7.60 (m, 3H, H-*m*,*p*-Ph); 8.08 (m, 2H, H-*o*-Ph); 8.94 (s, 1H, H-2). ^{13}C NMR (125.8 MHz, $CDCl_3$): 25.28, 27.64 ((**C**H_3)$_2$C); 63.46 (CH_2-5′); 81.50 (CH-3′); 82.85 (CH-2′); 85.54 (CH-4′); 95.88 (CH-1′); 101.12 (CH-5); 114.06 ((CH_3)$_2$**C**); 118.04 (C-4a); 128.91 and 128.98 (CH-*o*,*m*-Ph); 129.57 (CH-6); 130.50 (CH-*p*-Ph); 137.40 (C-*i*-

Ph); 150.28 (C-7a); 150.72 (CH-2); 158.63 (C-4). HR-ESI-MS: *found*: 368.1604 ([M + H]$^+$, calcd for $C_{20}H_{22}O_4N_3^+$: 368.1604); HR-ESI-MS: *found*: 390.1422 ([M + Na]$^+$, calcd for $C_{20}H_{21}O_4N_3Na^+$: 390.1424).

4-(Naphthalen-1-yl)-7-(2′,3′-*O*-isopropylidene-*β*-D-ribofuranosyl)-7*H*-pyrrolo[2,3-*d*]pyrimidine (4f)

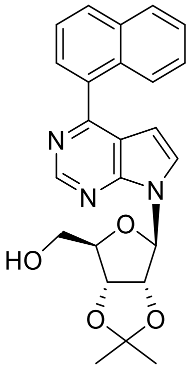

Nucleoside **4f** was prepared according to the general procedure A, from **8** (300 mg, 0.92 mmol) and naphthalene-1-boronic acid (318 mg 1.84 mmol) in DMF (5 mL). HPFC (SiO_2, cyclohexane/EtOAc 5 → 45%) gave **4f** (213 mg, 55%) as a white foam. ^{1}H NMR (500.0 MHz, $CDCl_3$): 1.41, 1.67 (2 × s, 2 × 3H, $(CH_3)_2C$); 3.87 (bdd, 1H, J_{gem} = 12.6, $J_{5'b,4'}$ = 2.0, H-5′b); 4.05 (dd, 1H, J_{gem} = 12.6, $J_{5'a,4'}$ = 1.8, H-5′a); 4.55 (dt, 1H, $J_{4',5'}$ = 2.0, 1.8, $J_{4',3'}$ = 1.8, H-4′); 5.19 (dd, 1H, $J_{3',2'}$ = 6.0, $J_{3',4'}$ = 1.8, H-3′); 5.40 (dd, 1H, $J_{2',3'}$ = 6.0, $J_{2',1'}$ = 5.0, H-2′); 5.94 (d, 1H, $J_{1',2'}$ = 5.0, H-1′); 6.41 (d, 1H, $J_{5,6}$ = 3.7, H-5); 7.30 (d, 1H, $J_{6,5}$ = 3.7, H-6); 7.47 (ddd, 1H, $J_{7,8}$ = 8.3, $J_{7,6}$ = 6.8, $J_{7,5}$ = 1.4, H-7-naphth); 7.54 (ddd, 1H, $J_{6,5}$ = 8.2, $J_{6,7}$ = 6.8, $J_{6,8}$ = 1.3, H-6-naphth); 7.61 (dd, 1H, $J_{3,4}$ = 8.2, $J_{3,2}$ = 7.1, H-3-naphth); 7.75 (dd, 1H, $J_{2,3}$ = 7.1, $J_{2,4}$ = 1.3, H-2-naphth); 7.95 (ddd, 1H, $J_{5,6}$ = 8.2, $J_{5,7}$ = 1.4, $J_{5,8}$ = 0.8, H-5-naphth); 8.01 (dd, 1H, $J_{4,3}$ = 8.2, $J_{4,2}$ = 1.3, $J_{4,8}$ = 0.8, H-4-naphth); 8.03 (ddt, 1H, $J_{8,7}$ = 8.3, $J_{8,6}$ = 1.3, $J_{8,4}$ = $J_{8,5}$ = 0.8, H-8-naphth); 9.04 (s, 1H, H-2). ^{13}C NMR (125.7 MHz, $CDCl_3$): 25.29, 27.66 ((**C**$H_3)_2C$); 63.47 (CH_2-5′); 81.51 (CH-3′); 82.87 (CH-2′); 85.51 (CH-4′); 95.83 (CH-1′); 101.47 (CH-5); 114.10 ($(CH_3)_2$**C**); 120.58 (C-4a); 125.14 (CH-3-naphth); 125.51 (CH-8-naphth); 126.27 (CH-6-naphth); 126.80 (CH-7-naphth); 128.32 (CH-2-naphth); 128.44 (CH-5-naphth); 129.43 (CH-6); 130.32 (CH-4-naphth); 130.74 (C-8a-naphth); 133.95 (C-1,4a-naphth); 149.88 (C-7a); 150.54 (CH-2); 159.80 (C-4). HR-ESI-MS: *found*: 418.1759 ([M + H]$^+$, calcd for $C_{24}H_{24}O_4N_3^+$: 418.1761); HR-ESI-MS: *found*: 440.1578 ([M + Na]$^+$, calcd for $C_{24}H_{23}O_4N_3Na^+$: 440.1580).

4-(Naphthalen-2-yl)-7-(2′,3′-*O*-isopropylidene-*β*-D-ribofuranosyl)-7*H*-pyrrolo[2,3-*d*]pyrimidine (4g)

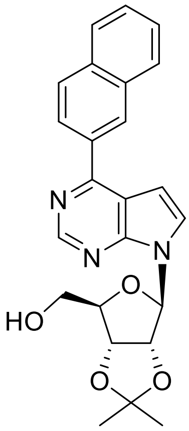

Nucleoside **4g** was prepared according to the general procedure A, from **8** (240 mg, 0.73 mmol) and naphthalen-2-boronic acid (254 mg 1.47 mmol) in DMF (5 mL). HPFC (SiO_2, cyclohexane/EtOAc 5 $\rightarrow$ 45%) gave crude **4g** (150 mg) as a white foam. Compound **4g** was used directly for the next step without further purification. HR-ESI-MS: *found*: 418.1760 ([M + H]$^+$, calcd for $C_{24}H_{24}O_4N_3^+$: 418.1761).

4-(Biphenyl-4-yl)-7-(2′,3′-*O*-isopropylidene-*β*-D-ribofuranosyl)-7*H*-pyrrolo[2,3-*d*]pyrimidine (4h)

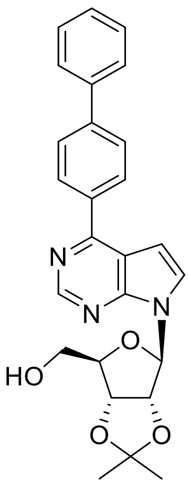

Nucleoside **4h** was prepared according to the general procedure A, from **8** (300 mg, 0.92 mmol) and biphenyl-4-boronic acid (366 mg 1.84 mmol) in DMF (5 mL). HPFC (SiO_2, cyclohexane/EtOAc 5 $\rightarrow$ 45%) gave **4h** (286 mg, 70%) as a white foam. ^{1}H NMR (500.0 MHz, $CDCl_3$): 1.39, 1.67 (2 × s, 2 × 3H, $(CH_3)_2C$); 3.85 (ddd, 1H, J_{gem} = 12.6, $J_{5'b,OH}$ = 11.4, $J_{5'b,4'}$ = 2.0, H-5′b); 4.02 (dt, 1H, J_{gem} = 12.6, $J_{5'a,OH}$ = $J_{5'a,4'}$ = 1.7, H-5′a); 4.54 (d, 1H, $J_{4',5'}$ = 2.0, 1.7, $J_{4',3'}$ = 1.7,

H-4′); 5.18 (dd, 1H, $J_{3',2'}$ = 6.0, $J_{3',4'}$ = 1.7, H-3′); 5.37 (dd, 1H, $J_{2',3'}$ = 6.0, $J_{2',1'}$ = 5.0, H-2′); 5.90 (d, 1H, $J_{1',2'}$ = 5.0, H-1′); 6.06 (bdd, 1H, $J_{OH,5'}$ = 11.4, 1.7, OH-5′); 6.88 (d, 1H, $J_{5,6}$ = 3.7, H-5); 7.34 (d, 1H, $J_{6,5}$ = 3.7, H-6); 7.40 (m, 1H, H-*p*-Ph); 7.47–7.51 (m, 2H, H-*m*-Ph); 7.66–7.70 (m, 2H, H-*o*-Ph); 7.77–7.81 (m, 2H, H-*m*-phenylene); 8.16–8.20 (m, 2H, H-*o*-phenylene); 8.94 (s, 1H, H-2). ^{13}C NMR (125.7 MHz, $CDCl_3$): 25.29, 27.67 ((**C**H_3)$_2$C); 63.51 (CH_2-5′); 81.53 (CH-3′); 82.74 (CH-2′); 85.49 (CH-4′); 96.10 (CH-1′); 101.02 (CH-5); 114.03 ((CH_3)$_2$**C**); 118.07 (C-4a); 127.21 (CH-*o*-Ph); 127.58 (CH-*m*-phenylene); 127.85 (CH-*p*-Ph); 128.91 (CH-*m*-Ph); 129.39 (CH-*o*-phenylene); 129.53 (CH-6); 136.56 (C-*i*-phenylene); 140.29 (C-*i*-Ph); 143.22 (C-*p*-phenylene); 150.24 (C-7a); 150.89 (CH-2); 158.40 (C-4). HR-ESI-MS: *found*: 444.1920 ($[M + H]^+$, calcd for $C_{26}H_{26}O_4N_3^+$: 444.1917); HR-ESI-MS: *found*: 466.1738 ($[M + Na]^+$, calcd for $C_{26}H_{25}O_4N_3Na^+$: 466.1737).

4-(Phenanthren-9-yl)-7-(2′,3′-*O*-isopropylidene-*β*-D-ribofuranosyl)-7*H*-pyrrolo[2,3-*d*]pyrimidine (4i)

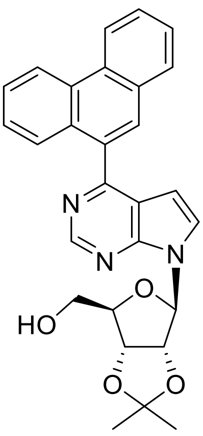

Nucleoside **4i** was prepared according to the general procedure A, from **8** (350 mg, 1.07 mmol) and phenanthrene-9-boronic acid (478 mg 2.14 mmol) in DMF (5 mL). HPFC (SiO_2, cyclohexane/EtOAc 5 $\rightarrow$ 45%) gave **4i** (488 mg, 94%) as a white foam. ^{1}H NMR (500.0 MHz, $CDCl_3$) : 1.41, 1.68 (2 × s, 2 × 3H, (CH_3)$_2$C); 3.88 (dd, 1H, J_{gem} = 12.6, $J_{5'b,4'}$ = 2.1, H-5′b); 4.06 (dd, 1H, J_{gem} = 12.6, $J_{5'a,4'}$ = 1.8, H-5′a); 4.56 (q, 1H, $J_{4',3'}$ = $J_{4',5'}$ = 1.8, H-4′); 5.20 (dd, 1H, $J_{3',2'}$ = 6.0, $J_{3',4'}$ = 1.7, H-3′); 5.40 (dd, 1H, $J_{2',3'}$ = 6.0, $J_{2',1'}$ = 4.9, H-2′); 5.97 (d, 1H, $J_{1',2'}$ = 4.8, H-1′); 6.42 (d, 1H, $J_{5,6}$ = 3.7, H-5); 7.33 (d, 1H, $J_{6,5}$ = 3.7, H-6); 7.55 (ddd, 1H, $J_{7,8}$ = 8.3, $J_{7,6}$ = 7.0, $J_{7,5}$ = 1.2, H-7-phenanthryl); 7.65 (ddd, 1H, $J_{2,1}$ = 8.0, $J_{2,3}$ = 7.0, $J_{2,4}$ = 1.2, H-2-phenanthryl); 7.71 (ddd, 1H, $J_{6,5}$= 8.4, $J_{6,7}$ = 7.0, $J_{6,8}$ = 1.3, H-6-phenanthryl); 7.75 (ddd, 1H, $J_{3,4}$ = 8.4, $J_{3,2}$ = 7.0, $J_{3,1}$ = 1.4, H-3-phenanthryl); 7.96 (dd, 1H, $J_{1,2}$ = 7.9, $J_{1,3}$ = 1.5, H-1-phenanthryl); 8.01 (bdd, 1H, $J_{8,7}$ = 8.3, $J_{8,6}$

= 1.2, H-8-phenanthryl); 8.03 (s, 1H, H-10-phenanthryl); 8.76 (bd, 1H, $J_{4,3}$ = 8.4, H-4-phenanthryl); 8.80 (dm, 1H, $J_{5,6}$ = 8.4, H-5-phenanthryl); 9.08 (s, 1H, H-2). ^{13}C NMR (125.7 MHz, $CDCl_3$): 25.30, 27.65 ((**C**H_3)$_2$C); 63.45 (CH_2-5′); 81.51 (CH-3′); 82.95 (CH-2′); 86.55 (CH-4′); 96.70 (CH-1′); 101.61 (CH-5); 114.13 ((CH_3)$_2$**C**); 120.64 (C-4a); 122.66 (CH-4-phenanthryl); 123.03 (CH-5-phenanthryl); 126.40 (CH-8-phenanthryl); 126.89 (CH-7-phenanthryl); 127.05, 127.07 (CH-2,6-phenanthryl); 127.89 (CH-3-phenanthryl); 129.34 (C-8a-phenanthryl); 129.39 (CH-1-phenanthryl); 129.59 (CH-6); 129.87 (CH-10-phenanthryl); 130.84, 130.87 (C-4a,4b-phenanthryl); 131.00 (C-10a-phenanthryl); 132.82 (C-9-phenanthryl); 149.93 (C-7a); 150.41 (CH-2); 159.64 (C-4). HR-ESI-MS: *found*: 468.1918 ([M + H]$^+$, calcd for $C_{28}H_{26}O_4N_3^+$: 468.1917).

4-(Dibenzofuran-4-yl)-7-(2′,3′-*O*-isopropylidene-*β*-D-ribofuranosyl)-7*H*-pyrrolo[2,3-*d*]pyrimidine (4j)

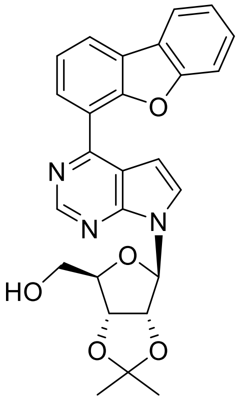

Nucleoside **4j** was prepared according to the general procedure A, from **8** (300 mg, 0.92 mmol) and dibenzofuran-4-boronic acid (391 mg 1.84 mmol) in DMF (5 mL). HPFC (SiO_2, cyclohexane/EtOAc 5 $\rightarrow$ 45%) gave **4j** (351 mg, 83%) as a white foam. ^{1}H NMR (500.0 MHz, $CDCl_3$): 1.41, 1.68 (2 × s, 2 × 3H, (CH_3)$_2$C); 3.87, 4.04 (2 × dd, 1H, J_{gem} = 12.5, $J_{5',4'}$ = 1.8, H-5′); 4.55 (q, 1H, $J_{4',3'}$ = $J_{4',5'}$ = 1.8, H-4′); 5.19 (dd, 1H, $J_{3',2'}$ = 6.0, $J_{3',4'}$ = 1.8, H-3′); 5.38 (dd, 1H, $J_{2',3'}$ = 6.0, $J_{2',1'}$ = 5.0, H-2′); 5.98 (bd, 1H, $J_{1',2'}$ = 5.0, H-1′); 6.75 (d, 1H, $J_{5,6}$ = 3.7, H-5); 7.38–7.42 (m, 2H, H-6, H-8-dibenzofuryl); 7.49 (ddd, 1H, $J_{7,6}$ = 8.3, $J_{7,8}$ = 7.2, $J_{7,9}$ = 1.4, H-7-dibenzofuryl); 7.56 (t, 1H, $J_{2,1}$ = $J_{2,3}$ = 7.6, H-2-dibenzofuryl); 7.57 (dt, 1H, $J_{6,7}$ = 8.3, $J_{6,8}$ = $J_{6,9}$ = 1.0, H-6-dibenzofuryl); 8.00–8.05 (m, 2H, H-3,9-dibenzofuryl); 8.14 (dd, 1H, $J_{1,2}$ = 7.6, $J_{1,3}$ = 1.3, H-1-dibenzofuryl); 9.07 (s, 1H, H-2). ^{13}C NMR (125.7 MHz, $CDCl_3$): 25.30, 27.64 ((**C**H_3)$_2$C); 63.44 (CH_2-5′); 81.47 (CH-3′); 83.00 (CH-2′); 85.58 (CH-4′); 95.61 (b, CH-1′); 102.43 (b, CH-5); 111.94 (CH-6-dibenzofuryl); 114.12 ((CH_3)$_2$**C**); 119.56 (C-4a); 120.78 (CH-9-dibenzofuryl); 121.70 (C-4-dibenzofuryl); 122.91

(b, CH-1-dibenzofuryl); 123.21 (CH-8-dibenzofuryl); 123.34 (CH-2-dibenzofuryl); 123.69 (C-9a-dibenzofuryl); 125.52 (C-9b-dibenzofuryl); 127.67 (CH-7-dibenzofuryl); 128.60 (CH-3-dibenzofuryl); 129.51 (CH-6); 150.14 (C-7a); 150.34 (b, CH-2); 153.36 (C-4a-dibenzofuryl); 155.00 (C-4); 156.11 (C-5a-dibenzofuryl). HR-ESI-MS: *found*: 458.1708 ($[M + H]^+$, calcd for $C_{26}H_{24}O_5N_3^+$: 458.1710); HR-ESI-MS: *found*: 480.1525 ($[M + Na]^+$, calcd for $C_{26}H_{23}O_5N_3Na^+$: 480.1529).

Diisopropyl[(5-{2′,3′-*O*-isopropylidene-[4-(thiophen-3-yl)-7*H*-pyrrolo[2,3-*d*]pyrimidin-7-yl]-*β*-D-ribofuranosyl}oxy)methyl]phosphonate (5a) and 4-(Thiophen-3-yl)-7-(5′-deoxy-4′,5′-didehydro-2′,3′-*O*-isopropylidene-*β*-D-ribofuranosyl)-7*H*-pyrrolo[2,3-*d*]pyrimidine (6a)

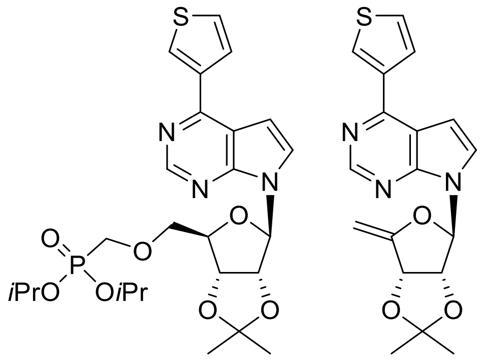

5a **6a**

Compound **5a** was prepared according to the general procedure B from **4a** (200 mg, 0.53 mmol) in dry DMF (6 mL). After 1 h, volatiles were removed in vacuo by co-distillation with toluene. HPFC (SiO_2, cyclohexane/EtOAc 0 → 80%) gave side product of elimination **6a** (40 mg, 21%) as a yellow oil and the desired product **5a** as a white oil (75 mg, 26%). Data for **6a**: ^{1}H NMR (500.0 MHz, $CDCl_3$): 1.44, 1.61 (2 × q, 2 × 3H, ^4J = 0.7, $(CH_3)_2C$); 4.47, 4.61 (2 × dd, 2 × 1H, J_{gem} = 2.4, $J_{5',3'}$ = 1.1, H-5′); 5.28 (dd, 1H, $J_{2',3'}$ = 6.1, $J_{2',1'}$ = 1.0, H-2′); 5.57 (dt, 1H, $J_{3',2'}$ = 6.1, $J_{3',5'}$ = 1.1, H-3′); 6.45 (d, 1H, $J_{1',2'}$ = 1.0, H-1′); 6.86 (d, 1H, $J_{5,6}$ = 3.8, H-5); 7.26 (d, 1H, $J_{6,5}$ = 3.8, H-6); 7.49 (dd, 1H, $J_{5,4}$ = 5.1, $J_{5,2}$ = 3.0, H-5-thienyl); 7.87 (dd, 1H, $J_{4,5}$ = 5.1, $J_{4,2}$ = 1.3, H-4-thienyl); 8.19 (bdd, 1H, $J_{2,5}$ = 3.0, $J_{2,4}$ = 1.3, H-2-thienyl); 8.89 (s, 1H, H-2). ^{13}C NMR (125.7 MHz, $CDCl_3$): 25.69, 26.84 ((**C**$H_3)_2C$); 79.79 (CH-3′); 83.21 (CH-2′); 87.71 (CH_2-5′); 91.60 (CH-1′); 101.72 (CH-5); 114.06 ($(CH_3)_2$**C**); 115.59 (C-4a); 126.55 (CH-5-thienyl); 127.20 (CH-6); 127.40 (CH-4-thienyl); 127.80 (CH-2-thienyl); 139.86 (C-3-thienyl); 151.44 (C-7a); 151.54 (CH-2); 152.76 (C-4); 161.94 (C-4′). HR-ESI-MS: *found*: 356.1059 ($[M + H]^+$, calcd for $C_{18}H_{18}O_3N_3S^+$:

356.1063). Data for **5a**: ^{1}H NMR (500.0 MHz, DMSO-d_6): 1.20, 1.21, 1.22, 1.23 (4 × d, 4 × 3H, J_{vic} = 6.0, (C**H$_3$**)$_2$CH); 1.33, 1.57 (2 × s, 2 × 3H, $(CH_3)_2C$); 3.68 (dd, 1H, J_{gem} = 10.6, $J_{5'b,4'}$ = 5.3, H-5′b); 3.77 (dd, 1H, J_{gem} = 10.6, $J_{5'a,4'}$ = 4.4, H-5′a); 3.79 (d, 2H, $J_{H,P}$ = 8.0, CH_2P); 4.31 (ddd, 1H, $J_{4',5'}$ = 5.3, 4.4, $J_{4',3'}$ = 3.0, H-4′); 4.53 – 4.64 (m, 2H, (CH$_3$)$_2$C**H**); 4.97 (dd, 1H, $J_{3',2'}$ = 6.3, $J_{3',4'}$ = 3.0, H-3′); 5.25 (dd, 1H, $J_{2',3'}$ = 6.3, $J_{2',1'}$ = 3.3, H-2′); 6.42 (d, 1H, $J_{1',2'}$ = 3.3, H-1′); 7.17 (d, 1H, $J_{5,6}$ = 3.9, H-5); 7.76 (dd, 1H, $J_{5,4}$ = 5.1, $J_{5,2}$ = 2.9, H-5-thienyl); 7.93 (d, 1H, $J_{6,5}$ = 3.9, H-6); 7.96 (dd, 1H, $J_{4,5}$ = 5.1, $J_{4,2}$ = 1.3, H-4-thienyl); 8.58 (dd, 1H, $J_{2,5}$ = 2.9, $J_{2,4}$ = 1.3, H-2-thienyl); 8.85 (s, 1H, H-2). ^{13}C NMR (125.7 MHz, DMSO-d_6): 23.87 (d, $J_{C,P}$ = 4.5, (**C**H$_3$)$_2$CH); 24.00 (d, $J_{C,P}$ = 3.6, (**C**H$_3$)$_2$CH); 25.40, 27.32 ((**C**H$_3$)$_2$C); 65.34 (d, $J_{C,P}$ = 164.1, CH_2P); 70.37 (d, $J_{C,P}$ = 6.3, (CH$_3$)$_2$**C**H); 72.69 (d, $J_{C,P}$ = 11.3, CH$_2$-5′); 81.33 (CH-3′); 83.56 (CH-4′); 83.66 (CH-2′); 88.94 (CH-1′); 101.70 (CH-5); 113.63 ((CH$_3$)$_2$**C**); 114.55 (C-4a); 127.44 (CH-5-thienyl); 127.58 (CH-4-thienyl); 128.43 (CH-6); 129.08 (CH-2-thienyl); 139.69 (C-3-thienyl); 151.21 (CH-2); 151.64 (C-4); 151.78 (C-7a). ^{31}P{^{1}H} NMR (202.4 MHz, DMSO-d_6): 20.59. HR-ESI-MS: *found*: 552.1923 ([M + H]$^+$, calcd for $C_{25}H_{35}O_7N_3PS^+$: 552.1927); HR-ESI-MS: *found*: 574.1740 ([M + Na]$^+$, calcd for $C_{25}H_{34}O_7N_3NaPS^+$: 574.1747).

Diisopropyl[(5-{2′,3′-*O*-isopropylidene-[4-(furan-2-yl)-7*H*-pyrrolo[2,3-*d*]pyrimidin-7-yl]-*β*-D-ribofuranosyl}oxy)methyl]phosphonate (5b)

4-(Furan-2-yl)-7-(5′-deoxy-4′,5′-didehydro-2′,3′-*O*-isopropylidene-*β*-D-ribofuranosyl)-7*H*-pyrrolo[2,3-*d*]pyrimidine (6b)

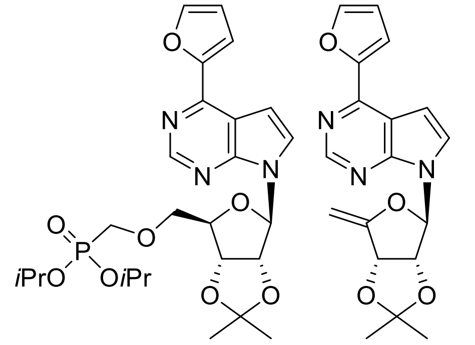

5b **6b**

Compound **5b** was prepared according to the general procedure B from **4b** (170 mg, 0.47 mmol) in dry DMF (5 mL). After 1 h, volatiles were removed in vacuo by co-distillation with toluene. HPFC (SiO_2, cyclohexane/EtOAc 0 → 80%) gave side product of elimination **6b** (25 mg, 16%) as a brown oil and the desired product **5b** as a yellow oil (80 mg, 32%). Data for **6b**: ^{1}H NMR (500.2 MHz, $CDCl_3$): 1.43, 1.60 (2 × s, 2 × 3H, $(CH_3)_2C$); 4.45 (dd, 1H, J_{gem} = 2.4, $J_{5'b,3'}$

= 1.0, H-5′b); 4.59 (dd, 1H, J_{gem} = 2.4, $J_{5'a,3'}$ = 1.2, H-5′a); 5.22 (dd, 1H, $J_{2',3'}$ = 6.1, $J_{2',1'}$ = 1.1, H-2′); 5.55 (dt, 1H, $J_{3',2'}$ = 6.1, $J_{3',5'a}$ = $J_{3',5'b}$ = 1.1, H-3′); 6.43 (d, 1H, $J_{1',2'}$ = 1.1, H-1′); 6.62 (dd, 1H, $J_{4,3}$ = 3.5, $J_{4,5}$ = 1.8, H-4-furyl); 7.04 (d, 1H, $J_{5,6}$ = 3.7, H-5); 7.21 (d, 1H, $J_{6,5}$ = 3.7, H-6); 7.39 (dd, 1H, $J_{3,4}$ = 3.5, $J_{3,5}$ = 0.9, H-3-furyl); 7.70 (dd, 1H, $J_{5,4}$ = 1.8, $J_{5,3}$ = 0.9, H-5-furyl); 8.82 (s, 1H, H-2). ^{13}C NMR (125.8 MHz, $CDCl_3$): 25.69, 26.83 ((**C**H_3)$_2$C); 79.78 (CH-3′); 83.17 (CH-2′); 87.67 (CH_2-5′); 91.44 (CH-1′); 102.57 (CH-5); 112.37 (CH-4-furyl); 113.11 (CH-3-furyl); 113.66 (C-4a); 114.01 ((CH_3)$_2$**C**); 127.04 (CH-6); 145.22 (CH-5-furyl); 147.63 (C-4); 151.62 (C-7a); 151.68 (CH-2); 153.04 (C-2-furyl); 161.95 (C-4′). HR-ESI-MS: *found*: 340.1293 ([M + H]$^+$, calcd for $C_{18}H_{18}O_4N_3^+$: 340.1291). Data for **5b**: ^{1}H NMR (500.0 MHz, DMSO-d_6): 1.20, 1.21, 1.22, 1.23 (4 × d, 4 × 3H, J_{vic} = 6.0, (C**H₃**)$_2$CH); 1.33, 1.57 (2 × s, 2 × 3H, (CH_3)$_2$C); 3.68 (dd, 1H, J_{gem} = 10.6, $J_{5'b,4'}$ = 5.2, H-5′b); 3.76 (dd, 1H, J_{gem} = 10.6, $J_{5'a,4'}$ = 4.3, H-5′a); 3.79 (d, 2H, $J_{H,P}$ = 8.1, CH_2P); 4.32 (ddd, 1H, $J_{4',5'}$ = 5.2, 4.3, $J_{4',3'}$ = 3.0, H-4′); 4.53 – 4.64 (m, 2H, (CH_3)$_2$C**H**); 4.96 (dd, 1H, $J_{3',2'}$ = 6.2, $J_{3',4'}$ = 3.0, H-3′); 5.24 (dd, 1H, $J_{2',3'}$ = 6.2, $J_{2',1'}$ = 3.3, H-2′); 6.41 (d, 1H, $J_{1',2'}$ = 3.3, H-1′); 6.80 (dd, 1H, $J_{4,3}$ = 3.5, $J_{4,5}$ = 1.7, H-4-furyl); 7.08 (d, 1H, $J_{5,6}$ = 3.8, H-5); 7.49 (dd, 1H, $J_{3,4}$ = 3.5, $J_{3,5}$ = 0.9, H-3-furyl); 7.91 (d, 1H, $J_{6,5}$ = 3.8, H-6); 8.08 (dd, 1H, $J_{5,4}$ = 1.7, $J_{5,3}$ = 0.9, H-5-furyl); 8.80 (s, 1H, H-2). ^{13}C NMR (125.7 MHz, DMSO-d_6): 23.86 (d, $J_{C,P}$ = 4.5, (**C**H_3)$_2$CH); 23.99 (d, $J_{C,P}$ = 3.6, (**C**H_3)$_2$CH); 25.39, 27.31 ((**C**H_3)$_2$C); 65.35 (d, $J_{C,P}$ = 164.2, CH_2P); 70.36 (d, $J_{C,P}$ = 6.2, (CH_3)$_2$**C**H); 72.69 (d, $J_{C,P}$ = 11.4, CH_2-5′); 81.30 (CH-3′); 83.60 (CH-4′); 83.70 (CH-2′); 88.98 (CH-1′); 101.86 (CH-5); 112.67 (C-4a); 112.91 (CH-4-furyl); 113.62 ((CH_3)$_2$**C**); 113.67 (CH-3-furyl); 128.53 (CH-6); 146.65 (C-4); 146.69 (CH-5-furyl); 151.37 (CH-2); 151.83 (C-7a); 152.47 (C-2-furyl). ^{31}P{^{1}H} NMR (202.4 MHz, DMSO-d_6): 20.60. HR-ESI-MS: *found*: 536.2155 ([M + H]$^+$, calcd for $C_{25}H_{35}O_8N_3P^+$: 536.2156); HR-ESI-MS: *found*: 558.1975 ([M + Na]$^+$, calcd for $C_{25}H_{34}O_8N_3NaP^+$: 558.1975).

Diisopropyl[(5-{2′,3′-*O*-isopropylidene-[4-(furan-3-yl)-7*H*-pyrrolo[2,3-*d*]pyrimidin-7-yl]-*β*-D-ribofuranosyl}oxy)methyl]phosphonate (5c)

4-(Furan-3-yl)-7-(5′-deoxy-4′,5′-didehydro-2′,3′-*O*-isopropylidene-*β*-D-ribofuranosyl)-7*H*-pyrrolo[2,3-*d*]pyrimidine (6c)

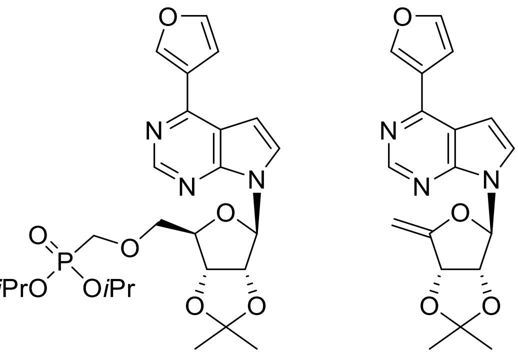

5c **6c**

Compound **5c** was prepared according to the general procedure B from **4c** (250 mg, 0.70 mmol) in dry DMF (7.5 mL). After 1 h, volatiles were removed in vacuo by co-distillation with toluene. HPFC (SiO_2, cyclohexane/EtOAc 0 $\rightarrow$ 80%) gave a side product of elimination **6c** (55 mg, 23%) as a brown oil and the desired product **5c** as a brown oil (74 mg, 20%). Data for **6c**: ^{1}H NMR (500.2 MHz, $CDCl_3$): 1.43, 1.59 (2 × s, 2 × 3H, $(CH_3)_2C$); 4.45 (dd, 1H, J_{gem} = 2.4, $J_{5'b,3'}$ = 0.9, H-5′b); 4.59 (dd, 1H, J_{gem} = 2.4, $J_{5'a,3'}$ = 1.2, H-5′a); 5.26 (dd, 1H, $J_{2',3'}$ = 6.1, $J_{2',1'}$ = 1.2, H-2′); 5.55 (dt, 1H, $J_{3',2'}$ = 6.1, $J_{3',5'a}$ = $J_{3',5'b}$ = 1.1, H-3′); 6.42 (d, 1H, $J_{1',2'}$ = 1.2, H-1′); 6.70 (d, 1H, $J_{5,6}$ = 3.8, H-5); 7.11 (dd, 1H, $J_{4,5}$ = 1.9, $J_{4,2}$ = 0.9, H-4-furyl); 7.21 (d, 1H, $J_{6,5}$ = 3.8, H-6); 7.56 (t, 1H, $J_{5,4}$ = $J_{5,2}$ = 1.7, H-5-furyl); 8.22 (dd, 1H, $J_{2,5}$ = 1.5, $J_{2,4}$ = 0.9, H-2-furyl); 8.83 (s, 1H, H-2). ^{13}C NMR (125.8 MHz, $CDCl_3$): 25.67, 26.80 ((**C**$H_3)_2C$); 79.76 (CH-3′); 83.14 (CH-2′); 87.68 (CH_2-5′); 91.50 (CH-1′); 101.11 (CH-5); 109.40 (CH-4-furyl); 114.00 ($(CH_3)_2$**C**); 115.46 (C-4a); 125.31 (C-3-furyl); 126.85 (CH-6); 143.84 (CH-2-furyl); 143.97 (CH-5-furyl); 151.06 and 151.33 (C-4,7a); 151.76 (CH-2); 161.94 (C-4′). HR-ESI-MS: *found*: 340.1293 ($[M + H]^+$, calcd for $C_{18}H_{18}O_4N_3^+$: 340.1291). Data for **5c**: ^{1}H NMR (500.0 MHz, DMSO-d_6): 1.20, 1.21, 1.22, 1.23 (4 × d, 4 × 3H, J_{vic} = 6.0, (C**H₃**)₂CH); 1.33, 1.57 (2 × s, 2 × 3H, $(CH_3)_2C$); 3.68 (dd, 1H, J_{gem} = 10.6, $J_{5'b,4'}$ = 5.3, H-5′b); 3.76 (dd, 1H, J_{gem} = 10.6, $J_{5'a,4'}$ = 4.4, H-5′a); 3.79 (d, 2H, $J_{H,P}$ = 8.0, CH_2P); 4.30 (ddd, 1H, $J_{4',5'}$ = 5.3, 4.4, $J_{4',3'}$ = 3.0, H-4′); 4.52 – 4.64 (m, 2H, $(CH_3)_2C$**H**); 4.96 (dd, 1H, $J_{3',2'}$ = 6.2, $J_{3',4'}$ = 3.0, H-3′); 5.25 (dd, 1H, $J_{2',3'}$ = 6.2, $J_{2',1'}$ = 3.3, H-2′); 6.40 (d, 1H, $J_{1',2'}$ = 3.3, H-1′); 7.12 (d, 1H, $J_{5,6}$ = 3.8, H-5); 7.26 (dd, 1H, $J_{4,5}$ = 1.9, $J_{4,2}$ = 0.8, H-4-furyl); 7.88 (d, 1H, $J_{6,5}$ = 3.8, H-6); 7.91 (dd, 1H, $J_{5,4}$ = 1.9, $J_{5,2}$ = 1.3, H-5-furyl); 8.76 (dd, 1H, $J_{2,5}$ = 1.3, $J_{2,4}$ = 0.8, H-2-furyl); 8.81 (s, 1H, H-2). ^{13}C NMR (125.7 MHz, DMSO-d_6): 23.87 (d, $J_{C,P}$ = 4.5, (**C**$H_3)_2CH$); 24.00 (d, $J_{C,P}$ = 3.7, (**C**$H_3)_2CH$); 25.40, 27.31 ((**C**$H_3)_2C$); 65.34 (d, $J_{C,P}$ = 164.1, CH_2P); 70.37, 70.38 (2 × d, $J_{C,P}$ = 6.3, $(CH_3)_2$**C**H); 72.68 (d, $J_{C,P}$ = 11.3, CH_2-5′); 81.34 (CH-3′); 83.53 (CH-4′); 83.62 (CH-2′); 88.89 (CH-1′); 101.43 (CH-5); 109.53 (CH-4-furyl); 113.63 ($(CH_3)_2$**C**); 114.52 (C-4a); 125.08 (C-3-furyl); 128.03 (CH-6); 146.69 (CH-5-furyl); 145.20 (CH-2-furyl); 150.37 (C-4); 151.36 (C-7a); 151.39 (CH-2). ^{31}P{^{1}H} NMR

(202.4 MHz, DMSO-d_6): 20.60. HR-ESI-MS: *found*: 536.21554 ([M + H]$^+$, calcd for $C_{25}H_{35}O_8N_3P^+$: 536.2156); HR-ESI-MS: *found*: 558.1974 ([M + Na]$^+$, calcd for $C_{25}H_{34}O_8N_3NaP^+$: 558.1975).

Diisopropyl[(5-{2′,3′-*O*-isopropylidene-[4-(benzofuran-2-yl)-7*H*-pyrrolo[2,3-*d*]pyrimidin-7-yl]-*β*-D-ribofuranosyl}oxy)methyl]phosphonate (5d)

4-(Benzofuran-2-yl)-7-(5′-deoxy-4′,5′-didehydro-2′,3′-*O*-isopropylidene-*β*-D-ribofuranosyl)-7*H*-pyrrolo[2,3-*d*]pyrimidine (6d)

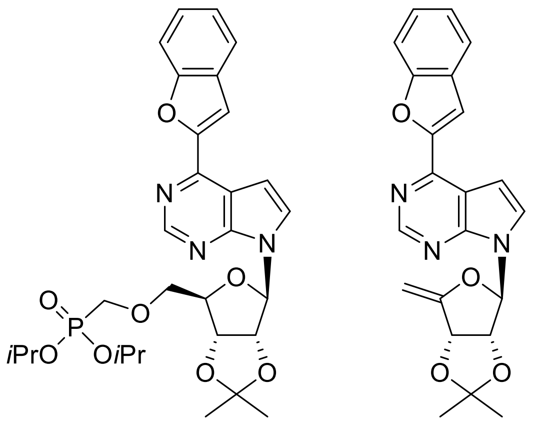

5d **6d**

Compound **5d** was prepared according to the general procedure B from **4d** (320 mg, 0.78 mmol) in dry DMF (9 mL). After 1 h, volatiles were removed in vacuo by co-distillation with toluene. HPFC (SiO_2, cyclohexane/EtOAc 0 → 80%) gave a side product of elimination **6d** (89 mg, 19%) as a white solid and the crude product **5d** as a white oil (124 mg). Data for **6d**: ^{1}H NMR (500.0 MHz, $CDCl_3$): 1.45, 1.61 (2 × q, 2 × 3H, ^4J = 0.5, $(CH_3)_2C$); 4.48 (dd, 1H, J_{gem} = 2.4, $J_{5'b,3'}$ = 1.0, H-5′b); 4.61 (dd, 1H, J_{gem} = 2.4, $J_{5'a,3'}$ = 1.2, H-5′a); 5.30 (dd, 1H, $J_{2',3'}$ = 6.1, $J_{2',1'}$ = 1.2, H-2′); 5.58 (ddd, 1H, $J_{3',2'}$ = 6.1, $J_{3',5'}$ = 1.2, 1.0, H-3′); 6.46 (d, 1H, $J_{1',2'}$ = 1.2, H-1′); 7.22 (d, 1H, $J_{5,6}$ = 3.7, H-5); 7.30 (d, 1H, $J_{6,5}$ = 3.7, H-6); 7.31 (ddd, 1H, $J_{5,4}$ = 7.8, $J_{5,6}$ = 7.2, $J_{5,7}$ = 1.1, H-5-benzofuryl); 7.42 (ddd, 1H, $J_{6,7}$ = 8.3, $J_{6,5}$ = 7.2, $J_{6,4}$ = 1.3, H-6-benzofuryl); 7.64 (ddt, 1H, $J_{7,6}$ = 8.3, $J_{7,5}$ = 1.1, $J_{7,3}$ = $J_{7,4}$ = 0.7, H-7-benzofuryl); 7.72 (ddd, 1H, $J_{4,5}$ = 7.8, $J_{4,6}$ = 1.3, $J_{4,7}$ = 0.7, H-4-benzofuryl); 7.85 (d, 1H, $J_{3,7}$ = 0.7, H-3-benzofuryl); 8.91 (s, 1H, H-2). ^{13}C NMR (125.7 MHz, $CDCl_3$): 25.70, 26.84 ((**C**$H_3)_2C$); 79.80 (CH-3′); 83.20 (CH-2′); 87.82 (CH_2-5′); 91.55 (CH-1′); 102.76 (CH-5); 109.22 (b, CH-3-benzofuryl); 111.80 (CH-7-benzofuryl); 114.07 ($(CH_3)_2$**C**); 114.81 (C-4a); 122.27 (CH-4-benzofuryl); 123.56 (CH-5-benzofuryl); 126.30 (CH-6-benzofuryl); 127.67 (CH-6); 128.09 (C-3a-benzofuryl); 147.66 (C-4); 151.65 (CH-2); 151.83 (C-7a); 154.24 (C-2-benzofuryl); 155.93 (C-7a-benzofuryl); 161.95 (C-4′). HR-ESI-MS: *found*: 390.1449 ([M +

H]$^+$, calcd for $C_{22}H_{20}O_4N_3^+$: 390.1448); HR-ESI-MS: *found*: 412.1268 ([M + Na]$^+$, calcd for $C_{22}H_{19}O_4N_3Na^+$: 412.1267). Data for **5d**: Compound **5d** was directly used for the next step. HR-ESI-MS: *found*: 586.2312 ([M + H]$^+$, calcd for $C_{29}H_{37}O_8N_3P^+$: 586.2312); HR-ESI-MS: *found*: 608.2130 ([M + Na]$^+$, calcd for $C_{29}H_{36}O_8N_3NaP^+$: 608.2132).

Diisopropyl[(5-{2′,3′-*O*-isopropylidene-[4-phenyl-7*H*-pyrrolo[2,3-*d*]pyrimidin-7-yl]-*β*-D-ribofuranosyl}oxy)methyl]phosphonate (5e)

4-Phenyl-7-(5′-deoxy-4′,5′-didehydro-2′,3′-*O*-isopropylidene-*β*-D-ribofuranosyl)-7*H*-pyrrolo[2,3-*d*]pyrimidine (6e)

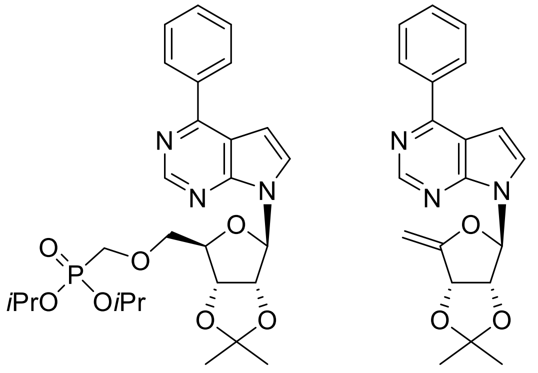

5e **6e**

Compound **5e** was prepared according to the general procedure B from **4e** (315 mg, 0.86 mmol) in dry DMF (9 mL). After 1 h, volatiles were removed in vacuo by co-distillation with toluene. HPFC (SiO_2, cyclohexane/EtOAc 0 → 80%) gave a side product of elimination **6e** (74 mg, 16%) as a white solid and the crude product **5e** as a white oil (76 mg). Data for **6e**: ^{1}H NMR (500.0 MHz, $CDCl_3$): 1.45, 1.61 (2 × q, 2 × 3H, ^4J = 0.5, $(CH_3)_2C$); 4.47 (dd, 1H, J_{gem} = 2.4, $J_{5'b,3'}$ = 1.0, H-5′b); 4.61 (dd, 1H, J_{gem} = 2.4, $J_{5'a,3'}$ = 1.2, H-5′a); 5.31 (dd, 1H, $J_{2',3'}$ = 6.1, $J_{2',1'}$ = 1.2, H-2′); 5.59 (dd 1H, $J_{3',2'}$ = 6.1, $J_{3',5'}$ = 1.2, 1.0, H-3′); 6.45 (d, 1H, $J_{1',2'}$ = 1.2, H-1′); 6.85 (d, 1H, $J_{5,6}$ = 3.7, H-5); 7.26 (d, 1H, $J_{6,5}$ = 3.7, H-6); 7.50–7.58 (m, 3H, H-*m,p*-Ph); 8.06–8.09 (m, 2H, H-*o*-Ph); 8.96 (s, 1H, H-2). ^{13}C NMR (125.7 MHz, $CDCl_3$): 25.69, 26.84 ((**C**$H_3)_2C$); 79.83 (CH-3′); 83.20 (CH-2′); 87.71 (CH_2-5′); 91.65 (CH-1′); 101.95 (CH-5); 114.04 ($(CH_3)_2$**C**); 116.50 (C-4a); 127.20 (CH-6); 128.83, 128.86 (CH-*o,m*-Ph); 130.26 (CH-*p*-Ph); 137.69 (C-*i*-Ph); 151.34 (C-7a); 151.74 (CH-2); 157.96 (C-4); 162.00 (C-4′). HR-ESI-MS: *found*: 350.1499 ([M + H]$^+$, calcd for $C_{20}H_{20}O_3N_3^+$: 350.1499); HR-ESI-MS: *found*: 372.1319 ([M + Na]$^+$, calcd for $C_{20}H_{19}O_3N_3Na^+$: 372.1318). Data for **5e**: Compound **5e** was used directly for the next step. HR-ESI-MS: *found*:

546.2362 ([M + H]$^+$, calcd for $C_{27}H_{37}O_7N_3P^+$: 546.2363); HR-ESI-MS: *found*: 568.2181 ([M + Na]$^+$, calcd for $C_{27}H_{36}O_7N_3NaP^+$: 568.2183).

Diisopropyl[(5-{2′,3′-*O*-isopropylidene-[4-(naphth-1-yl)-7*H*-pyrrolo[2,3-*d*]pyrimidin-7-yl]-*β*-D-ribofuranosyl}oxy)methyl]phosphonate (5f)

4-(Naphth-1-yl)-7-(5′-deoxy-4′,5′-didehydro-2′,3′-*O*-isopropylidene-*β*-D-ribofuranosyl)-7*H*-pyrrolo[2,3-*d*]pyrimidine (6f)

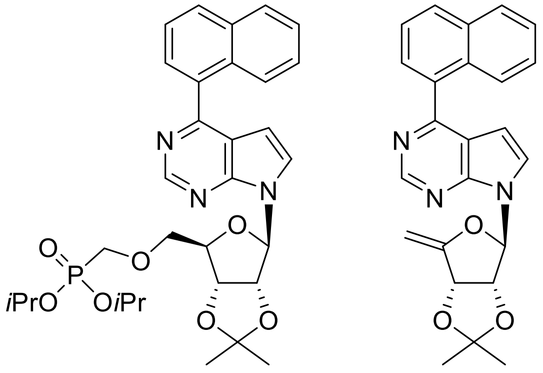

5f **6f**

Compound **5f** was prepared according to the general procedure B from **4f** (200 mg, 0.48 mmol) in dry DMF (10 mL). After 1.5 h, volatiles were removed in vacuo by co-distillation with toluene. HPFC (SiO_2, cyclohexane/EtOAc 5 → 50%) gave a side product of elimination **6f** (47 mg, 25%) as a white solid and the desired crude product **5f** as a white oil (95 mg, 85% purity), which was used directly for the next step without further purification. Data for **6f**: ^{1}H NMR (500.0 MHz, $CDCl_3$) : 1.46, 1.64 (2 × s, 2 × 3H, $(CH_3)_2C$); 4.49 (dd, 1H, J_{gem} = 2.4, $J_{5'b,3'}$ = 1.0, H-5′b); 4.64 (dd, 1H, J_{gem} = 2.4, $J_{5'a,3'}$ = 1.2, H-5′a); 5.36 (dd, 1H, $J_{2',3'}$ = 6.1, $J_{2',1'}$ = 1.2, H-2′); 5.62 (dt, 1H, $J_{3',2'}$ = 6.1, $J_{3',5'a}$ = $J_{3',5'b}$ = 1.1, H-3′); 6.43 (d, 1H, $J_{5,6}$ = 3.7, H-5); 6.49 (d, 1H, $J_{1',2'}$ = 1.2, H-1′); 7.20 (d, 1H, $J_{6,5}$ = 3.7, H-6); 7.47 (ddd, 1H, $J_{7,8}$ = 8.5, $J_{7,6}$ = 6.8, $J_{7,5}$ = 1.5, H-7-naphth); 7.53 (ddd, 1H, $J_{6,5}$ = 8.2, $J_{6,7}$ = 6.8, $J_{6,8}$ = 1.3, H-6-naphth); 7.61 (dd, 1H, $J_{3,4}$= 8.3, $J_{3,2}$ = 7.1, H-3-naphth); 7.74 (dd, 1H, $J_{2,3}$ = 7.1, $J_{2,4}$ = 1.3, H-2-naphth); 7.94 (dm, 1H, $J_{5,6}$ = 8.2, H-5-naphth); 8.00 (dt, 1H, $J_{4,3}$ = 8.2, $J_{4,2}$ = $J_{4,8}$ = 1.1, H-4-naphth); 8.07 (dq, 1H, $J_{8,7}$ = 8.4, $J_{8,6}$ = $J_{8,5}$ = $J_{8,4}$ = 1.0, H-8-naphth); 9.06 (s, 1H, H-2). ^{13}C NMR (125.7 MHz, $CDCl_3$): 25.69, 26.85 ((**C**$H_3)_2C$); 79.84 (CH-3′); 83.19 (CH-2′); 87.71 (CH_2-5′); 91.62 (CH-1′); 102.20 (CH-5); 114.06 ($(CH_3)_2$**C**); 119.04 (C-4a); 125.12 (CH-3-naphth); 125.60 (CH-8-naphth); 126.17 (CH-6-naphth); 126.88 (CH-7-naphth); 127.03 (CH-6); 128.14 (CH-2-naphth); 128.38 (CH-5-naphth); 130.03 (CH-4-naphth); 130.81 (C-8a-naphth); 133.96 (C-4a-naphth); 134.78 (C-1-naphth); 150.96 (C-7a); 151.67 (CH-2); 159.34

(C-4); 161.98 (C-4′). HR-ESI-MS: *found*: 400.16503 ([M + H]$^+$, calcd for $C_{24}H_{22}O_3N_3$: 400.16557). Data for **5f**: HR-ESI-MS: *found*: 596.25189 ([M + H]$^+$, calcd for $C_{31}H_{39}O_7N_3P$: 596.25201).

Diisopropyl[(5-{2′,3′-*O*-isopropylidene-[4-(naphthalen-2-yl)-7*H*-pyrrolo[2,3-*d*]pyrimidin-7-yl]-*β*-D-ribofuranosyl}oxy)methyl]phosphonate (5g)

4-(Naphthalen-2-yl)-7-(5′-deoxy-4′,5′-didehydro-2′,3′-*O*-isopropylidene-*β*-D-ribofuranosyl)-7*H*-pyrrolo[2,3-*d*]pyrimidine (6g)

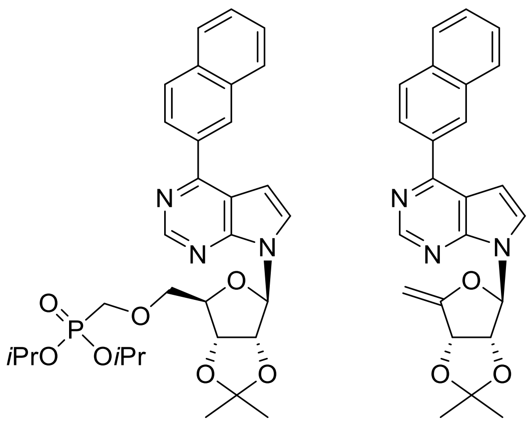

5g **6g**

Compound **5g** was prepared according to the general procedure B from crude **4g** (140 mg, 0.33 mmol) in dry DMF (10 mL). After 1.5 h, volatiles were removed in vacuo by co-distillation with toluene. HPFC (SiO_2, cyclohexane/EtOAc 0 $\rightarrow$ 80%) gave a side product of elimination **6g** (30 mg, 11% over 2 steps) as a white solid and the desired product **5g** as a white oil (83 mg, 80% purity), which was used directly for the deprotection step. Data for **6g**: ^{1}H NMR (500.0 MHz, $CDCl_3$) : 1.46, 1.62 (2 × s, 2 × 3H, $(CH_3)_2C$); 4.49 (dd, 1H, J_{gem} = 2.4, $J_{5'b,3'}$ = 0.9, H-5′b); 4.62 (dd, 1H, J_{gem} = 2.4, $J_{5'a,3'}$ = 1.2, H-5′a); 5.33 (dd, 1H, $J_{2',3'}$ = 6.1, $J_{2',1'}$ = 1.2, H-2′); 5.60 (dt, 1H, $J_{3',2'}$ = 6.1, $J_{3',5'a}$ = $J_{3',5'b}$ = 1.1, H-3′); 6.48 (d, 1H, $J_{1',2'}$ = 1.2, H-1′); 6.95 (d, 1H, $J_{5,6}$ = 3.7, H-5); 7.29 (d, 1H, $J_{6,5}$ = 3.7, H-6); 7.55 (ddd, 1H, $J_{7,8}$ = 8.3, $J_{7,6}$ = 6.8, $J_{7,5}$ = 1.8, H-7-naphth); 7.57 (ddd, 1H, $J_{6,5}$ = 8.4, $J_{6,7}$ = 6.8, $J_{6,8}$ = 1.8, H-6-naphth); 7.92 (m, 1H, H-5-naphth); 7.99 (m, 1H, H-8-naphth); 8.02 (d, 1H, $J_{4,3}$ = 8.7, H-4-naphth); 8.20 (dd, 1H, $J_{3,4}$ = 8.5, $J_{3,1}$ = 1.8, H-3-naphth); 8.59 (bd, 1H, $J_{1,3}$ = 1.8, H-1-naphth); 9.01 (s, 1H, H-2). ^{13}C NMR (125.7 MHz, $CDCl_3$): 25.69, 26.84 ((**C**H_3)$_2$C); 79.84 (CH-3′); 83.21 (CH-2′); 87.73 (CH_2-5′); 91.67 (CH-1′); 102.04 (CH-5); 114.05 ((CH_3)$_2$**C**); 116.74 (C-4a); 125.68 (CH-3-naphth); 126.56, 127.28 (CH-6,7-naphth); 127.32 (CH-6); 127.77 (CH-5-naphth); 128.63 (CH-4-naphth); 128.88 (CH-8-naphth); 129.08 (CH-1-naphth); 133.21 (C-8a-naphth); 134.16 (C-4a-naphth); 135.08 (C-4a-naphth); 151.41 (C-7a); 151.77 (CH-2);

157.86 (C-4); 162.01 (C-4′). HR-ESI-MS: *found*: 400.16537 ([M + H]$^+$, calcd for $C_{24}H_{22}O_3N_3^+$: 400.16557). Data for **5g**: HR-ESI-MS: *found*: 596.25184 ([M + H]$^+$, calcd for $C_{31}H_{39}O_7N_3P^+$: 596.25201).

Diisopropyl[(5-{2′,3′-*O*-isopropylidene-[4-(biphenyl-4-yl)-7*H*-pyrrolo[2,3-*d*]pyrimidin-7-yl]-*β*-D-ribofuranosyl}oxy)methyl]phosphonate (5h)

4-(Biphenyl-4-yl)-7-(5′-deoxy-4′,5′-didehydro-2′,3′-*O*-isopropylidene-*β*-D-ribofuranosyl)-7*H*-pyrrolo[2,3-*d*]pyrimidine (6h)

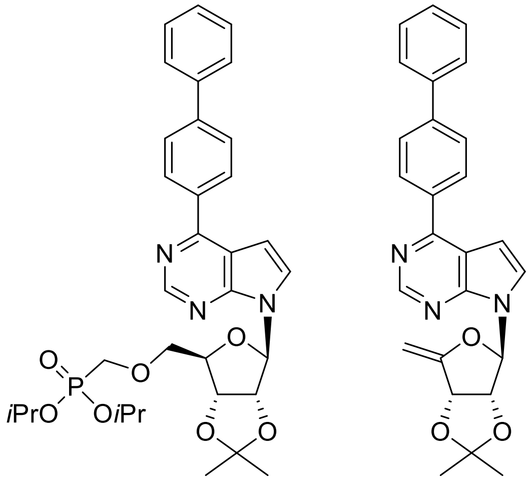

5h **6h**

Compound **5h** was prepared according to the general procedure B from **4h** (250 mg, 0.56 mmol) in dry DMF (10 mL). After 1 h, volatiles were removed in vacuo by co-distillation with toluene. HPFC (SiO_2, cyclohexane/EtOAc 5 → 50%) gave a side product of elimination **6h** (73 mg, 30%) as a white solid and the desired product **5h** as a white oil (166 mg, 47%). Data for **6h**: ^{1}H NMR (500.0 MHz, DMSO-d_6) : 1.38, 1.51 (2 × s, 2 × 3H, $(CH_3)_2C$); 4.37 (dd, 1H, J_{gem} = 2.2, $J_{5'b,3'}$ = 0.9, H-5′b); 4.45 (dd, 1H, J_{gem} = 2.2, $J_{5'a,3'}$ = 1.1, H-5′a); 5.39 (dd, 1H, $J_{2',3'}$ = 6.0, $J_{2',1'}$ = 1.3, H-2′); 5.66 (dt, 1H, $J_{3',2'}$ = 6.1, $J_{3',5'a}$ = $J_{3',5'b}$ = 1.0, H-3′); 6.65 (d, 1H, $J_{1',2'}$ = 1.4, H-1′); 7.10 (d, 1H, $J_{5,6}$ = 3.8, H-5); 7.43 (m, 1H, H-*p*-Ph); 7.50–7.55 (m, 2H, H-*m*-Ph); 7.76–7.80 (m, 2H, H-*o*-Ph); 7.82 (d, 1H, $J_{5,6}$ = 3.8, H-6); 7.88–7.92 (m, 2H, H-*m*-phenylene); 8.26–8.30 (m, 2H, H-*o*-phenylene); 8.93 (s, 1H, H-2). ^{13}C NMR (125.7 MHz, DMSO-d_6): 25.65, 26.88 (($\mathbf{C}H_3)_2C$); 79.64 (CH-3′); 82.41 (CH-2′); 87.32 (CH_2-5′); 90.20 (CH-1′); 101.77 (CH-5); 113.27 ($(CH_3)_2\mathbf{C}$); 115.74 (C-4a); 127.08 (CH-*o*-Ph); 127.45 (CH-*m*-phenylene); 128.33 (CH-*p*-Ph); 129.31 (CH-6); 129.39 (CH-*o*-phenylene); 129.55 (CH-*m*-Ph); 136.48 (C-*i*-phenylene); 139.53 (C-*i*-Ph); 142.27 (C-*p*-phenylene); 151.51 (C-7a); 151.56 (CH-2); 156.27 (C-4); 161.96 (C-4′). HR-ESI-MS: *found*:

426.1807 ([M + H]$^+$, calcd for $C_{26}H_{24}O_3N_3^+$: 426.1812). Data for **5h**: ^{1}H NMR (500.0 MHz, DMSO-*d*$_6$): 1.20, 1.21, 1.22, 1.23 (4 × d, 4 × 3H, J_{vic} = 6.2, (C**H**$_3$)$_2$CH); 1.34, 1.58 (2 × s, 2 × 3H, $(CH_3)_2C$); 3.70 (dd, 1H, J_{gem} = 10.6, $J_{5'b,4'}$ = 5.3, H-5′b); 3.78 (dd, 1H, J_{gem} = 10.6, $J_{5'a,4'}$ = 4.3, H-5′a); 3.79, 3.82 (2 × dd, 2 × 1H, J_{gem} = 13.9, $J_{H,P}$ = 8.1, CH_2P); 4.33 (ddd, 1H, $J_{4',5'}$ = 5.3, 4.3, $J_{4',3'}$ = 3.1, H-4′); 4.53 – 4.64 (m, 2H, $(CH_3)_2$C**H**); 4.98 (dd, 1H, $J_{3',2'}$ = 6.2, $J_{3',4'}$ = 3.1, H-3′); 5.27 (dd, 1H, $J_{2',3'}$ = 6.2, $J_{2',1'}$ = 3.3, H-2′); 6.46 (d, 1H, $J_{1',2'}$ = 3.3, H-1′); 7.08 (d, 1H, $J_{5,6}$ = 3.8, H-5); 7.43 (m, 1H, H-*p*-Ph); 7.50–7.55 (m, 2H, H-*m*-Ph); 7.77–7.81 (m, 2H, H-*o*-Ph); 7.89–7.93 (m, 2H, H-*m*-phenylene); 7.96 (d, 1H, $J_{6,5}$ = 3.8, H-6); 8.27–8.31 (m, 2H, H-*o*-phenylene); 8.94 (s, 1H, H-2). ^{13}C NMR (125.7 MHz, DMSO-*d*$_6$): 23.88 (d, $J_{C,P}$ = 4.5, (**C**H$_3$)$_2$CH); 24.01 (d, $J_{C,P}$ = 3.7, (**C**H$_3$)$_2$CH); 25.42, 27.33 ((**C**H$_3$)$_2$C); 65.36 (d, $J_{C,P}$ = 164.1, CH_2P); 70.40 (d, $J_{C,P}$ = 6.3, $(CH_3)_2$**C**H); 72.72 (d, $J_{C,P}$ = 11.3, CH$_2$-5′); 81.35 (CH-3′); 83.63 (CH-4′); 83.72 (CH-2′); 89.04 (CH-1′); 101.61 (CH-5); 113.66 ((CH$_3$)$_2$**C**); 115.49 (C-4a); 127.01 (CH-*o*-Ph); 127.37 (CH-*m*-phenylene); 128.23 (CH-*p*-Ph); 128.61 (CH-6); 129.31 (CH-*m*-Ph); 129.45 (CH-*o*-phenylene); 136.56 (C-*i*-phenylene); 139.49 (C-*i*-Ph); 142.11 (C-*p*-phenylene); 151.47 (CH-2); 151.80 (C-7a); 155.97 (C-4). ^{31}P{^{1}H} NMR (202.4 MHz, DMSO-*d*$_6$): 20.45. HR-ESI-MS: *found*: 622.26721 ([M + H]$^+$, calcd for $C_{33}H_{41}O_7N_3P^+$: 622.26766). HR-ESI-MS: *found*: 644.2489 ([M + Na]$^+$, calcd for $C_{33}H_{40}O_7N_3NaP^+$: 644.2496).

Diisopropyl[(5-{2′,3′-*O*-isopropylidene-[4-(phenanthren-9-yl)-7*H*-pyrrolo[2,3-*d*]pyrimidin-7-yl]-*β*-D-ribofuranosyl}oxy)methyl]phosphonate (5i)

4-(Phenanthren-9-yl)-7-(5′-deoxy-4′,5′-didehydro-2′,3′-*O*-isopropylidene-*β*-D-ribofuranosyl)-7*H*-pyrrolo[2,3-*d*]pyrimidine (6i)

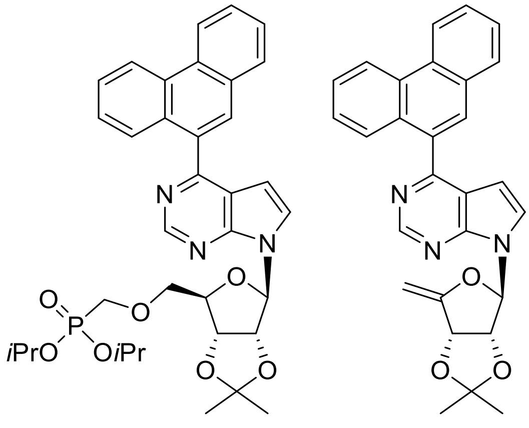

5i **6i**

Compound **5i** was prepared according to the general procedure B from **4i** (450 mg, 0.96 mmol) in dry DMF (10 mL). After 1.5 h, volatiles were removed in vacuo by co-distillation with

toluene. HPFC (SiO_2, cyclohexane/EtOAc 5 $\rightarrow$ 50%) gave a side product of elimination **6i** (43 mg, 10%) as a white solid and the desired product **5i** as a yellowish oil (288 mg, 46%). Data for **6i**: ^{1}H NMR (500.0 MHz, $CDCl_3$) : 1.47, 1.64 (2 × s, 2 × 3H, $(CH_3)_2C$); 4.50 (dd, 1H, J_{gem} = 2.4, $J_{5'b,3'}$ = 1.0, H-5′b); 4.65 (dd, 1H, J_{gem} = 2.4, $J_{5'a,3'}$ = 1.2, H-5′a); 5.37 (dd, 1H, $J_{2',3'}$ = 6.1, $J_{2',1'}$ = 1.2, H-2′); 5.63 (dt, 1H, $J_{3',2'}$ = 6.1, $J_{3',5'a}$ = $J_{3',5'b}$ = 1.1, H-3′); 6.44 (d, 1H, $J_{5,6}$ = 3.7, H-5); 6.50 (d, 1H, $J_{1',2'}$ = 1.2, H-1′); 7.20 (d, 1H, $J_{6,5}$ = 3.7, H-6); 7.55 (ddd, 1H, $J_{7,8}$ = 8.3, $J_{7,6}$ = 7.0, $J_{7,5}$ = 1.2, H-7-phenanthryl); 7.64 (ddd, 1H, $J_{2,1}$ = 7.9, $J_{2,3}$ = 7.0, $J_{2,4}$ = 1.2, H-2-phenanthryl); 7.70 (ddd, 1H, $J_{6,5}$= 8.4, $J_{6,7}$ = 7.0, $J_{6,8}$ = 1.4, H-6-phenanthryl); 7.73 (ddd, 1H, $J_{3,4}$ = 8.4, $J_{3,2}$ = 7.0, $J_{3,1}$ = 1.4, H-3-phenanthryl); 7.94 (dd, 1H, $J_{1,2}$ = 7.9, $J_{1,3}$ = 1.5, H-1-phenanthryl); 8.00 (s, 1H, H-10-phenanthryl); 8.07 (dd, 1H, $J_{8,7}$ = 8.3, $J_{8,6}$ = 1.5, H-8-phenanthryl); 8.76 (bd, 1H, $J_{4,3}$ = 8.5, H-4-phenanthryl); 8.80 (dm, 1H, $J_{5,6}$ = 8.4, H-5-phenanthryl); 9.09 (s, 1H, H-2). ^{13}C NMR (125.7 MHz, $CDCl_3$): 25.69, 26.85 ((**C**$H_3)_2C$); 79.84 (CH-3′); 83.19 (CH-2′); 87.76 (CH_2-5′); 91.61 (CH-1′); 102.19 (CH-5); 114.07 ($(CH_3)_2$**C**); 119.19 (C-4a); 122.63 (CH-4-phenanthryl); 122.95 (CH-5-phenanthryl); 126.50 (CH-8-phenanthryl); 126.80 (CH-7-phenanthryl); 126.91 (CH-2-phenanthryl); 126.96 (CH-6-phenanthryl); 127.07 (CH-6); 127.63 (CH-3-phenanthryl); 129.21 (CH-1-phenanthryl); 129.45 (CH-10-phenanthryl); 129.54 (C-8a-phenanthryl); 130.82, 130.85 (C-4a,4b-phenanthryl); 130.97 (C-10a-phenanthryl); 133.58 (C-9-phenanthryl); 150.98 (C-7a); 151.73 (CH-2); 159.38 (C-4); 161.96 (C-4′). HR-ESI-MS: *found*: 450.18067 ([M + H]$^+$, calcd for $C_{28}H_{24}O_3N_3^+$: 450.18122). Data for **5i**: ^{1}H NMR (500.0 MHz, DMSO-d_6) : 1.197, 1.20, 1.21, 1.22 (4 × d, 4 × 3H, J_{vic} = 6.0, (C**H$_3$**)$_2$CH); 1.35, 1.60 (2 × s, 2 × 3H, $(CH_3)_2C$); 3.72 (dd, 1H, J_{gem} = 10.7, $J_{5'b,4'}$ = 5.3, H-5′b); 3.80 (dd, 1H, J_{gem} = 10.7, $J_{5'a,4'}$ = 4.4, H-5′a); 3.80, 3.81 (2 × dd, 2 × 1H, J_{gem} = 14.0, $J_{H,P}$ = 8.1, CH_2P); 4.36 (btd, 1H, $J_{4',5'}$ = 4.8, $J_{4',3'}$ = 3.0, H-4′); 4.51–4.63 (m, 2H, $(CH_3)_2C$**H**); 5.00 (dd, 1H, $J_{3',2'}$ = 6.2, $J_{3',4'}$ = 3.0, H-3′); 5.30 (dd, 1H, $J_{2',3'}$ = 6.2, $J_{2',1'}$ = 3.2, H-2′); 6.50 (d, 1H, $J_{5,6}$ = 3.8, H-5); 6.51 (d, 1H, $J_{1',2'}$ = 3.2, H-1′); 7.60 (ddd, 1H, $J_{7,8}$ = 8.3, $J_{7,6}$ = 7.0, $J_{7,5}$ = 1.2, H-7-phenanthryl); 7.73 (ddd, 1H, $J_{2,1}$ = 8.0, $J_{2,3}$ = 7.0, $J_{2,4}$ = 1.2, H-2-phenanthryl); 7.77 (ddd, 1H, $J_{6,5}$= 8.4, $J_{6,7}$ = 6.9, $J_{6,8}$ = 1.4, H-6-phenanthryl); 7.81 (ddd, 1H, $J_{3,4}$ = 8.4, $J_{3,2}$ = 6.9, $J_{3,1}$ = 1.4, H-3-phenanthryl); 7.90 (d, 1H, $J_{6,5}$ = 3.8, H-6); 8.02 (dd, 1H, $J_{8,7}$ = 8.3, $J_{8,6}$ = 1.4, H-8-phenanthryl); 8.11 (s, 1H, H-10-phenanthryl); 8.12 (dd, 1H, $J_{1,2}$ = 8.0, $J_{1,3}$ = 1.5, H-1-phenanthryl); 8.94 (dm, 1H, $J_{4,3}$ = 8.4, H-4-phenanthryl); 8.99 (dm, 1H, $J_{5,6}$ = 8.4, H-5-phenanthryl); 9.06 (s, 1H, H-2). ^{13}C NMR (125.7 MHz, DMSO-d_6): 23.88 (d, $J_{C,P}$ = 4.4, (**C**$H_3)_2CH$); 24.00 (d, $J_{C,P}$ = 3.7, (**C**$H_3)_2CH$); 25.43, 27.36 ((**C**$H_3)_2C$); 65.36 (d, $J_{C,P}$ = 164.2, CH_2P); 70.38 (d, $J_{C,P}$ = 6.1, $(CH_3)_2C$**H**); 72.74 (d, $J_{C,P}$ = 11.3, CH_2-5′); 81.36 (CH-3′); 83.63, 83.74 (CH-2′,4′); 89.08 (CH-1′); 101.50 (CH-5); 113.68 ($(CH_3)_2$**C**);

118.46 (C-4a); 123.14 (CH-4-phenanthryl); 123.37 (CH-5-phenanthryl); 126.55 (CH-8-phenanthryl); 127.10 (CH-7-phenanthryl); 127.37 (CH-6-phenanthryl); 127.51 (CH-2-phenanthryl); 128.20 (CH-3-phenanthryl); 128.41 (CH-6); 129.28 (CH-1-phenanthryl); 129.31 (C-8a-phenanthryl); 129.50 (CH-10-phenanthryl); 130.37, 130.47 (C-4a,4b-phenanthryl); 130.75 (C-10a-phenanthryl); 133.44 (C-9-phenanthryl); 151.34 (CH-2); 151.35 (C-7a); 158.44 (C-4). $^{31}P\{^1H\}$ NMR (202.4 MHz, DMSO-d_6): 20.47. HR-ESI-MS: *found*: 646.2674 ($[M + H]^+$, calcd for $C_{33}H_{41}O_7N_3P^+$: 646.2676); HR-ESI-MS: *found*: 668.2489 ($[M + Na]^+$, calcd for $C_{35}H_{40}O_7N_3NaP^+$: 668.2496).

Diisopropyl[(5-{2′,3′-*O*-isopropylidene-[4-(dibenzofuran-4-yl)-7*H*-pyrrolo[2,3-*d*]pyrimidin-7-yl]-*β*-D-ribofuranosyl}oxy)methyl]phosphonate (5j)

4-(Dibenzofuran-4-yl)-7-(5′-deoxy-4′,5′-didehydro-2′,3′-*O*-isopropylidene-*β*-D-ribofuranosyl)-7*H*-pyrrolo[2,3-*d*]pyrimidine (6j)

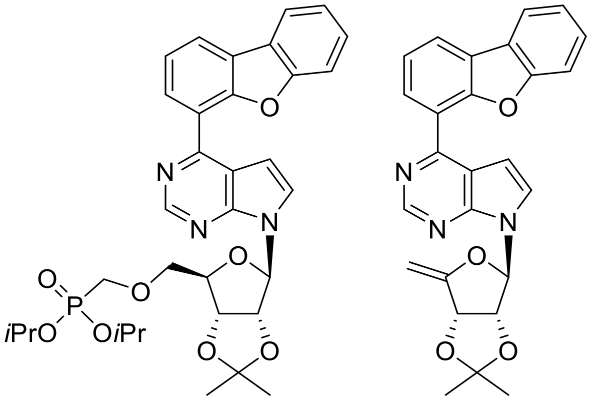

5j **6j**

Compound **5j** was prepared according to the general procedure B from **4j** (340 mg, 0.74 mmol) in dry DMF (10 mL). After 1.5 h, volatiles were removed in vacuo by co-distillation with toluene. HPFC (SiO_2, cyclohexane/EtOAc 5 → 50%) gave a side product of elimination **6j** (66 mg, 20%) as a white solid and the desired product **5j** as a white oil (173 mg, 37%). Data for **6j**: ^1H NMR (500.0 MHz, $CDCl_3$): 1.47, 1.63 (2 × q, 2 × 3H, ^4J = 0.6, $(CH_3)_2C$); 4.49 (dd, 1H, J_{gem} = 2.4, $J_{5'b,3'}$ = 0.9, H-5′b); 4.63 (dd, 1H, J_{gem} = 2.4, $J_{5'a,3'}$ = 1.2, H-5′a); 5.36 (dd, 1H, $J_{2',3'}$ = 6.1, $J_{2',1'}$ = 1.2, H-2′); 5.62 (ddd, 1H, $J_{3',2'}$ = 6.1, $J_{3',5'}$ = 1.2, 0.9, H-3′); 6.50 (d, 1H, $J_{1',2'}$ = 1.2, H-1′); 6.77 (d, 1H, $J_{5,6}$ = 3.7, H-5); 7.28 (d, 1H, $J_{6,5}$ = 3.7, H-6); 7.40 (ddd, 1H, $J_{8,9}$ = 7.7, $J_{8,7}$ = 7.2, $J_{8,6}$ = 1.0, H-8-dibenzofuryl); 7.49 (ddd, 1H, $J_{7,6}$ = 8.2, $J_{7,8}$ = 7.2, $J_{7,9}$ = 1.3, H-7-dibenzofuryl); 7.55 (t, 1H, $J_{2,1}$ = $J_{2,3}$ = 7.6, H-2-dibenzofuryl); 7.58 (dt, 1H, $J_{6,7}$ = 8.2, $J_{6,8}$ = $J_{6,9}$ = 1.0, H-6-dibenzofuryl); 8.01 (dd, 1H, $J_{3,2}$ = 7.6, $J_{3,1}$ = 1.3, H-3-dibenzofuryl); 8.03 (ddd, 1H, $J_{9,8}$ = 7.7, $J_{9,7}$ = 1.3, $J_{9,6}$ = 1.0, H-9-

dibenzofuryl); 8.13 (dd, 1H, $J_{1,2}$ = 7.6, $J_{1,3}$ = 1.3, H-1-dibenzofuryl); 9.08 (s, 1H, H-2). ^{13}C NMR (125.7 MHz, $CDCl_3$): 25.72, 26.87 ((**C**H_3)$_2$C); 79.87 (CH-3′); 83.26 (CH-2′); 87.77 (CH_2-5′); 91.69 (CH-1′); 103.08 (CH-5); 111.93 (CH-6-dibenzofuryl); 114.08 ((CH_3)$_2$**C**); 118.19 (C-4a); 120.77 (CH-9-dibenzofuryl); 122.30 (b, C-4-dibenzofuryl); 122.54 (b, CH-1-dibenzofuryl); 123.15 (CH-8-dibenzofuryl); 123.29 (CH-2-dibenzofuryl); 123.82 (C-9a-dibenzofuryl); 125.45 (C-9b-dibenzofuryl); 127.04 (b, CH-6); 127.57 (CH-7-dibenzofuryl); 128.58 (CH-3-dibenzofuryl); 151.20 (C-7a); 151.66 (b, CH-2); 153.42 (C-4a-dibenzofuryl); 154.70 (C-4); 156.14 (C-5a-dibenzofuryl); 162.05 (C-4′). HR-ESI-MS: *found*: 440.1596 ([M + H]$^+$, calcd for $C_{26}H_{22}O_4N_3^+$: 440.1604). Data for **5j**: ^{1}H NMR (500.0 MHz, DMSO-d_6): 1.199, 1.207, 1.210, 1.219 (4 × d, 4 × 3H, J_{vic} = 6.2, (C**H₃**)$_2$CH); 1.35, 1.60 (2 × s, 2 × 3H, (CH_3)$_2$C); 3.72 (dd, 1H, J_{gem} = 10.6, $J_{5',4'}$ = 5.2, H-5′b); 3.79 - 3.86 (m, 3H, H-5′a, CH_2P); 4.35 (ddd, 1H, $J_{4',5'}$ = 5.2, 4.3, $J_{4',3'}$ = 3.0, H-4′); 4.53 – 4.64 (m, 2H, (CH_3)$_2$C**H**); 5.00 (dd, 1H, $J_{3',2'}$ = 6.2, $J_{3',4'}$ = 3.0, H-3′); 5.30 (dd, 1H, $J_{2',3'}$ = 6.2, $J_{2',1'}$ = 3.3, H-2′); 6.49 (d, 1H, $J_{1',2'}$ = 3.3, H-1′); 6.75 (d, 1H, $J_{5,6}$ = 3.8, H-5); 7.47 (ddd, 1H, $J_{8,9}$ = 7.7, $J_{8,7}$ =7.3, $J_{8,6}$ = 0.9, H-8-dibenzofuryl); 7.56 (ddd, 1H, $J_{7,6}$ = 8.2, $J_{7,8}$ = 7.3, $J_{7,9}$ = 1.4, H-7-dibenzofuryl); 7.63 (t, 1H, $J_{2,1}$ = $J_{2,3}$ = 7.6, H-2-dibenzofuryl); 7.69 (dt, 1H, $J_{6,7}$ = 8.2, $J_{6,8}$ = $J_{6,9}$ = 0.9, H-6-dibenzofuryl); 7.94 (d, 1H, $J_{6,5}$ = 3.8, H-6); 7.99 (dd, 1H, $J_{3,2}$ = 7.6, $J_{3,1}$ = 1.3, H-3-dibenzofuryl); 8.26 (dd, 1H, $J_{9,8}$ = 7.7, $J_{9,7}$ = 1.4, $J_{9,6}$ = 0.9, H-9-dibenzofuryl); 8.38 (dd, 1H, $J_{1,2}$ = 7.6, $J_{1,3}$ = 1.3, H-1-dibenzofuryl); 9.04 (s, 1H, H-2). ^{13}C NMR (125.7 MHz, DMSO-d_6): 23.87 (d, $J_{C,P}$ = 4.5, (CH_3)$_2$CH); 23.99 (d, $J_{C,P}$ = 3.7, (**C**H_3)$_2$CH); 25.42, 27.35 ((**C**H_3)$_2$C); 65.36 (d, $J_{C,P}$ = 164.1, CH_2P); 70.36, 70.37 (2 × d, $J_{C,P}$ = 6.3, (CH_3)$_2$**C**H); 72.74 (d, $J_{C,P}$ = 11.4, CH_2-5′); 81.34 (CH-3′); 83.61 (CH-4′); 83.75 (CH-2′); 89.06 (CH-1′); 102.20 (CH-5); 111.94 (CH-6-dibenzofuryl); 113.66 ((CH_3)$_2$**C**); 117.42 (C-4a); 121.59 (CH-9-dibenzofuryl); 122.47 (C-4-dibenzofuryl); 123.11 (CH-1-dibenzofuryl); 123.43 (C-9a-dibenzofuryl); 123.63, 123.67 (CH-2,8-dibenzofuryl); 124.97 (C-9b-dibenzofuryl); 128.21, 128.24 (CH-6, CH-7-dibenzofuryl); 128.70 (CH-3-dibenzofuryl); 151.37 (C-7a); 151.59 (CH-2); 152.92 (C-4a-dibenzofuryl); 153.97 (C-4); 155.68 (C-5a-dibenzofuryl). ^{31}P{^{1}H} NMR (202.4 MHz, DMSO-d_6): 20.47.

HR-ESI-MS: *found*: 636.2461 ([M + H]$^+$, calcd for $C_{33}H_{39}O_8N_3P^+$: 636.2469); HR-ESI-MS: *found*: 658.2277 ([M + Na]$^+$, calcd for $C_{33}H_{38}O_8N_3NaP^+$: 658.2288).

[(5-{[4-(Thiophen-3-yl)-7*H*-pyrrolo[2,3-*d*]pyrimidin-7-yl]-*β*-D-ribofuranosyl}oxy)methyl]phosphonic acid (7a)

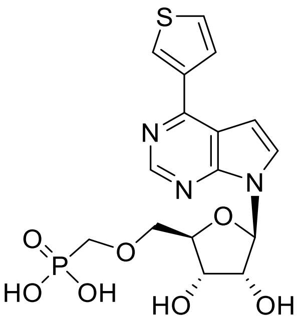

Compound **7a** was prepared according to the general procedure C from corresponding starting nucleoside **5a** (70 mg, 0.13 mmol) in dry MeCN (5 mL). RP-HPFC (C-18, H_2O/MeOH 0 →100 %) and lyophilization from mixture $^tBuOH/H_2O$ gave product **5a** as a white lyophilizate (10 mg, 20 %). ^1H NMR (500.0 MHz, DMSO-d_6): 3.61, 3.64 (2 × dd, 2 × 1H, J_{gem} = 13.4, $J_{H,P}$ = 8.9, CH_2P); 3.69 (dd, 1H, J_{gem} = 10.8, $J_{5'b,4'}$ = 4.3, H-5′b); 3.73 (dd, 1H, J_{gem} = 10.8, $J_{5'a,4'}$ = 3.4, H-5′a); 4.04 (dt, 1H, $J_{4',5'}$ = 4.3, 3.4, $J_{4',3'}$ = 3.4, H-4′); 4.15 (dd, 1H, $J_{3',2'}$ = 5.1, $J_{3',4'}$ = 3.4, H-3′); 4.49 (dd, 1H, $J_{2',1'}$ = 6.0, $J_{2',3'}$ = 5.1, H-2′); 6.30 (d, 1H, $J_{1',2'}$ = 6.0, H-1′); 7.09 (d, 1H, $J_{5,6}$ = 3.9, H-5); 7.75 (dd, 1H, $J_{5,4}$ = 5.1, $J_{5,2}$ = 2.9, H-5-thienyl); 7.94 (dd, 1H, $J_{4,5}$ = 5.1, $J_{4,2}$ = 1.3, H-4-thienyl); 8.00 (d, 1H, $J_{6,5}$ = 3.9, H-6); 8.53 (dd, 1H, $J_{2,5}$ = 2.9, $J_{2,4}$ = 1.3, H-2-thienyl); 8.81 (s, 1H, H-2). ^{13}C NMR (125.7 MHz, DMSO-d_6): 67.44 (d, $J_{C,P}$ = 159.4, CH_2P); 71.10 (CH-3′); 73.07 (d, $J_{C,P}$ = 11.8, CH_2-5′); 74.02 (CH-2′); 83.39 (CH-4′); 86.53 (CH-1′); 101.33 (CH-5); 114.56 (C-4a); 127.33 (CH-5-thienyl); 127.58 (CH-4-thienyl); 128.14 (CH-6); 128.65 (CH-2-thienyl); 140.09 (C-3-thienyl); 151.15 (CH-2); 151.51 (C-4); 152.43 (C-7a). $^{31}P\{^1H\}$ NMR (202.4 MHz, DMSO-d_6): 17.59. HR-ESI-MS: *found*: 426.0526 ([M – H]$^-$, calcd for $C_{16}H_{17}O_7N_3PS^+$: 426.0530).

[(5-{[4-(Furan-2-yl)-7*H*-pyrrolo[2,3-*d*]pyrimidin-7-yl]-*β*-D-ribofuranosyl}oxy)methyl]-phosphonate triethylammonium salt (7b)

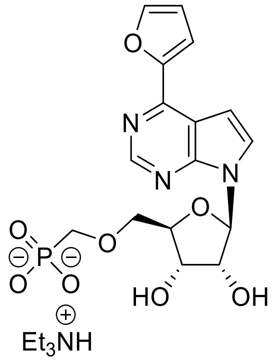

Compound **7b** was prepared according to the general procedure C from corresponding starting material **5b** (75 mg, 0.14 mmol) in dry MeCN (5 mL). RP-HPFC (C-18, H_2O/MeOH 0

→100 %), re-purification by HPLC (C-18, H_2O/MeOH 0 →100 %) and lyophilization from a mixture $^tBuOH/H_2O$ gave product **7b** (1 : 0.33 of triethylammonium salt*) as a yellow powder (33 mg, 46%). ^1H NMR (500.0 MHz, DMSO-d_6): 3.53 (dd, 1H, J_{gem} = 12.9, $J_{H,P}$ = 9.1, $CH_a\mathbf{H_b}P$); 3.57 (dd, 1H, J_{gem} = 12.9, $J_{H,P}$ = 8.9, $C\mathbf{H_a}H_bP$); 3.66, 3.69 (2 × dd, 2 × 1H, J_{gem} = 10.7, $J_{5',4'}$ = 3.5, H-5′); 4.03 (td, 1H, $J_{4',5'}$ = 3.5, $J_{4',3'}$ = 2.8, H-4′); 4.17 (dd, 1H, $J_{3',2'}$ = 5.0, $J_{3',4'}$ = 2.8, H-3′); 4.56 (dd, 1H, $J_{2',1'}$ = 6.5, $J_{2',3'}$ = 5.0, H-2′); 6.29 (d, 1H, $J_{1',2'}$ = 6.5, H-1′); 6.77 (dd, 1H, $J_{4,3}$ = 3.5, $J_{4,5}$ = 1.7, H-4-furyl); 7.02 (d, 1H, $J_{5,6}$ = 3.8, H-5); 7.44 (dd, 1H, $J_{3,4}$ = 3.5, $J_{3,5}$ = 0.9, H-3-furyl); 8.04 (dd, 1H, $J_{5,4}$ = 1.7, $J_{5,3}$ = 0.9, H-5-furyl); 8.07 (d, 1H, $J_{6,5}$ = 3.8, H-6); 8.75 (s, 1H, H-2). ^{13}C NMR (125.7 MHz, DMSO-d_6): 68.19 (d, $J_{C,P}$ = 160.3, CH_2P); 71.29 (CH-3′); 72.91 (d, $J_{C,P}$ = 11.9, CH_2-5′); 74.07 (CH-2′); 83.66 (CH-4′); 86.21 (CH-1′); 101.51 (CH-5); 112.71 (C-4a); 112.77 (CH-4-furyl); 113.22 (CH-3-furyl); 128.55 (CH-6); 146.35 (C-4); 146.39 (CH-5-furyl); 151.11 (CH-2); 152.55 (C-7a); 152.74 (C-2-furyl). $^{31}P\{^1H\}$ NMR (202.4 MHz, DMSO-d_6): 16.49. HR-ESI-MS: *found*: 410.0756 ($[M-H]^-$, calcd for $C_{16}H_{17}O_8N_3P^+$: 410.0758).

* HPLC column (Phenomenex, Luna Omega Polar C18 column of 150 x 21.2 mm) is mainly used for purifications of mono- or triphosphates in 0.1 M TEAB buffer, the triethylammonium residue stays on the column, therefore compound **7b** was obtained as a triethylammonium salt.

[(5-{[4-(Furan-3-yl)-7*H*-pyrrolo[2,3-*d*]pyrimidin-7-yl]-*β*-D-ribofuranosyl}oxy)methyl]phosphonic acid (7c)

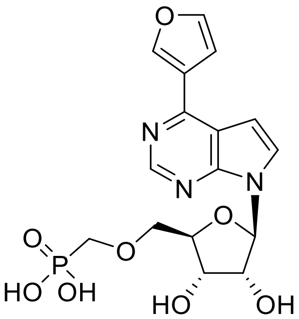

Compound **7c** was prepared according to the general procedure C from corresponding starting material **5c** (70 mg, 0.13 mmol) in dry MeCN (5 mL). RP-HPFC (C-18, H_2O/MeOH 0 →100 %) and lyophilization from mixture $^tBuOH/H_2O$ gave product **7c** as a white powder (28 mg, 53%). ^1H NMR (500.2 MHz, DMSO-d_6): 3.49–3.62 (m, 2H, OCH_2P); 3.66 (dd, 1H, J_{gem} = 10.8, $J_{5'b,4'}$ = 4.1, H-5′b); 3.70 (dd, 1H, J_{gem} = 10.7, $J_{5'a,4'}$ = 3.5, H-5′a); 4.03 (td, 1H, $J_{4'5'}$ = 3.7, $J_{4',3'}$ = 3.3, H-4′); 4.18 (dd, 1H, $J_{3',2'}$ = 5.0, $J_{3',4'}$ = 3.2, H-3′); 4.54 (bt, 1H, $J_{2',3'}$ = $J_{2',1'}$ = 5.7, H-2′); 6.28 (d, 1H, $J_{1',2'}$ = 6.3, H-1′); 7.04 (d, 1H, $J_{5,6}$ = 3.9, H-5); 7.23 (dd, 1H, $J_{4,5}$ = 1.8, $J_{4,2}$ = 0.7, H-4-furyl); 7.87 (t, 1H,

$J_{5,4}$ = $J_{5,2}$ = 1.7, H-5-furyl); 7.98 (d, 1H, $J_{6,5}$ = 3.9, H-6); 8.67 (bt, 1H, $J_{2,5}$ = $J_{2,4}$ = 1.1, H-2-furyl); 8.77 (s, 1H, H-2). ^{13}C NMR (125.8 MHz, DMSO-d_6): 68.36 (bd, $J_{C,P'}$ = 158.3, CH_2P); 71.15 (CH-3′); 72.91 (d, $J_{C,P'}$ = 11.1, CH_2-5′); 73.96 (CH-2′); 83.53 (CH-4′); 86.43 (CH-1′); 101.11 (CH-5); 109.54 (CH-4-furyl); 114.54 (C-4a); 125.22 (C-3-furyl); 127.94 (CH-6); 144.70 (CH-5-furyl); 144.87 (CH-5-furyl); 149.99 (C-4); 151.13 (CH-2); 152.01 (C-7a). ^{31}P{^{1}H} NMR (202.5 MHz, DMSO-d_6): 13.82. HR-ESI-MS: *found*: 410.0756 ([M – H]$^-$, calcd for $C_{16}H_{17}O_8N_3P^+$: 410.0758).

[(5-{[4-(Benzofuran-2-yl)-7*H*-pyrrolo[2,3-*d*]pyrimidin-7-yl]-*β*-D-ribofuranosyl}oxy)methyl]phosphonic acid (7d)

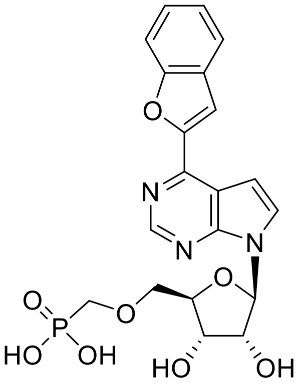

Compound **7d** was prepared according to the general procedure C from corresponding crude starting material **5d** (100 mg, 0.17 mmol) in dry MeCN (6.5 mL). RP-HPFC (C-18, H_2O/MeOH 0 →100 %) and lyophilization from H_2O gave product **7d** as a yellow powder (41 mg, 14% over 2 steps). ^{1}H NMR (500.0 MHz, DMSO-d_6): 3.65 (d, 2H, $J_{H,P}$ = 8.9, CH_2P); 3.71 (dd, 1H, J_{gem} = 10.8, $J_{5'b,4'}$ = 3.9, H-5′b); 3.75 (dd, 1H, J_{gem} = 10.7, $J_{5'a,4'}$ = 3.3, H-5′a); 4.06 (q, 1H, $J_{4'3'}$ = $J_{4',5'}$ = 3.5, H-4′); 4.16 (dd, 1H, $J_{3',2'}$ = 5.1, $J_{3',4'}$ = 3.2, H-3′); 4.51 (dd, 1H, $J_{2',1'}$ = 6.2, $J_{2',3'}$ = 5.1, H-2′); 6.32 (d, 1H, $J_{1',2'}$ = 6.2, H-1′); 7.25 (d, 1H, $J_{5,6}$ = 3.8, H-5); 7.37 (bddd, 1H, $J_{5,4}$ = 7.8, $J_{5,6}$ = 7.2, $J_{5,7}$ = 1.1, H-5-benzofuryl); 7.48 (ddd, 1H, $J_{6,7}$ = 8.6, $J_{6,5}$ = 7.2, $J_{6,4}$ = 1.3, H-6-benzofuryl);. 7.80–7.84 (m, 2H, H-4,7-benzofuryl); 7.93 (d, 1H, $J_{3,7}$ = 1.0, H-3-benzofuryl);. 8.12 (d, 1H, $J_{6,5}$ = 3.8, H-6); 8.88 (s, 1H, H-2). ^{13}C NMR (125.7 MHz, DMSO-d_6): 67.36 (d, $J_{C,P}$ = 160.9, CH_2P); 71.13 (CH-3′); 73.04 (d, $J_{C,P}$ = 12.2, CH_2-5′); 74.19 (CH-2′); 83.54 (CH-4′); 86.59 (CH-1′); 101.85 (CH-5); 109.04 (CH-3-benzofuryl); 112.06 (CH-7-benzofuryl); 114.02 (C-4a); 122.61 (CH-4-benzofuryl); 123.98 (CH-5-benzofuryl); 126.65 (CH-6-benzofuryl); 127.94 (C-3a-benzofuryl); 129.06 (CH-6); 146.30 (C-4); 151.26 (CH-2); 152.77 (C-7a); 154.34 (C-2-benzofuryl); 155.48 (C-7a-benzofuryl). ^{31}P{^{1}H} NMR (202.4 MHz, DMSO-d_6): 17.62. HR-ESI-MS: *found*: 460.0915 ([M – H]$^-$, calcd for $C_{20}H_{19}O_8N_3P^+$: 460.0915).

[(5-{[4-Phenyl-7*H*-pyrrolo[2,3-*d*]pyrimidin-7-yl]-*β*-D-ribofuranosyl}oxy)methyl]phosphonic acid (7e)

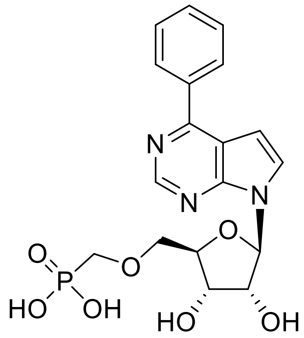

Compound **7e** was prepared according to the general procedure C from corresponding crude starting material **5e** (60 mg, 0.11 mmol) in dry MeCN (4 mL). RP-HPFC (C-18, H_2O/MeOH 0 →100%) and lyophilization from H_2O gave product **7e** as a white powder (26 mg, 9% over 2 steps). ^{1}H NMR (500.0 MHz, DMSO-d_6): 3.62 (d, 2H, $J_{H,P}$ = 8.9, CH_2P); 3.69 (dd, 1H, J_{gem} = 10.8, $J_{5'b,4'}$ = 4.1, H-5′b); 3.73 (dd, 1H, J_{gem} = 10.8, $J_{5'a,4'}$ = 3.4, H-5′a); 4.05 (q, 1H, $J_{4'3'}$ = $J_{4',5'}$ = 3.6, H-4′); 4.15 (dd, 1H, $J_{3',2'}$ = 5.1, $J_{3',4'}$ = 3.2, H-3′); 4.50 (dd, 1H, $J_{2',1'}$ = 6.2, $J_{2',3'}$ = 5.1, H-2′); 6.33 (d, 1H, $J_{1',2'}$ = 6.2, H-1′); 6.96 (d, 1H, $J_{5,6}$ = 3.8, H-5); 7.53 – 7.63 (m, 3H, H-*m*,*p*-Ph); 8.03 (d, 1H, $J_{6,5}$ = 3.8, H-6); 8.15 (m, 2H, H-*o*-Ph); 8.89 (s, 1H, H-2). ^{13}C NMR (125.7 MHz, DMSO-d_6): 67.48 (d, $J_{C,P}$ = 161.7, CH_2P); 71.12 (CH-3′); 73.03 (d, $J_{C,P}$ = 11.9, CH_2-5′); 74.12 (CH-2′); 83.45 (CH-4′); 86.60 (CH-1′); 101.26 (CH-5); 115.50 (C-4a); 128.38 (CH-6); 128.78 (CH-*o*-Ph); 129.09 (CH-*m*-Ph); 130.41 (CH-*p*-Ph); 137.73 (C-*i*-Ph); 151.23 (CH-2); 152.37 (C-7a); 156.15 (C-4). ^{31}P{^{1}H} NMR (202.4 MHz, DMSO-d_6): 17.43. HR-ESI-MS: *found*: 420.0966 ([M – H]$^-$, calcd for $C_{18}H_{19}O_7N_3P^+$: 420.0966).

[(5-{[4-(Naphth-1-yl)-7*H*-pyrrolo[2,3-*d*]pyrimidin-7-yl]-*β*-D-ribofuranosyl}oxy)methyl]phosphonic acid (7f)

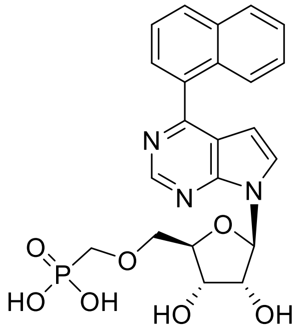

Compound **7f** was prepared according to the general procedure C from the corresponding crude starting material **5f** (90 mg, 0.15 mmol) in dry MeCN (8 mL). RP-HPFC (C-18, H_2O/MeOH 0 →100 %) and lyophilization from H_2O gave product **7f** as a white powder (50 mg, 23% over 2 steps). ^{1}H NMR (500.0 MHz, DMSO-*d*$_6$): 3.62 (d, 2H, $J_{H,P}$ = 8.9, CH_2P); 3.69 (dd, 1H, J_{gem} = 10.7, $J_{5'b,4'}$ = 4.1, H-5′b); 3.73 (dd, 1H, J_{gem} = 10.7, $J_{5'a,4'}$ = 3.4, H-5′a); 4.07 (q, 1H, $J_{4'3'}$ = $J_{4',5'}$ = 3.6, H-4′); 4.16 (dd, 1H, $J_{3',2'}$ = 5.0, $J_{3',4'}$ = 3.2, H-3′); 4.52 (dd, 1H, $J_{2',1'}$ = 6.3, $J_{2',3'}$ = 5.0, H-2′); 6.36 (d, 1H, $J_{1',2'}$ = 6.3, H-1′); 6.39 (d, 1H, $J_{5,6}$ = 3.8, H-5); 7.51 (ddd, 1H, $J_{7,8}$ = 8.5, $J_{7,6}$ = 6.8, $J_{7,5}$ = 1.4, H-7-naphth); 7.59 (ddd, 1H, $J_{6,5}$ = 8.2, $J_{6,7}$ = 6.8, $J_{6,8}$ = 1.3, H-6-naphth); 7.69 (dd, 1H, $J_{3,4}$ = 8.2, $J_{3,2}$ = 7.1, H-3-naphth); 7.77 (dd, 1H, $J_{2,3}$ =7.1, $J_{2,4}$ = 1.3, H-2-naphth); 7.98 (d, 1H, $J_{6,5}$ = 3.8, H-6); 8.04 (dm, 1H, $J_{8,7}$ = 8.4, H-8-naphth); 8.06 (dm, 1H, $J_{5,6}$ = 8.2, H-5-naphth); 8.12 (dm, 1H, $J_{4,3}$ = 8.2, H-4-naphth); 8.99 (s, 1H, H-2). ^{13}C NMR (125.7 MHz, DMSO-*d*$_6$): 67.33 (d, $J_{C,P}$ = 161.0, CH_2P); 71.17 (CH-3′); 73.08 (d, $J_{C,P}$ = 11.9, CH_2-5′); 74.15 (CH-2′); 83.49 (CH-4′); 86.67 (CH-1′); 101.09 (CH-5); 118.25 (C-4a); 125.59 (CH-3-naphth); 125.69 (CH-8-naphth); 126.45 (CH-6-naphth); 126.82 (CH-7-naphth); 128.23 (CH-2-naphth, CH-6); 128.58 (CH-5-naphth); 129.89 (CH-4-naphth); 130.50 (C-8a-naphth); 133.72 (C-4a-naphth); 134.86 (C-1-naphth); 151.09 (CH-2); 151.93 (C-7a); 158.03 (C-4). ^{31}P{^{1}H} NMR (202.4 MHz, DMSO-*d*$_6$): 17.58. HR-ESI-MS: *found*: 470.1120 ([M – H]$^-$, calcd for $C_{22}H_{21}O_7N_3P^+$: 470.1122).

[(5-{[4-(Naphthalen-2-yl)-7*H*-pyrrolo[2,3-*d*]pyrimidin-7-yl]-*β*-D-ribofuranosyl}oxy)methyl]phosphonic acid (7g)

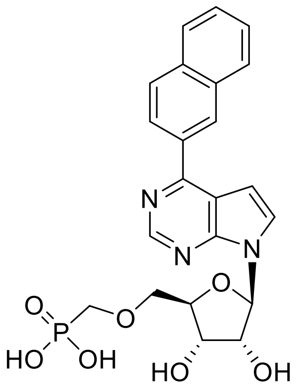

Compound **7g** was prepared according to the general procedure C from the corresponding crude starting material **5g** (80 mg, 0.13 mmol) in dry MeCN (10 mL). RP-HPFC (C-18, H_2O/MeOH 0 →100 %) and lyophilization from H_2O gave product **7g** as a white powder (37 mg, 12% over 3 steps). ^{1}H NMR (500.0 MHz, DMSO-d_6): 3.62 (bd, 2H, $J_{H,P}$ = 8.9, CH_2P); 3.70 (bdd, 1H, J_{gem} = 10.7, $J_{5'b,4'}$ = 4.1, H-5′b); 3.74 (bdd, 1H, J_{gem} = 10.7, $J_{5'a,4'}$ = 3.7, H-5′a); 4.06 (bddd, 1H, $J_{4'5'}$ = 4.1, 3.7, $J_{4',5'}$ = 3.1, H-4′); 4.19 (bdd, 1H, $J_{3',2'}$ = 4.9, $J_{3',4'}$ = 3.1, H-3′); 4.56 (bdd, 1H, $J_{2',1'}$ = 6.3, $J_{2',3'}$ = 4.9, H-2′); 6.37 (d, 1H, $J_{1',2'}$ = 6.3, H-1′); 7.14 (d, 1H, $J_{5,6}$ = 3.8, H-5); 7.59 – 7.65 (m, 2H, H-6,7-naphth); 8.01 (m, 1H, H-5-naphth); 8.09–8.13 (m, 2H, H-6, H-4-naphth); 8.18 (m, 1H, H-8-naphth); 8.31 (dd, 1H, $J_{3,4}$ = 8.6, $J_{3,1}$ = 1.8, H-3-naphth); 8.75 (bd, 1H, $J_{1,3}$ = 1.8, H-1-naphth); 8.94 (s, 1H, H-2). ^{13}C NMR (125.7 MHz, DMSO-d_6): 67.81 (bd, $J_{C,P}$ = 164.3, CH_2P); 71.19 (CH-3′); 73.03 (d, $J_{C,P}$ = 11.2, CH_2-5′); 74.11 (CH-2′); 83.55 (CH-4′); 86.56 (CH-1′); 101.54 (CH-5); 115.70 (C-4a); 125.77 (CH-3-naphth); 126.82 (CH-7-naphth); 127.59 (CH-5-naphth); 127.78 (CH-6-naphth); 128.56 (CH-6); 128.60 (CH-4-naphth); 128.84 (CH-1-naphth); 129.21 (CH-8-naphth); 133.03 (C-8a-naphth); 133.84 (C-4a-naphth); 135.19 (C-2-naphth); 151.22 (CH-2); 152.50 (C-7a); 155.91 (C-4). ^{31}P{^{1}H} NMR (202.4 MHz, DMSO-d_6): 16.62. HR-ESI-MS: *found*: 470.1119 ([M – H]$^-$, calcd for $C_{22}H_{21}O_7N_3P^+$: 470.1122).

[(5-{[4-(Biphenyl-4-yl)-7*H*-pyrrolo[2,3-*d*]pyrimidin-7-yl]-*β*-D-ribofuranosyl}oxy)methyl]phosphonic acid (7h)

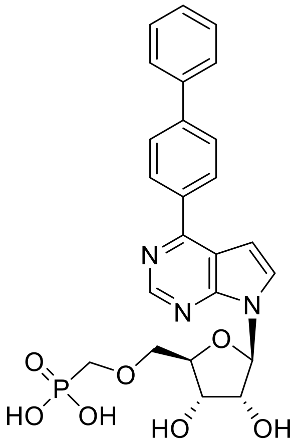

Compound **7h** was prepared according to the general procedure C from corresponding starting material **5h** (160 mg, 0.26 mmol) in dry MeCN (10 mL). RP-HPFC (C-18, H_2O/MeOH 0 →100 %) and lyophilization from H_2O gave product **7h** as a white powder (102 mg, 80%). ^{1}H NMR (500.0 MHz, DMSO-d_6): 3.64 (d, 2H, $J_{H,P}$ = 8.9, CH_2P); 3.70 (dd, 1H, J_{gem} = 10.7, $J_{5'b,4'}$ = 4.1, H-5′b); 3.75 (dd, 1H, J_{gem} = 10.7, $J_{5'a,4'}$ = 3.3, H-5′a); 4.06 (dt, 1H, $J_{4',5'}$ = 4.1, 3.3, $J_{4',3'}$ = 3.3, H-4′); 4.16 (dd, 1H, $J_{3',2'}$ = 4.9, $J_{3',4'}$ = 3.3, H-3′); 4.51 (dd, 1H, $J_{2',1'}$ = 6.2, $J_{2',3'}$ = 4.9, H-2′); 6.34 (d, 1H, $J_{1',2'}$ = 6.2, H-1′); 7.02 (d, 1H, $J_{5,6}$ = 3.8, H-5); 7.43 (m, 1H, H-*p*-Ph); 7.50 – 7.55 (m, 2H, H-*m*-Ph); 7.77–7.80 (m, 2H, H-*o*-Ph); 7.88–7.92 (m, 2H, H-*m*-phenylene); 8.06 (d, 1H, $J_{6,5}$ = 3.8, H-6); 8.26–8.30 (m, 2H, H-*o*-phenylene); 8.91 (s, 1H, H-2). ^{13}C NMR (125.7 MHz, DMSO-d_6): 67.38 (d, $J_{C,P}$ = 159.8, CH_2P); 71.12 (CH-3′); 73.06 (d, $J_{C,P}$ = 11.7, CH_2-5′); 74.14 (CH-2′); 83.45 (CH-4′); 86.63 (CH-1′); 101.28 (CH-5); 115.46 (C-4a); 127.00 (CH-*o*-Ph); 127.34 (CH-*m*-phenylene); 128.19 (CH-*p*-Ph); 128.44 (CH-6); 129.29 (CH-*m*-Ph); 129.40 (CH-*o*-phenylene); 136.75 (C-*i*-phenylene); 139.53 (C-*i*-phenylene); 142.00 (C-*p*-phenylene); 151.26 (CH-2); 152.43 (C-7a); 155.62 (C-4). ^{31}P{^{1}H} NMR (202.4 MHz, DMSO-d_6): 17.55. HR-ESI-MS: *found*: 496.1278 ([M – H]$^-$, calcd for $C_{24}H_{23}O_7N_3P^+$: 496.1279).

[(5-{[4-(Phenanthren-9-yl)-7*H*-pyrrolo[2,3-*d*]pyrimidin-7-yl]-*β*-D-ribofuranosyl}oxy)methyl]phosphonate triethylammonium salt (7i)

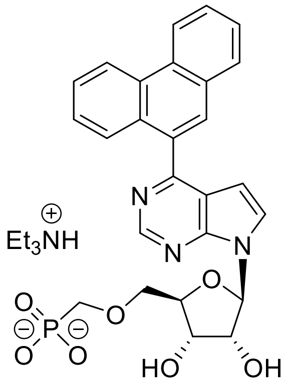

Compound **7i** was prepared according to the general procedure C from the corresponding starting material **5i** (280 mg, 0.43 mmol) in dry MeCN (10 mL). RP-HPFC (C-18, H_2O/MeOH 0 →100 %) and then HPLC (Kinetex C18, H_2O/MeCN 5 →100 %) and lyophilization from H_2O gave product **7i** (1:0.66 of triethylammonium salt*) as a white powder (58 mg, 17%). ^{1}H NMR (500.0 MHz, DMSO-d_6): 1.08 (t, 9H, J_{vic} = 7.2, C**H_3**CH_2N); 2.86 (bs, 6H, (CH_3CH_2N); 3.35 (dd, 1H, J_{gem} = 12.5, $J_{H,P}$ = 9.3, CH_a**H_b**P); 3.41 (dd, 1H, J_{gem} = 12.5, $J_{H,P}$ = 8.0, C**H_a**H_bP); 3.59, 3.66 (2 × dd, 2 × 1H, J_{gem} = 10.5, $J_{5',4'}$ = 3.5, H-5'); 4.03 (td, 1H, $J_{4',5'}$ = 3.5, $J_{4',3'}$ = 2.2, H-4'); 4.24 (dd, 1H, $J_{3',2'}$ = 4.7, $J_{3',4'}$ = 2.2, H-3'); 4.81 (bm, 1H, H-2'); 6.36 (d, 1H, $J_{1',2'}$ = 7.5, H-1'); 6.37 (d, 1H, $J_{5,6}$ = 3.8, H-5); 7.58 (ddd, 1H, $J_{7,8}$ = 8.2, $J_{7,6}$ = 6.9, $J_{7,5}$ = 1.2, H-7-phenanthryl); 7.66 (ddd, 1H, $J_{2,1}$ = 8.0, $J_{2,3}$ = 7.0, $J_{2,4}$ = 1.1, H-2-phenanthryl); 7.70 (ddd, 1H, $J_{6,5}$ = 8.3, $J_{6,7}$ = 6.9, $J_{6,8}$ = 1.3, H-6-phenanthryl); 7.76 (ddd, 1H, $J_{3,4}$ = 8.4, $J_{3,2}$ = 7.0, $J_{3,1}$ = 1.4, H-3-phenanthryl); 8.03 (dd, 1H, $J_{8,7}$ = 8.2, $J_{8,6}$ = 1.3, H-8-phenanthryl); 8.07 (dd, 1H, $J_{1,2}$ = 8.0, $J_{1,3}$ = 1.4, H-1-phenanthryl); 8.08 (s, 1H, H-10-phenanthryl); 8.10 (d, 1H, $J_{6,5}$ = 3.8, H-6); 8.89 (m, 1H, H-4-phenanthryl); 8.92 (m, 1H, H-5-phenanthryl); 8.98 (s, 1H, H-2). ^{13}C NMR (125.7 MHz, DMSO-d_6): 9.24 (**C**H_3CH_2N); 45.47 (CH_3**C**H_2N); 68.95 (d, $J_{C,P}$ = 157.1, CH_2P); 71.55 (CH-3'); 72.72 (d, $J_{C,P}$ = 10.6, CH_2-5'); 73.60 (CH-2'); 83.93 (CH-4'); 85.99 (CH-1'); 100.90 (CH-5); 118.38 (C-4a); 123.05 (CH-4-phenanthryl); 123.46 (CH-5-phenanthryl); 126.62 (CH-8-phenanthryl); 127.05 (CH-7-phenanthryl); 127.24 (CH-6-phenanthryl); 127.39 (CH-2-phenanthryl); 128.04 (CH-3-phenanthryl); 128.64 (CH-6); 129.18 (CH-10-phenanthryl); 129.37 (C-8a-phenanthryl); 129.44 (CH-1-phenanthryl); 130.27 (C-4a-phenanthryl); 130.42 (C-4b-phenanthryl); 130.78 (C-10a-phenanthryl); 133.64 (C-9-phenanthryl); 150.97 (CH-2); 152.19 (C-7a); 157.86 (C-4). ^{31}P{^{1}H} NMR (202.4 MHz, D_2O): 14.75. HR-ESI-MS: *found*: 520.12746 ([M – H]$^-$, calcd for $C_{26}H_{23}O_7N_3P^-$: 520.12791).

* HPLC column (Phenomenex, Luna Omega Polar C18 column of 150 x 21.2 mm) is mainly used for purifications of mono- or triphosphates in 0.1 M TEAB buffer, the triethylammonium

residue stays on the column, therefore residual peaks of triethylammonium salt are observed in NMR, giving **7i** as a mixture of free acid and triethylammonium salt form.

[(5-{[4-(Dibenzofuran-4-yl)-7*H*-pyrrolo[2,3-*d*]pyrimidin-7-yl]-*β*-D-ribofuranosyl}oxy)methyl]phosphonic acid (7j)

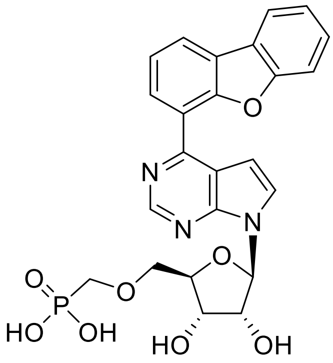

Compound **7j** was prepared according to the general procedure C from the corresponding starting material **5j** (160 mg, 0.25 mmol) in dry MeCN (10 mL). RP-HPFC (C-18, H_2O/MeOH 0 →100 %) and lyophilization from H_2O gave product **7j** as a white powder (103 mg, 80%). ^{1}H NMR (500.0 MHz, DMSO-*d6*): 3.62 (dd, 1H, J_{gem} = 13.6, $J_{H,P}$ = 8.8, CH_2P-a); 3.64 (dd, 1H, J_{gem} = 13.6, $J_{H,P}$ = 8.8, CH_2P-b); 3.70 (dd, 1H, J_{gem} = 10.7, $J_{5'b,4'}$ = 4.1, H-5′b); 3.74 (dd, 1H, J_{gem} = 10.7, $J_{5'a,4'}$ = 3.5, H-5′a); 4.08 (q, 1H, $J_{4'3'}$ = $J_{4',5'}$ = 3.6, H-4′); 4.17 (dd, 1H, $J_{3',2'}$ = 5.0, $J_{3',4'}$ = 3.1, H-3′); 4.54 (dd, 1H, $J_{2',1'}$ = 6.3, $J_{2',3'}$ = 5.1, H-2′); 6.37 (d, 1H, $J_{1',2'}$ = 6.3, H-1′); 6.70 (d, 1H, $J_{5,6}$ = 3.8, H-5); 7.46 (btd, 1H, $J_{8,7}$ = $J_{8,9}$ = 7.5, $J_{8,6}$ = 1.0, H-8-dibenzofuryl); 7.56 (ddd, 1H, $J_{7,6}$ = 8.3, $J_{7,8}$ = 7.3, $J_{7,9}$ = 1.4, H-7-dibenzofuryl); 7.62 (t, 1H, $J_{2,3}$ = $J_{2,1}$ = 7.6, H-2-dibenzofuryl); 7.72 (dt, 1H, $J_{6,7}$ =8.2, $J_{6,8}$ = $J_{6,9}$ = 0.8, H-6-dibenzofuryl); 7.98 (dd, 1H, $J_{3,2}$ = 7.6, $J_{3,1}$ = 1.3, H-3-dibenzofuryl); 8.03 (d, 1H, $J_{6,5}$ = 3.8, H-6); 8.25 (dm, 1H, $J_{9,8}$ = 7.7, H-9-dibenzofuryl); 8.37 (dd, 1H, $J_{1,2}$ = 7.7, $J_{1,3}$ = 1.3, H-1-dibenzofuryl); 9.00 (s, 1H, H-2). ^{13}C NMR (125.7 MHz, DMSO-*d6*): 67.37 (d, $J_{C,P}$ = 161.1, CH_2P); 71.18 (CH-3′); 73.08 (d, $J_{C,P}$ = 11.9, CH_2-5′); 74.12 (CH-2′); 83.51 (CH-4′); 86.59 (CH-1′); 101.88 (CH-5); 112.05 (CH-6-dibenzofuryl); 117.42 (C-4a); 121.56 (CH-9-dibenzofuryl); 122.64 (C-4-dibenzofuryl); 122.98 (CH-1-dibenzofuryl); 123.44 (C-9a-dibenzofuryl); 123.62 and 123.64 (CH-2,8-dibenzofuryl); 124.94 (C-9b-dibenzofuryl); 128.06 (CH-6); 128.19 (CH-7-dibenzofuryl); 128.70 (CH-3-dibenzofuryl); 151.38 (CH-2); 152.03 (C-7a); 152.93 (C-4a-dibenzofuryl); 153.65 (C-4); 155.70 (C-5a-dibenzofuryl). ^{31}P{^{1}H} NMR (202.4 MHz, DMSO-*d*6): 17.49. HR-ESI-MS: *found*: 510.1069 ([M – H]$^-$, calcd for $C_{22}H_{21}O_7N_3P^+$: 510.1071).

6-(2,4-Dichloro)-7-(2,3-*O*-isopropylidene-β-D-ribofuranosyl)-7*H*-pyrrolo[2,3-*d*]pyrimidine (9)

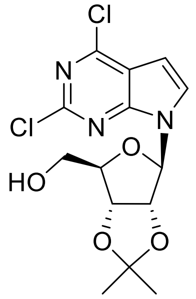

Compound **9** was prepared according to the published procedure.[3]

6-(2-Chloro-4-(furan-2-yl)-7-(2,3-*O*-isopropylidene-β-D-ribofuranosyl)-7*H*-pyrrolo[2,3-*d*]pyrimidine (10b)

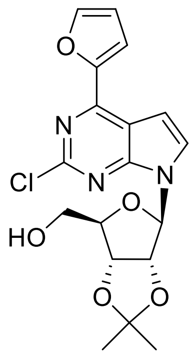

Compound **9** (231.5 mg, 0.64 mmol) was reacted with 2-furanylboronic acid (79.1 mg, 0.71 mmol) for 10 min at 100 °C according to general procedure A. HPFC (SiO_2, cHex/EtOAc 5.67:1 → 1:1) gave **10b** (190.6 mg, 76 %) as a white foam. ^{1}H NMR (500 MHz, DMSO-d_6): 1.33 and 1.56 (2×s, 2×3H, $(CH_3)_2C$); 3.52 – 3.62 (m, 2H, H-5´); 4.18 (td, 1H, $J_{4',5'a} = J_{4',5'b}$ = 4.8 Hz, $J_{4',3'}$ = 3.1 Hz, H-4´); 4.94 (dd, 1H, $J_{3',2'}$ = 6.3 Hz, $J_{3',4'}$ = 3.1 Hz, H-3´); 5.10 (bs, 1H, OH-5´); 5.20 (dd, 1H, $J_{2',3'}$ = 6.3 Hz, $J_{2',1'}$ = 3.2 Hz, H-2´); 6.32 (d, 1H, $J_{1',2'}$ = 3.2 Hz, H-1´); 6.83 (dd, 1H, $J_{4,3}$ = 3.6 Hz, $J_{4,5}$ = 1.8 Hz, H-4-furyl); 7.11 (d, 1H, $J_{5,6}$ = 3.8 Hz, H-5); 7.55 (dd, 1H, $J_{3,4}$ = 3.6 Hz, $J_{3,5}$ = 0.8 Hz, H-3-furyl); 7.96 (d, 1H, $J_{6,5}$ = 3.8 Hz, H-6); 8.11 (dd, 1H, $J_{5,4}$ = 1.8 Hz, $J_{5,3}$ = 0.8 Hz, H-5-furyl); ^{13}C NMR (125.7 MHz, DMSO-d_6): 25.22 and 27.11 ((**C**$H_3)_2C$); 61.47 (CH_2-5´); 81.03 (CH-3´); 83.62 (CH-2´); 85.93 (CH-4´); 88.76 (CH-1´); 101.91 (C-5); 111.61 ($(CH_3)_2$**C**); 113.08 (CH-4-furyl); 113.35 (C-4a); 115.16 (CH-3-furyl); 129.11 (CH-6); 147.34 (CH-5-furyl); 148.12 (C-4); 150.97 (C-2-furyl);

152.39 (C-2); 152.93 (C-7a). HR-ESI-MS: *found*: 392.1009 ([M + H]$^+$, calcd for $C_{18}H_{19}O_5N_3Cl^+$: 392.1008); HR-ESI-MS: *found*: 414.0828 ([M + Na]$^+$, calcd for $C_{18}H_{18}O_5N_3ClNa^+$: 414.0827).

6-(4-(Benzofuran-2-yl)-2-chloro-7-(2,3-*O*-isopropylidene-β-D-ribofuranosyl)-7*H*-pyrrolo[2,3-*d*]pyrimidine (10d)

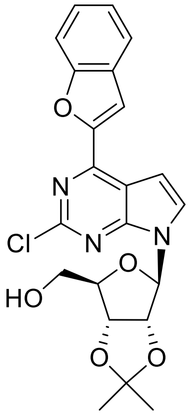

Compound **9** (272.2 mg, 0.76 mmol) was reacted with 2-benzofurylboronic acid (134.6 mg, 0.83 mmol) for 10 min at 100 °C according to general procedure A. 3 HPFC (SiO_2, cHex/EtOAc 5.67:1 → 1:1) gave **10d** (144.5 mg, 43 %) as a yellow oil. ^{1}H NMR (500 MHz, DMSO-d$_6$): 1.34 and 1.57 (2×s, 2×3H, $(CH_3)_2C$); 3.57 (bdt, 1H, J_{gem} = 11.6 Hz, $J_{5'a,4'}$ = $J_{5'a,OH}$ = 5.1 Hz, H-5´a); 3.60 (bdt, 1H, J_{gem} = 11.6 Hz, $J_{5'b,4'}$ = $J_{5'b,OH}$ = 5.1 Hz, H-5´b); 4.20 (td, 1H, $J_{4',5'a}$ = $J_{4',5'b}$ = 4.8 Hz, $J_{4',3'}$ = 3.0 Hz, H-4´); 4.97 (dd, 1H, $J_{3',2'}$ = 6.3 Hz, $J_{3',4'}$ = 3.0 Hz, H-3´); 5.12 (t, 1H, $J_{OH,5'a}$ = $J_{OH,5'b}$ = 5.4 Hz, OH-5´); 5.24 (dd, 1H, $J_{2',3'}$ = 6.3 Hz, $J_{2',1'}$ = 3.2 Hz, H-2´); 6.32 (d, 1H, $J_{1',2'}$ = 3.2 Hz, H-1´); 7.33 (d, 1H, $J_{5,6}$ = 3.8 Hz, H-5); 7.39 (m, 1H, H-5-benzofuryl); 7.52 (ddd, 1H, $J_{6,7}$ = 8.2 Hz, $J_{6,5}$ = 7.2 Hz, $J_{6,4}$ = 1.3 Hz, H-6-benzofuryl); 7.78 - 7.87 (m, 2H, H-4,7-benzofuryl); 8.01 (d, 1H, $J_{3,LR}$ = 1.0 Hz, H-3-benzofuryl); 8.06 (d, 1H, $J_{6,5}$ = 3.8 Hz, H-6); ^{13}C NMR (125.7 MHz, DMSO-d$_6$): 25.24 and 27.11 ((**C**H$_3$)$_2$C); 61.47 (CH$_2$-5´); 81.06 (CH-3´); 83.63 (CH-2´); 86.03 (CH-4´); 88.90 (CH-1´); 102.18 (C-5); 110.77 (CH-3-benzofuryl); 112.04 (CH-7-benzofuryl); 113.04 (C-4a); 113.37 ((CH$_3$)$_2$**C**); 122.73 (CH-4-furyl); 124.01 (CH-5-benzofuryl); 127.17 (CH-6-benzofuryl); 127.61 (C-3a-benzofuryl); 129.83 (CH-6); 148.09 (C-4); 152.35 and 152.40 (C-2, C-2-benzofuryl); 153.25 (C-7a); 155.46 (C-7a-benzofuryl). HR-ESI-MS: *found*: 442.1163 ([M + H]$^+$, calcd for $C_{22}H_{21}O_5N_3Cl^+$: 442.1164); HR-ESI-MS: *found*: 464.0982 ([M + Na]$^+$, calcd for $C_{22}H_{20}O_5N_3ClNa^+$: 464.0984).

6-(2-Chloro-4-(naphth-1-yl)-7-(2,3-*O*-isopropylidene-β-D-ribofuranosyl)-7*H*-pyrrolo[2,3-*d*]pyrimidine (10f)

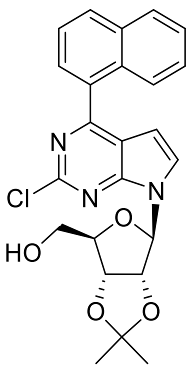

Compound **9** (311.2 mg, 0.86 mmol) was reacted with naphthalene-1-boronic acid (163.5 mg, 0.95 mmol) for 10 min at 100 °C according to general procedure A. HPFC (SiO_2, cHex/EtOAc 5.67:1 → 1:1) gave **10f** (317.5 mg, 81 %) as a yellowish oil. ^{1}H NMR (500 MHz, DMSO-d$_6$): 1.35 and 1.59 (2×s, 2×3H, $(CH_3)_2C$); 3.59 (bdt, 1H, J_{gem} = 11.8 Hz, $J_{5'a,OH}$ = $J_{5'a,4'}$ = 5.1 Hz, H-5´a); 3.61 (bdt, 1H, J_{gem} = 11.7 Hz, $J_{5'b,OH}$ = $J_{5'b,4'}$ = 5.1 Hz, H-5´b); 4.22 (td, 1H, $J_{4',5'a}$ = $J_{4',5'b}$ = 4.8 Hz, $J_{4',3'}$ = 3.0 Hz, H-4´); 4.97 (dd, 1H, $J_{3',2'}$ = 6.3 Hz, $J_{3',4'}$ = 3.1 Hz, H-3´); 5.11 (t, 1H, $J_{OH,5'a}$ = $J_{OH,5'b}$ = 5.4 Hz, OH-5´); 5.25 (dd, 1H, $J_{2',3'}$ = 6.3 Hz, $J_{2',1'}$ = 3.2 Hz, H-2´); 6.37 (d, 1H, $J_{1',2'}$ = 3.2 Hz, H-1´); 6.49 (d, 1H, $J_{5,6}$ = 3.8 Hz, H-5); 7.54 (ddd, 1H, $J_{7,8}$ = 8.5 Hz, $J_{7,6}$ = 6.8 Hz, $J_{7,5}$ = 1.4 Hz, H-7-naphthyl); 7.61 (ddd, 1H, $J_{6,5}$ = 8.2 Hz, $J_{6,7}$ = 6.8 Hz, $J_{6,8}$ = 1.3 Hz, H-6-naphthyl); 7.70 (dd, 1H, $J_{3,4}$ = 8.2 Hz, $J_{3,2}$ = 7.1 Hz, H-3-naphthyl); 7.78 (dd, 1H, $J_{2,3}$ = 7.1 Hz, $J_{2,4}$ = 1.3 Hz, H-2-naphthyl); 7.94 (d, 1H, $J_{6,5}$ = 3.8 Hz, H-6); 8.02 (bd, 1H, $J_{8,7}$ = 8.5 Hz, H-8-naphthyl); 8.08 (bd, 1H, $J_{4,5}$ = 8.2 Hz, H-5-naphthyl); 8.16 (bd, 1H, $J_{4,3}$ = 8.2 Hz, H-4-naphthyl); ^{13}C NMR (125.7 MHz, DMSO-d$_6$): 25.24 and 27.14 ($(\mathbf{C}H_3)_2C$); 61.50 (CH_2-5´); 81.06 (CH-3´); 83.68 (CH-2´); 85.97 (CH-4´); 88.95 (CH-1´); 101.58 (CH-5); 113.37 ($(CH_3)_2\mathbf{C}$); 117.44 (C-4a); 125.18 (CH-8-naphthyl); 125.40 (CH-3-naphthyl); 126.44 (CH-6-naphthyl); 126.98 (CH-7-naphthyl); 128.45 and 128.46 (CH-2,5-naphthyl); 129.13 (CH-6); 130.05 (C-8a-naphthyl); 130.39 (CH-4-naphthyl); 133.24 (C-1-naphthyl); 133.46 (C-4a-naphthyl); 152.20 (C-2); 152.37 (C-7a); 160.23 (C-4). HR-ESI-MS: *found*: 452.1370 ([M + H]$^+$, calcd for $C_{24}H_{23}O_4N_3Cl^+$: 452.1372); HR-ESI-MS: *found*: 474.1188 ([M + Na]$^+$, calcd for $C_{24}H_{22}O_4N_3ClNa^+$: 474.1191).

6-(2-Chloro-4-(naphthalen-2-yl)-7-(2,3-*O*-isopropylidene-β-D-ribofuranosyl)-7*H*-pyrrolo[2,3-*d*]pyrimidine (10g)

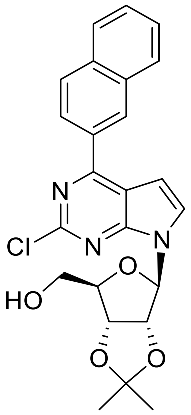

Compound **9** (302.1 mg, 0.84 mmol) was reacted with naphthalene-2-boronic acid (158.7 mg, 0.92 mmol) for 10 min at 100 °C according to general procedure A. HPFC (SiO_2, cHex/EtOAc 5.67:1 → 1:1) and RP-HPFC (C-18, H_2O/MeOH 0 → 100 %) gave **10g** (273.9 mg, 72 %) as a white foam. ^{1}H NMR (500 MHz, DMSO-d$_6$): 1.35 and 1.58 (2×s, 2×3H, $(CH_3)_2C$); 3.58 (dt, 1H, J_{gem} = 11.4 Hz, $J_{5'a,OH}$ = $J_{5'a,4'}$ = 5.1 Hz, H-5´a); 3.61 (dt, 1H, J_{gem} = 11.4 Hz, $J_{5'b,OH}$ = $J_{5'b,4'}$ = 5.2 Hz, H-5´b); 4.21 (td, 1H, $J_{4',5'a}$ = $J_{4',5'b}$ = 4.8 Hz, $J_{4',3'}$ = 3.0 Hz, H-4´); 4.98 (dd, 1H, $J_{3',2'}$ = 6.3 Hz, $J_{3',4'}$ = 3.1 Hz, H-3´); 5.12 (t, 1H, $J_{OH,5'a}$ = $J_{OH,5'b}$ = 5.4 Hz, OH-5´); 5.26 (dd, 1H, $J_{2',3'}$ = 6.3 Hz, $J_{2',1'}$ = 3.2 Hz, H-2´); 6.36 (d, 1H, $J_{1',2'}$ = 3.2 Hz, H-1´); 7.28 (d, 1H, $J_{5,6}$ = 3.9 Hz, H-5); 7.63 (ddd; 1H, $J_{7,8}$ = 7.9 Hz, $J_{7,6}$ = 6.9 Hz, $J_{7,5}$ = 1.9 Hz, H-7-naphthyl); 7.66 (ddd; 1H, $J_{6,5}$ = 8.0 Hz, $J_{6,7}$ = 6.9 Hz, $J_{6,8}$ = 1.6 Hz, H-6-naphthyl); 8.04 (m, 1H, H-5-naphthyl); 8.05 (d, 1H, $J_{6,5}$ = 3.9 Hz, H-6); 8.13 (d, 1H, $J_{4,3}$ =8.7 Hz, H-4-naphthyl); 8.22 (m, 1H, H-8-naphthyl); 8.27 (dd, 1H, $J_{3,4}$ = 8.6 Hz, $J_{3,1}$ = 1.9 Hz, H-3-naphthyl); 8.77 (d, 1H, $J_{1,3}$ = 1.9 Hz, H-1-naphthyl); ^{13}C NMR (125.7 MHz, DMSO-d$_6$): 25.24 and 27.12 ((**C**H$_3$)$_2$C); 61.50 (CH$_2$-5´); 81.09 (CH-3´); 83.67 (CH-2´); 86.02 (CH-4´); 88.94 (CH-1´); 102.09 (CH-5); 113.34 ((CH$_3$)$_2$**C**); 114.74 (C-4a); 125.33 (CH-3-naphthyl); 126.82 (CH-7-naphthyl); 127.63 (CH-5-naphthyl); 127.91 (CH-6-naphthyl); 128.66 (CH-4-naphthyl); 128.27 and 129.29 (CH-1,8-naphthyl); 129.35 (CH-6); 132.75 (C-8a-naphthyl); 133.44 (C-2-naphthyl); 134.00 (C-4a-naphthyl); 152.49 (C-2); 152.99 (C-7a); 158.21 (C-4). HR-ESI-MS: *found*: 452.1373 ([M + H]$^+$, calcd for $C_{24}H_{23}O_4N_3Cl^+$: 452.1372); HR-ESI-MS: *found*: 474.1193 ([M + Na]$^+$, calcd for $C_{24}H_{22}O_4N_3ClNa^+$: 474.1191).

Diisopropyl[(5-{2′,3′-*O*-isopropylidene-[2-chloro-4-(furan-2-yl)-7*H*-pyrrolo[2,3-*d*]pyrimidin-7-yl]-β-D-ribofuranosyl}oxy)methyl]phosphonate (11b)

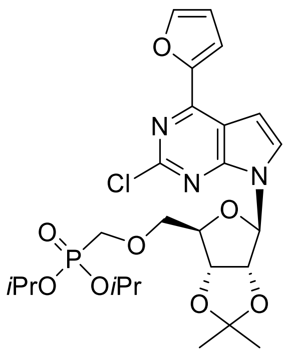

Compound **11b** was prepared according to the general procedure G from **10b** (291.3 mg, 0.74 mmol) in dry DMF (9 mL). After 1 h, volatiles were removed in vacuo by co-distillation with toluene. HPFC (SiO_2, cyclohexane/EtOAc 0 $\rightarrow$ 80%) gave crude product **11b** as a yellowish oil (119.8 mg), which was used directly for the deprotection step. HR-ESI-MS: *found*: 570.1768 ($[M + H]^+$, calcd for $C_{25}H_{34}O_8N_3ClP^+$: 570.1767).

Diisopropyl[(5-{2′,3′-*O*-isopropylidene-[4-(benzofuran-2-yl)-2-chloro-7*H*-pyrrolo[2,3-*d*]pyrimidin-7-yl]-β-D-ribofuranosyl}oxy)methyl]phosphonate (11d)

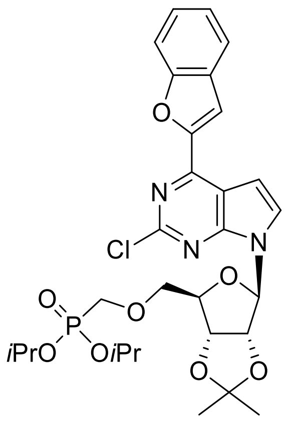

Compound **11d** was prepared according to the general procedure B from **10d** (350.6 mg, 0.79 mmol) in dry DMF (9 mL). After 1 h, volatiles were removed in vacuo by co-distillation with toluene. HPFC (SiO_2, cyclohexane/EtOAc 0 $\rightarrow$ 80%) gave desired crude **11d** as a yellow glassy solid (115.7 mg), which was used directly for the deprotection step. HR-ESI-MS: *found*: 620.1920 ($[M + H]^+$, calcd for $C_{29}H_{36}O_8N_3ClP^+$: 620.1923).

Diisopropyl[(5-{2′,3′-*O*-isopropylidene-[2-chloro-4-(naphth-1-yl)-7*H*-pyrrolo[2,3-*d*]pyrimidin-7-yl]-β-D-ribofuranosyl}oxy)methyl]phosphonate (11f)

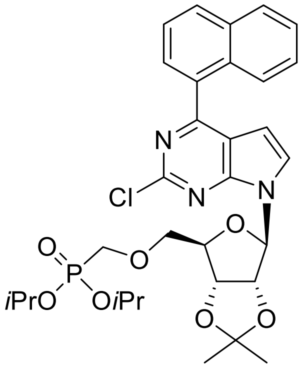

Compound **11f** was prepared according to the general procedure B from **10f** (302.1 mg, 0.67 mmol) in dry DMF (9 mL). After 1 h, volatiles were removed in vacuo by co-distillation with toluene. HPFC (SiO_2, cyclohexane/EtOAc 0 $\rightarrow$ 80%) gave desired crude **11f** as a colorless oil (137.6 mg), which was used directly for the deprotection step. HR-ESI-MS: *found*: 630.2130 ($[M + H]^+$, calcd for $C_{31}H_{38}O_7N_3ClP^+$: 630.2130).

Diisopropyl[(5-{2′,3′-*O*-isopropylidene-[2-chloro-4-(naphthalen-2-yl)-7*H*-pyrrolo[2,3-*d*]pyrimidin-7-yl]-β-D-ribofuranosyl}oxy)methyl]phosphonate (11g)

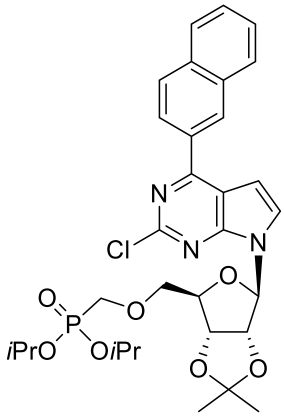

Compound **11g** was prepared according to the general procedure B from **10g** (268.0 mg, 0.59 mmol) in dry DMF (8 mL). After 1 h, volatiles were removed in vacuo by co-distillation with toluene. HPFC (SiO_2, cyclohexane/EtOAc 0 $\rightarrow$ 80%) gave desired crude **11g** as a colorless oil (100.4 mg), which was used directly for the deprotection step. HR-ESI-MS: *found*: 630.2134 ($[M + H]^+$, calcd for $C_{31}H_{38}O_7N_3ClP^+$: 630.2130).

[(5-{[2-Chloro-4-(furan-2-yl)-7*H*-pyrrolo[2,3-*d*]pyrimidin-7-yl]-*β*-D-ribofuranosyl}oxy)methyl]phosphonate triethylammonium salt (12b)

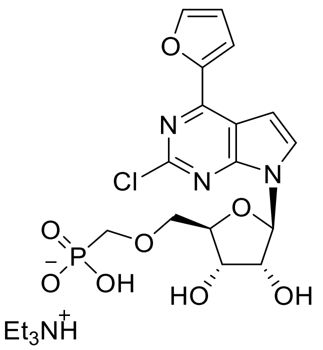

Compound **12b** was prepared according to the general procedure C from the corresponding crude starting material **11b** (119.8 mg, 0.21 mmol) in dry MeCN (7 mL). RP-HPFC (C-18, H_2O/MeOH 0 →100 %), HPLC (C-18, H_2O + 0.05% TFA/MeCN 0 → 80 %) and lyophilization from H_2O gave product **12b** (1:0.26 of triethylammonium salt*) as a pale-yellow powder (9.8 mg, 3 % over 2 steps). ^{1}H NMR (500 MHz, DMSO-d$_6$): 3.61 (d, 2H, $J_{CH2,P}$ = 9.0 Hz, OCH$_2$P); 3.65 – 3.73 (m, 2H, H-5´); 4.05 (q, 1H, $J_{4´,5´a}$ = $J_{4´,5´b}$ = $J_{4´,3´}$ = 3.3 Hz, H-4´); 4.13 (dd, 1H, $J_{3´,2´}$ = 5.0 Hz, $J_{3´,4´}$ = 2.8 Hz, H-3´); 4.48 (dd, 1H, $J_{2´,1´}$ = 6.4 Hz, $J_{2´,3´}$ = 5.0 Hz, H-2´); 6.17 (d, 1H, $J_{1´,2´}$ = 6.4 Hz, H-1´); 6.81 (dd, 1H, $J_{4,3}$ = 3.5 Hz, $J_{4,5}$ = 1.7 Hz, H-4-furyl); 7.04 (d, 1H, $J_{5,6}$ = 3.8 Hz, H-5); 7.51 (d, 1H, $J_{3,4}$ = 3.5 Hz, H-3-furyl); 8.06 (d, 1H, $J_{6,5}$ = 3.8 Hz, H-6); 8.10 (bd, 1H, $J_{5,4}$ = 1.7 Hz, H-5-furyl); ^{13}C NMR (125.7 MHz, DMSO-d$_6$): 67.37 (d, $J_{C,P}$ = 160.8 Hz, OCH$_2$P); 71.05 (CH-3´); 72.77 (d, $J_{C,P}$ = 12.3 Hz, CH$_2$-5´); 74.00 (CH-2´); 83.68 (CH-4´); 86.29 (CH-1´); 101.91 (C-5); 111.57 (C-4a); 113.00 (CH-4-furyl); 114.86 (CH-3-furyl); 129.06 (CH-6); 147.21 (CH-5-furyl); 147.85 (C-4); 151.13 (C-2-furyl); 152.29 (C-2); 153.65 (C-7a); ^{31}P NMR (202.4 MHz, DMSO-d$_6$): 17.27 (bs, 1P, OCH$_2$P). HR-ESI-MS: *found*: 444.0367 ([M – H]$^-$, calcd for $C_{16}H_{16}O_8N_3ClP^-$: 444.0369).

*HPLC column (Kinetex EVO, C18 100 Å) is mainly used for purification of mono- or triphosphates in 0.1 M triethylammonium bicarbonate buffer, the triethylammonium residue stays on the column, therefore residual peaks of triethylammonium salt are observed in NMR, giving **12b** as a mixture of free acid and triethylammonium salt form.

[(5-{[4-(Benzofuran-2-yl)-2-chloro-7*H*-pyrrolo[2,3-*d*]pyrimidin-7-yl]-*β*-D-ribofuranosyl}oxy)methyl]phosphonic acid (12d)

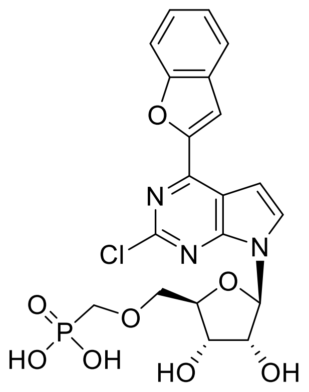

Compound **12d** was prepared according to the general procedure C from the corresponding crude starting material **11d** (209.0 mg, 0.34 mmol) in dry MeCN (9 mL). RP-HPFC (C-18, H_2O/MeOH 0 →100 %), HPLC (C-18, H_2O + 0.05% TFA/MeCN 0 → 80 %) and lyophilization from H_2O gave product **12d** as a yellow powder (14.8 mg, 2 % over 2 steps). ^{1}H NMR (500 MHz, DMSO-d$_6$): 3.65 (d, 2H, $J_{CH2,P}$ = 8.9 Hz, OCH$_2$P); 3.71 (dd, 1H, J_{gem} = 10.8 Hz, $J_{5'a,4'}$ = 3.8 Hz, H-5´a); 3.74 (dd, 1H, J_{gem} = 10.8 Hz, $J_{5'b,4'}$ = 3.3 Hz, H-5´b); 4.08 (q, 1H, $J_{4',5'a}$ = $J_{4',5'b}$ = $J_{4',3'}$ = 3.3 Hz, H-4´); 4.14 (dd, 1H, $J_{3',2'}$ = 5.0 Hz, $J_{3',4'}$ = 2.9 Hz, H-3´); 4.48 (bdd, 1H, $J_{2',1'}$ = 6.4 Hz, $J_{2',3'}$ = 5.0 Hz, H-2´); 6.21 (d, 1H, $J_{1',2'}$ = 6.4 Hz, H-1´); 7.27 (d, 1H, $J_{5,6}$ = 3.8 Hz, H-5); 7.38 (btd, 1H, $J_{5,4}$ = $J_{5,6}$ = 7.6 Hz, $J_{5,7}$ = 1.0 Hz, H-5-benzofuryl); 7.51 (ddd, 1H, $J_{6,7}$ = 8.5 Hz, $J_{6,5}$ = 7.2 Hz, $J_{6,4}$ = 1.3 Hz, H-6-benzofuryl); 7.81 – 7.85 (m, 2H, H-4,7-benzofuryl); 7.99 (d, 1H, $J_{3,LR}$ = 0.9 Hz, H-3-benzofuryl); 8.15 (d, 1H, $J_{6,5}$ = 3.8 Hz, H-6); ^{13}C NMR (125.7 MHz, DMSO-d$_6$): 67.13 (d, $J_{C,P}$ = 160.9 Hz, OCH$_2$P); 71.00 (CH-3´); 72.80 (d, $J_{C,P}$ = 12.5 Hz, CH$_2$-5´); 74.05 (CH-2´); 83.70 (CH-4´); 86.47 (CH-1´); 102.19 (C-5); 110.57 (CH-3-benzofuryl); 112.00 (CH-7-benzofuryl); 112.97 (C-4a); 122.71 (CH-4-benzofuryl); 123.99 (CH-5-benzofuryl); 127.10 (CH-6-benzofuryl); 127.61 (C-3a-benzofuryl); 129.69 (CH-6); 147.84 (C-4); 152.30 (C-2); 152.59 (C-2-benzofuryl); 153.96 (C-7a); 155.45 (C-7a-benzofuryl); ^{31}P NMR (202.4 MHz, DMSO-d$_6$): 17.61 (s, 1P, OCH$_2$P). HR-ESI-MS: *found*: 494.0528 ([M – H]$^-$, calcd for $C_{20}H_{18}O_8N_3ClP^-$: 494.0526).

[(5-{[2-Chloro-4-(naphth-1-yl)-7*H*-pyrrolo[2,3-*d*]pyrimidin-7-yl]-*β*-D-ribofuranosyl}oxy)methyl]phosphonate triethylammonium salt (12f)

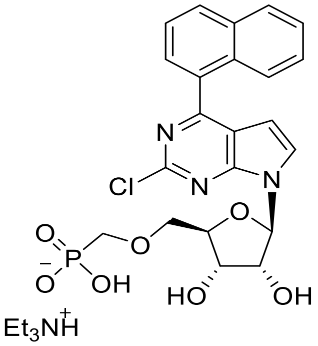

Compound **12f** was prepared according to the general procedure C from the corresponding crude starting material **11f** (137.6 mg, 0.22 mmol) in dry MeCN (7 mL). RP-HPFC (C-18, H_2O/MeOH 0 →100 %), HPLC (C-18, H_2O + 0.05% TFA/MeCN 0 → 80 %) and lyophilization from H_2O gave product **12f** (1:0.21 of triethylammonium salt*) as a white powder (17.7 mg, 5 % over 2 steps). ^{1}H NMR (500 MHz, DMSO-d$_6$): 3.54 – 3.65 (m, 2H, PCH_2O); 3.65 – 3.74 (m, 2H, H-5´); 4.08 (m, 1H, H-4´); 4.14 (m, 1H, H-3´); 4.52 (t, 1H, $J_{2´,3´}$ = $J_{2´,1´}$ = 5.7 Hz, H-2´); 6.25 (d, 1H, $J_{1´,2´}$ = 6.5 Hz, H-1´); 6.41 (d, 1H, $J_{5,6}$ = 3.7 Hz, H-5); 7.54 (ddd, 1H, $J_{7,8}$ = 8.5 Hz, $J_{7,6}$ = 6.8 Hz, $J_{7,5}$ = 1.4 Hz, H-7-naphthyl); 7.60 (ddd, 1H, $J_{6,5}$ = 8.2 Hz, $J_{6,7}$ = 6.8 Hz, $J_{6,8}$ = 1.3 Hz, H-6-naphthyl); 7.69 (dd, 1H, $J_{3,4}$ = 8.2 Hz, $J_{3,2}$ = 7.1 Hz, H-3-naphthyl); 7.78 (dd, 1H, $J_{2,3}$ = 7.1 Hz, $J_{2,4}$ = 1.3 Hz, H-2-naphthyl); 8.02 (bd, 1H, $J_{8,7}$ = 8.5 Hz, H-8-naphthyl); 8.04 (d, 1H, $J_{6,5}$ = 3.7 Hz, H-6); 8.07 (bd, 1H, $J_{4,5}$ = 8.2 Hz, H-5-naphthyl); 8.15 (bd, 1H, $J_{4,3}$ = 8.2 Hz, H-4-naphthyl); ^{13}C NMR (125.7 MHz, DMSO-d$_6$): 66.95 (PCH_2O); 71.10 (CH-3´); 72.08 (CH_2-5´); 74.02 (CH-2´); 83.74 (CH-4´); 86.45 (CH-1´); 101.50 (CH-5); 117.35 (C-4a); 125.20 (CH-8-naphthyl); 125.40 (CH-3-naphthyl); 126.43 (CH-6-naphthyl); 126.98 (CH-7-naphthyl); 128.43 and 128.48 (CH-2,5-naphthyl); 129.05 (CH-6); 130.05 (C-8a-naphthyl); 130.35 (CH-4-naphthyl); 133.34 and 133.37 (C-1,4a-naphthyl); 152.16 (C-2); 152.09 (C-7a); 159.91 (C-4); ^{31}P NMR (202.4 MHz, DMSO-d$_6$): 17.34 (s, 1P, PCH_2O). HR-ESI-MS: *found*: 504.0730 ([M – H]$^-$, calcd for $C_{22}H_{20}O_7N_3ClP^-$: 504.0733).

*HPLC column (Kinetex EVO, C18 100 Å) is mainly used for purification of mono- or triphosphates in 0.1 M triethylammonium bicarbonate buffer, the triethylammonium residue stays on the column, therefore residual peaks of triethylammonium salt are observed in NMR, giving **12b** as a mixture of free acid and triethylammonium salt form.

[(5-{[2-Chloro-4-(naphthalen-2-yl)-7*H*-pyrrolo[2,3-*d*]pyrimidin-7-yl]-*β*-D-ribofuranosyl}oxy)methyl]phosphonate triethylammonium salt (12g)

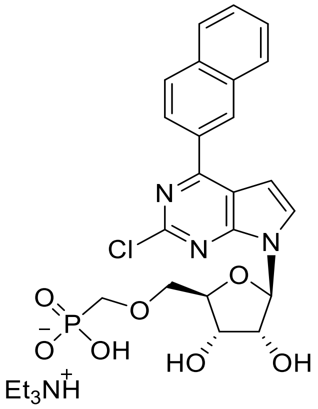

Compound **12g** was prepared according to the general procedure C from the corresponding crude starting material **11g** (100.4 mg, 0.16 mmol) in dry MeCN (7 mL). RP-HPFC (C-18, H_2O/MeOH 0 →100 %), HPLC (C-18, H_2O + 0.05% TFA/MeCN 0 → 80 %) and lyophilization from H_2O gave product **12g** (1:0.26 of triethylammonium salt *) as a pale-yellow powder (6.6 mg, 2 % over 2 steps). ^{1}H NMR (500 MHz, DMSO-d$_6$): 3.58 (d, 2H, $J_{CH2,P}$ = 9.1 Hz, OCH$_2$P); 3.62 – 3.77 (m, 2H, H-5´); 4.07 (m, 1H, H-4´); 4.18 (m, 1H, H-3´); 4.61 (bt, 1H, $J_{2',3'}$ = $J_{2',1'}$ = 5.7 Hz, H-2´); 6.24 (d, 1H, $J_{1',2'}$ = 6.6 Hz, H-1´); 7.16 (d, 1H, $J_{5,6}$ = 3.8 Hz, H-5); 7.56 – 7.66 (m; 2H, H-6,7-naphthyl); 8.00 (bd, 1H, $J_{5,6}$ = 7.9 Hz, H-5-naphthyl); 8.09 (bd, 1H, $J_{4,3}$ = 8.6 Hz, H-4-naphthyl); 8.15 – 8.25 (m, 3H, H-6, H-3,8-naphthyl); 8.71 (bs, 1H, H-1-naphthyl); ^{13}C NMR (125.7 MHz, DMSO-d$_6$): 67.92 (d, $J_{C,P}$ = 160.9 Hz, OCH$_2$P); 71.22 (CH-3´); 72.74 (d, $J_{C,P}$ = 12.0 Hz, CH$_2$-5´); 73.93 (CH-2´); 83.91 (CH-4´); 86.23 (CH-1´); 101.98 (CH-5); 114.64 (C-4a); 125.32 (CH-3-naphthyl); 126.78 (CH-7-naphthyl); 127.61 (CH-5-naphthyl); 127.80 (CH-6-naphthyl); 128.60 (CH-4-naphthyl); 129.14 and 129.22 (CH-1,8-naphthyl); 129.47 (CH-6); 132.74 (C-8a-naphthyl); 133.57 (C-2-naphthyl); 133.93 (C-4a-naphthyl); 152.36 (C-2); 153.75 (C-7a); 157.78 (C-4); ^{31}P NMR (202.4 MHz, DMSO-d$_6$): 16.58 (bs, 1P, OCH$_2$P). HR-ESI-MS: *found*: 504.0724 ([M − H]$^-$, calcd for $C_{22}H_{20}O_7N_3ClP^-$: 504.0722).

*HPLC column (Kinetex EVO, C18 100 Å) is mainly used for purification of mono- or triphosphates in 0.1 M triethylammonium bicarbonate buffer, the triethylammonium residue stays on the column, therefore residual peaks of triethylammonium salt are observed in NMR, giving 12b as a mixture of free acid and triethylammonium salt form.

Table S4. HPLC and UPLC purity of compounds 1, 7a-j and 12b,d,f,g.

Compound	Method	t_r [min]	Purity [%]	Compound	Method	t_r [min]	Purity [%]
**1**	D	10.29	97.50	**7h**	A	32.09	96.24
					B	25.81	95.81

**7a**	A	26.73	96.01	**7i**	A	31.62	98.51
	B	22.98	96.94		B	25.66	98.99
**7b**	A	25.92	97.36	**7j**	A	31.15	95.27
	B	22.35	98.28		B	25.23	98.69
**7c**	A	25.96	95.30	**12b**	C	6.99	99.58
	B	22.46	96.12				
**7d**	A	29.95	97.21	**12d**	C	8.22	95.60
	B	24.44	98.31				
**7e**	A	27.33	97.99	**12f**	C	9.53	100
	B	23.23	98.98				
**7f**	A	29.30	98.01	**12g**	C	8.72	99.14
	B	24.37	98.88				
**7g**	A	30.37	95.44				
	B	24.88	99.40				

Methods: **A:** 5% MeOH in 0.1 M TEAB to 100% MeOH in 40 min, **B:** 5% MeCN 0.1 M TEAB to 100% MeOH in 40 min, **C**: 1 % H_2O + 12 mM TEA to 100% (80 % ACN + 12 mM TEA) in 13 min, **D**: 2% MeCN in H_2O + 0.1% TFA to 100% MeCN in 30 min.

Table S5: Yields of compounds 3-7, 10, 12.

Entry		R	3	4	5	6	7	10	12
**1**	**a**	thiophen-3-yl	95[a]	86[b]	26	21	20		
**2**	**b**	furan-2-yl	80[a]	51[b]	32	16	46	76[a]	3[c]
**3**	**c**	furan-3-yl	96[a]	71[b]	20	23	53		
**4**	**d**	benzofuran-2-yl	-	88	nd	19	14[c]	43[a]	2[c]
**5**	**e**	phenyl	-	96	nd	16	9[c]		
**6**	**f**	naphth-1-yl	-	55	nd	25	23[c]	81[a]	5[c]
**7**	**g**	naphthalen-2-yl	-	nd	nd	11[c]	12[d]	72[a]	2[c]
**8**	**h**	1,1´-biphenyl-4-yl	-	70	47	30	80		
**9**	**i**	phenanthren-9-yl	-	94	46	10	17		
**10**	**j**	dibenzofuran-4-yl	-	83	37	20	80		

[a] yield of cross-coupling step; [b] yield of deprotection of nucleoside **7**; [c] yield over 2 steps; [d] yield over 3 steps; nd – yield not calculated due to insufficient purity of the intermediate

HPLC purity analysis of final nucleotides **1, 7a-j** (>95%) was carried out using a Waters assembly equipped with a model 600 Controller pump and a model 2996 Photodiode Array Detector. Measurements were performed on Phenomenex Gemini 5u C18 110A column of 250 x 4.60 mm, flow was 1mL/min, and UV detection was employed. Individual methods are written under the table with HPLC purity (Supplementary Table S3). The injector was a model 717 plus Waters Autosampler (10 µL). Linear gradients were used in all methods. Run time was 40 min. The acquisition and treatment of data were processed with the Empower Pro software.

UPLC-MS analysis of final nucleotides **12b**,**d**,**f**,**g** (>95%) was performed on an *Agilent 1260 Infinity II LC* system with an *Agilent 1260 Photodiode Array Detector*, Column: *Kinetex EVO C18 100 Å 1.7 µm* (2.1 × 150 mm), Flow: 0.2-0.25 mL/min.

NMR spectra of reported compounds

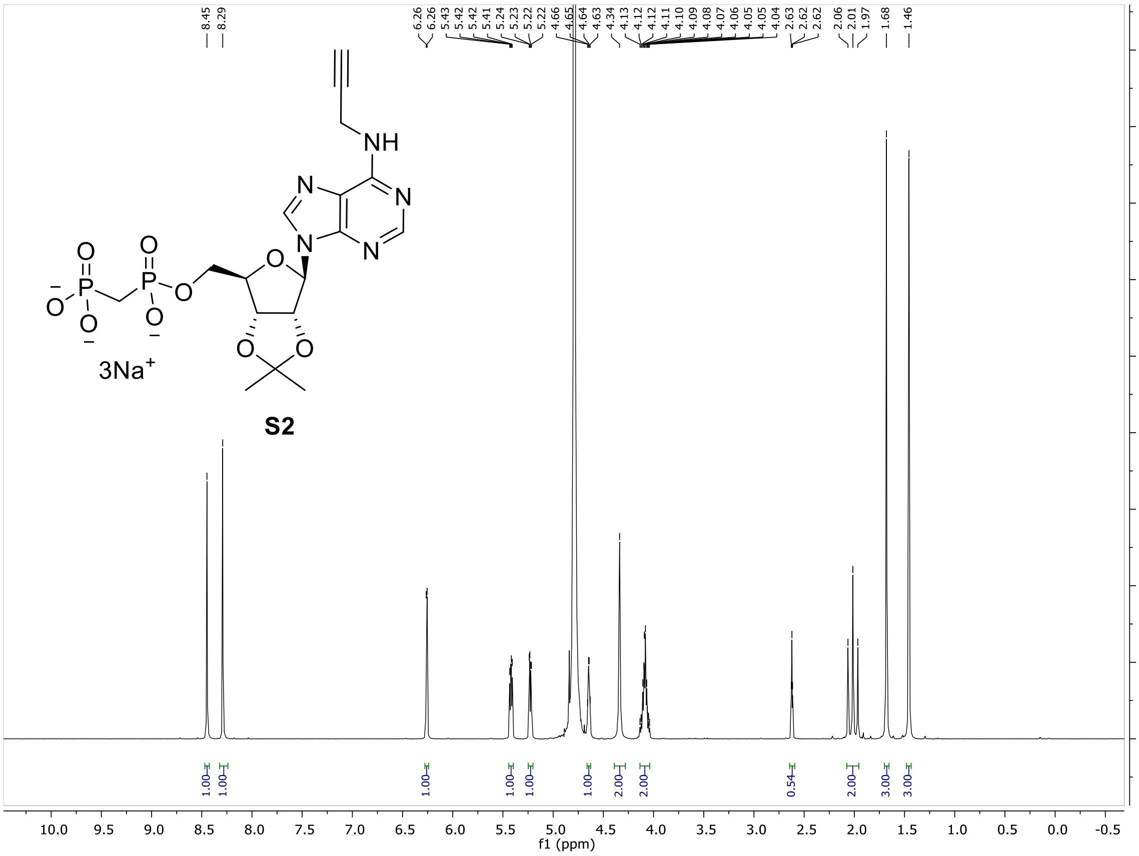

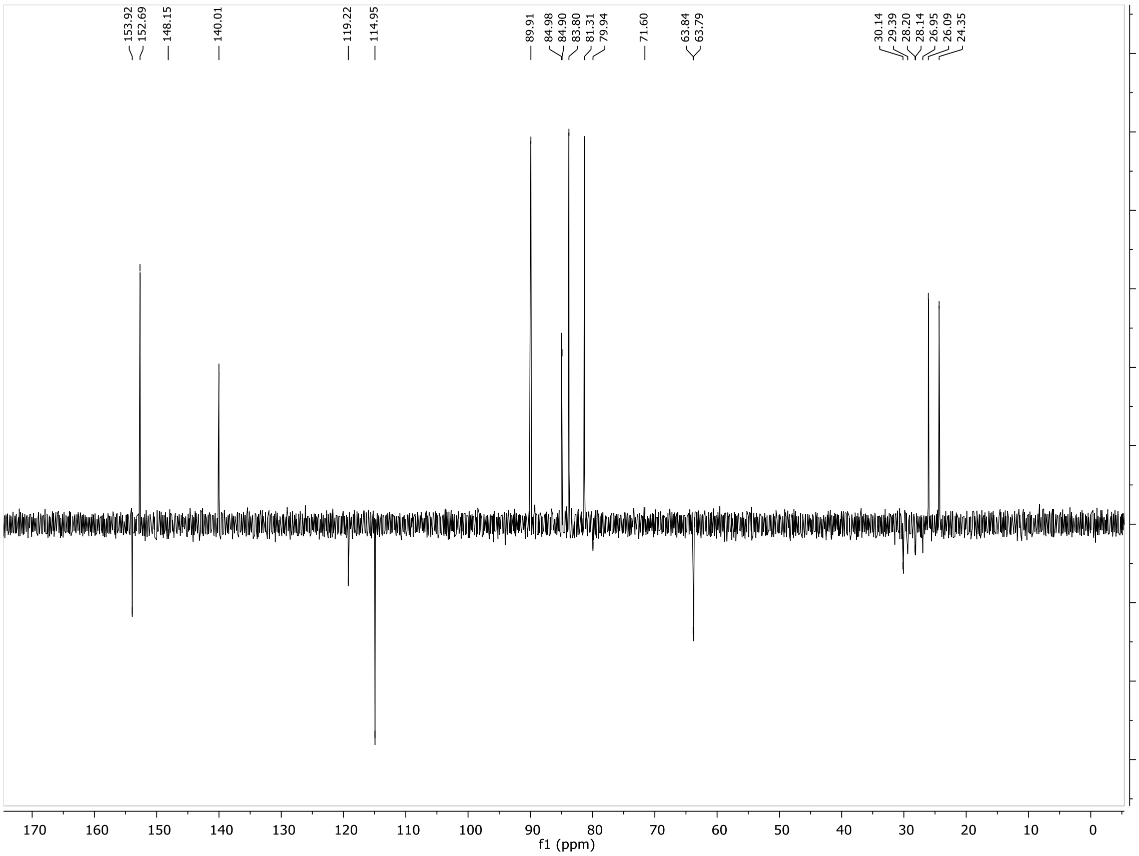

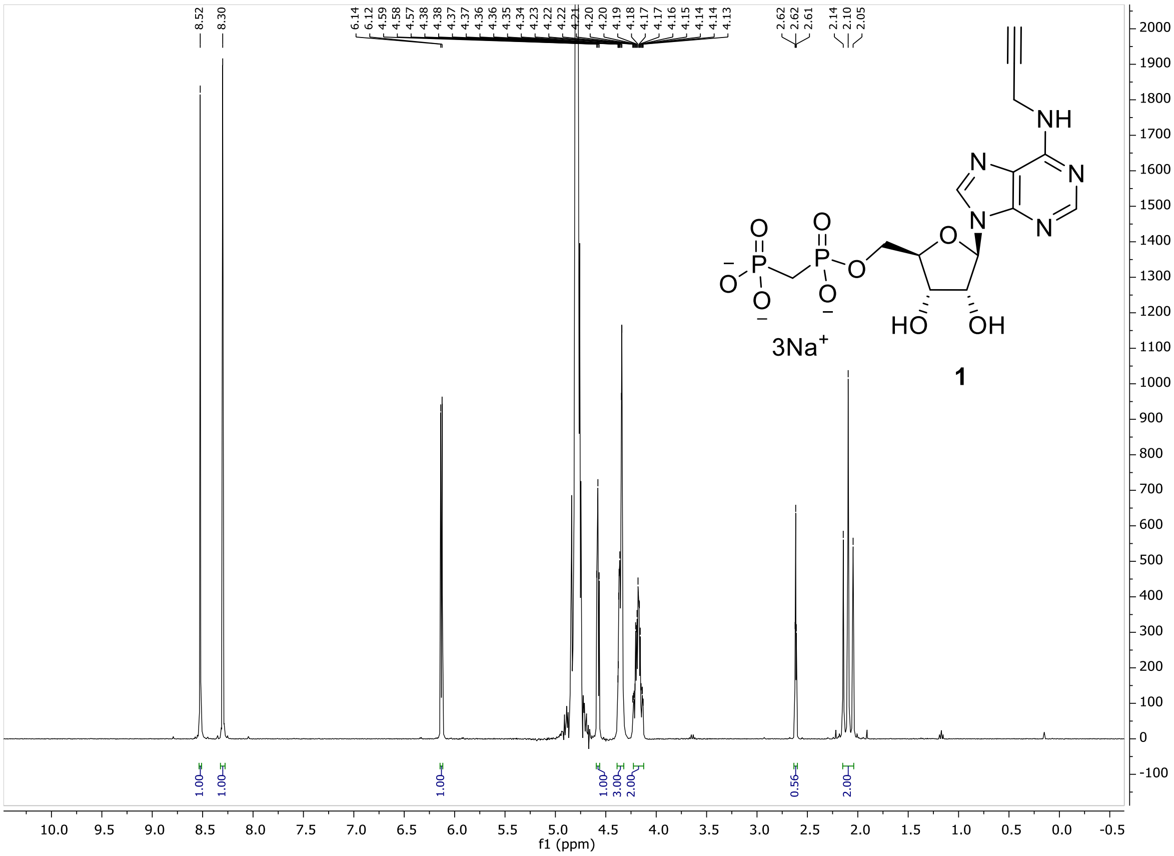
8.52
8.30
6.14
6.12
4.59
4.58
4.57
4.38
4.38
4.37
4.37
4.36
4.36
4.35
4.34
4.23
4.22
4.22
4.21
4.20
4.20
4.19
4.18
4.17
4.17
4.16
4.15
4.14
4.14
4.13
2.62
2.62
2.61
2.14
2.10
2.05
1.00
1.00
1.00
1.00
3.00
2.00
0.56
2.00
NH
N
N
N
N
O
P
HO
OH
3Na+
1
f1 (ppm)

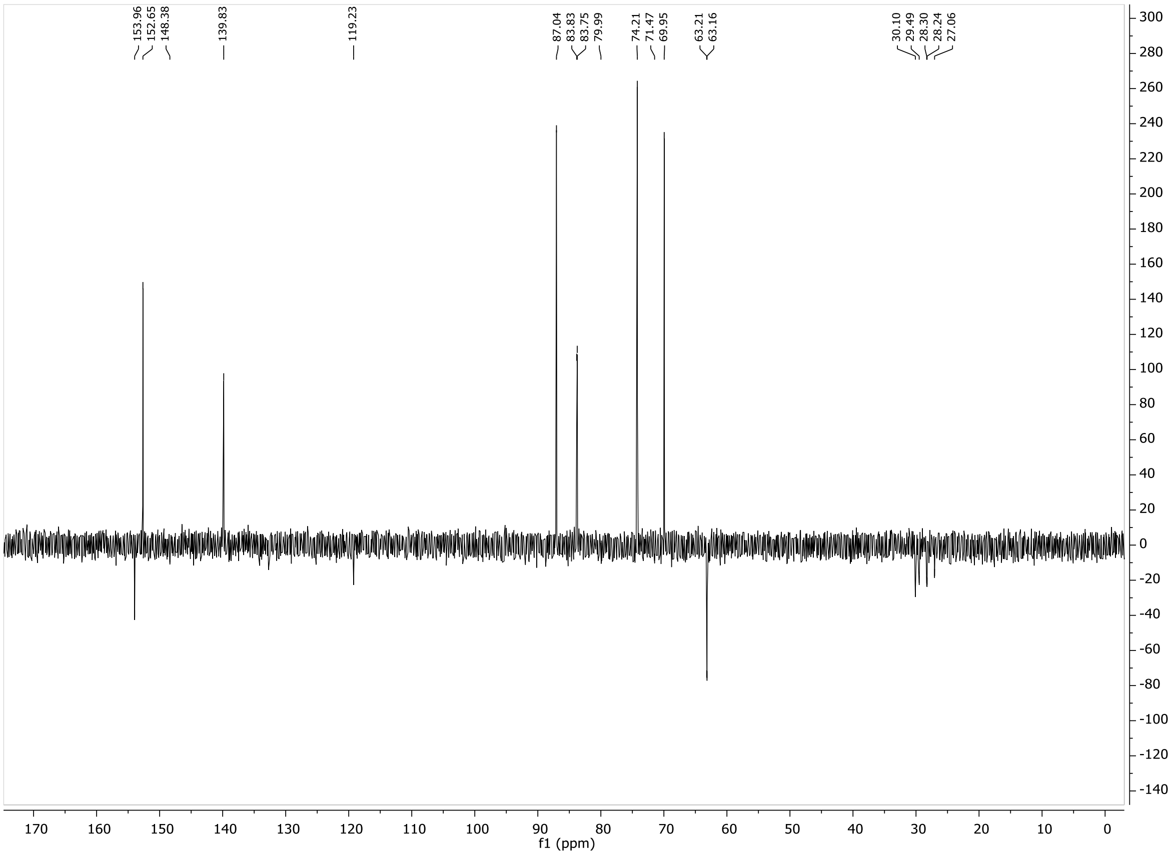
153.96
152.65
148.38
139.83
119.23
87.04
83.83
83.75
79.99
74.21
71.47
69.95
63.21
63.16
30.10
29.49
28.30
28.24
27.06
f1 (ppm)

VESELOVSKA LV631
1H NMR in CDCl3
16-10-20 RA
****

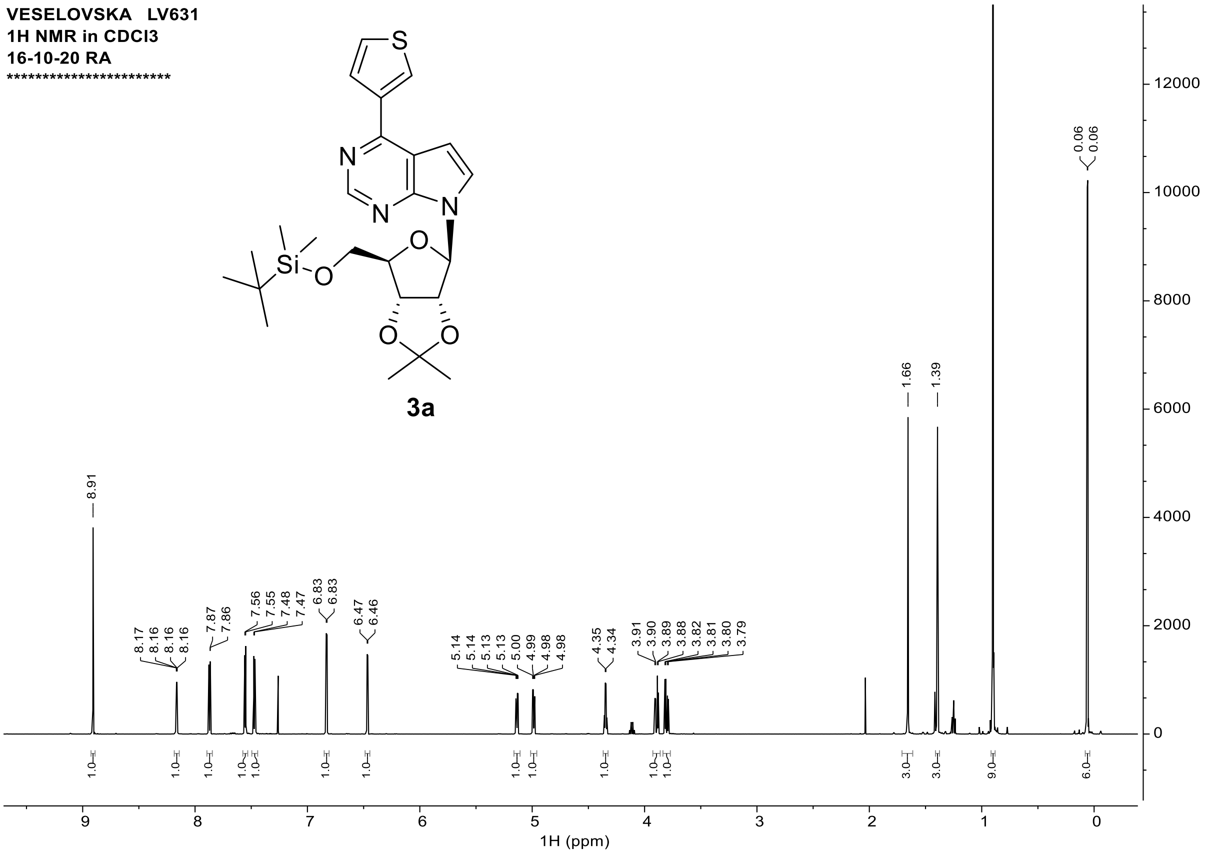

VESELOVSKA LV631
APT in CDCl3
16-10-20 RA
****

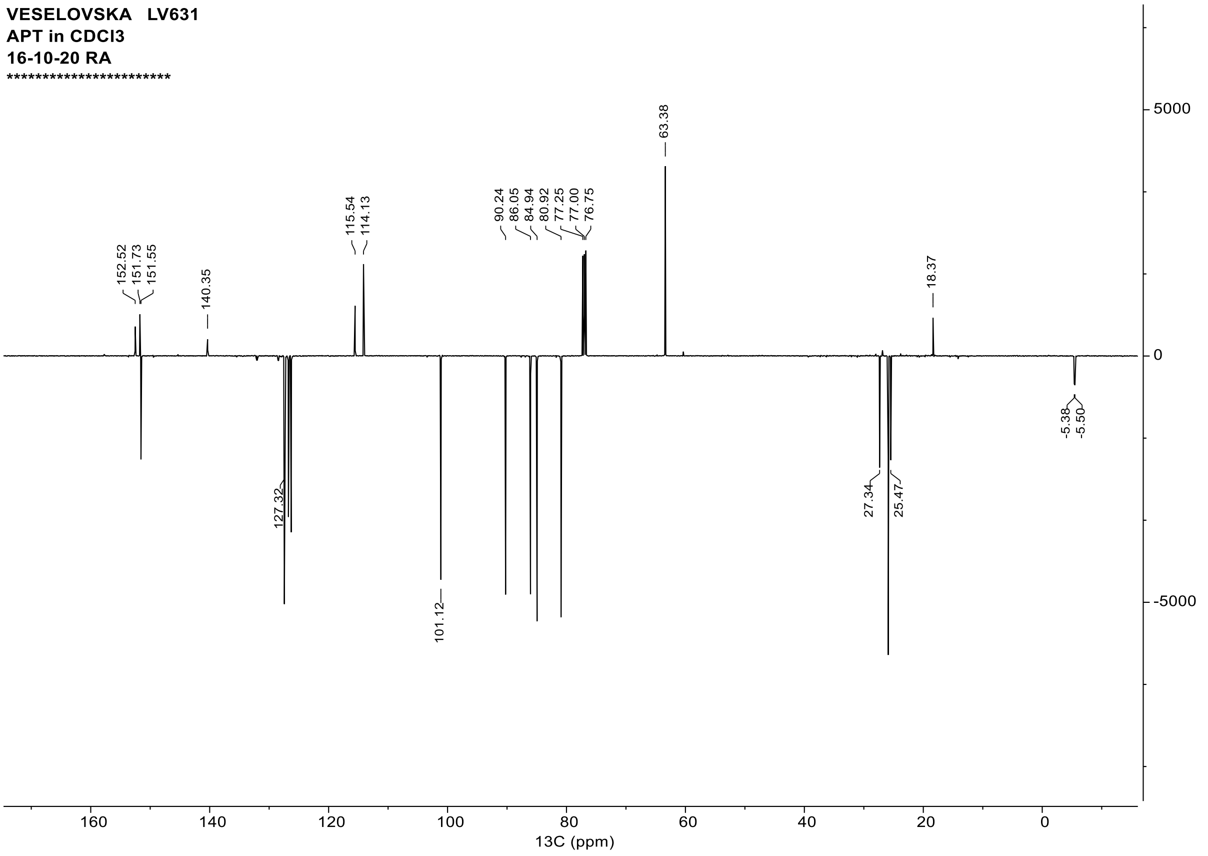

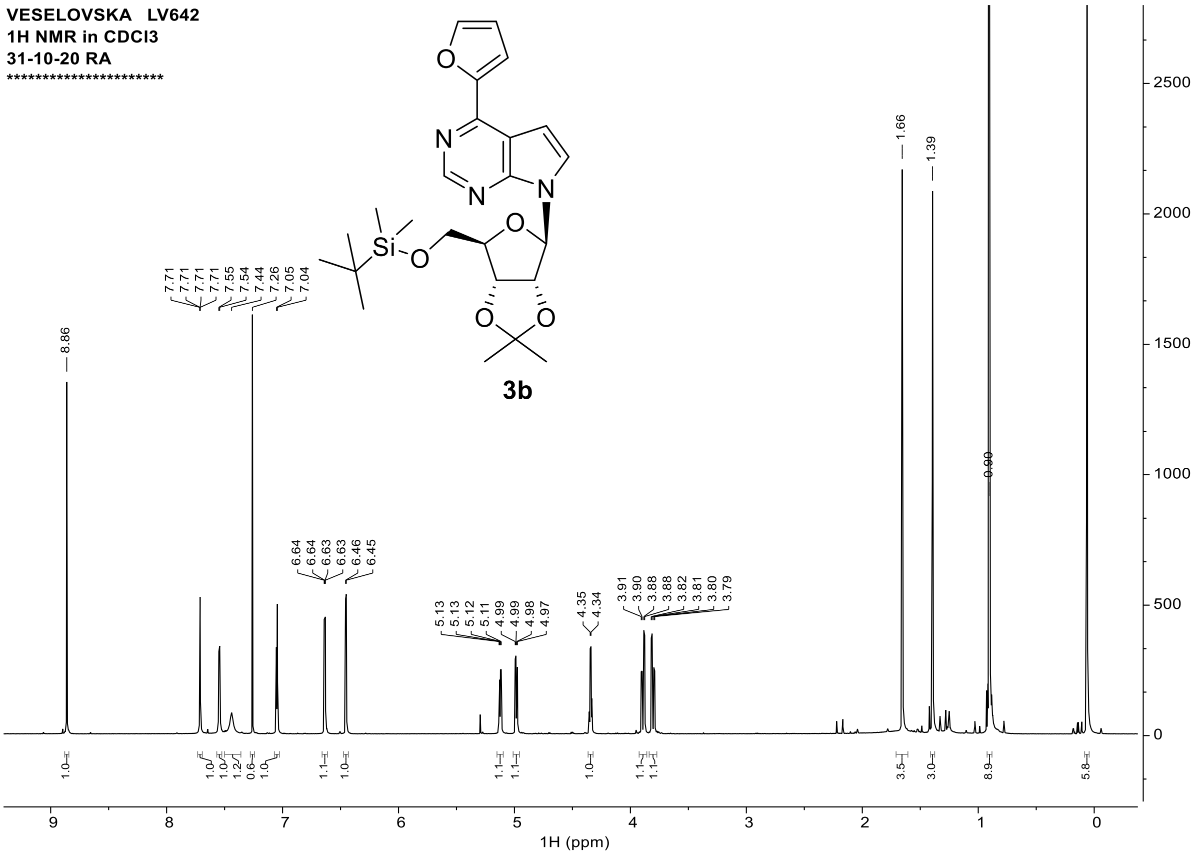
VESELOVSKA LV642
1H NMR in CDCl3
31-10-20 RA
****
3b
8.86
7.71 7.71 7.71 7.71 7.55 7.54 7.44 7.26 7.05 7.04
6.64 6.64 6.63 6.63 6.46 6.45
5.13 5.13 5.12 5.11 4.99 4.99 4.98 4.97
4.35 4.34
3.91 3.90 3.88 3.88 3.82 3.81 3.80 3.79
1.66
1.39
0.90
1.0 1.0 1.0 1.2 0.6 1.0 1.1 1.0 1.1 1.1 1.0 1.1 1.1 3.5 3.0 8.9 5.8
1H (ppm)

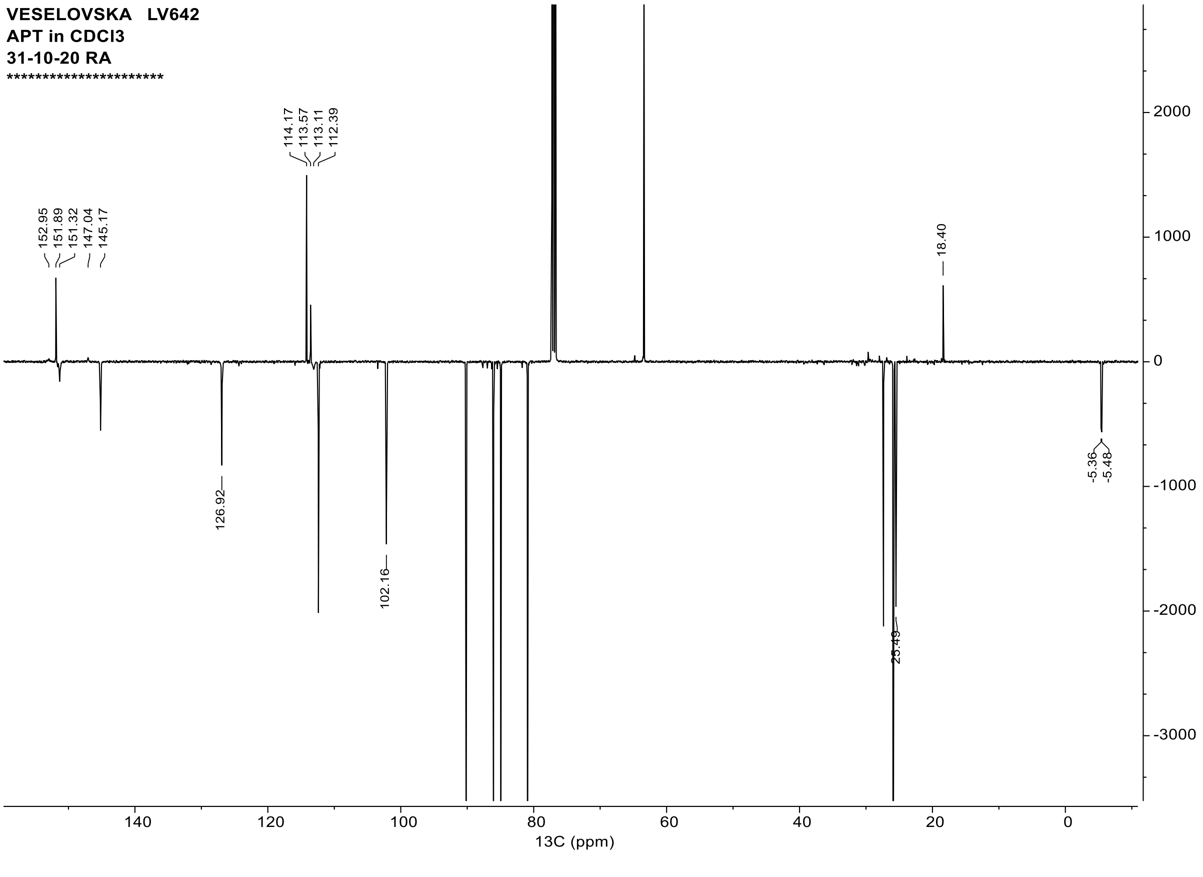
VESELOVSKA LV642
APT in CDCl3
31-10-20 RA
****
152.95 151.89 151.32 147.04 145.17
114.17 113.57 113.11 112.39
126.92
102.16
18.40
25.49
-5.36 -5.48
13C (ppm)

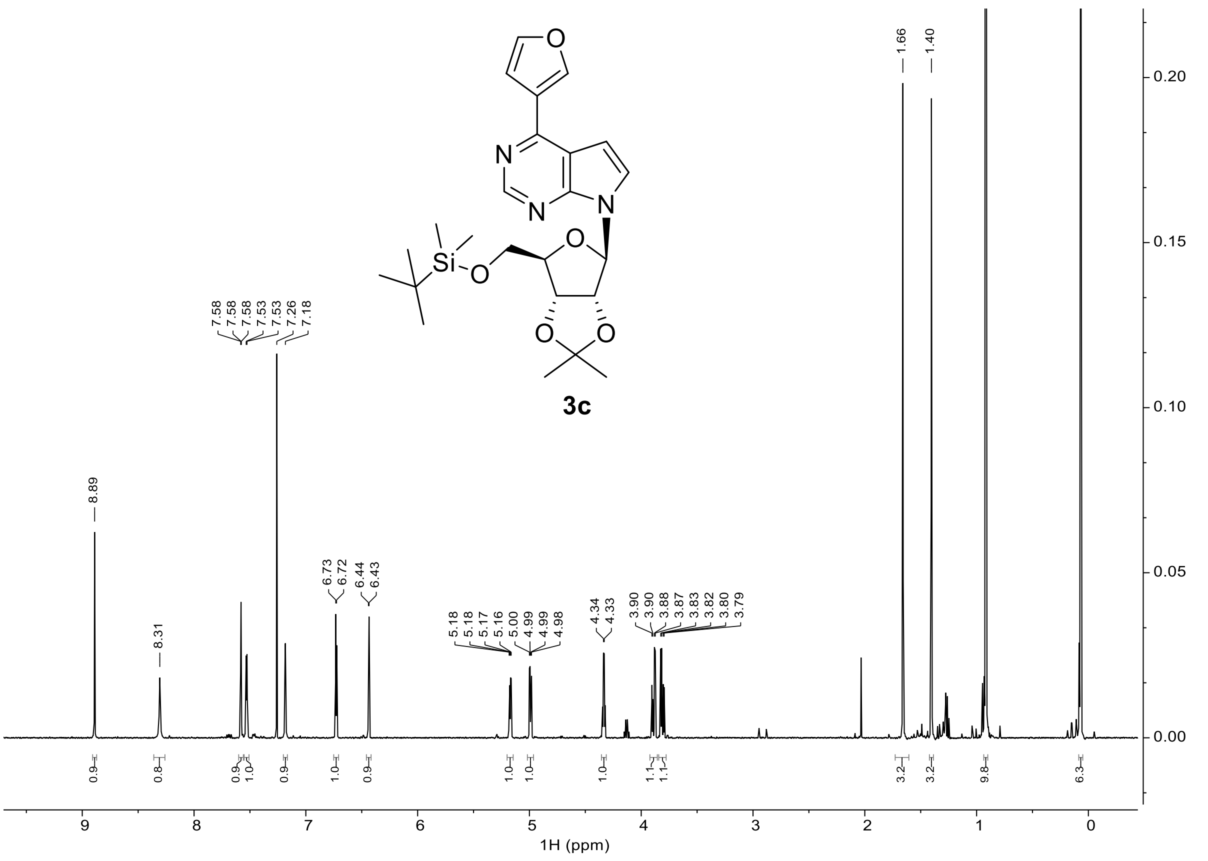
3c
8.89
8.31
7.58 7.58 7.58 7.53 7.53 7.26 7.18
6.73 6.72 6.44 6.43
5.18 5.18 5.17 5.16 5.00 4.99 4.99 4.98
4.34 4.33
3.90 3.90 3.88 3.87 3.83 3.82 3.80 3.79
1.66
1.40
1H (ppm)

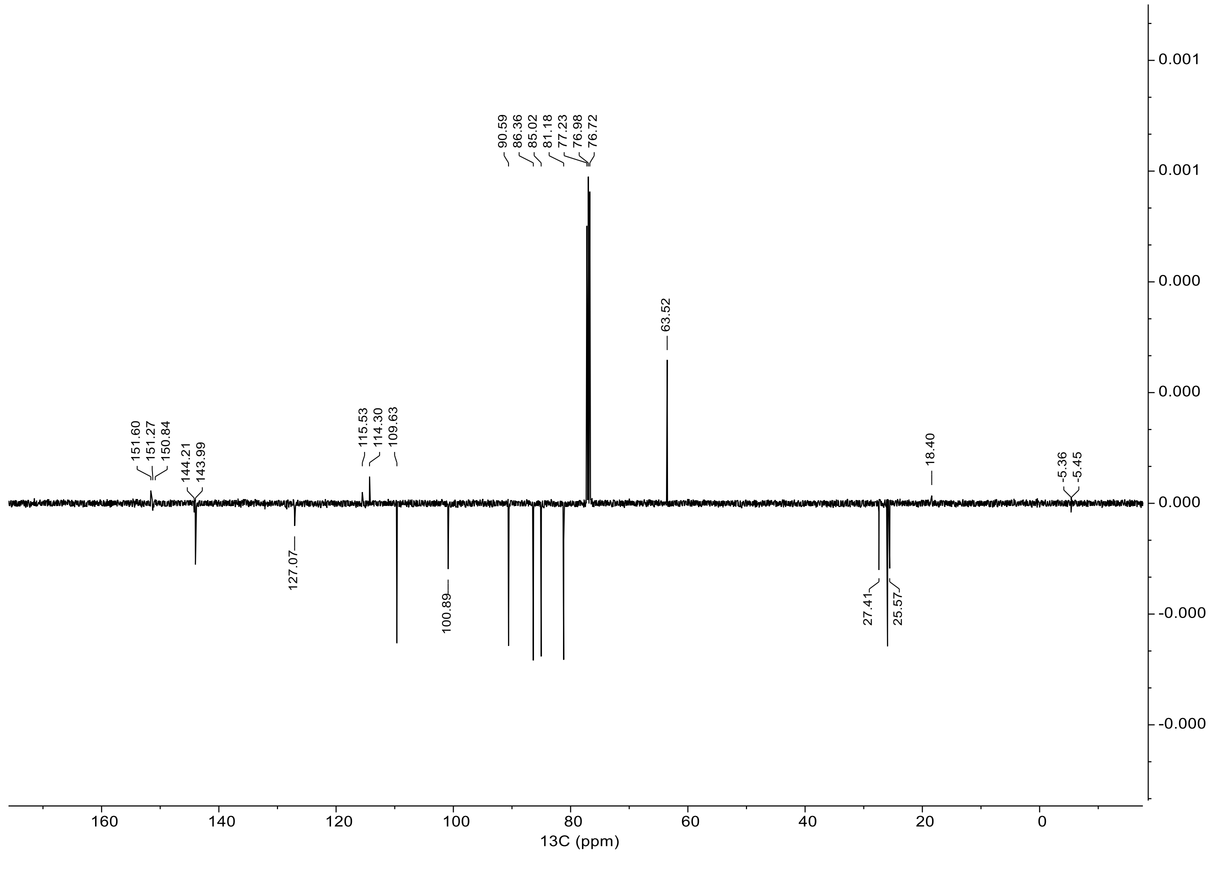
151.60 151.27 150.84 144.21 143.99
127.07
115.53 114.30 109.63
100.89
90.59 86.36 85.02 81.18 77.23 76.98 76.72
63.52
27.41 25.57
18.40
-5.36 -5.45
13C (ppm)

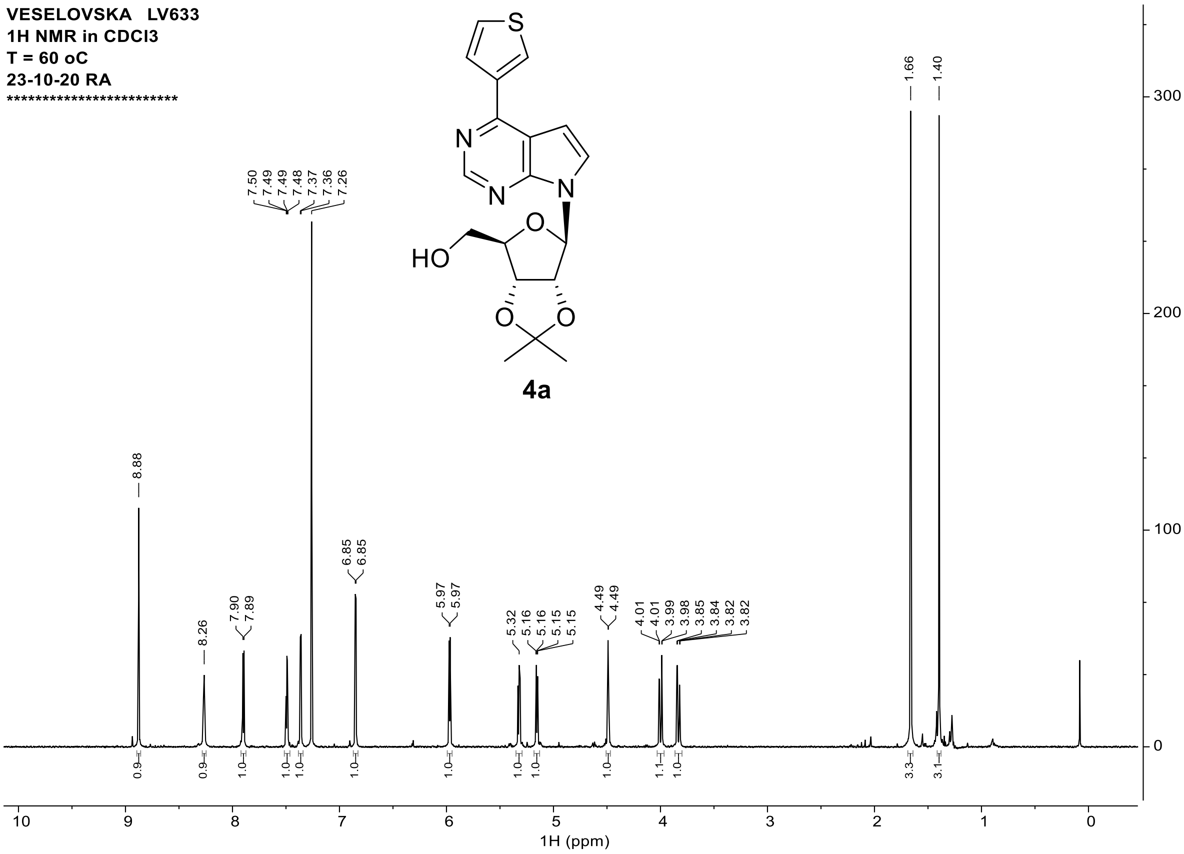

VESELOVSKA LV633
1H NMR in CDCl3
T = 60 oC
23-10-20 RA
****
4a
8.88
8.26
7.90 7.89
7.50 7.49 7.49 7.48 7.37 7.36 7.26
6.85 6.85
5.97 5.97
5.32 5.16 5.16 5.15 5.15
4.49 4.49
4.01 4.01 3.99 3.98 3.85 3.84 3.82 3.82
1.66
1.40
1H (ppm)

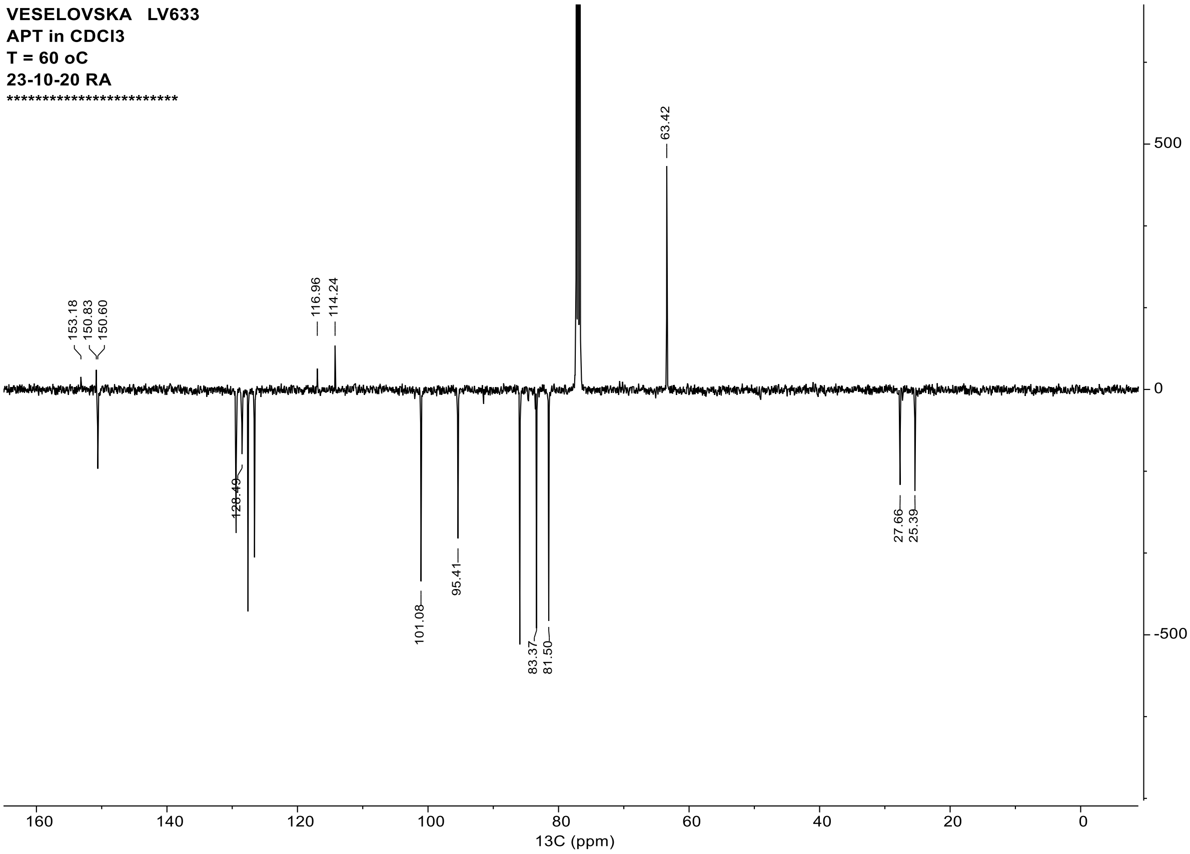

VESELOVSKA LV633
APT in CDCl3
T = 60 oC
23-10-20 RA
****
153.18 150.83 150.60
116.96 114.24
128.49
101.08
95.41
83.37 81.50
63.42
27.66 25.39
13C (ppm)

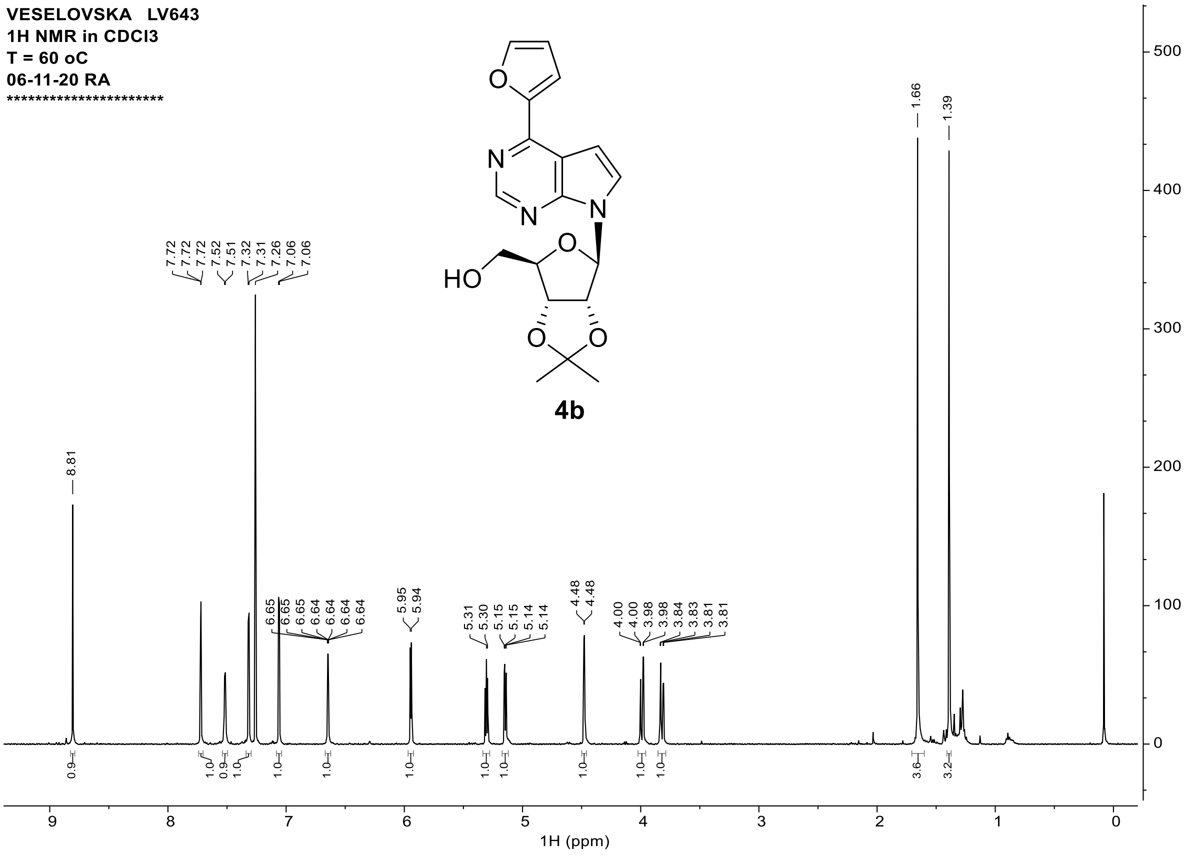
VESELOVSKA LV643
1H NMR in CDCl3
T = 60 oC
06-11-20 RA
****
4b
HO
8.81
7.72 7.72 7.72 7.52 7.51 7.32 7.31 7.26 7.06 7.06
6.65 6.65 6.64 6.64 6.64 6.64
5.95 5.94
5.31 5.30 5.15 5.15 5.14 5.14
4.48 4.48
4.00 4.00 3.98 3.98 3.84 3.83 3.81 3.81
1.66
1.39
0.9 1.0 0.9 1.0 1.0 1.0 1.0 1.0 1.0 1.0 1.0 1.0 3.6 3.2
1H (ppm)

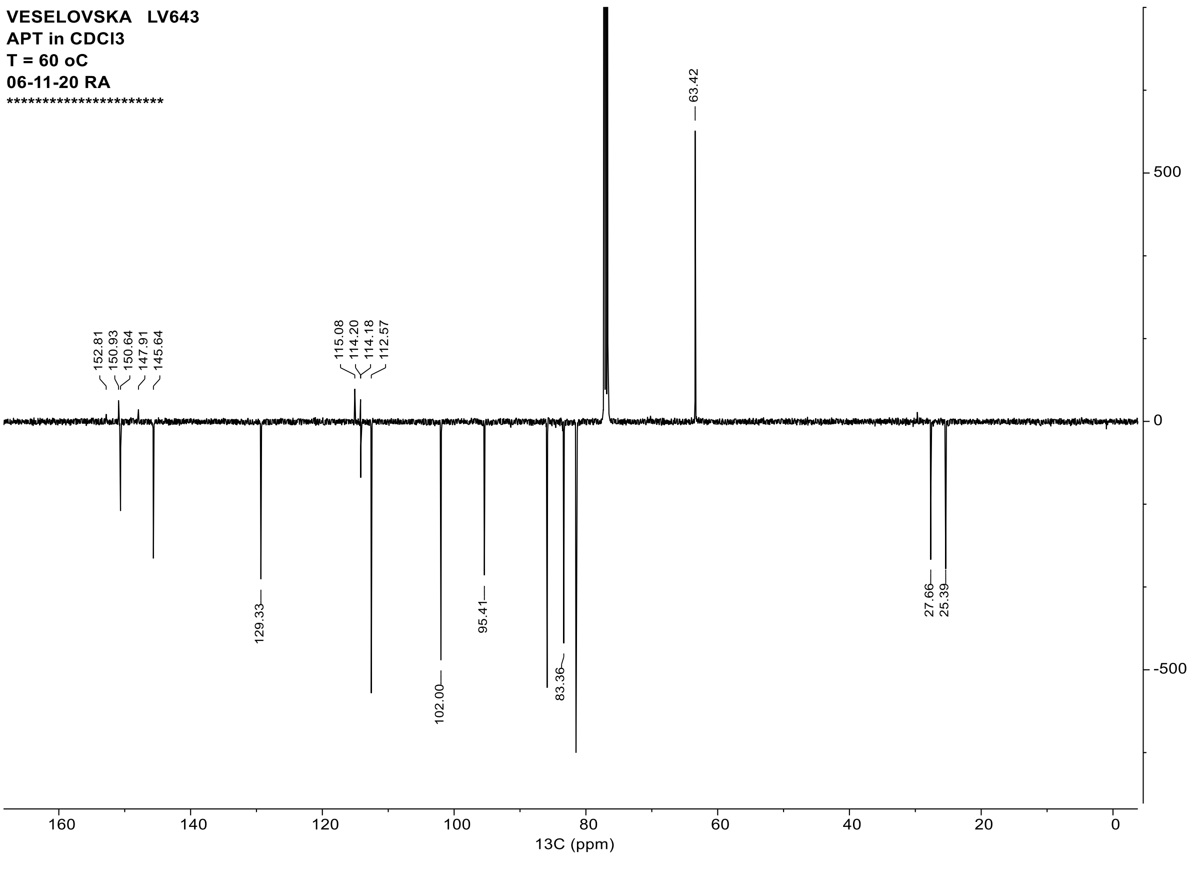
VESELOVSKA LV643
APT in CDCl3
T = 60 oC
06-11-20 RA
****
152.81 150.93 150.64 147.91 145.64
115.08 114.20 114.18 112.57
63.42
129.33
102.00
95.41
83.36
27.66 25.39
13C (ppm)

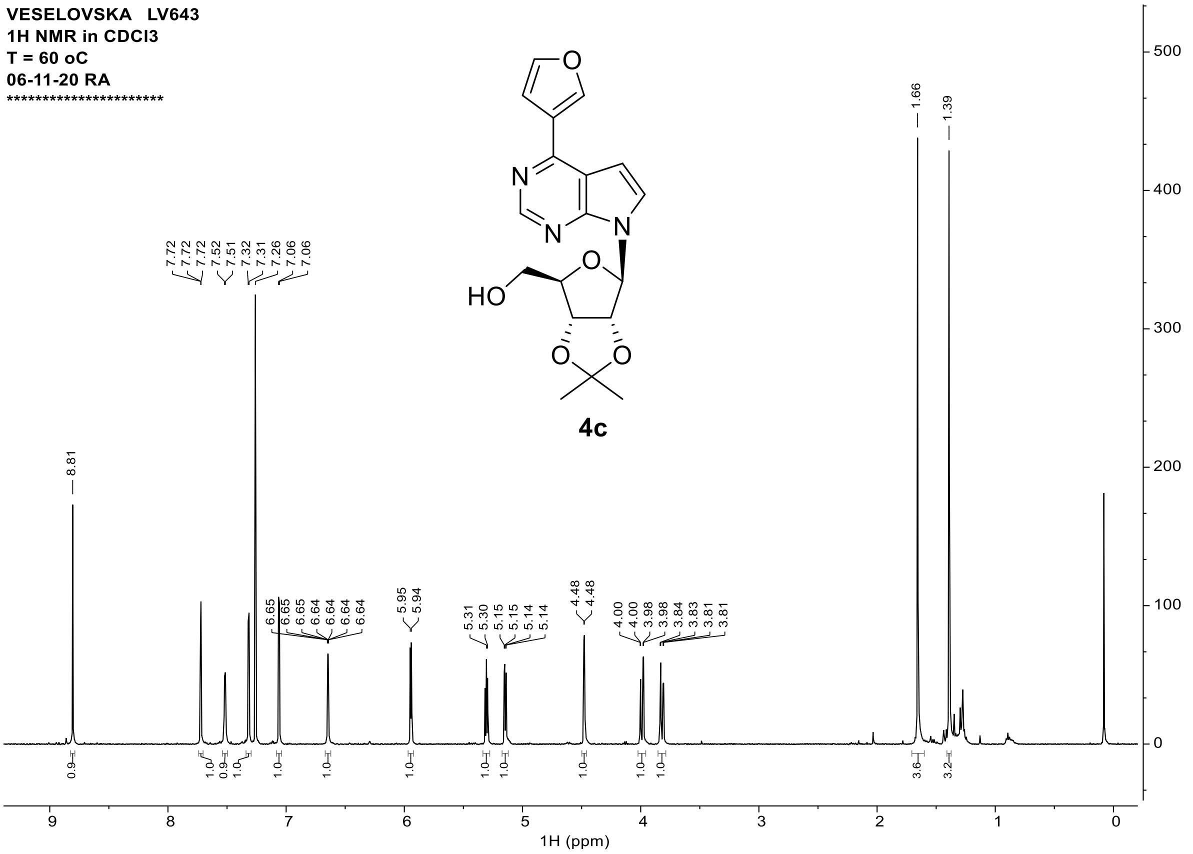
VESELOVSKA LV643
1H NMR in CDCl3
T = 60 oC
06-11-20 RA
****
4c
HO
N
O
8.81
7.72
7.72
7.72
7.52
7.51
7.32
7.31
7.26
7.06
7.06
6.65
6.65
6.64
6.64
6.64
6.64
5.95
5.94
5.31
5.30
5.15
5.15
5.14
5.14
4.48
4.48
4.00
4.00
3.98
3.98
3.84
3.83
3.81
3.81
1.66
1.39
0.9
1.0
0.9
1.0
1.0
1.0
1.0
1.0
1.0
1.0
1.0
1.0
1.0
3.6
3.2
1H (ppm)

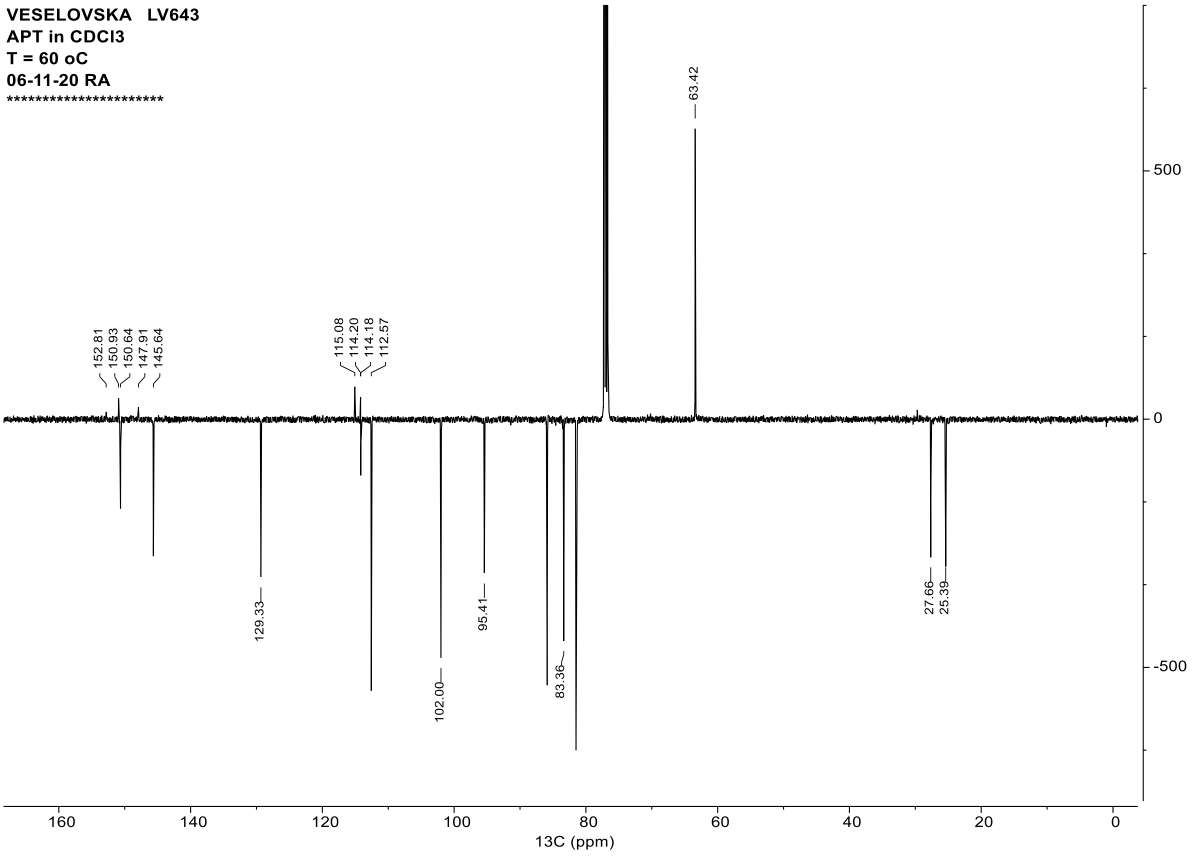
VESELOVSKA LV643
APT in CDCl3
T = 60 oC
06-11-20 RA
****
152.81
150.93
150.64
147.91
145.64
115.08
114.20
114.18
112.57
63.42
129.33
102.00
95.41
83.36
27.66
25.39
13C (ppm)

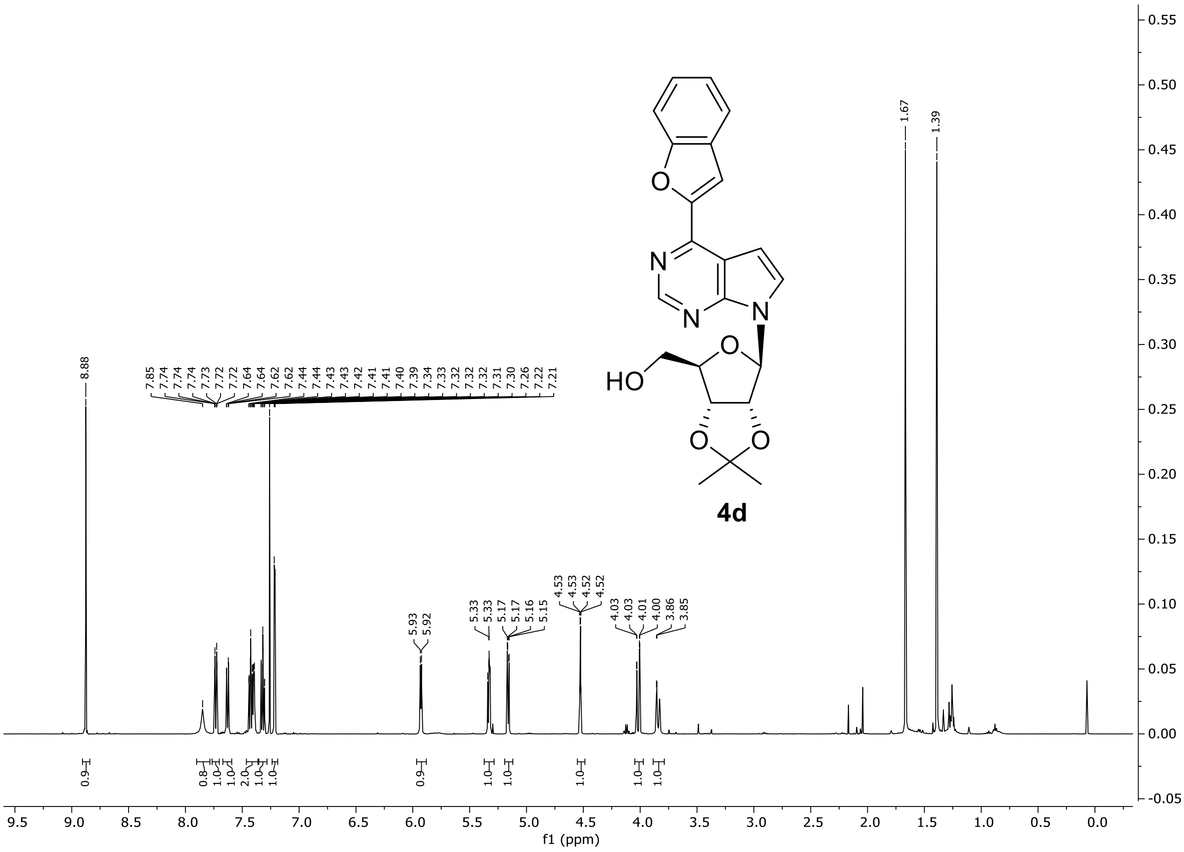
8.88
7.85 7.74 7.74 7.74 7.73 7.72 7.72 7.64 7.64 7.62 7.62 7.44 7.44 7.43 7.43 7.42 7.41 7.40 7.39 7.34 7.33 7.32 7.32 7.32 7.31 7.30 7.26 7.22 7.21
5.93 5.92
5.33 5.33 5.17 5.17 5.16 5.15
4.53 4.53 4.52 4.52
4.03 4.03 4.01 4.00 3.86 3.85
1.67
1.39
0.9 0.8 1.0 1.0 2.0 1.0 1.0 0.9 1.0 1.0 1.0 1.0 1.0
HO
N
O
4d
f1 (ppm)

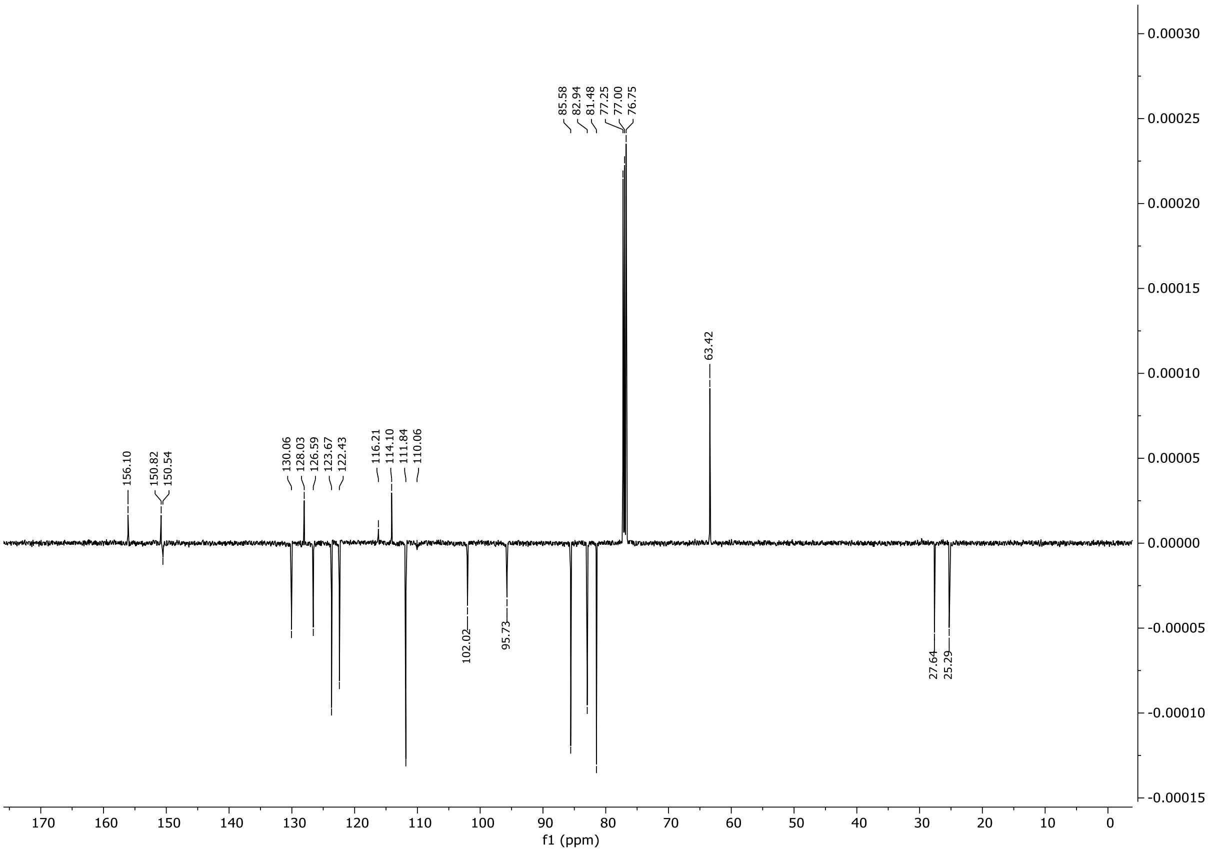
156.10
150.82 150.54
130.06 128.03 126.59 123.67 122.43
116.21 114.10 111.84 110.06
102.02
95.73
85.58 82.94 81.48 77.25 77.00 76.75
63.42
27.64 25.29
f1 (ppm)

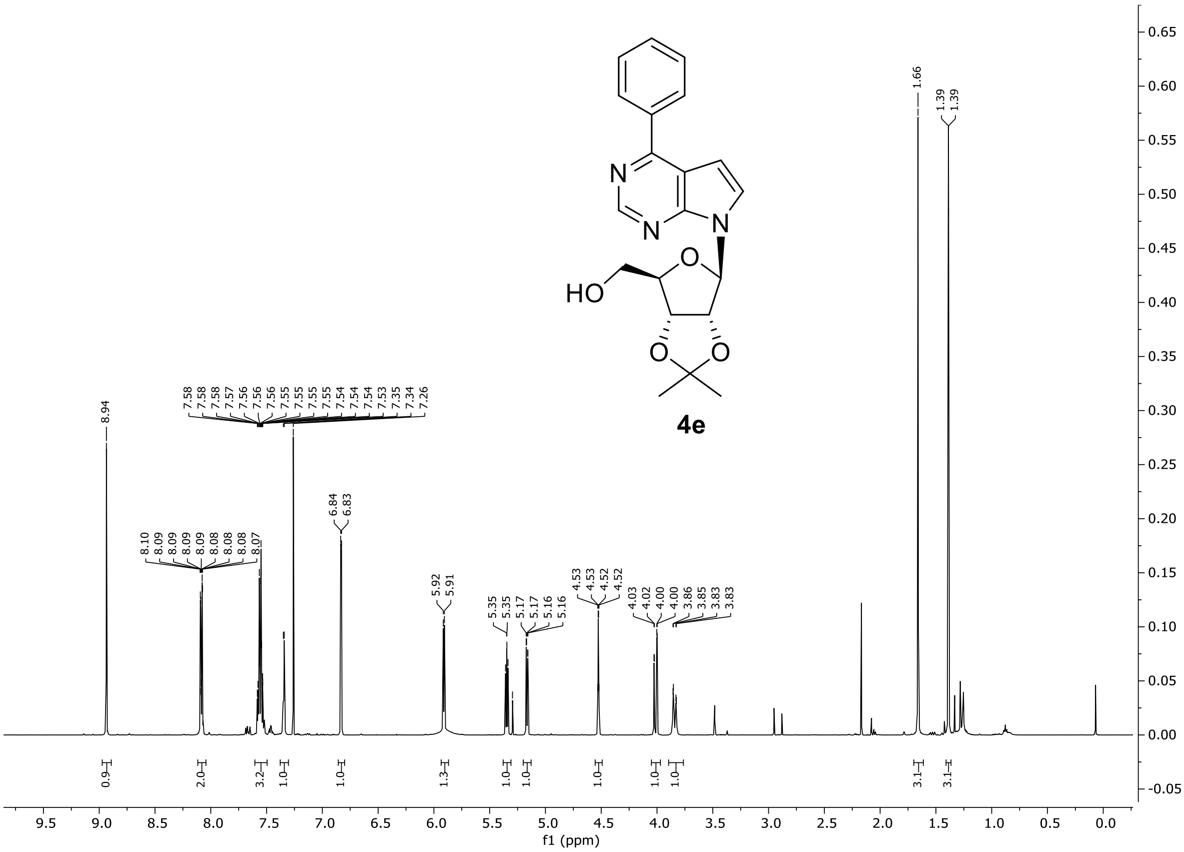
4e
f1 (ppm)

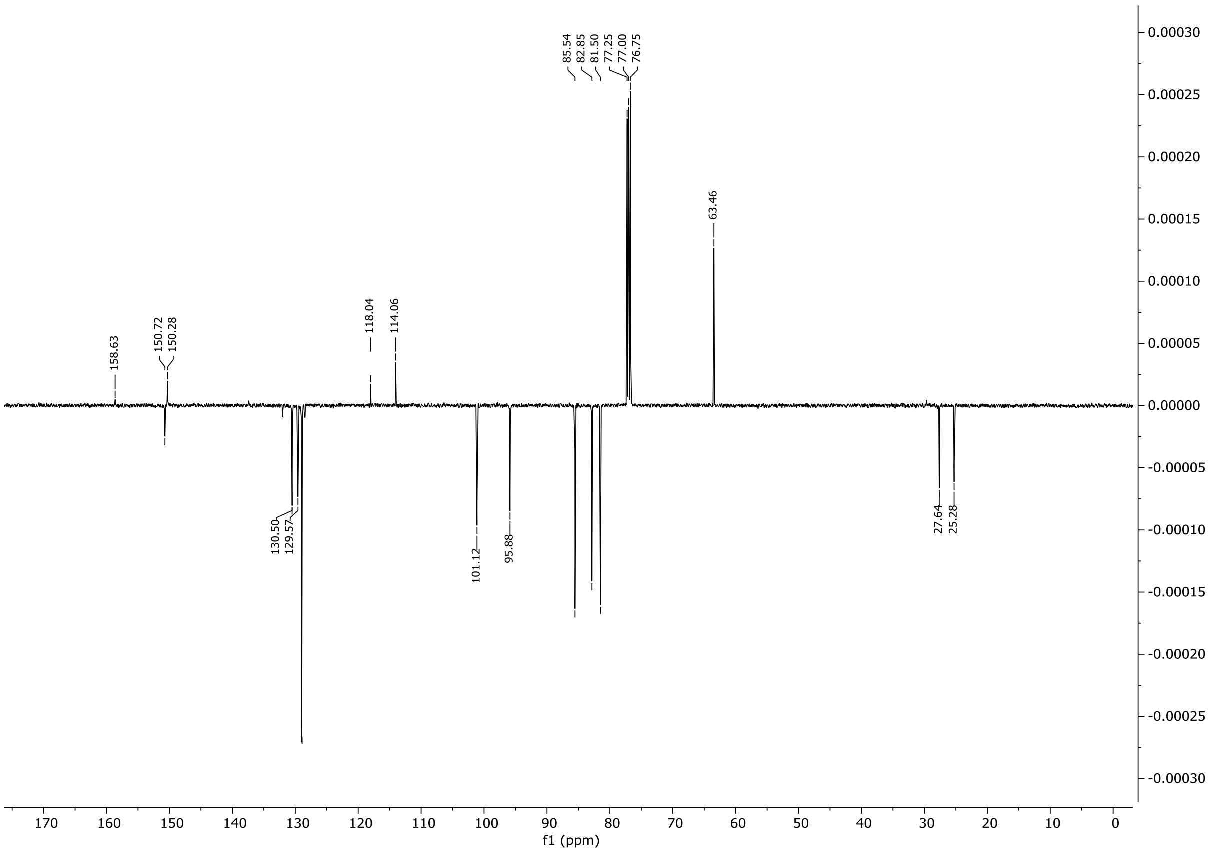
f1 (ppm)

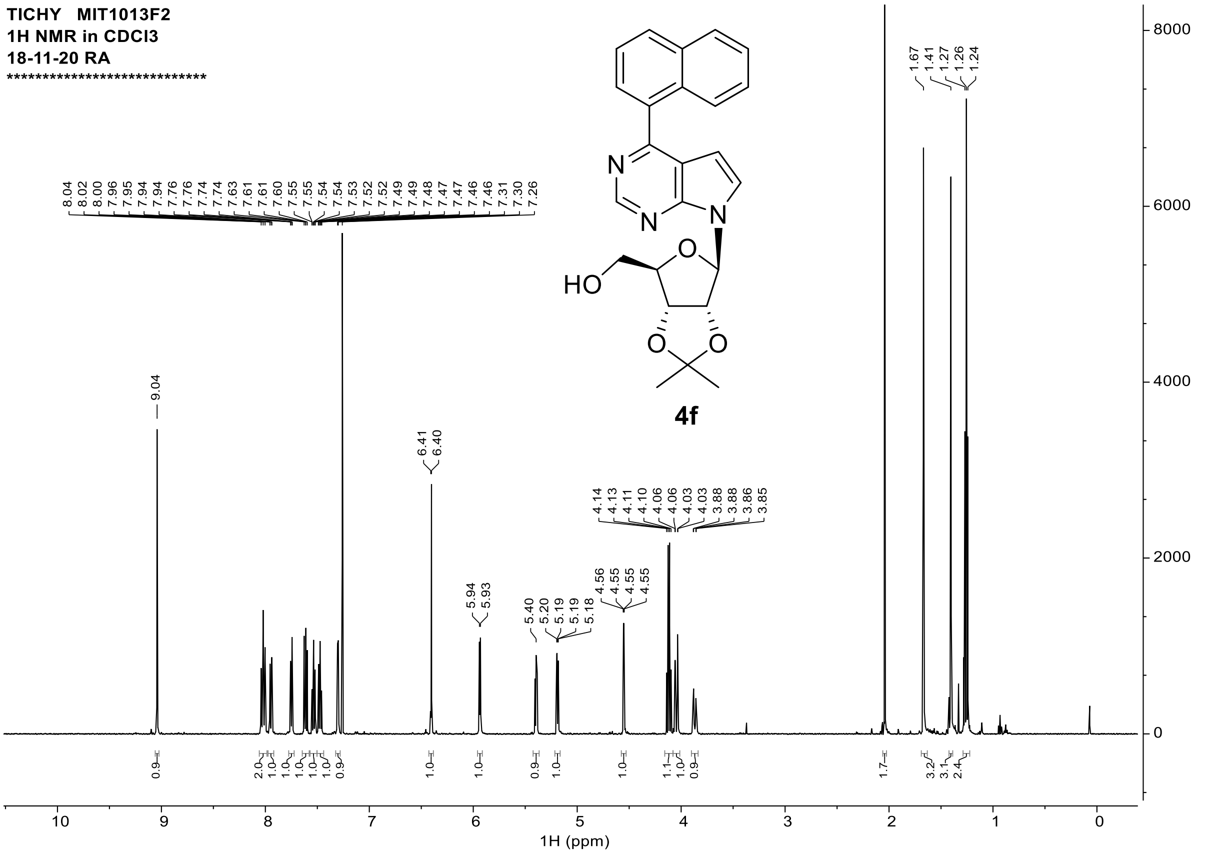
TICHY MIT1013F2
1H NMR in CDCl3
18-11-20 RA
****
4f
HO
N
N
N
O
O
O
9.04
8.04 8.02 8.00 7.96 7.95 7.94 7.94 7.76 7.76 7.74 7.74 7.63 7.61 7.61 7.60 7.55 7.55 7.54 7.54 7.53 7.52 7.52 7.49 7.49 7.48 7.47 7.47 7.46 7.46 7.31 7.30 7.26
6.41 6.40
5.94 5.93
5.40 5.20 5.19 5.19 5.18
4.56 4.55 4.55 4.55
4.14 4.13 4.11 4.10 4.06 4.06 4.03 4.03 3.88 3.88 3.86 3.85
1.67 1.41 1.27 1.26 1.24
0.9 2.0 1.0 1.0 1.0 1.0 1.0 0.9 1.0 1.0 0.9 1.0 1.0 1.1 1.0 0.9 1.7 3.2 3.1 2.4
8000
6000
4000
2000
0
10 9 8 7 6 5 4 3 2 1 0
1H (ppm)

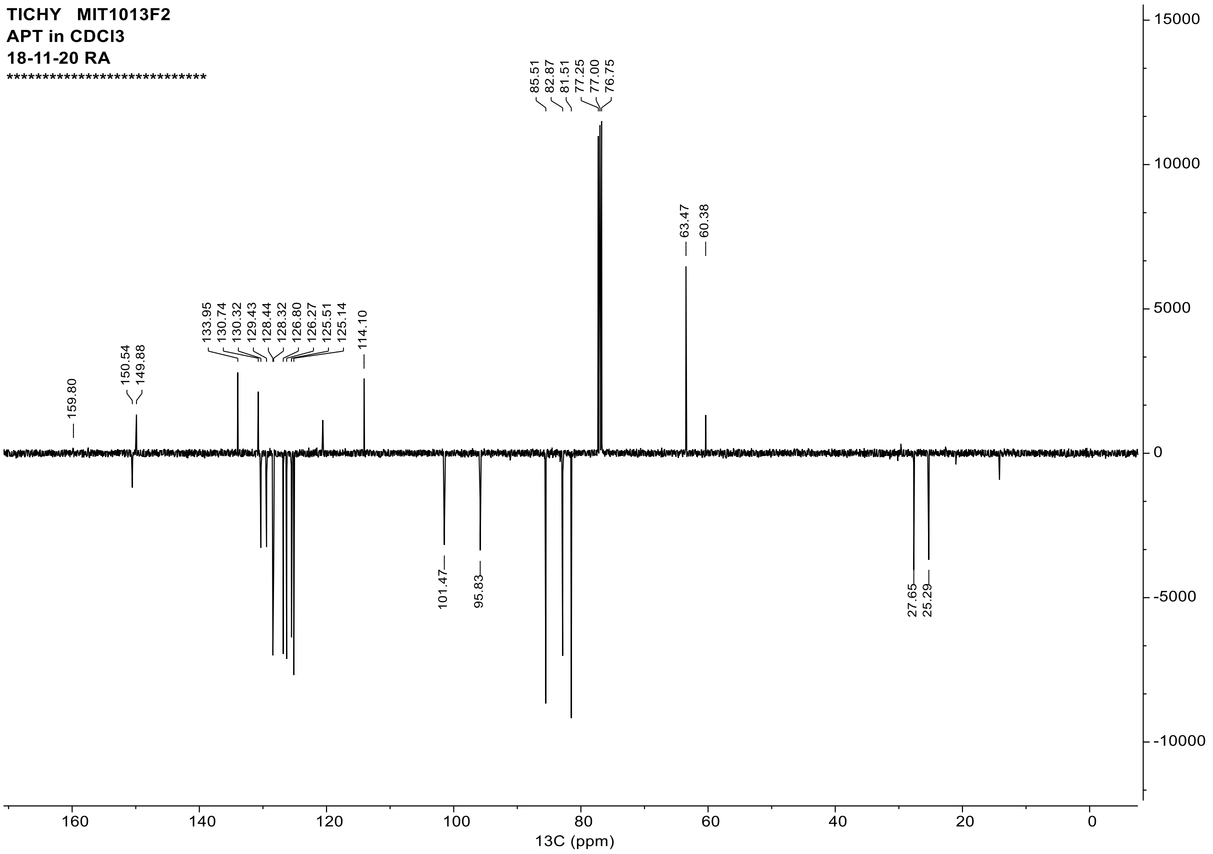
TICHY MIT1013F2
APT in CDCl3
18-11-20 RA
85.51 82.87 81.51 77.25 77.00 76.75
63.47 60.38
133.95 130.74 130.32 129.43 128.44 128.32 126.80 126.27 125.51 125.14
114.10
150.54 149.88
159.80
101.47 95.83
27.65 25.29
13C (ppm)

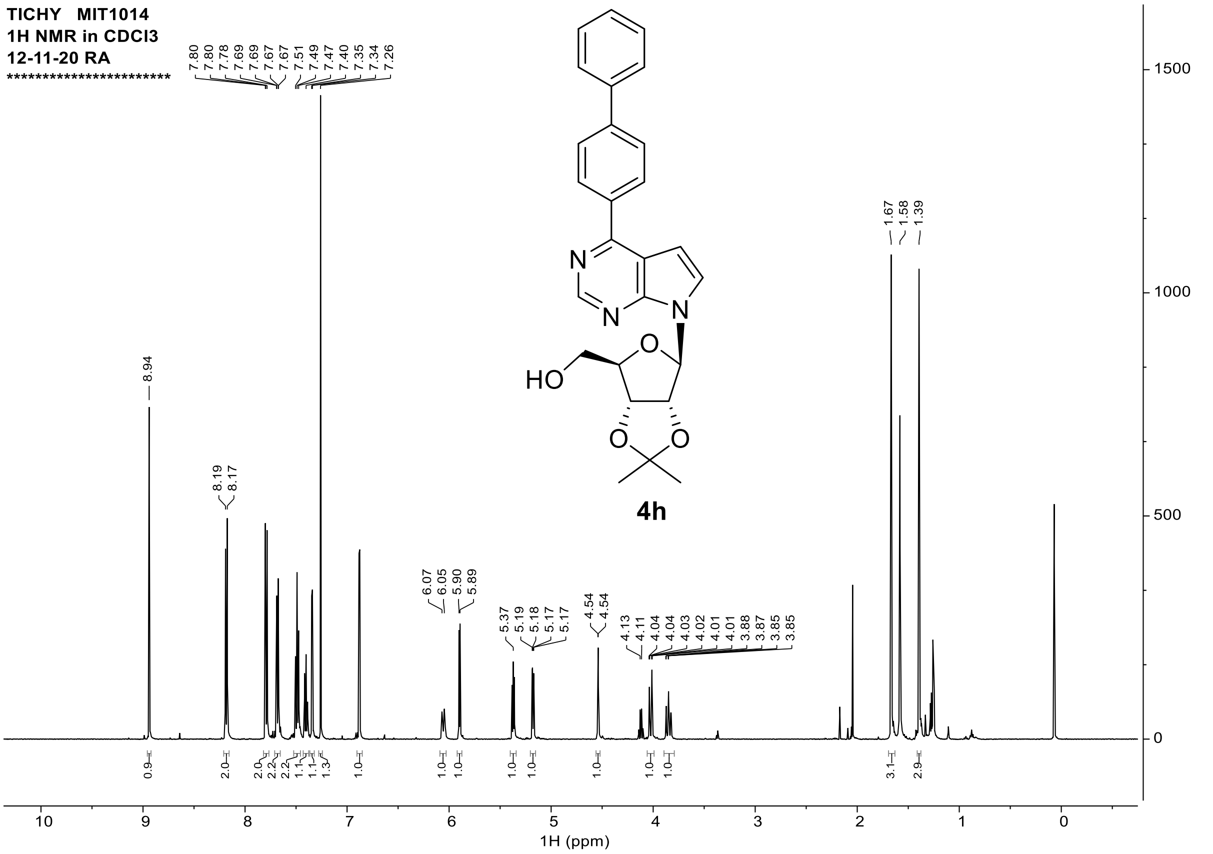
TICHY MIT1014
1H NMR in CDCl3
12-11-20 RA
7.80 7.80 7.78 7.69 7.69 7.67 7.67 7.51 7.49 7.47 7.40 7.35 7.34 7.26
1.67 1.58 1.39
8.94
8.19 8.17
6.07 6.05 5.90 5.89
5.37 5.19 5.18 5.17 5.17
4.54 4.54
4.13 4.11 4.04 4.04 4.03 4.02 4.01 4.01 3.88 3.87 3.85 3.85
1H (ppm)

4h

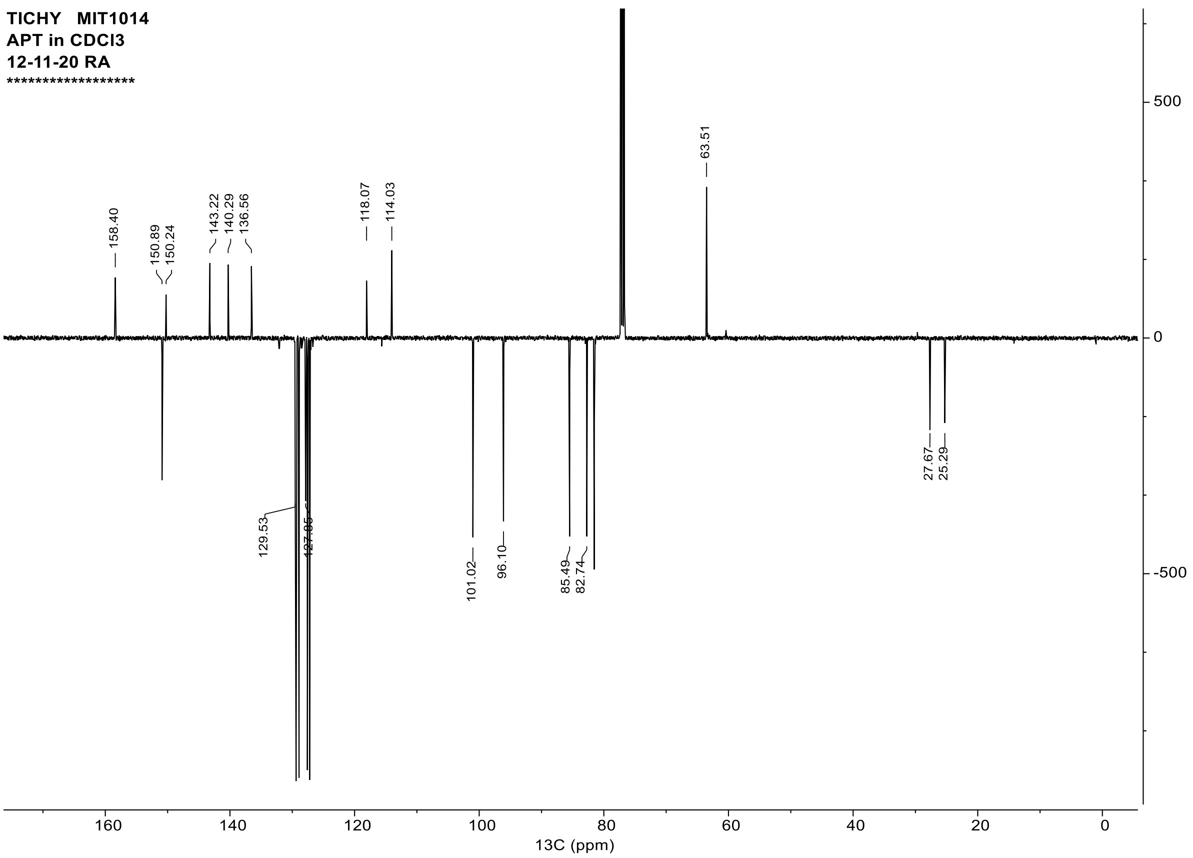
TICHY MIT1014
APT in CDCl3
12-11-20 RA
13C (ppm)

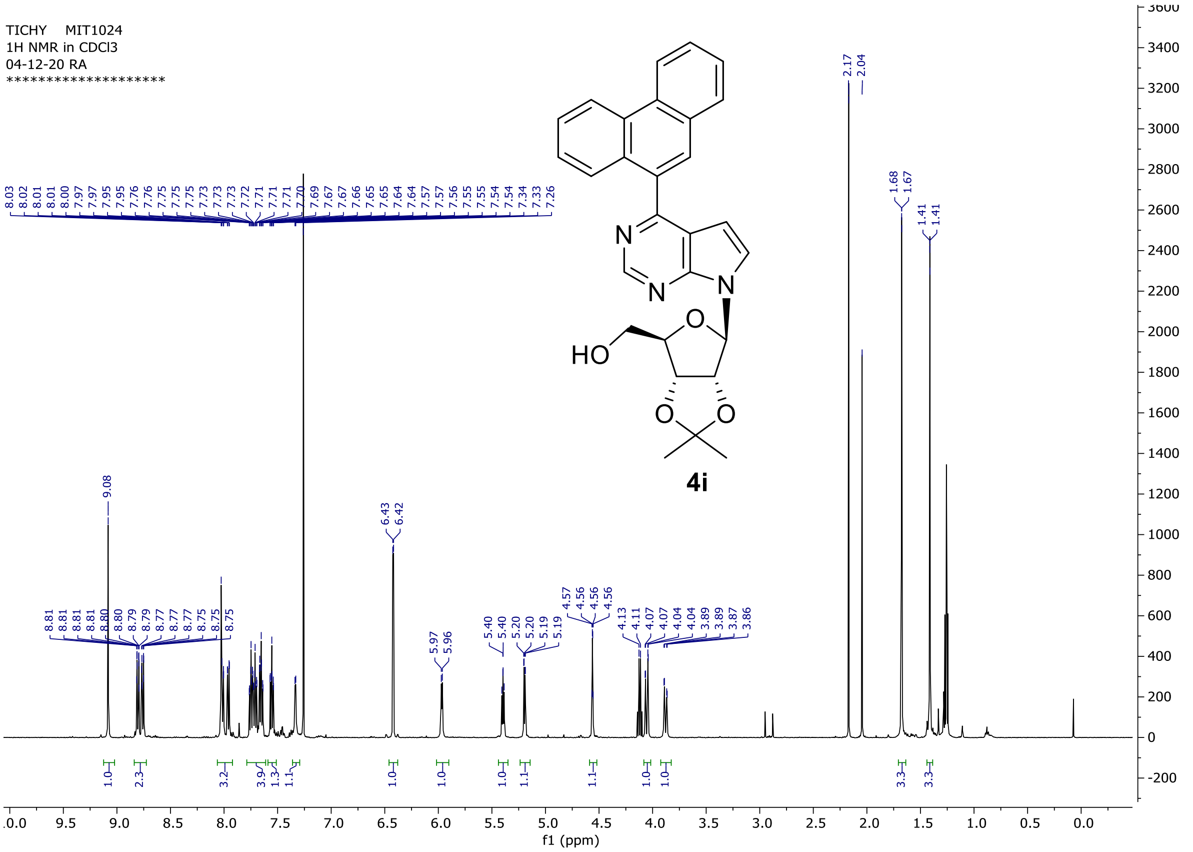
TICHY MIT1024
1H NMR in CDCl3
04-12-20 RA
HO
4i
f1 (ppm)

TICHY MIT1024
APT in CDCl3
04-12-20 RA
****

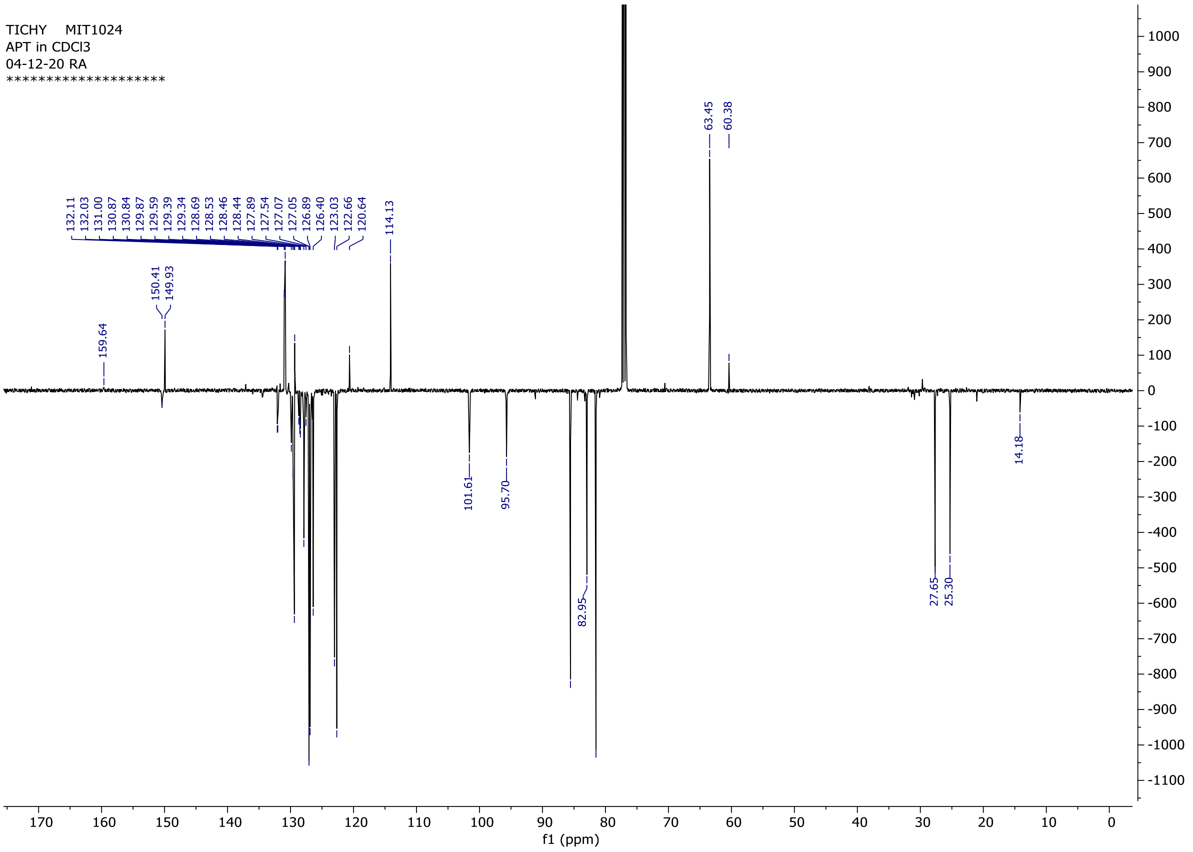

TICHY MIT1015F2
1H NMR in CDCl3
18-11-20 RA
****

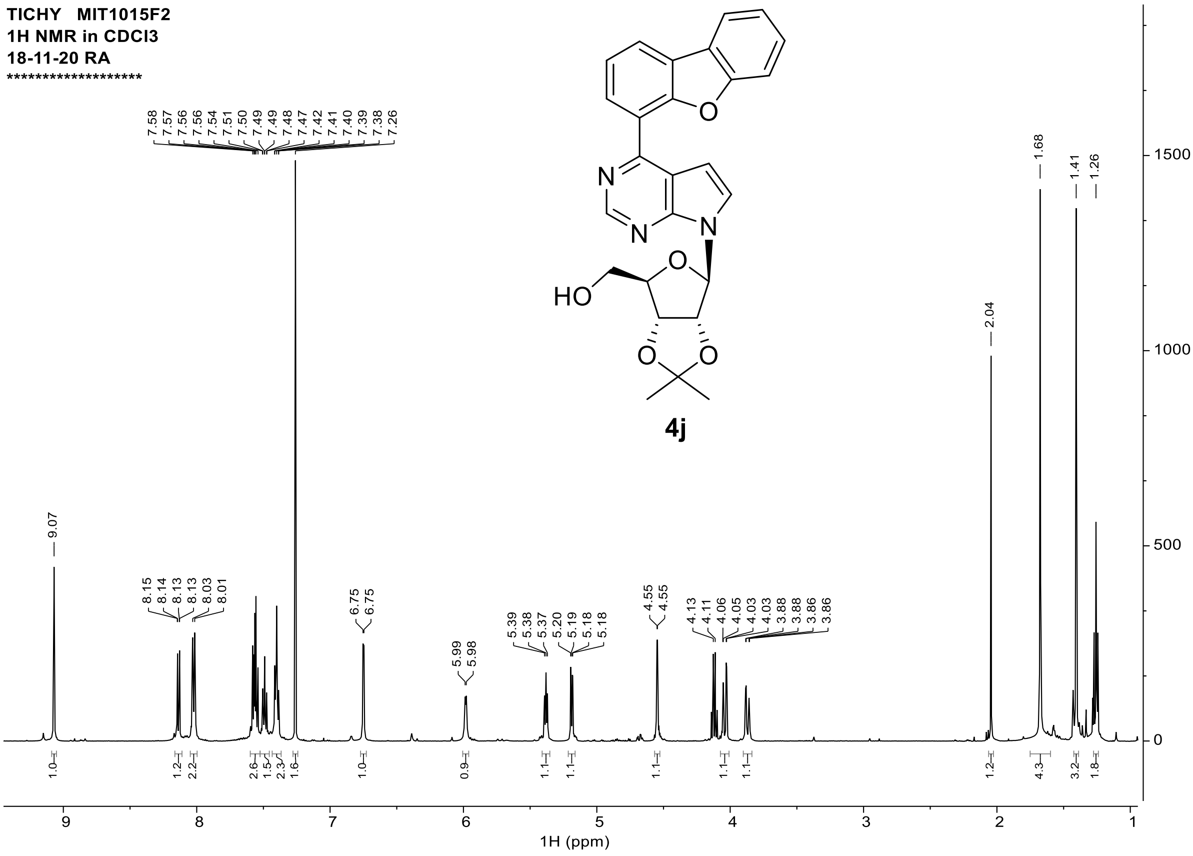

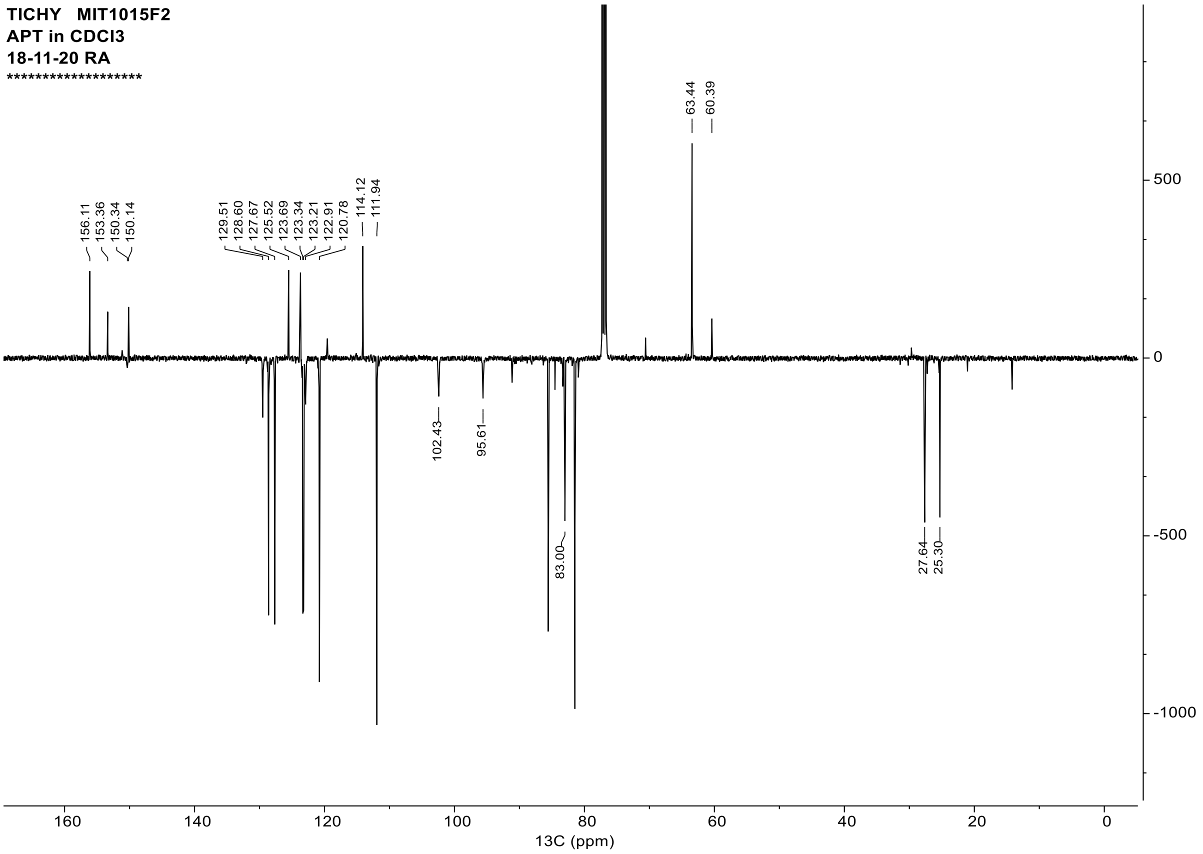
TICHY MIT1015F2
APT in CDCl3
18-11-20 RA
13C (ppm)

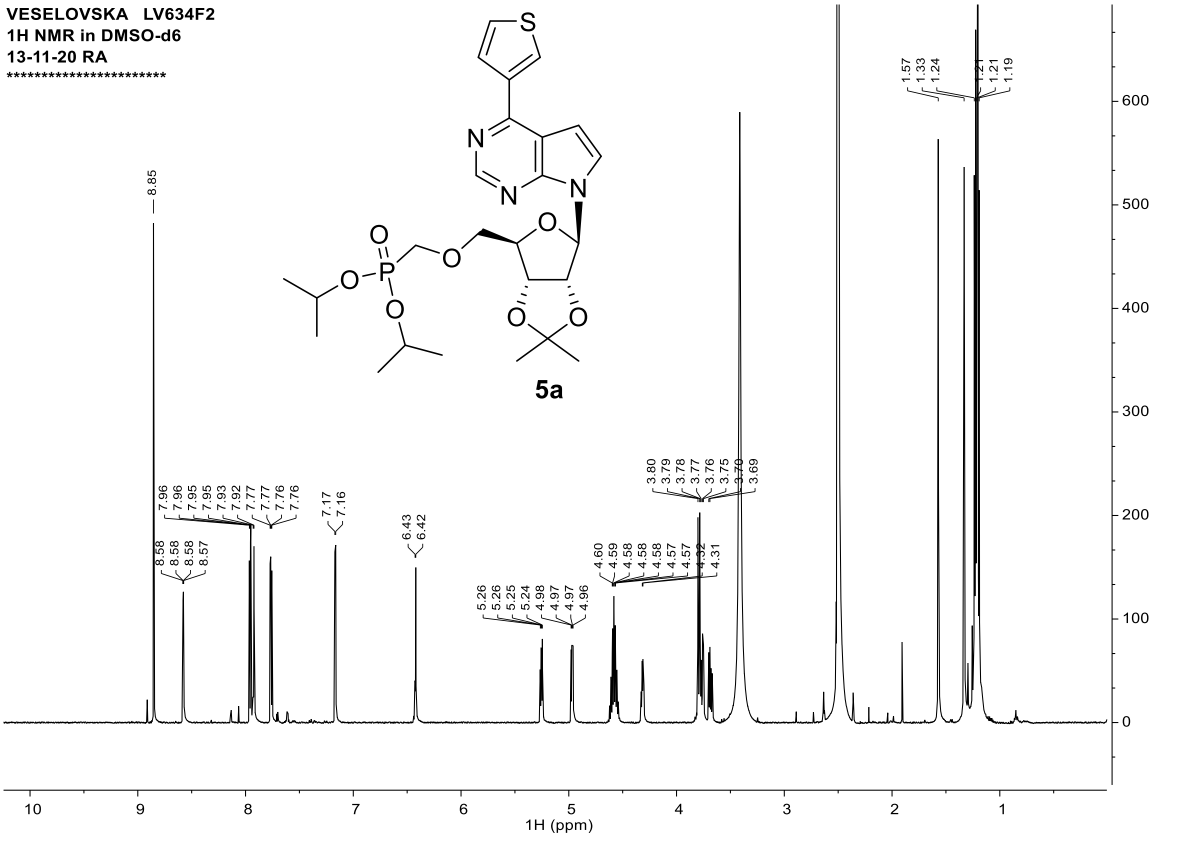
VESELOVSKA LV634F2
1H NMR in DMSO-d6
13-11-20 RA
5a
1H (ppm)

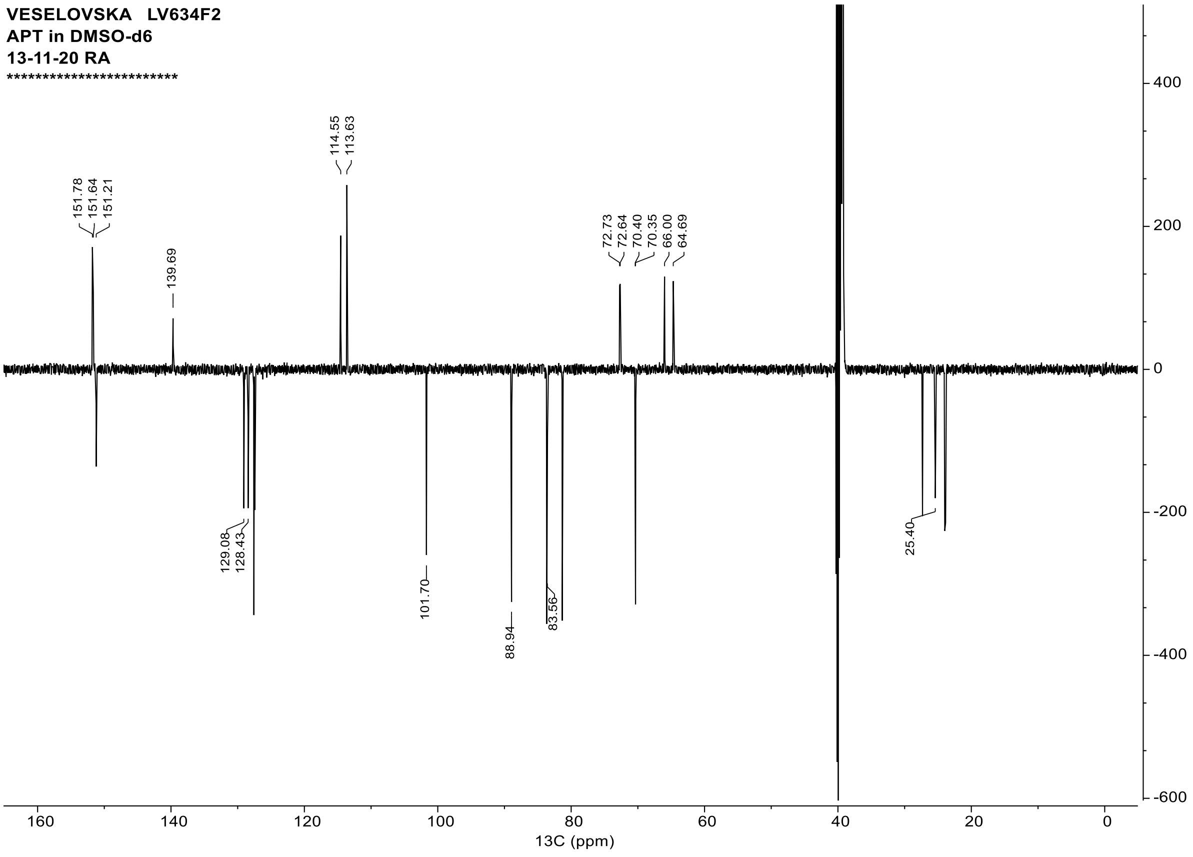

VESELOVSKA LV634F2
APT in DMSO-d6
13-11-20 RA
****
151.78
151.64
151.21
139.69
114.55
113.63
72.73
72.64
70.40
70.35
66.00
64.69
129.08
128.43
101.70
88.94
83.56
25.40
400
200
0
-200
-400
-600
160
140
120
100
80
60
40
20
0
13C (ppm)

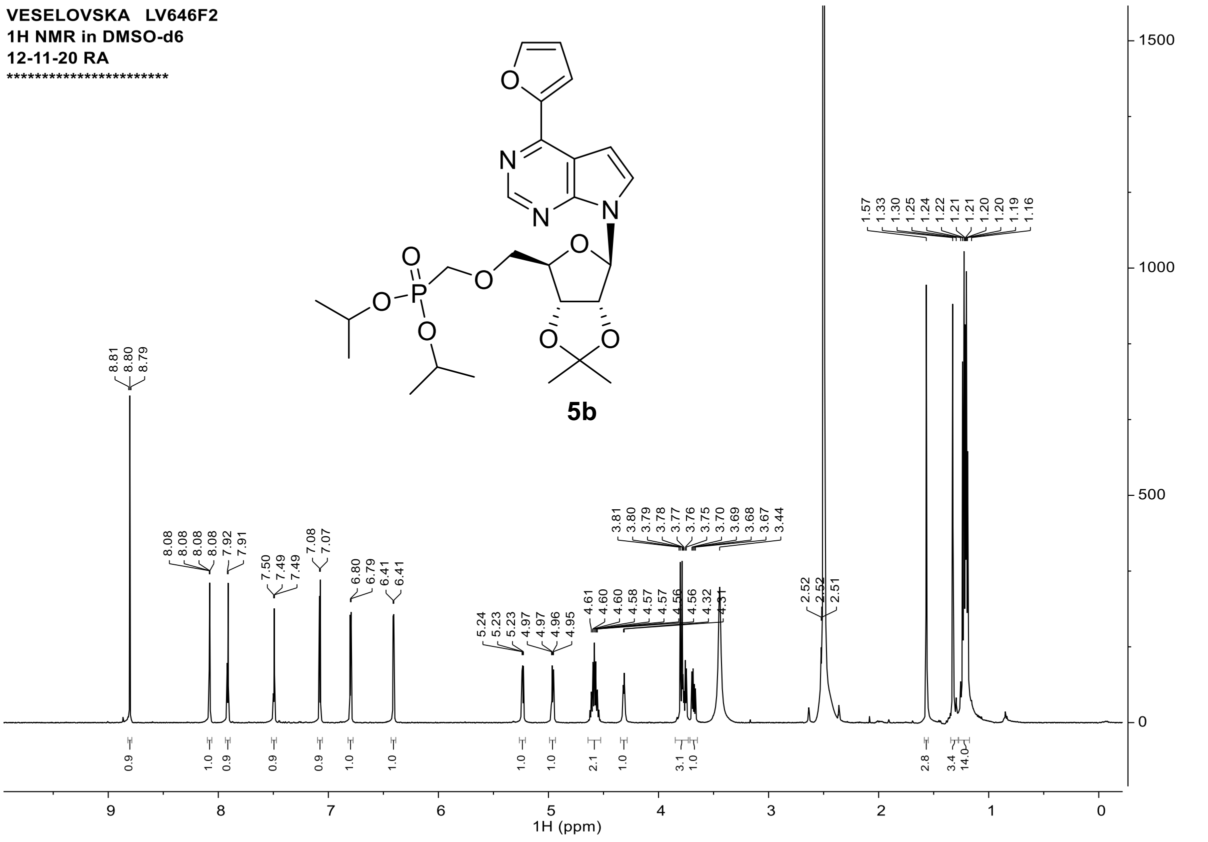

VESELOVSKA LV646F2
1H NMR in DMSO-d6
12-11-20 RA
****
5b
8.81
8.80
8.79
8.08
8.08
8.08
8.08
7.92
7.91
7.50
7.49
7.49
7.08
7.07
6.80
6.79
6.41
6.41
5.24
5.23
5.23
4.97
4.97
4.96
4.95
4.61
4.60
4.60
4.58
4.57
4.57
4.56
4.56
4.32
4.31
3.81
3.80
3.79
3.78
3.77
3.76
3.75
3.70
3.69
3.68
3.67
3.44
2.52
2.52
2.51
1.57
1.33
1.30
1.25
1.24
1.22
1.21
1.21
1.20
1.20
1.19
1.16
0.9
1.0
0.9
0.9
0.9
1.0
1.0
1.0
1.0
2.1
1.0
3.1
1.0
2.8
3.4
14.0
1H (ppm)

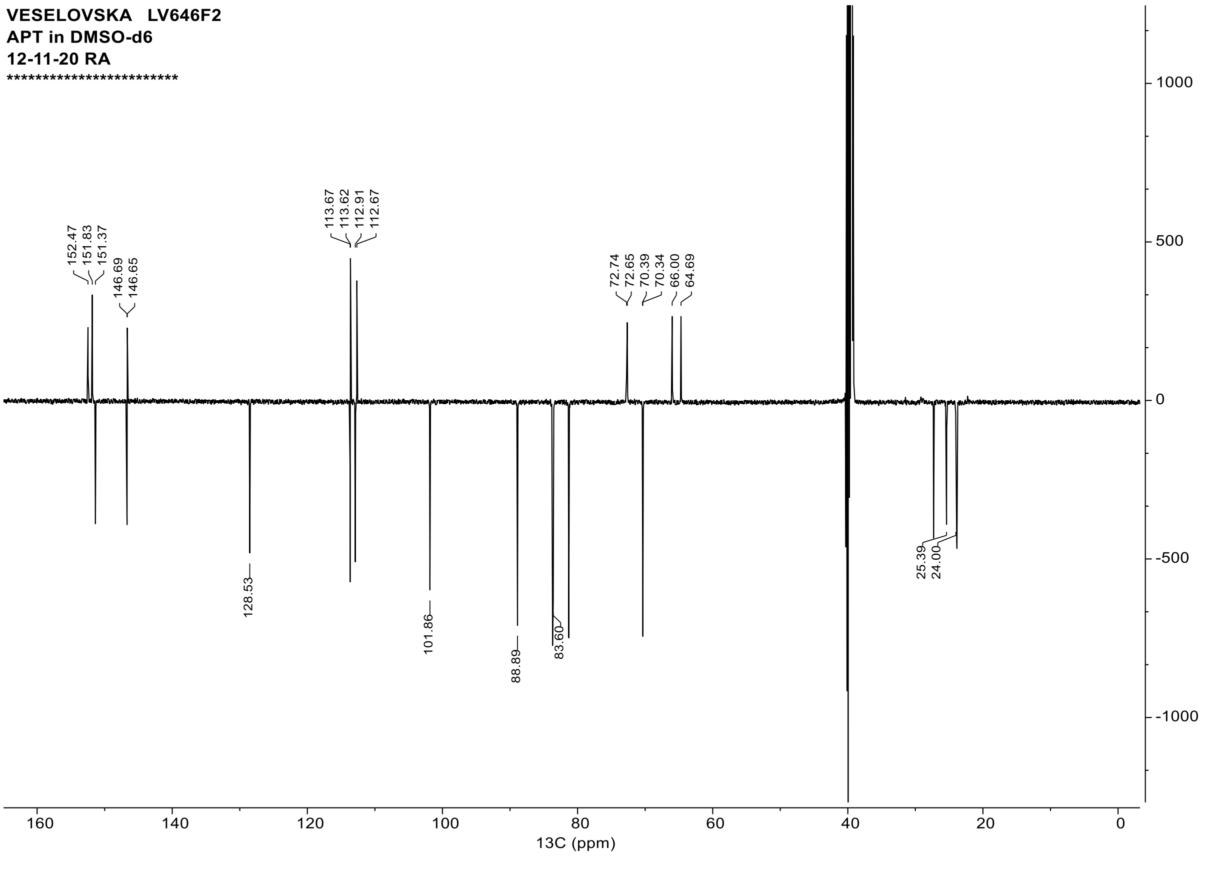

VESELOVSKA LV646F2
APT in DMSO-d6
12-11-20 RA
****
152.47
151.83
151.37
146.69
146.65
113.67
113.62
112.91
112.67
128.53
101.86
88.89
83.60
72.74
72.65
70.39
70.34
66.00
64.69
25.39
24.00
13C (ppm)

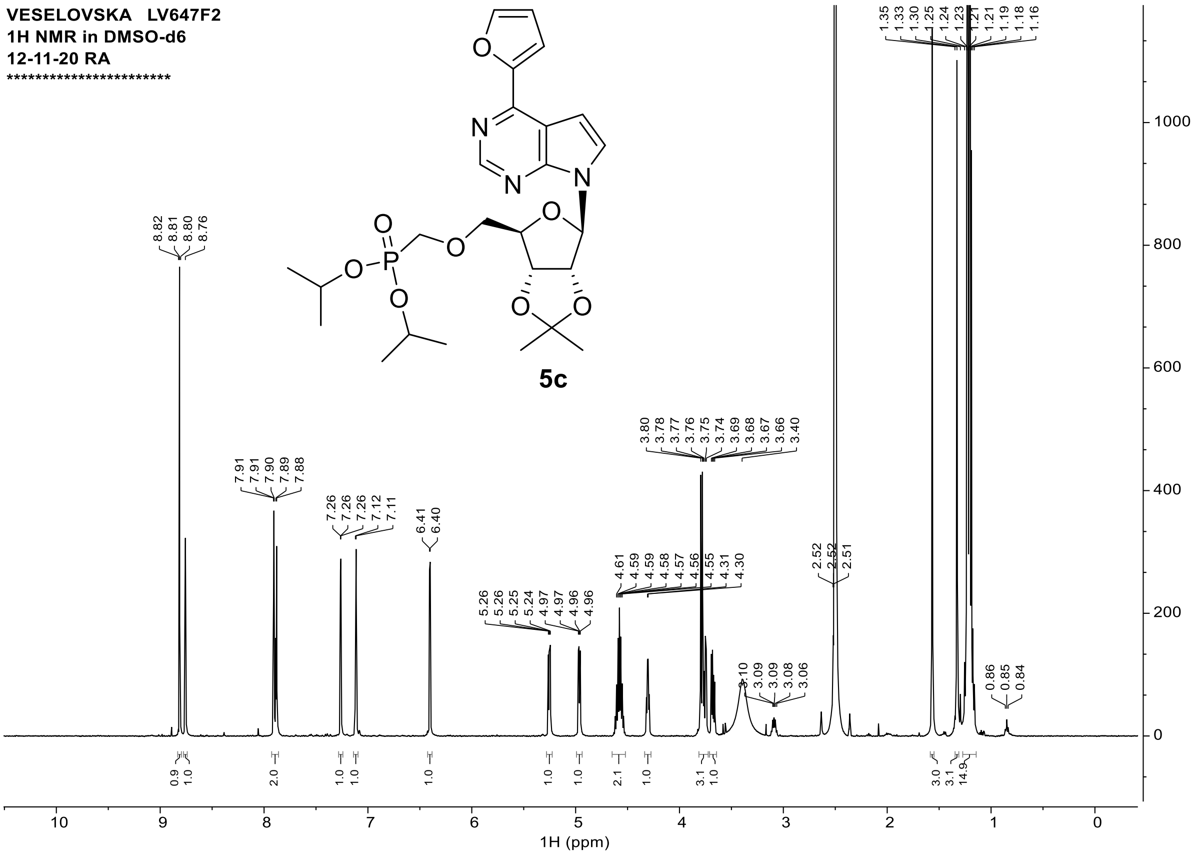
VESELOVSKA LV647F2
1H NMR in DMSO-d6
12-11-20 RA
5c
1H (ppm)

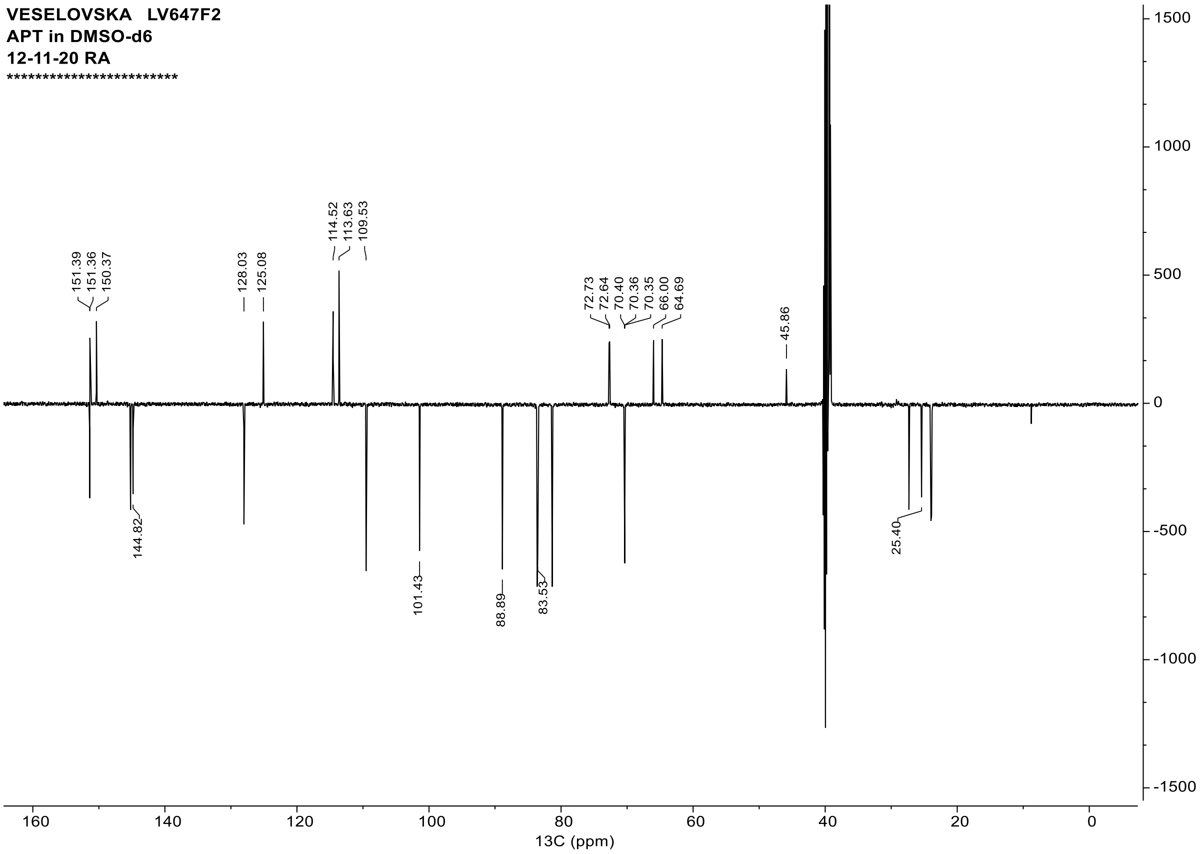
VESELOVSKA LV647F2
APT in DMSO-d6
12-11-20 RA
13C (ppm)

TICHY MIT1018F3
1H NMR in DMSO-d6
18-11-20 RA
****

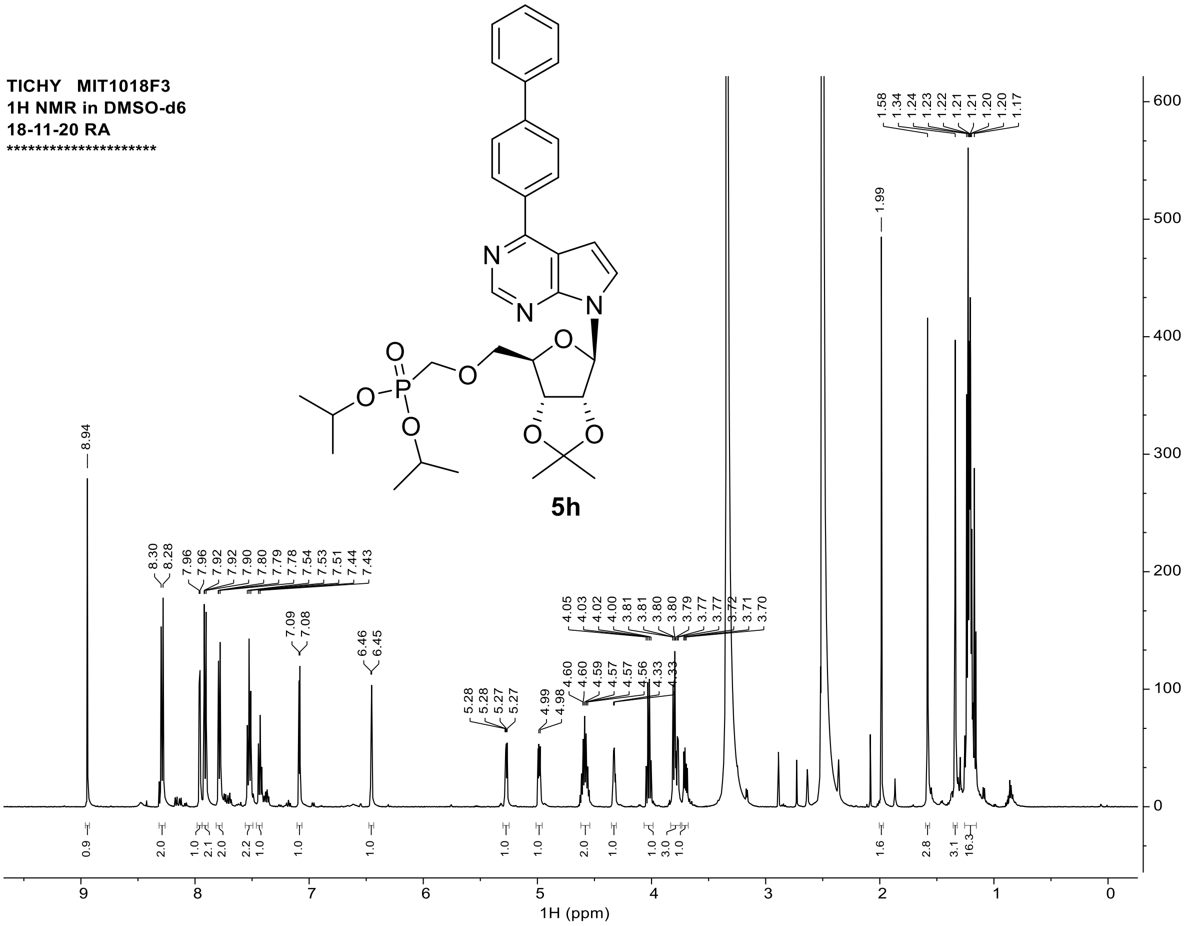

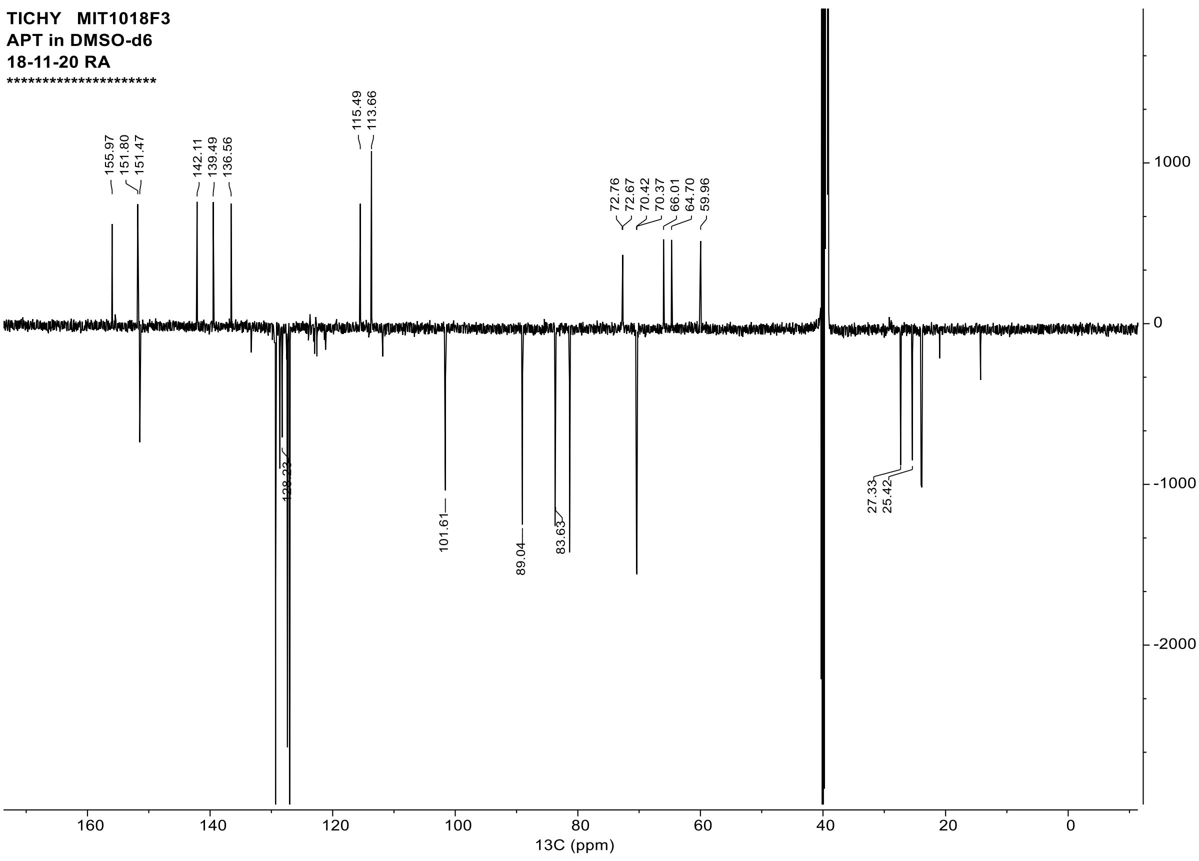
TICHY MIT1018F3
APT in DMSO-d6
18-11-20 RA
155.97
151.80
151.47
142.11
139.49
136.56
115.49
113.66
72.76
72.67
70.42
70.37
66.01
64.70
59.96
128.23
101.61
89.04
83.63
27.33
25.42
13C (ppm)

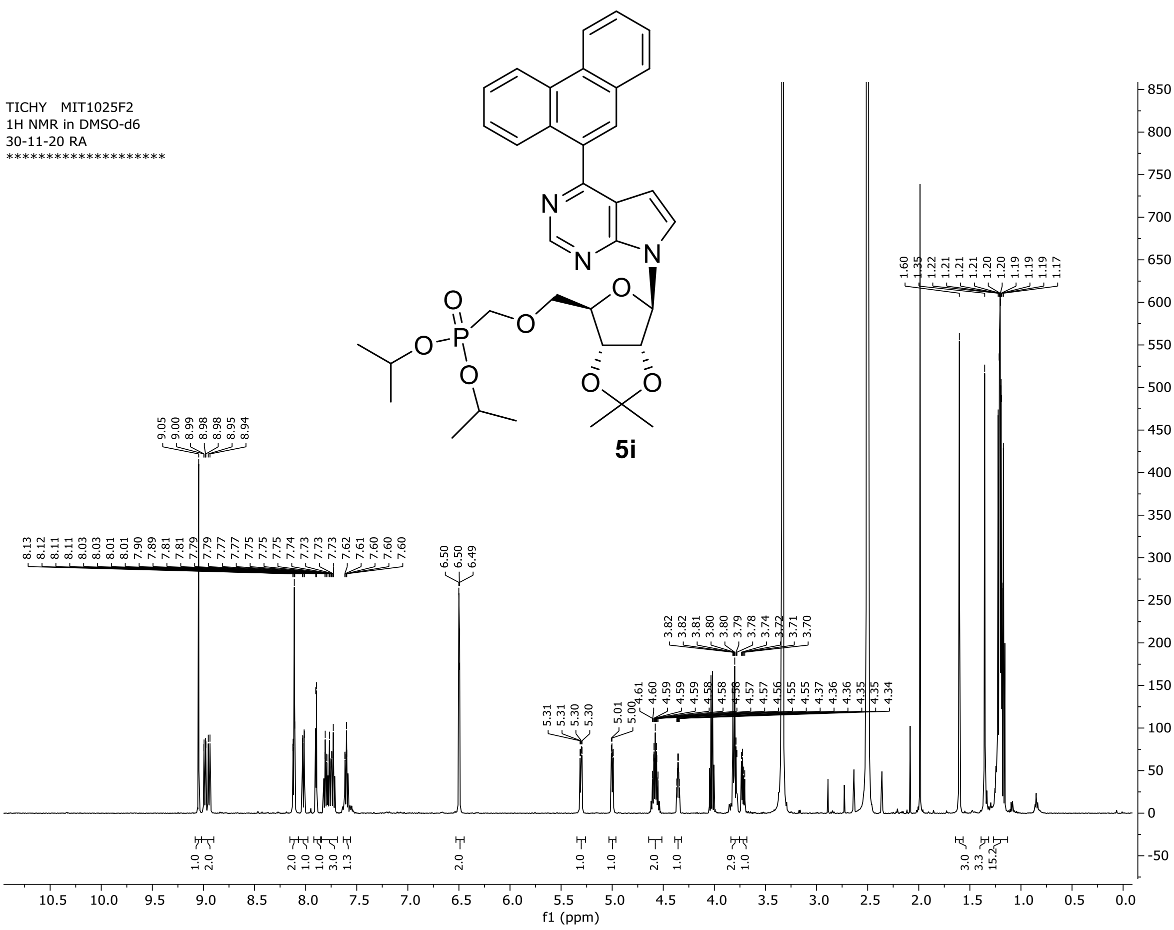
TICHY MIT1025F2
1H NMR in DMSO-d6
30-11-20 RA
5i
f1 (ppm)

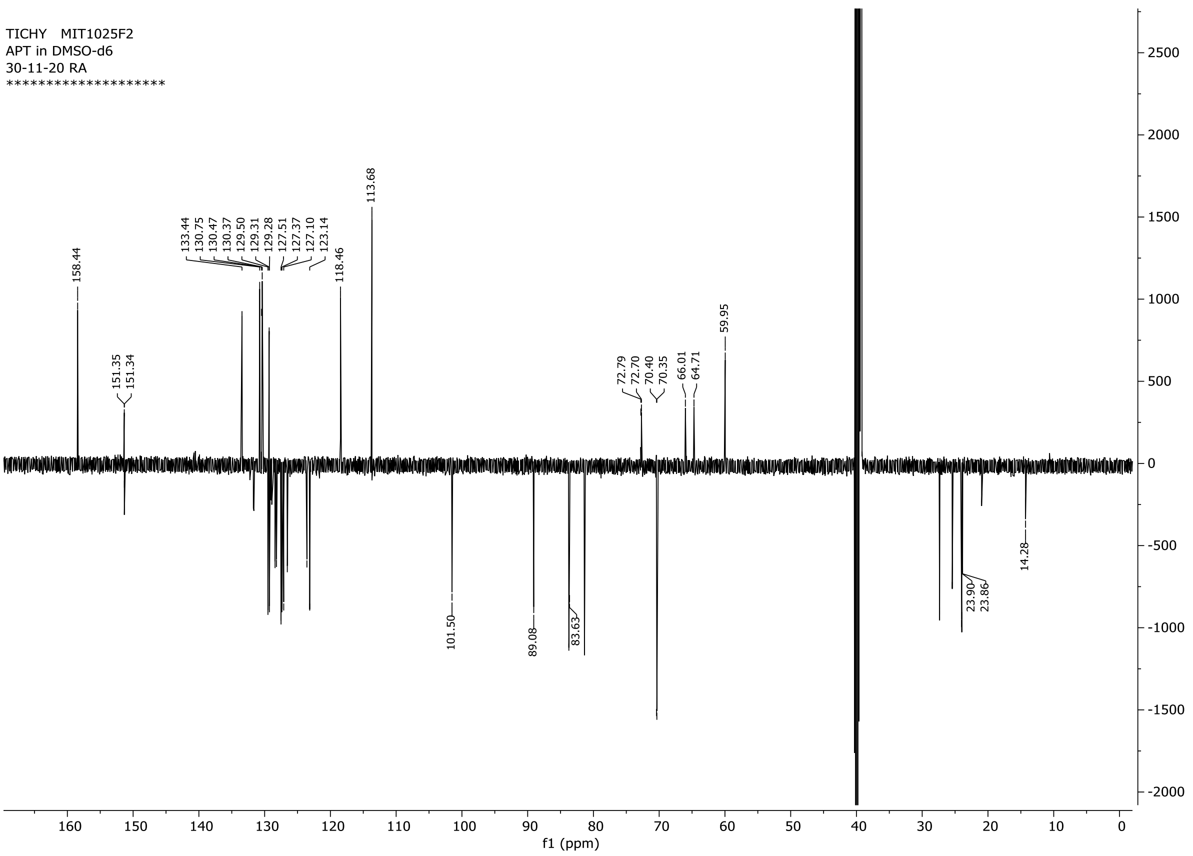
TICHY MIT1025F2
APT in DMSO-d6
30-11-20 RA
****
f1 (ppm)

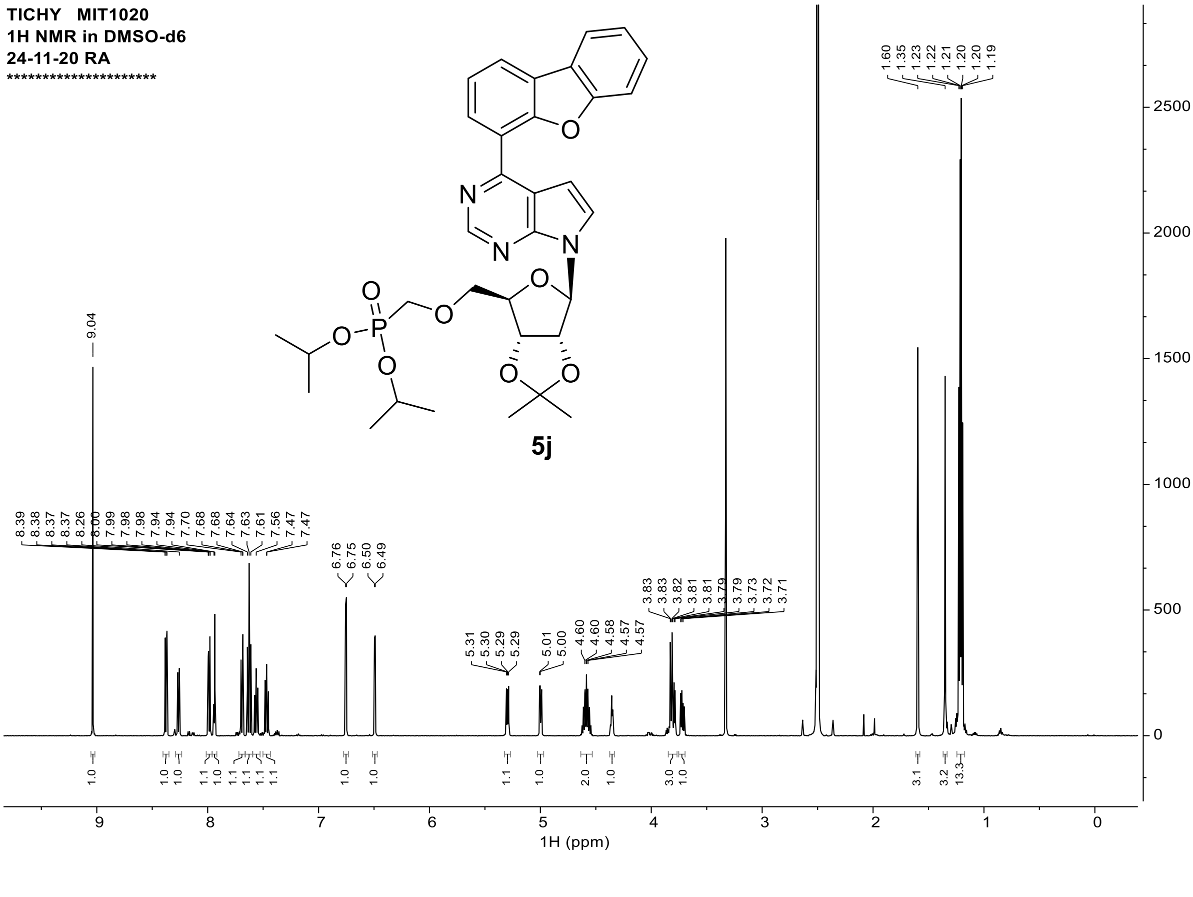
TICHY MIT1020
1H NMR in DMSO-d6
24-11-20 RA
****
5j
1H (ppm)

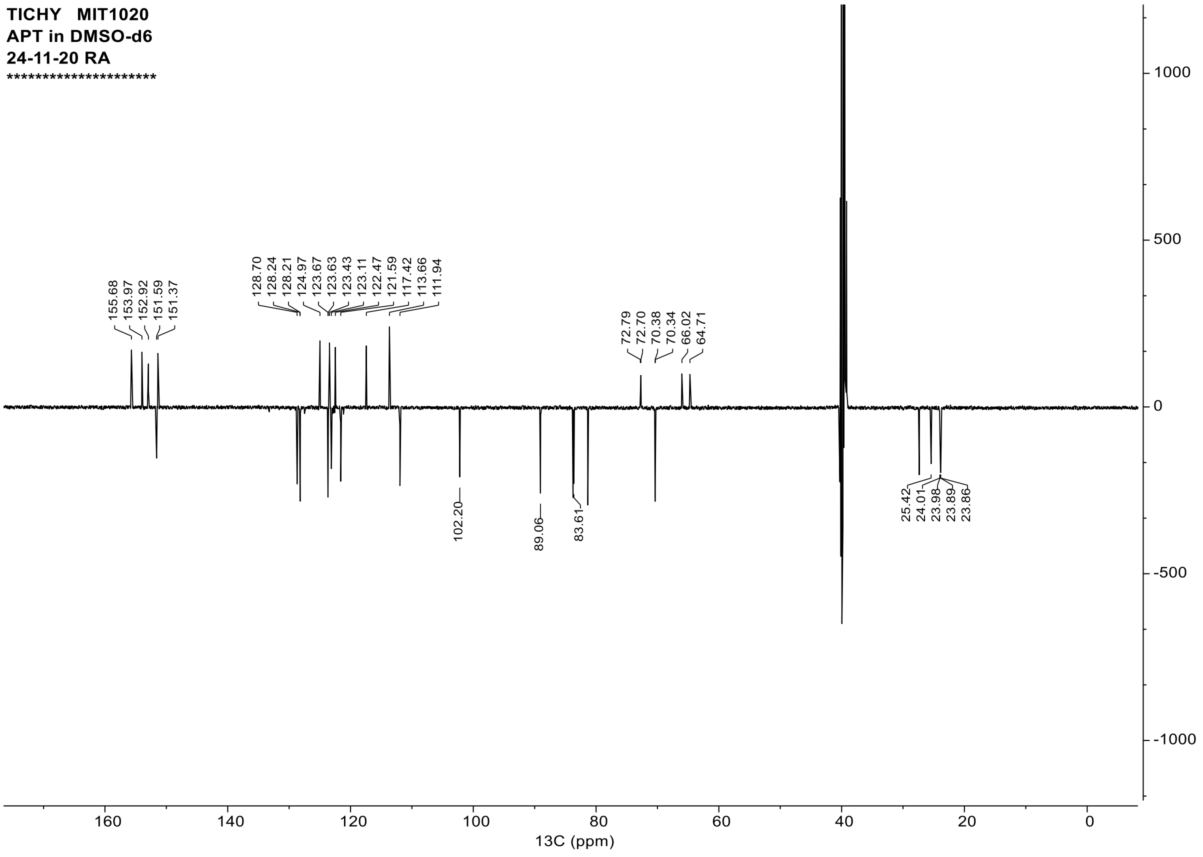
TICHY MIT1020
APT in DMSO-d6
24-11-20 RA
****
13C (ppm)

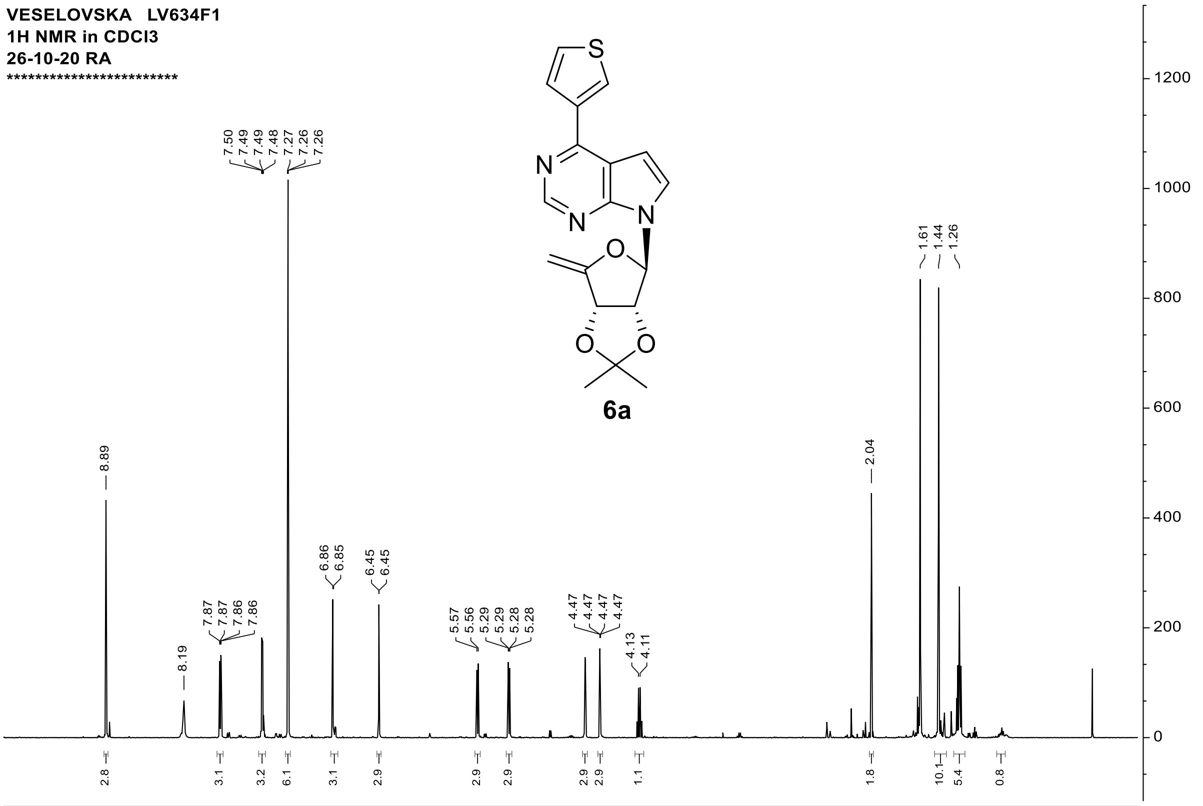
VESELOVSKA LV634F1
1H NMR in CDCl3
26-10-20 RA
****
6a
1H (ppm)

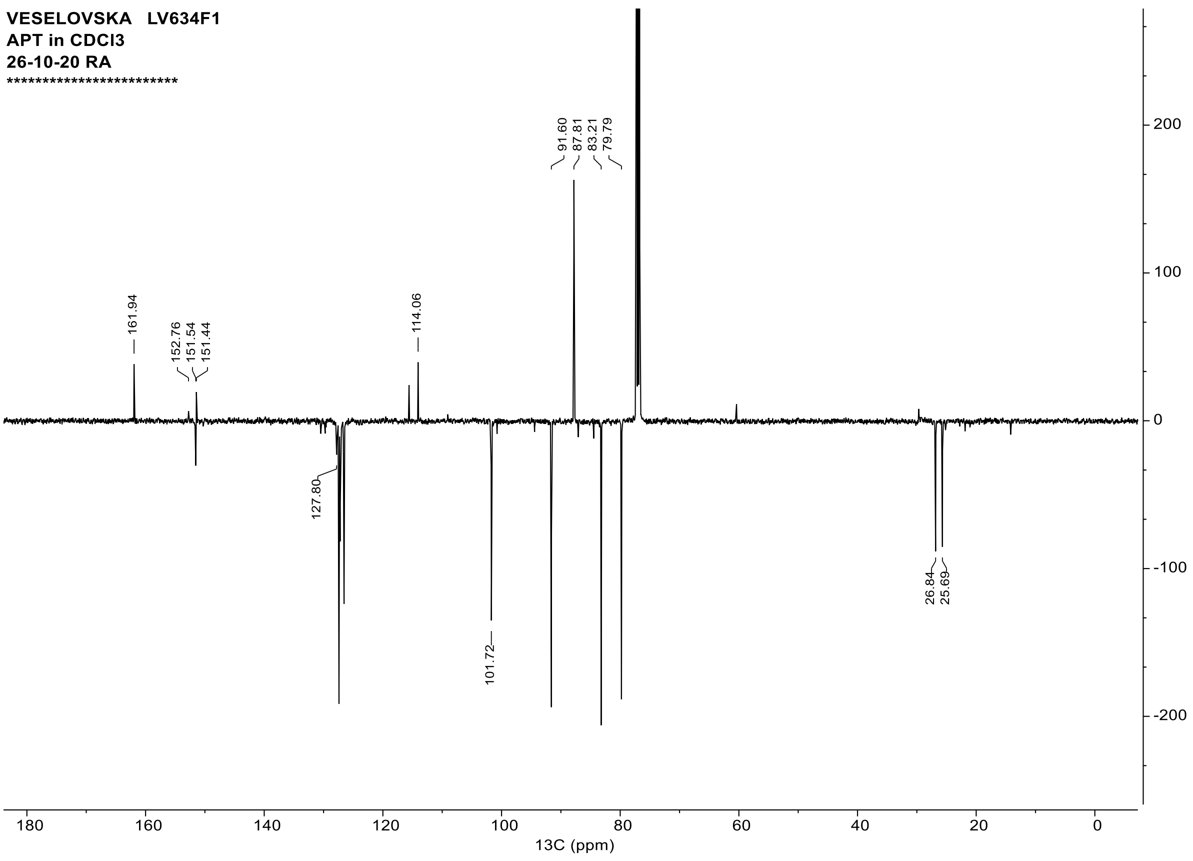
VESELOVSKA LV634F1
APT in CDCl3
26-10-20 RA
****
161.94
152.76
151.54
151.44
127.80
114.06
101.72
91.60
87.81
83.21
79.79
26.84
25.69
13C (ppm)

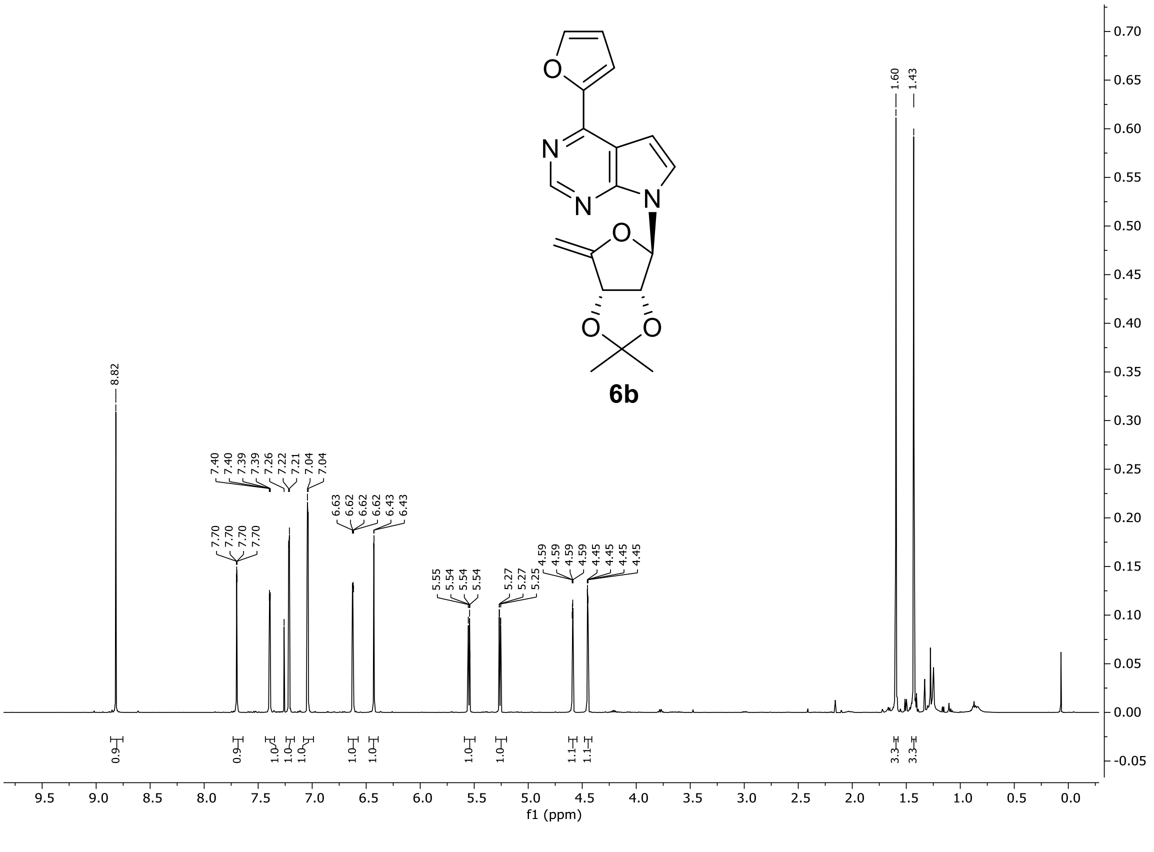
6b
8.82
7.70
7.40
7.39
7.26
7.22
7.21
7.04
6.63
6.62
6.43
5.55
5.54
5.27
5.25
4.59
4.45
1.60
1.43
f1 (ppm)

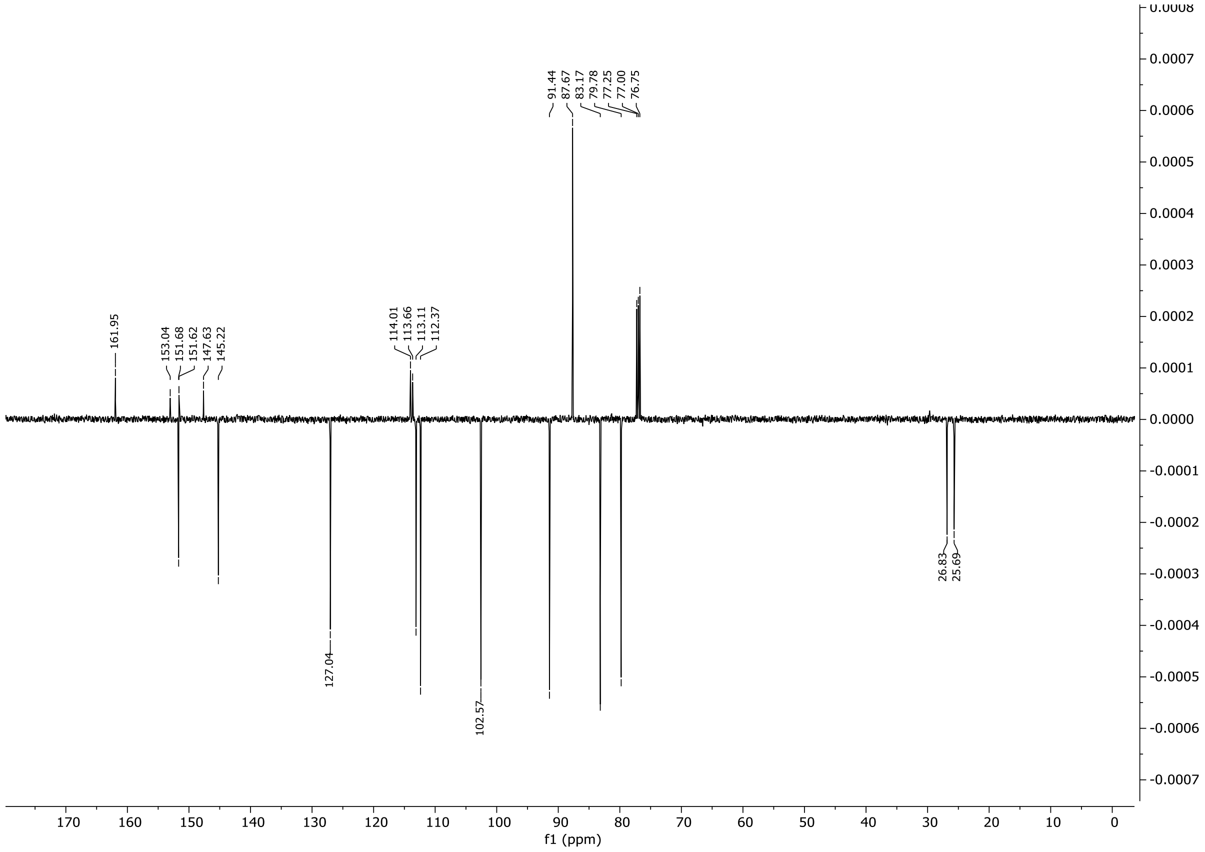
161.95
153.04
151.68
151.62
147.63
145.22
114.01
113.66
113.11
112.37
91.44
87.67
83.17
79.78
77.25
77.00
76.75
127.04
102.57
26.83
25.69
f1 (ppm)

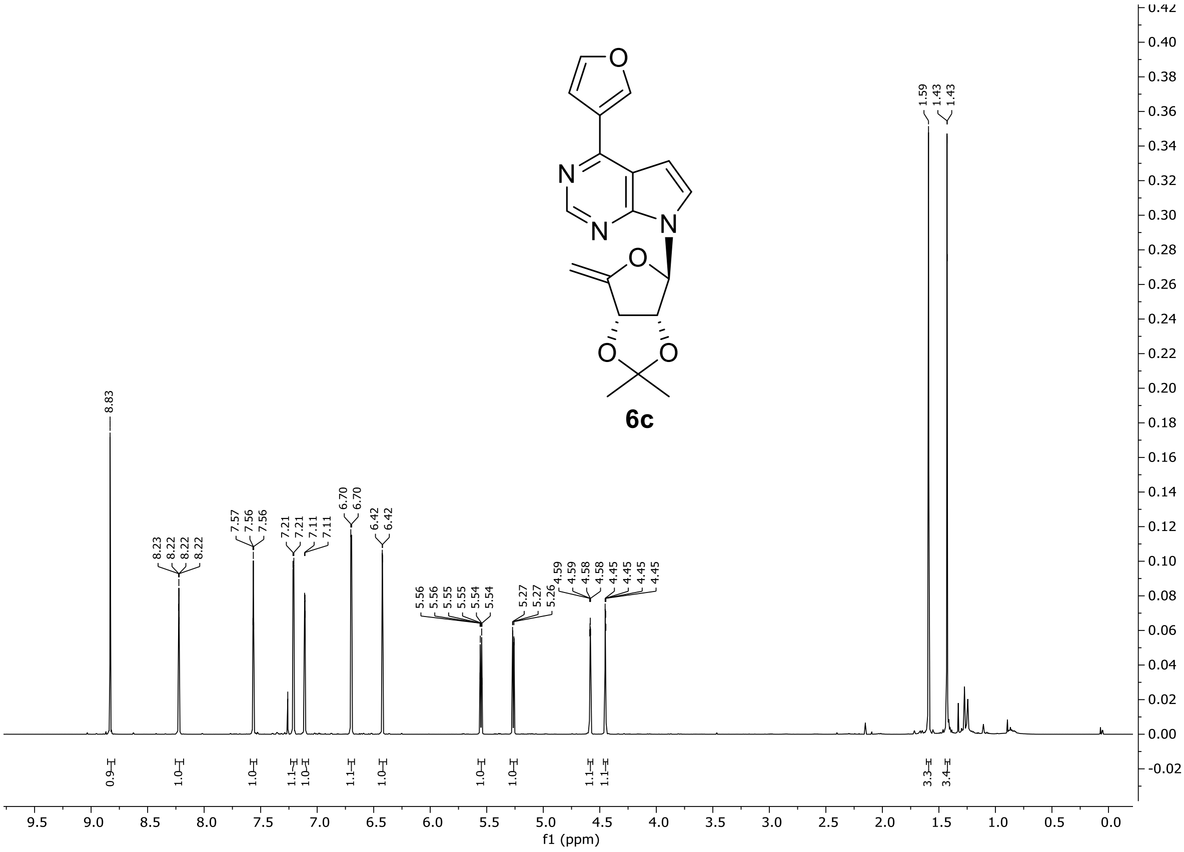
6c
f1 (ppm)

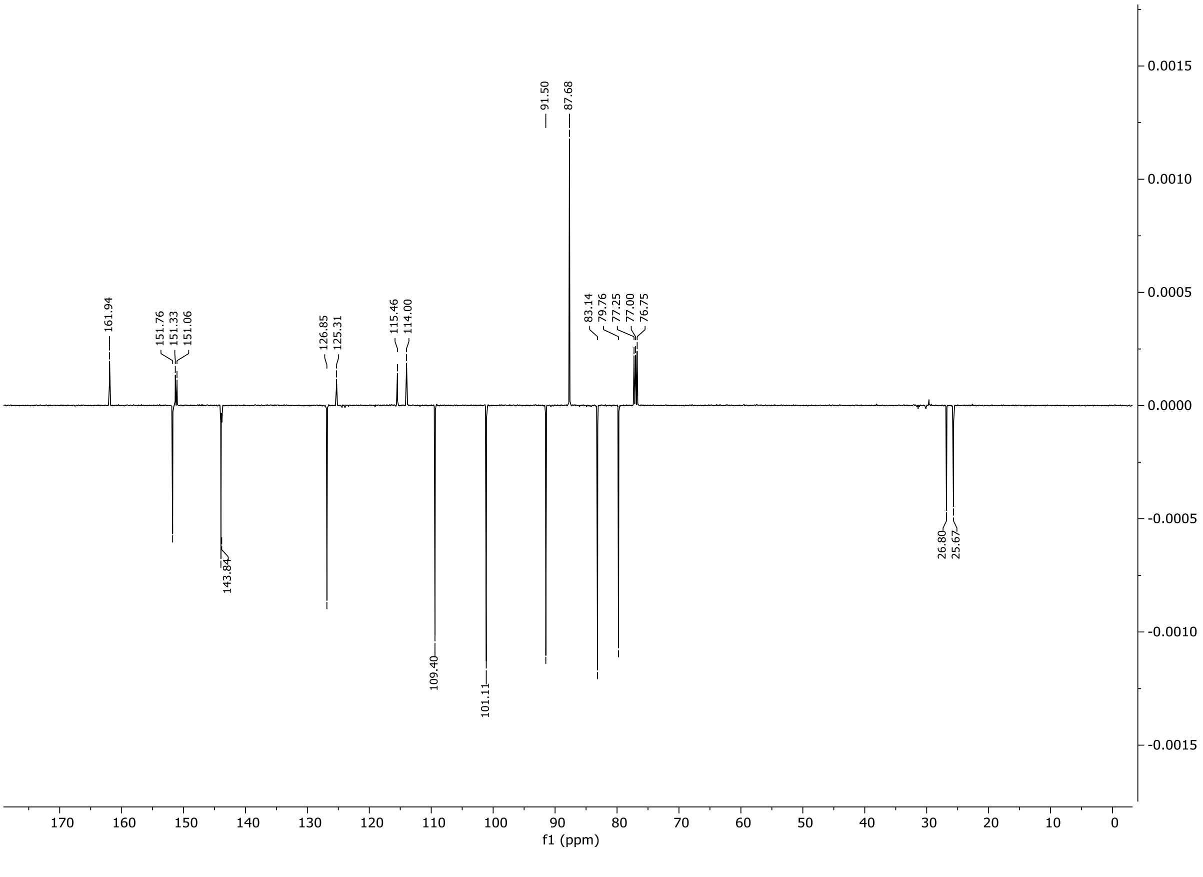
f1 (ppm)

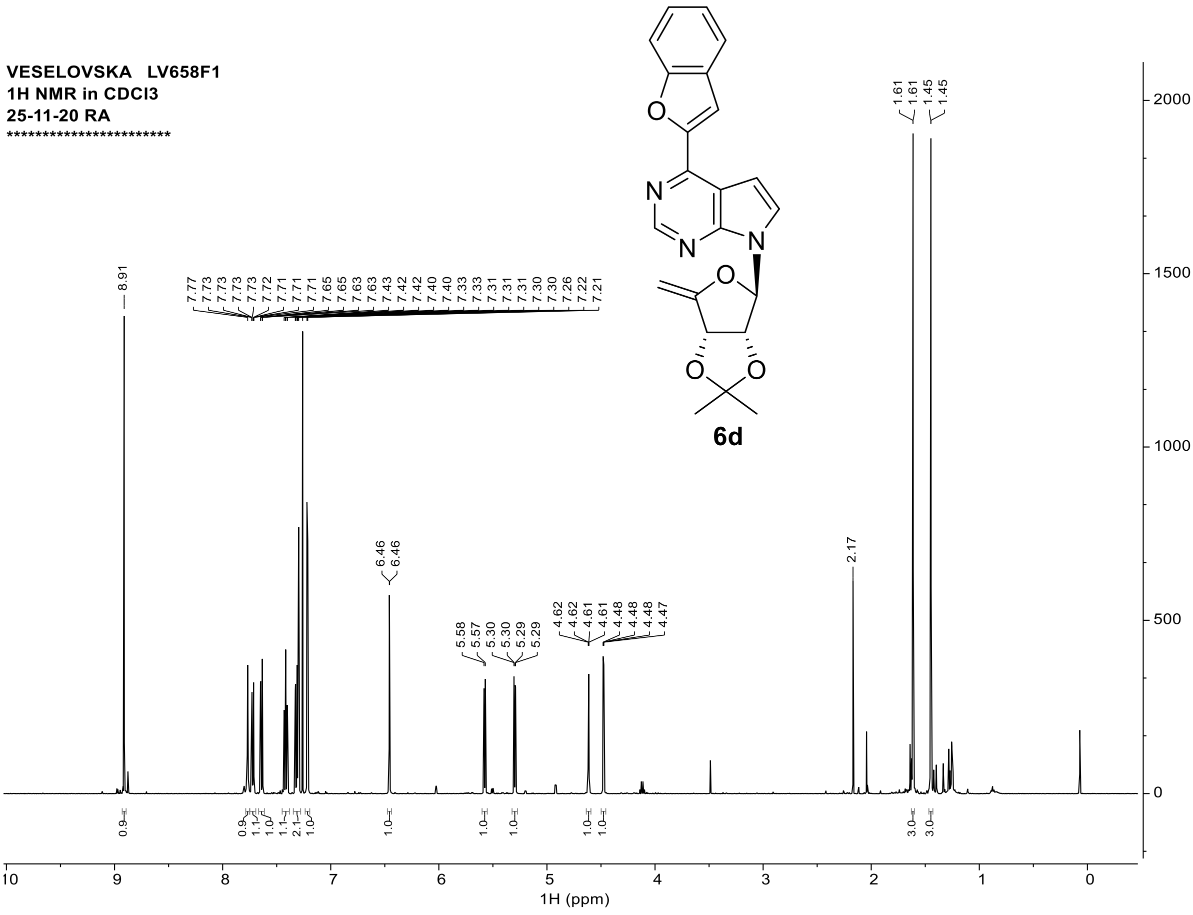
VESELOVSKA LV658F1
1H NMR in CDCl3
25-11-20 RA
****
6d
8.91
7.77 7.73 7.73 7.73 7.73 7.72 7.71 7.71 7.71 7.71 7.65 7.65 7.63 7.63 7.43 7.42 7.42 7.40 7.40 7.33 7.33 7.31 7.31 7.31 7.30 7.30 7.26 7.22 7.21
6.46 6.46
5.58 5.57 5.30 5.30 5.29 5.29
4.62 4.62 4.61 4.61 4.48 4.48 4.48 4.47
2.17
1.61 1.61 1.45 1.45
0.9 0.9 1.1 1.0 1.1 2.1 1.0 1.0 1.0 1.0 1.0 1.0 3.0 3.0
1H (ppm)

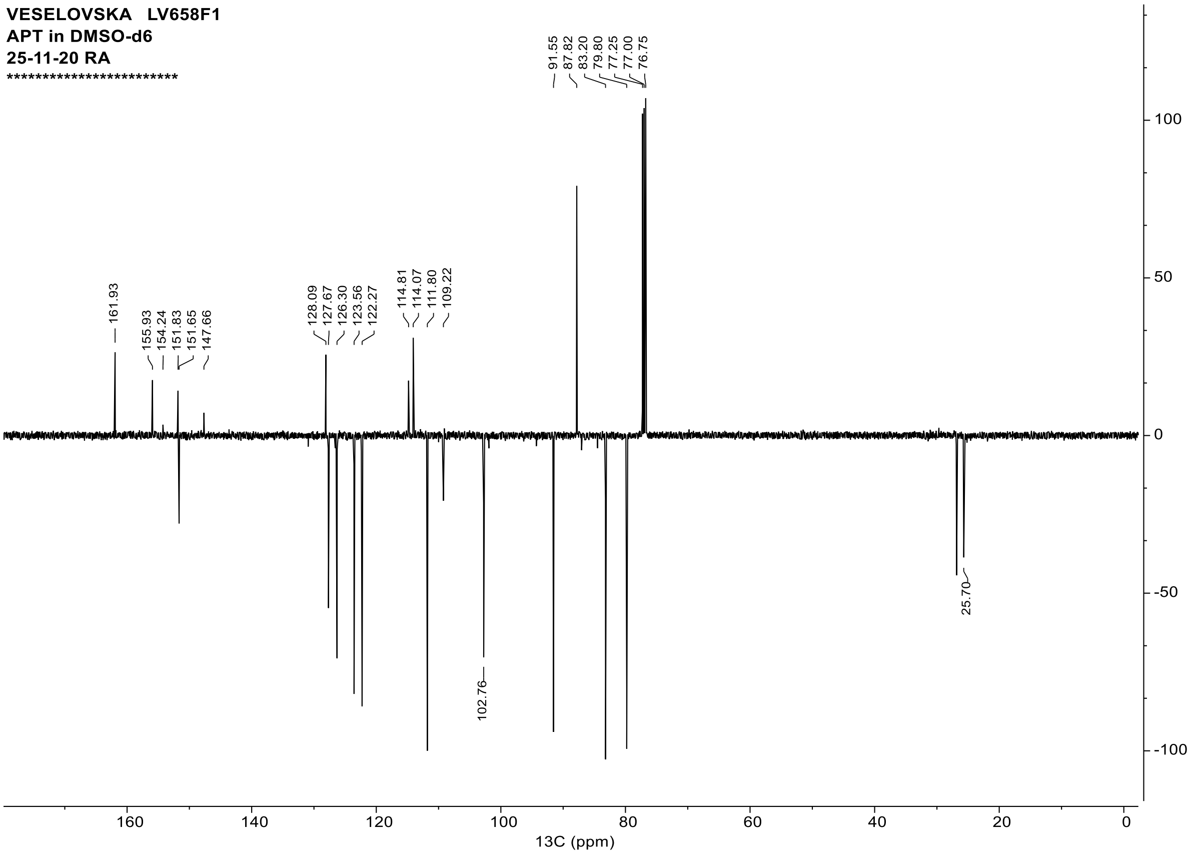
VESELOVSKA LV658F1
APT in DMSO-d6
25-11-20 RA
****
161.93
155.93 154.24 151.83 151.65 147.66
128.09 127.67 126.30 123.56 122.27
114.81 114.07 111.80 109.22
102.76
91.55 87.82 83.20 79.80 77.25 77.00 76.75
25.70
13C (ppm)

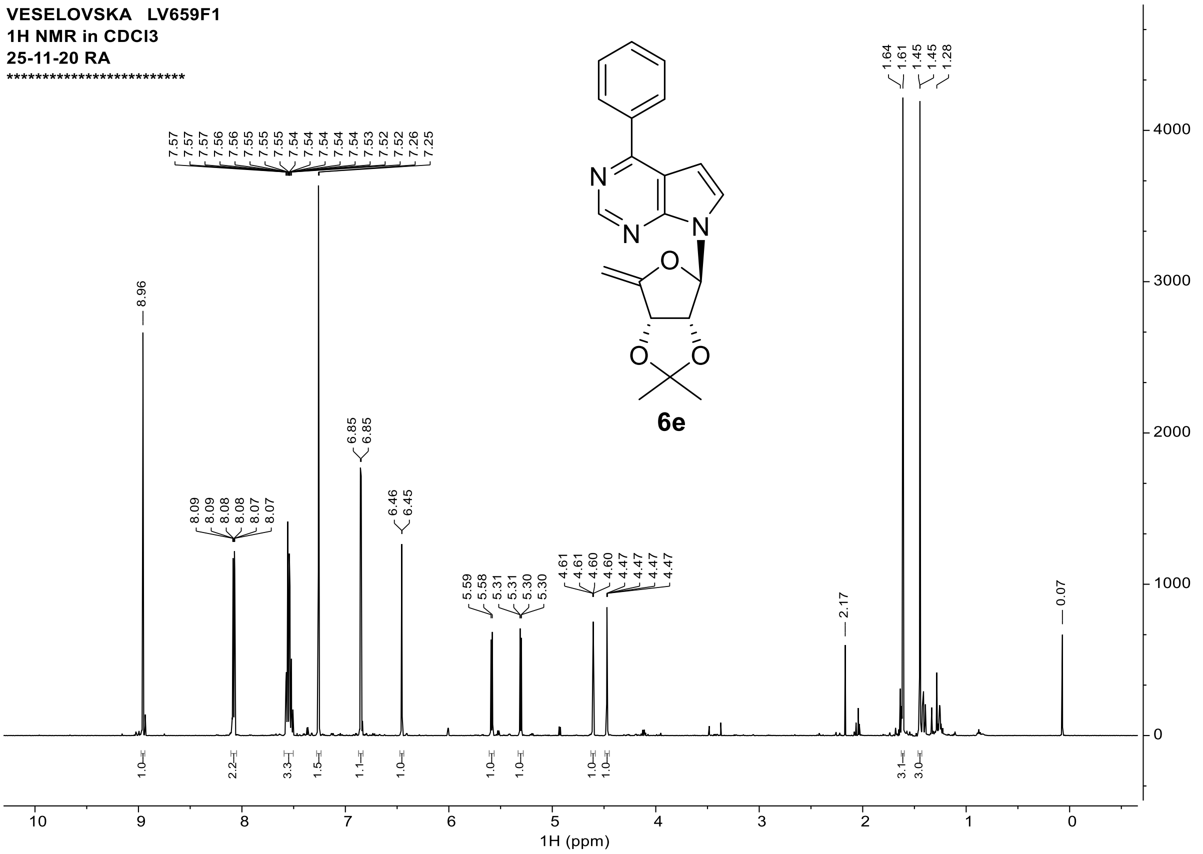

VESELOVSKA LV659F1
1H NMR in CDCl3
25-11-20 RA
6e
1H (ppm)

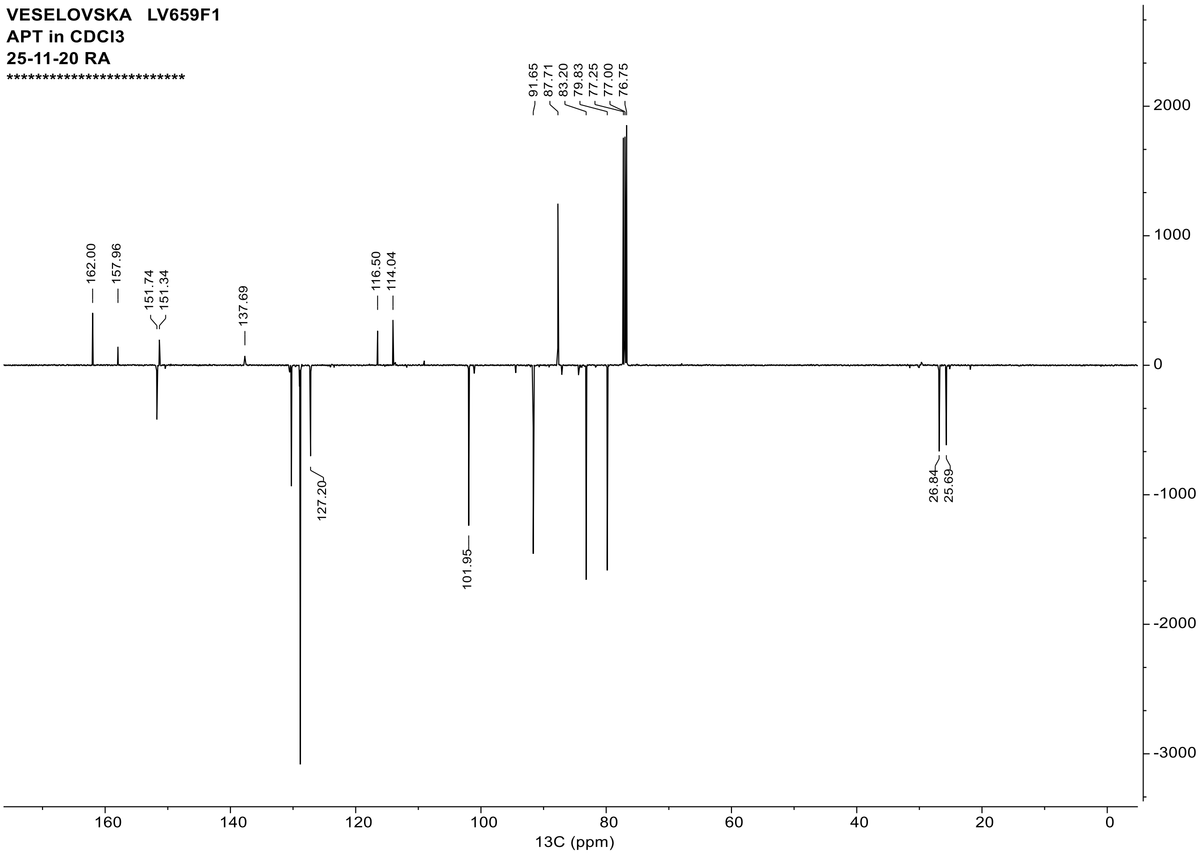

VESELOVSKA LV659F1
APT in CDCl3
25-11-20 RA
13C (ppm)

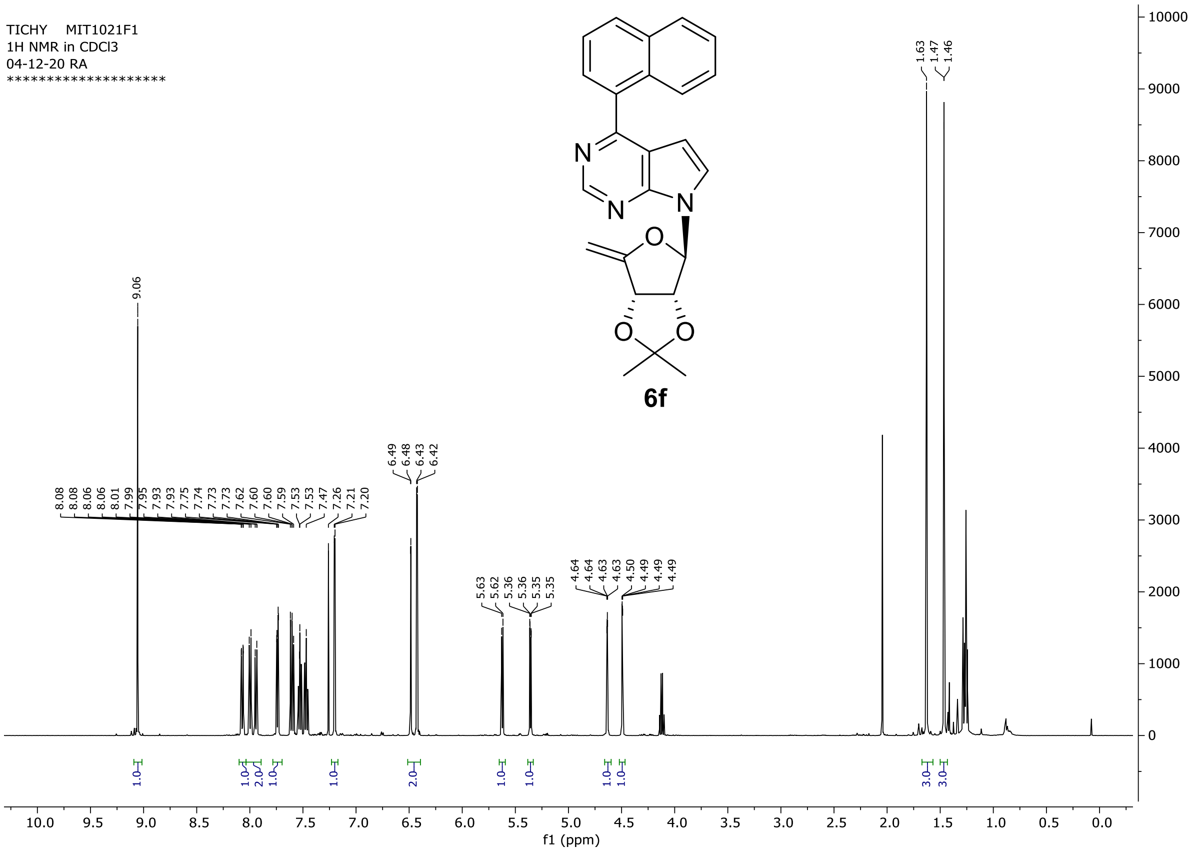
TICHY MIT1021F1
1H NMR in CDCl3
04-12-20 RA
****
6f
9.06
8.08 8.08 8.06 8.06 8.01 7.99 7.95 7.93 7.93 7.75 7.74 7.73 7.73 7.62 7.60 7.60 7.59 7.53 7.53 7.47 7.26 7.21 7.20
6.49 6.48 6.43 6.42
5.63 5.62 5.36 5.36 5.35 5.35
4.64 4.64 4.63 4.63 4.50 4.49 4.49 4.49
1.63 1.47 1.46
f1 (ppm)

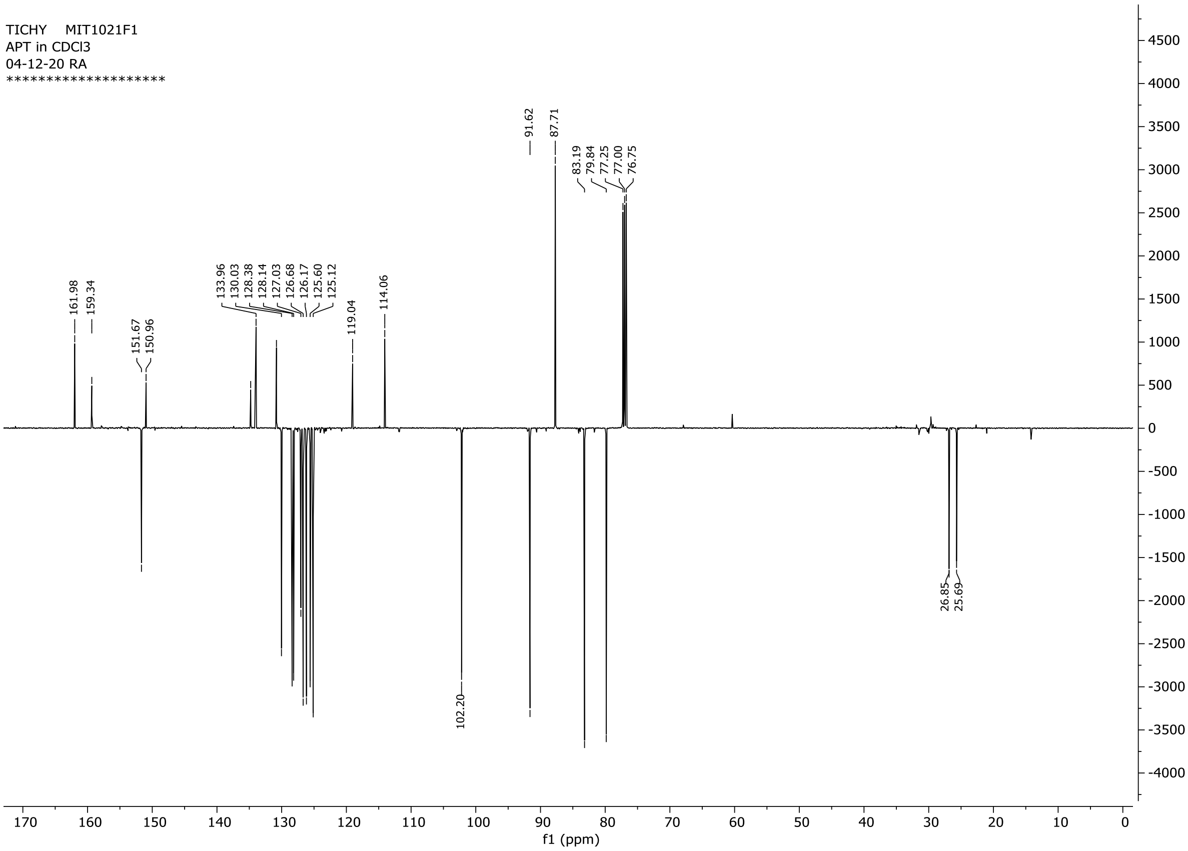
TICHY MIT1021F1
APT in CDCl3
04-12-20 RA
****
161.98 159.34
151.67 150.96
133.96 130.03 128.38 128.14 127.03 126.68 126.17 125.60 125.12
119.04
114.06
102.20
91.62 87.71
83.19 79.84 77.25 77.00 76.75
26.85 25.69
f1 (ppm)

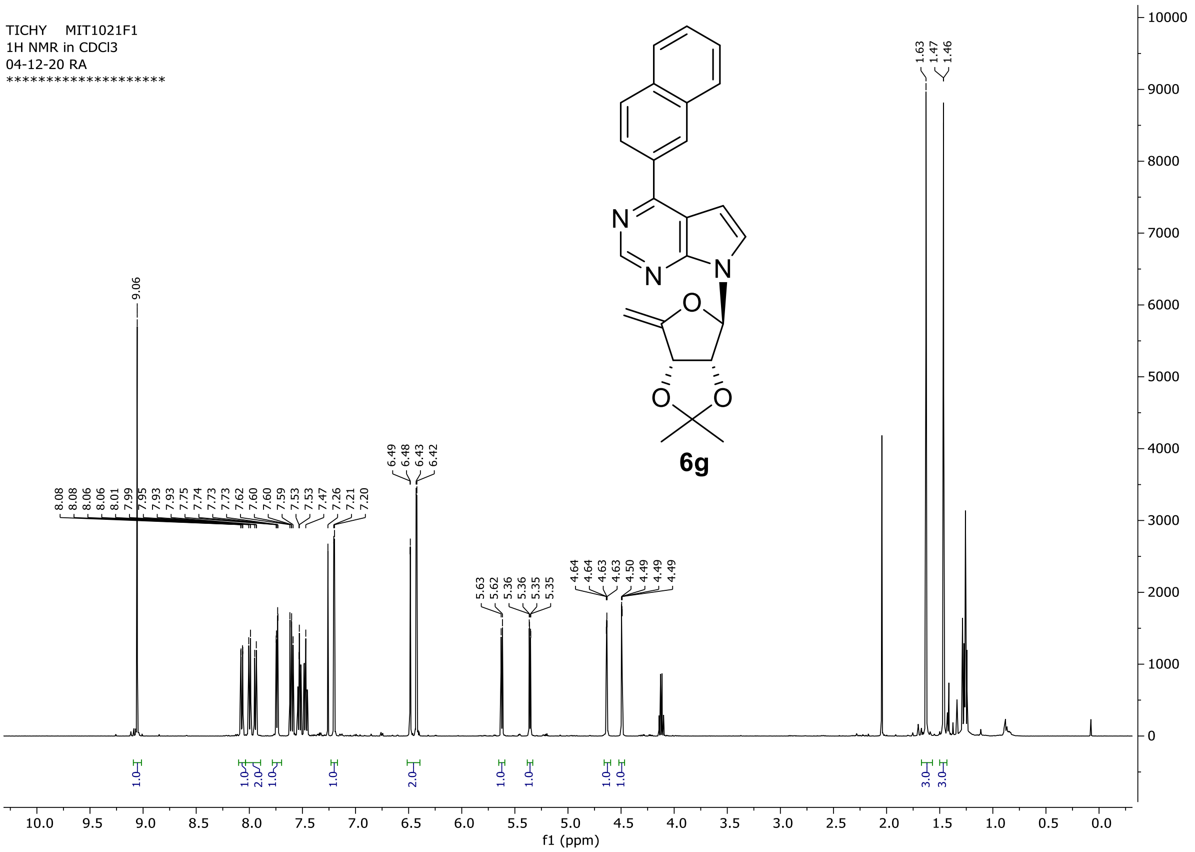
TICHY MIT1021F1
1H NMR in CDCl3
04-12-20 RA
****
6g
f1 (ppm)

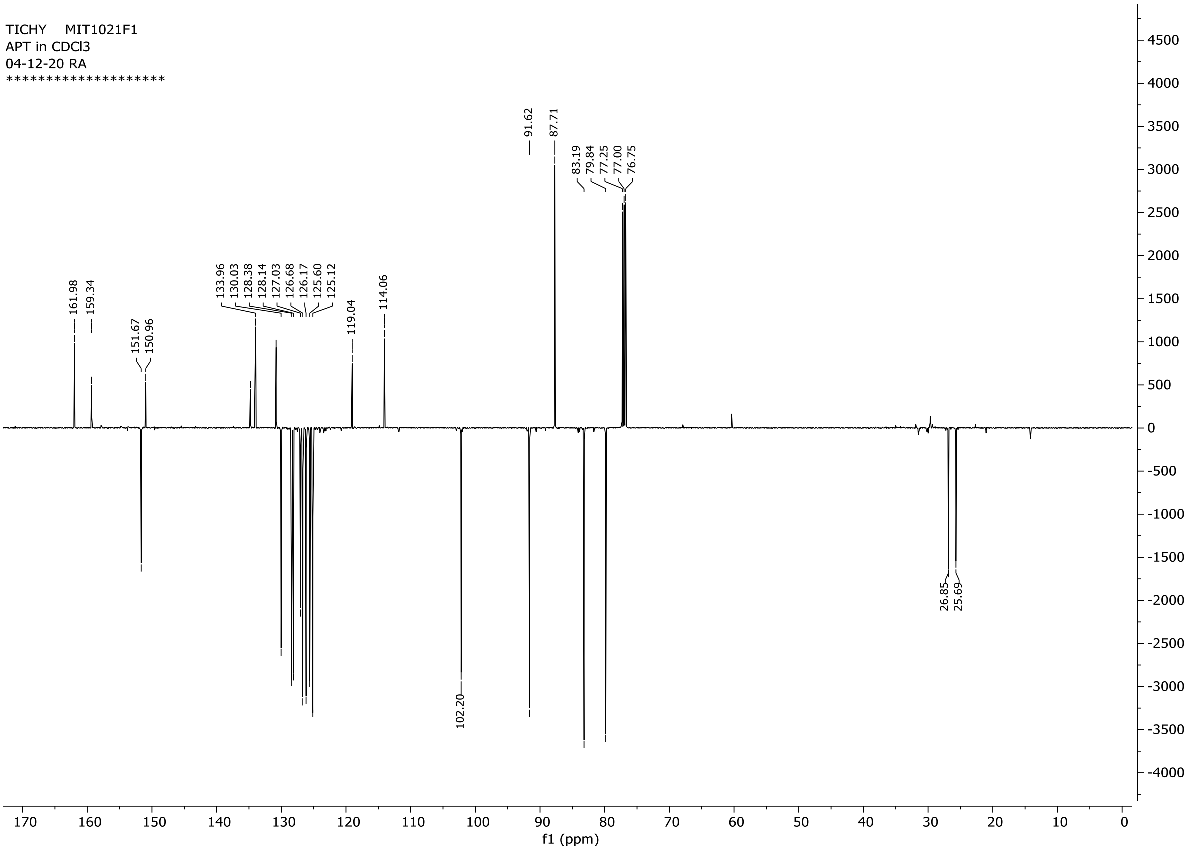
TICHY MIT1021F1
APT in CDCl3
04-12-20 RA
****
f1 (ppm)

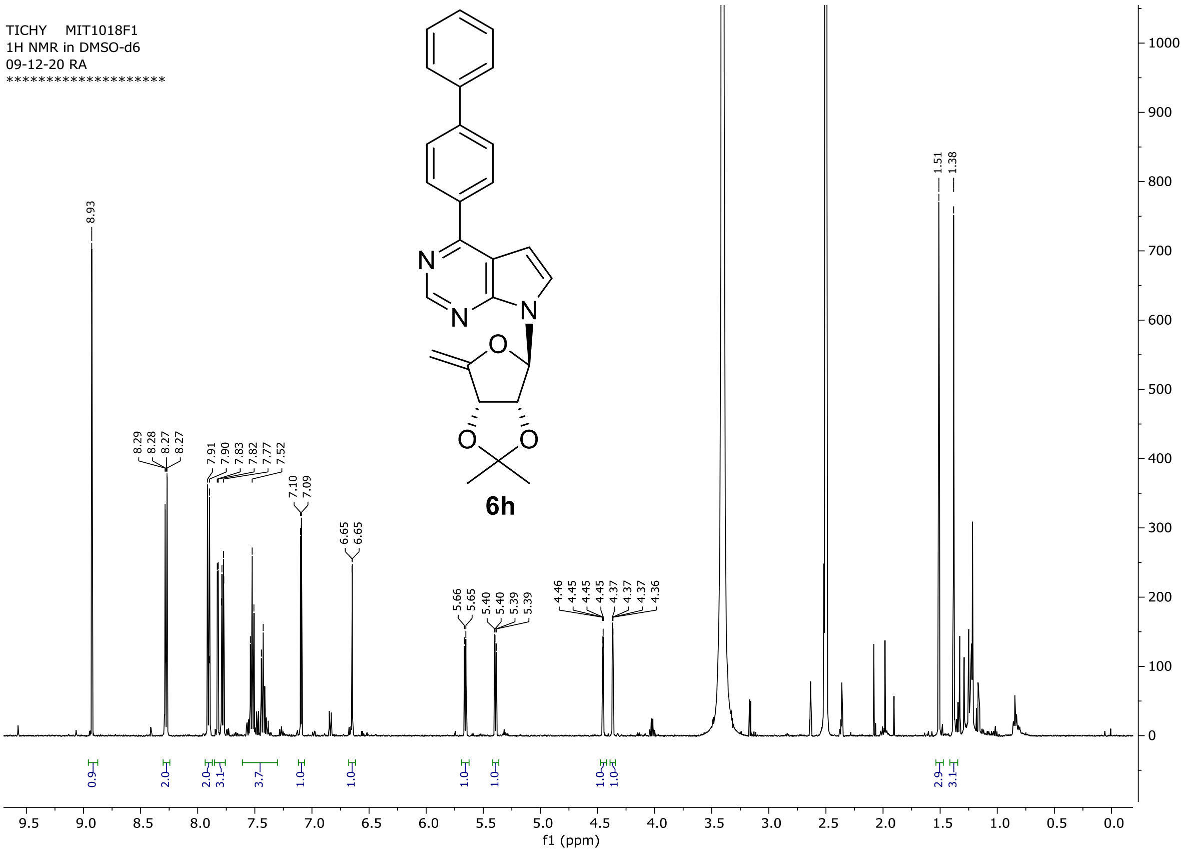
TICHY MIT1018F1
1H NMR in DMSO-d6
09-12-20 RA
****
6h
8.93
8.29 8.28 8.27 8.27
7.91 7.90 7.83 7.82 7.77 7.52
7.10 7.09
6.65 6.65
5.66 5.65 5.40 5.40 5.39 5.39
4.46 4.45 4.45 4.45 4.37 4.37 4.37 4.36
1.51 1.38
0.9 2.0 2.0 3.1 3.7 1.0 1.0 1.0 1.0 1.0 1.0 2.9 3.1
f1 (ppm)

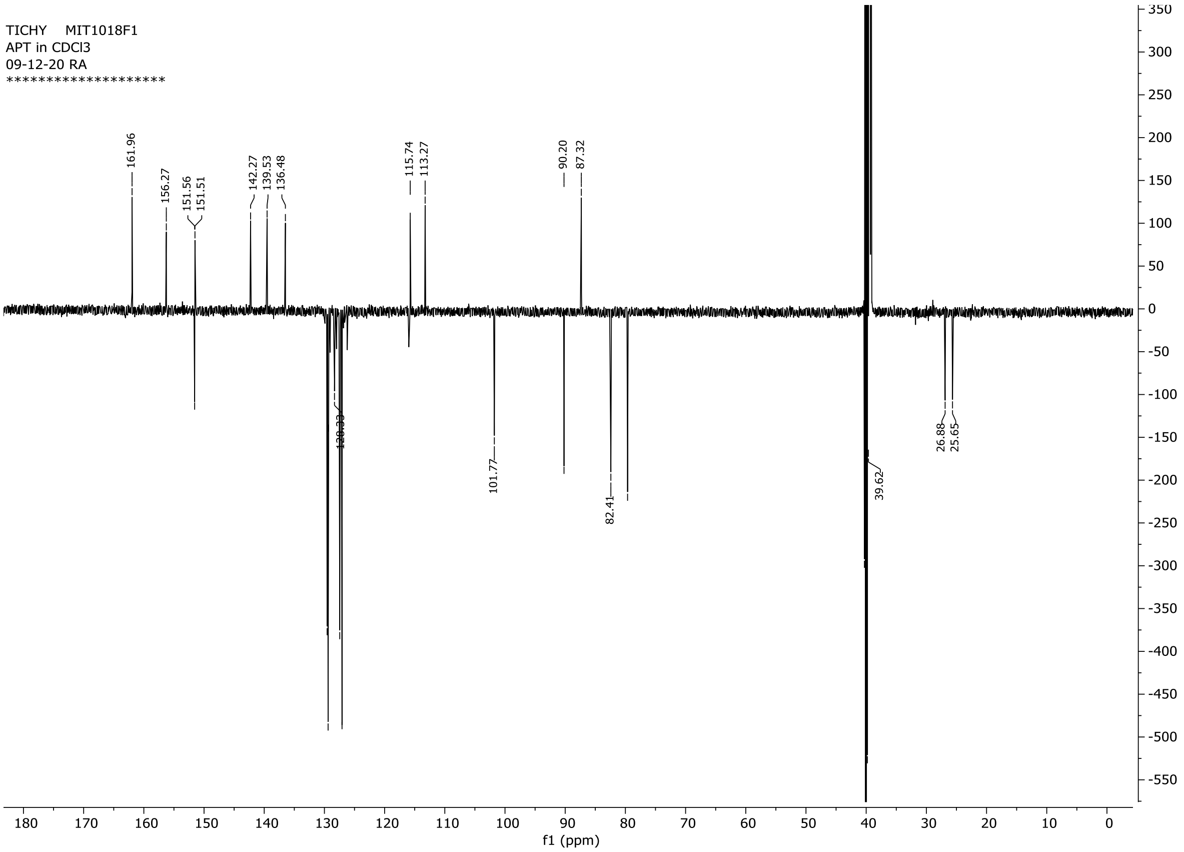
TICHY MIT1018F1
APT in CDCl3
09-12-20 RA
****
161.96
156.27
151.56 151.51
142.27 139.53 136.48
115.74 113.27
90.20 87.32
130.33
101.77
82.41
39.62
26.88 25.65
f1 (ppm)

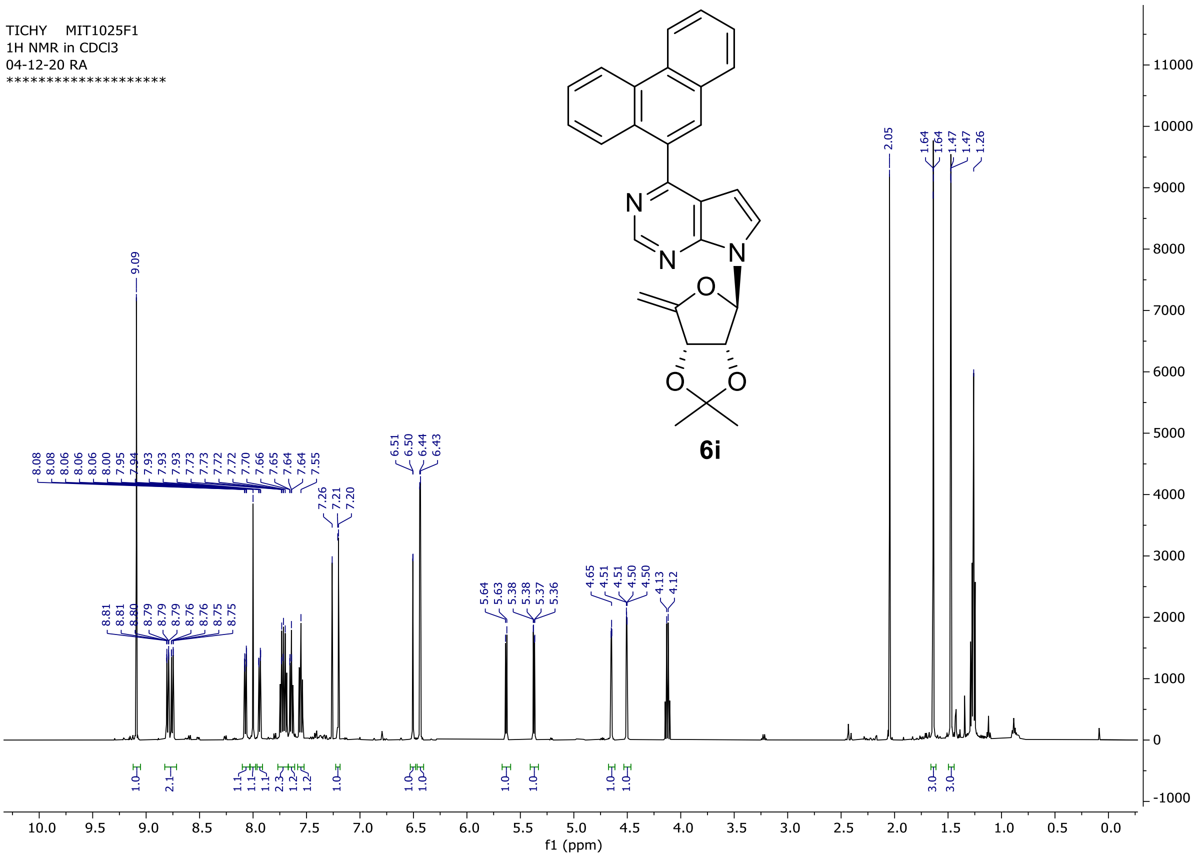
TICHY MIT1025F1
1H NMR in CDCl3
04-12-20 RA
****
6i
f1 (ppm)

TICHY MIT1025F1
APT in CDCl3
04-12-20 RA
****

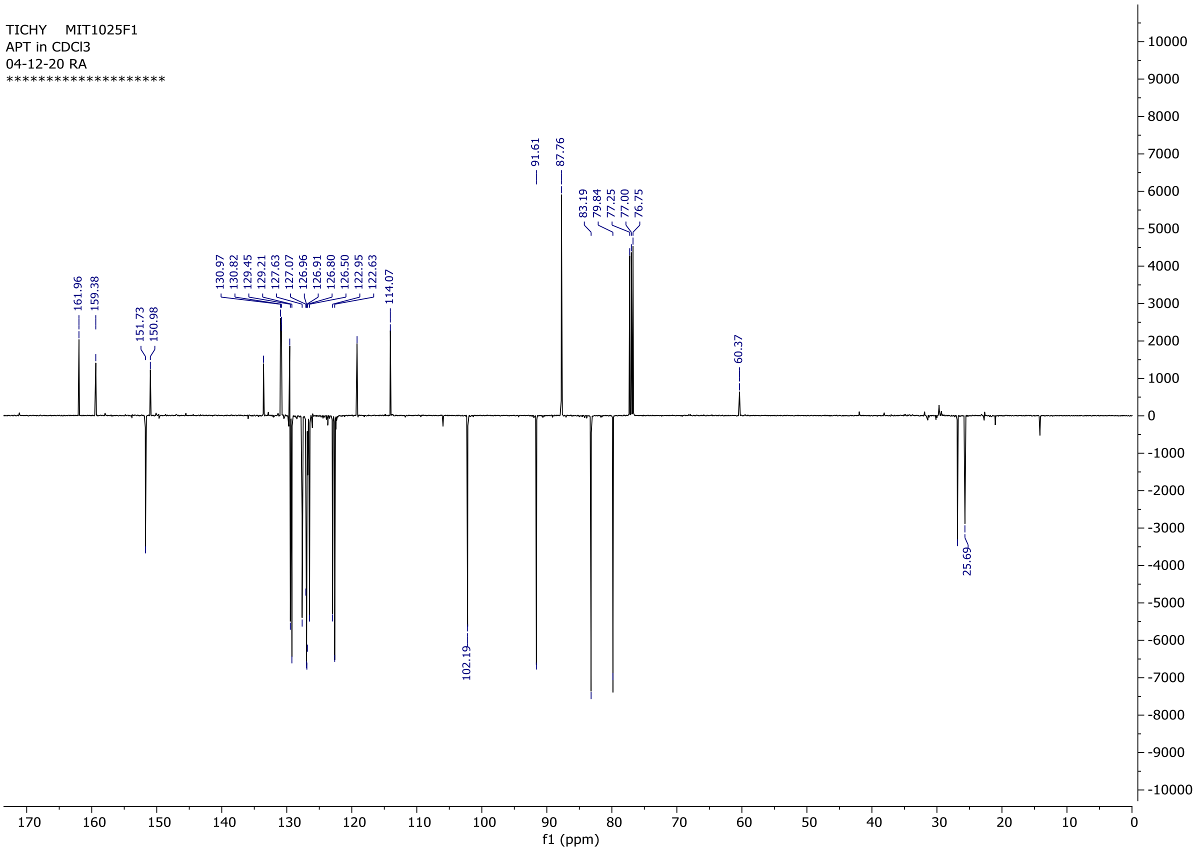

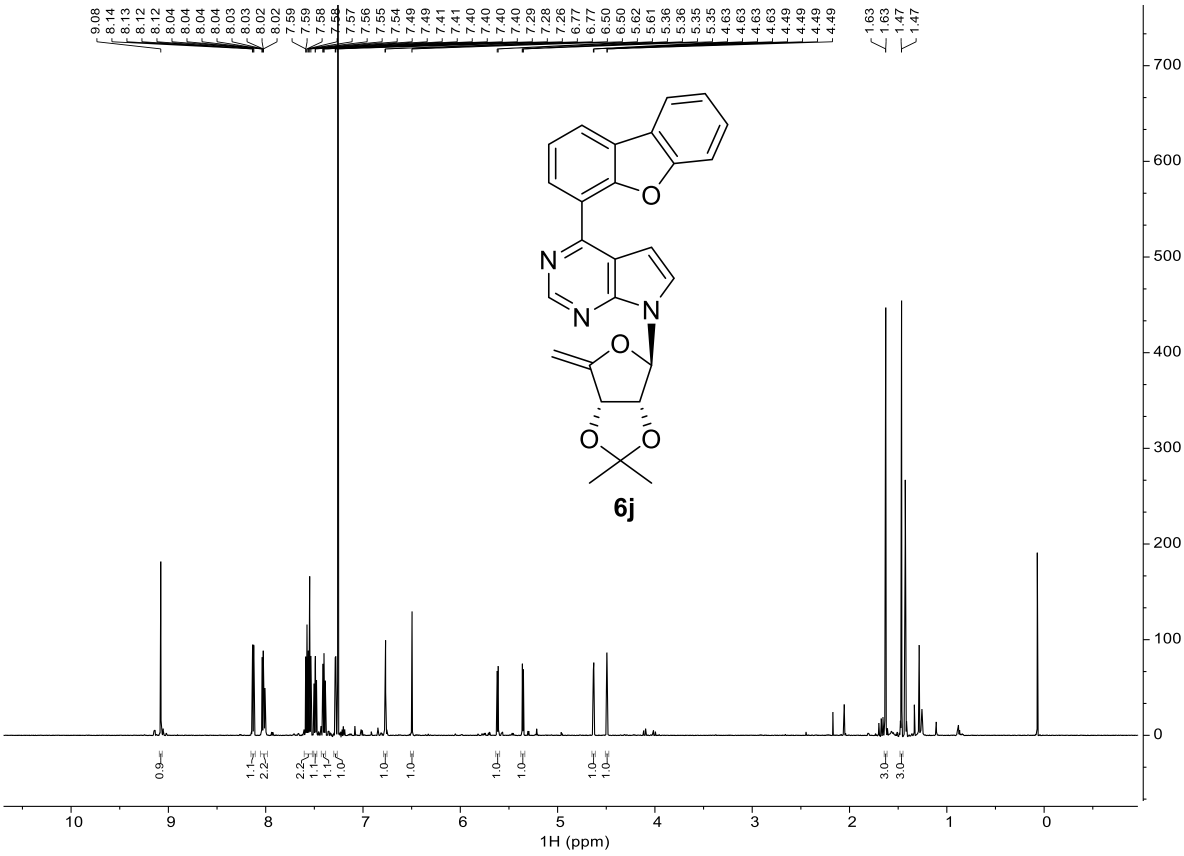

6j

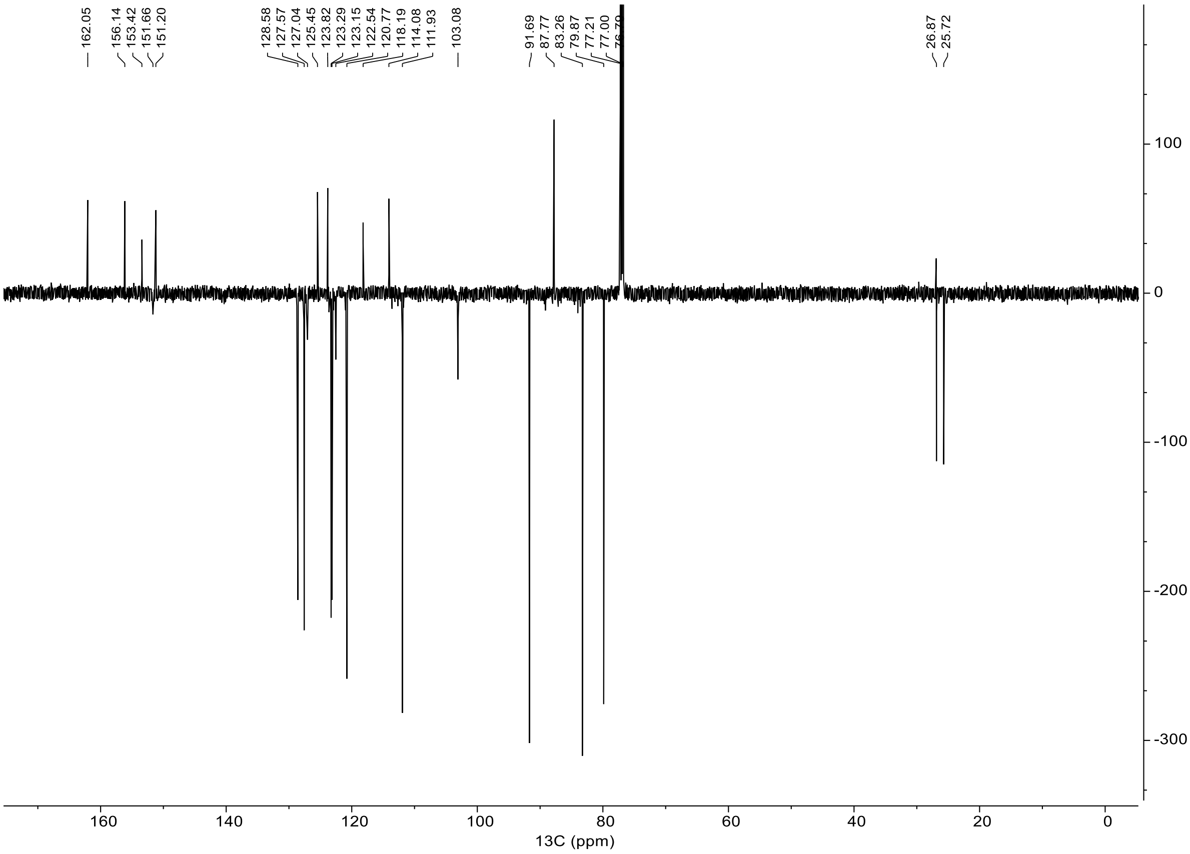

VESELOVSKA LV630F1
1H NMR in DMSO-d6
12-11-20 RA
****

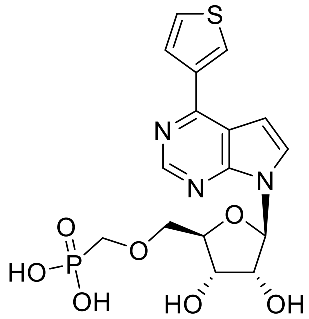

7a

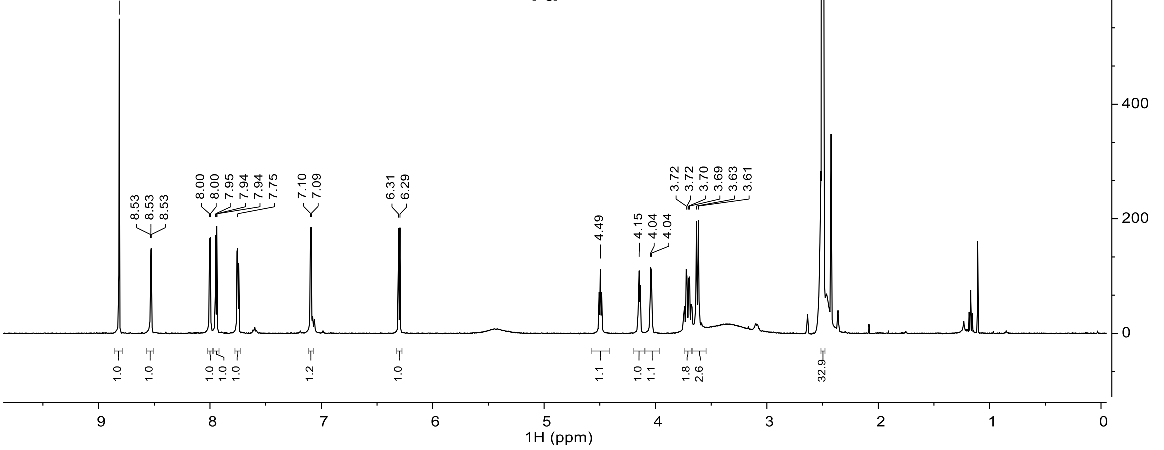

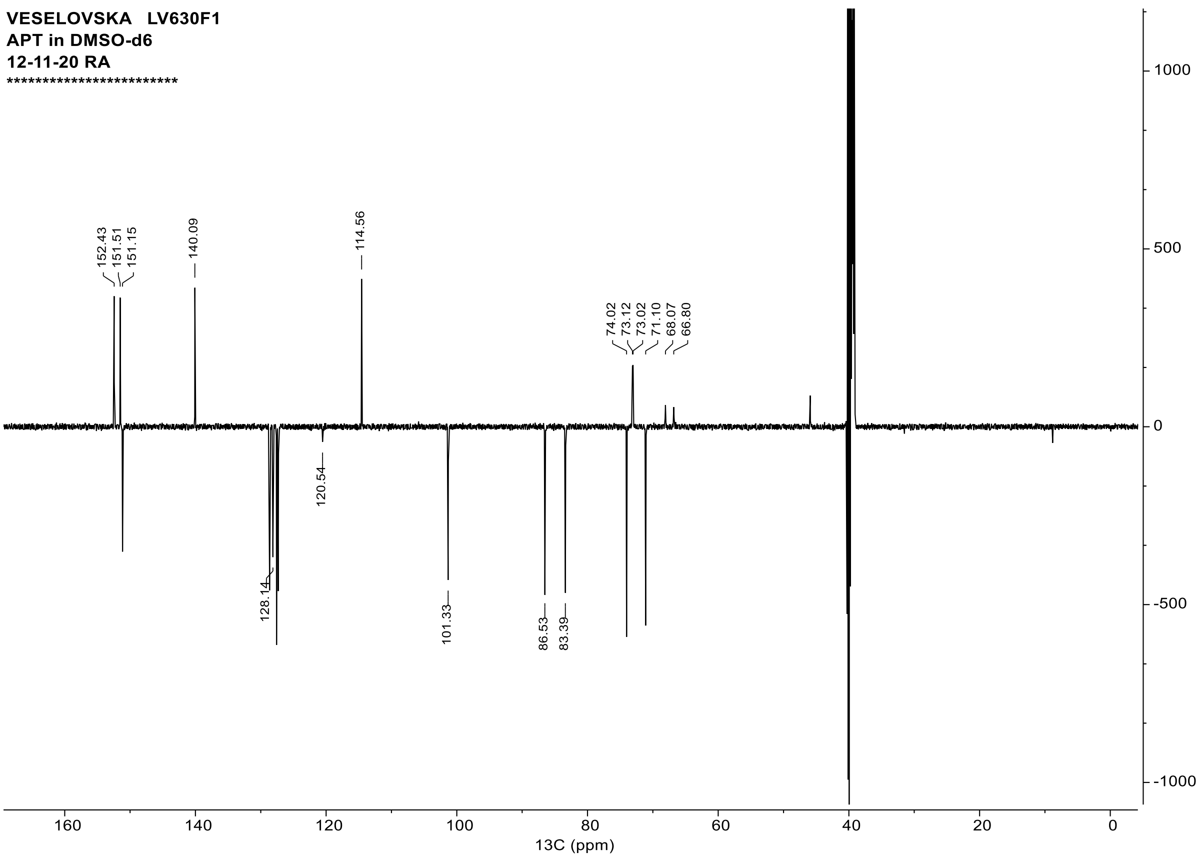

VESELOVSKA LV630F1
APT in DMSO-d6
12-11-20 RA
****
152.43
151.51
151.15
140.09
114.56
120.54
128.14
101.33
86.53
83.39
74.02
73.12
73.02
71.10
68.07
66.80
13C (ppm)

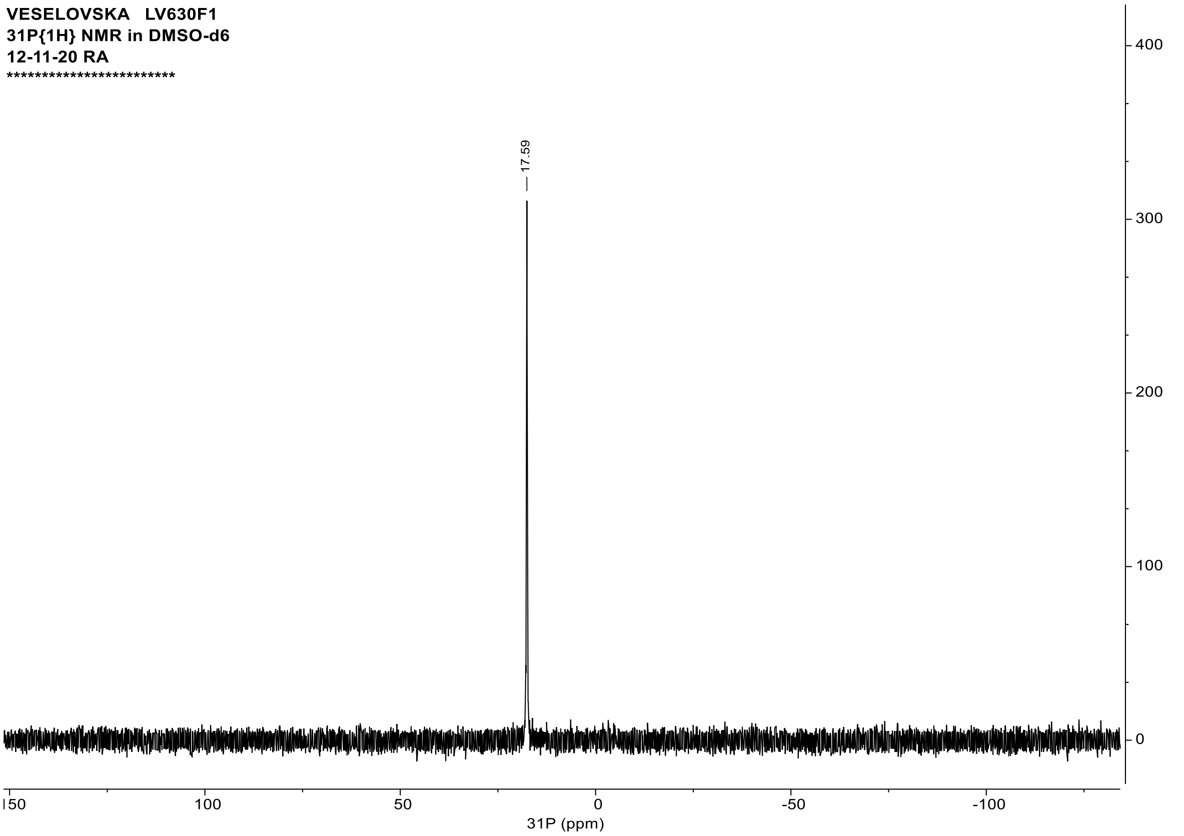

VESELOVSKA LV630F1
31P{1H} NMR in DMSO-d6
12-11-20 RA
****
17.59
31P (ppm)

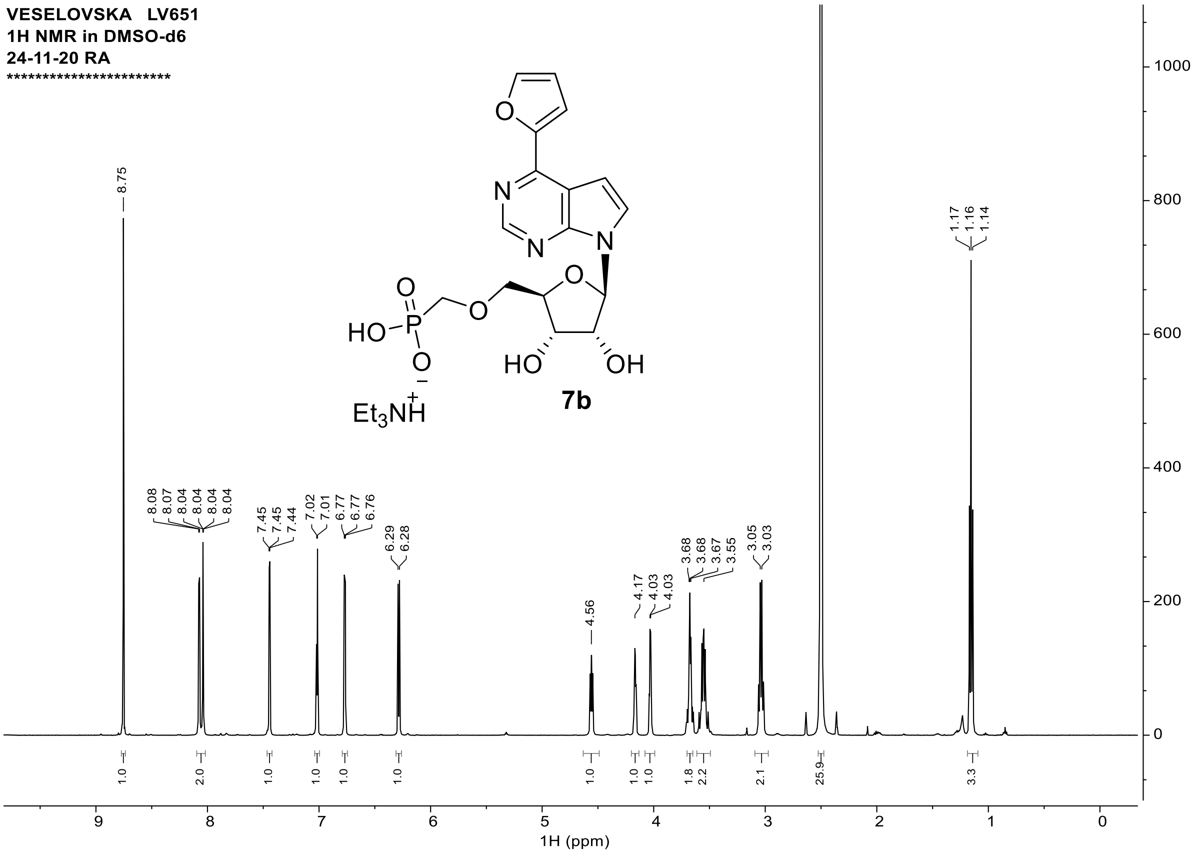
VESELOVSKA LV651
1H NMR in DMSO-d6
24-11-20 RA
****
7b
8.75
8.08 8.07 8.04 8.04 8.04 8.04
7.45 7.45 7.44
7.02 7.01
6.77 6.77 6.76
6.29 6.28
4.56
4.17 4.03 4.03
3.68 3.68 3.67 3.55
3.05 3.03
1.17 1.16 1.14
1.0 2.0 1.0 1.0 1.0 1.0 1.0 1.0 1.0 1.8 2.2 2.1 25.9 3.3
1H (ppm)

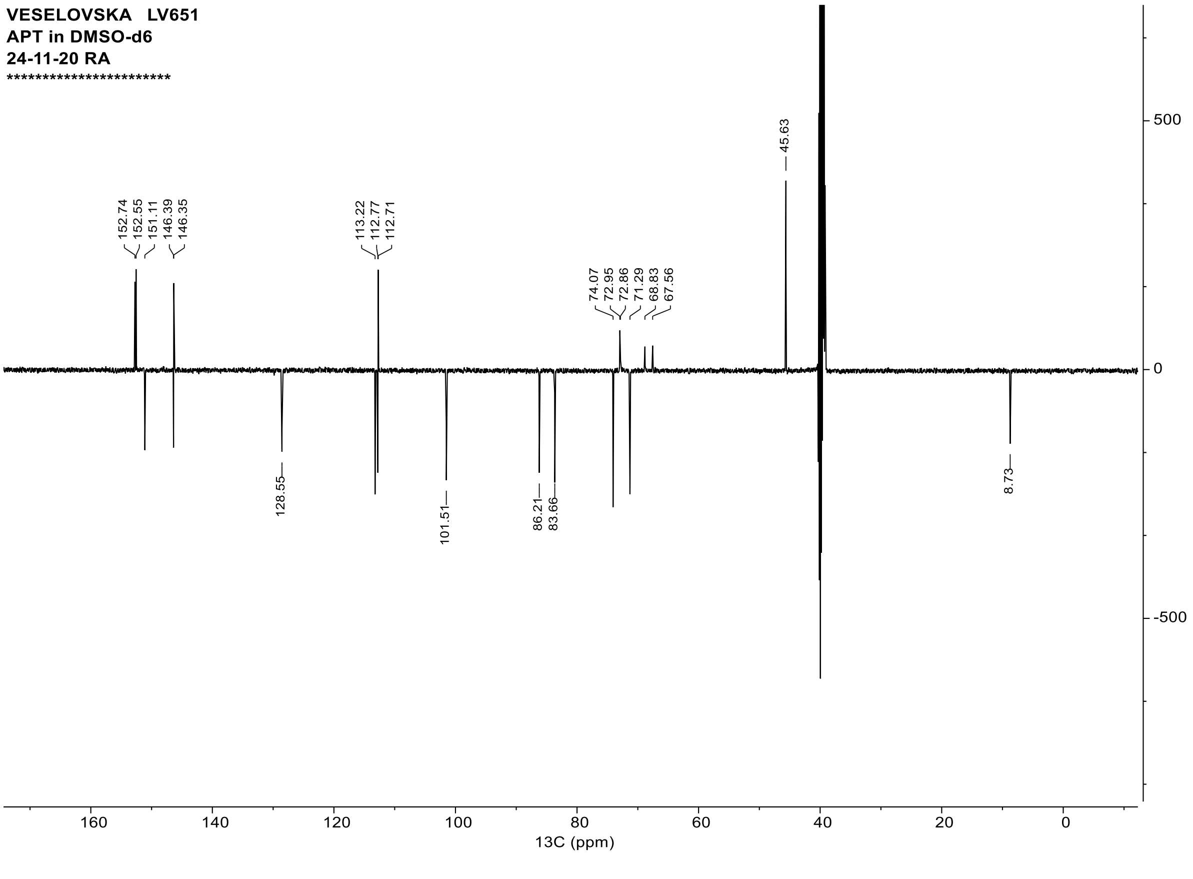
VESELOVSKA LV651
APT in DMSO-d6
24-11-20 RA
****
152.74 152.55 151.11 146.39 146.35
128.55
113.22 112.77 112.71
101.51
86.21 83.66
74.07 72.95 72.86 71.29 68.83 67.56
45.63
8.73
13C (ppm)

VESELOVSKA LV651
31P{1H} NMR in DMSO-d6
24-11-20 RA
****

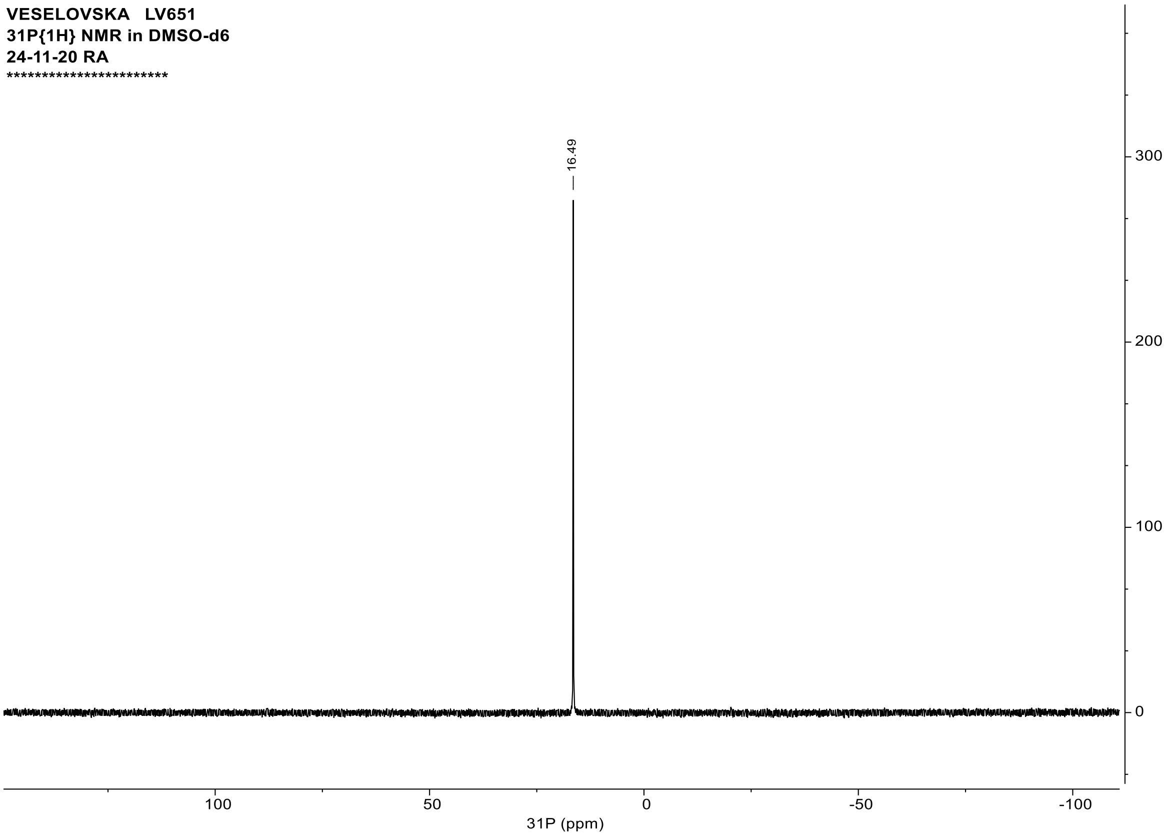

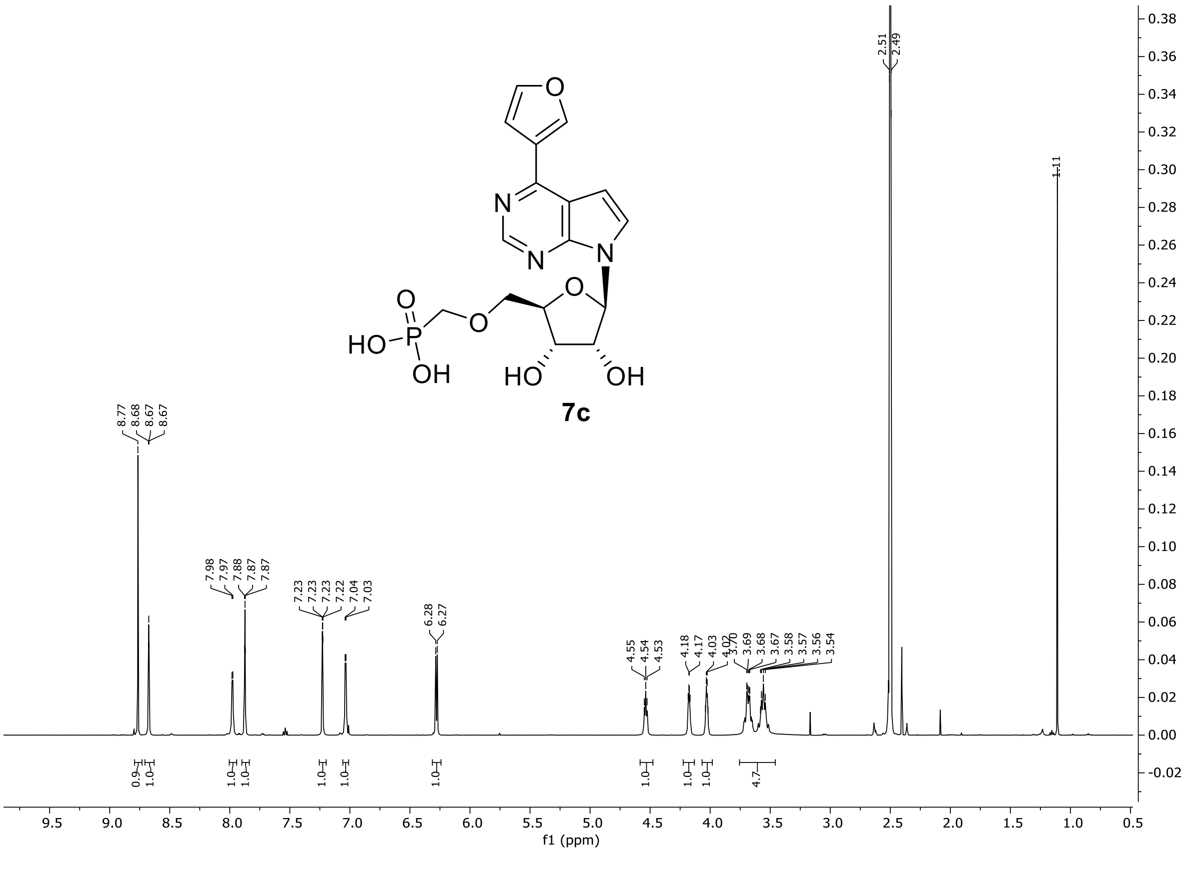

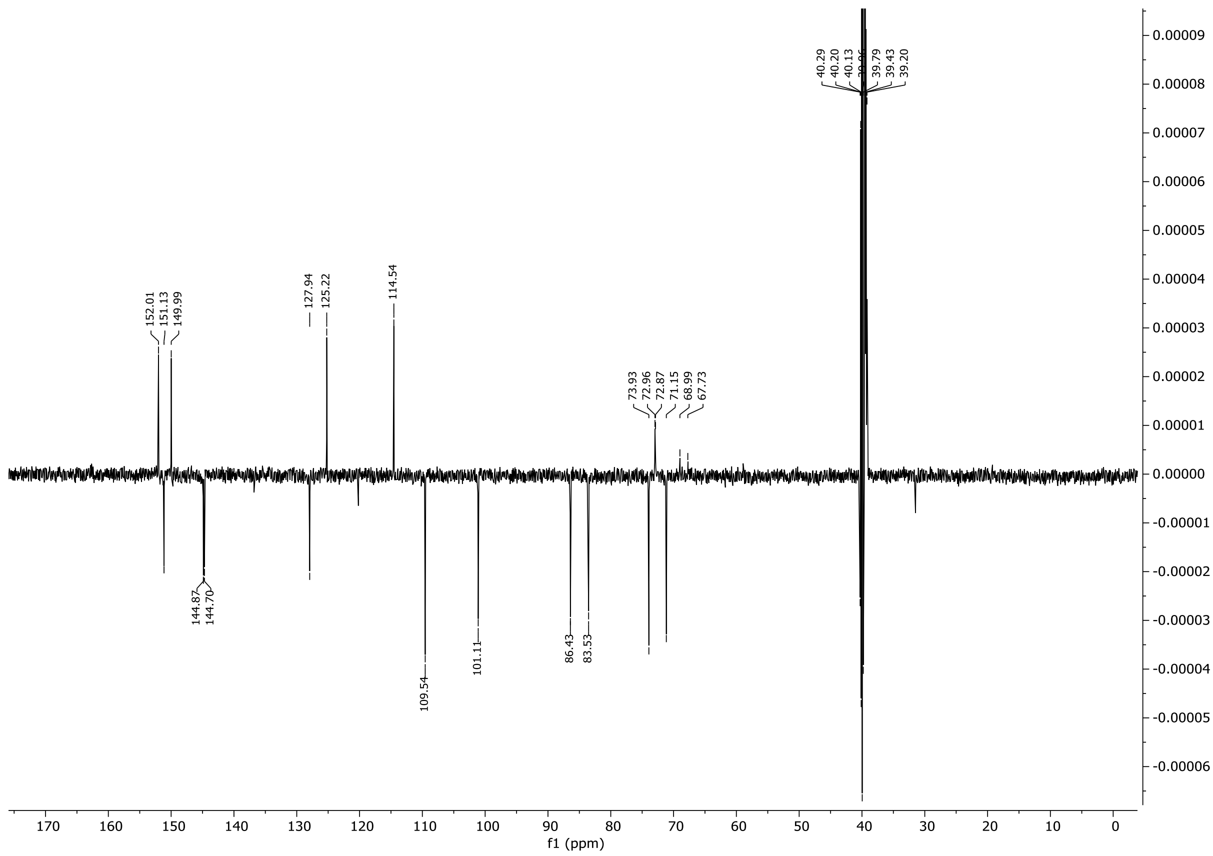

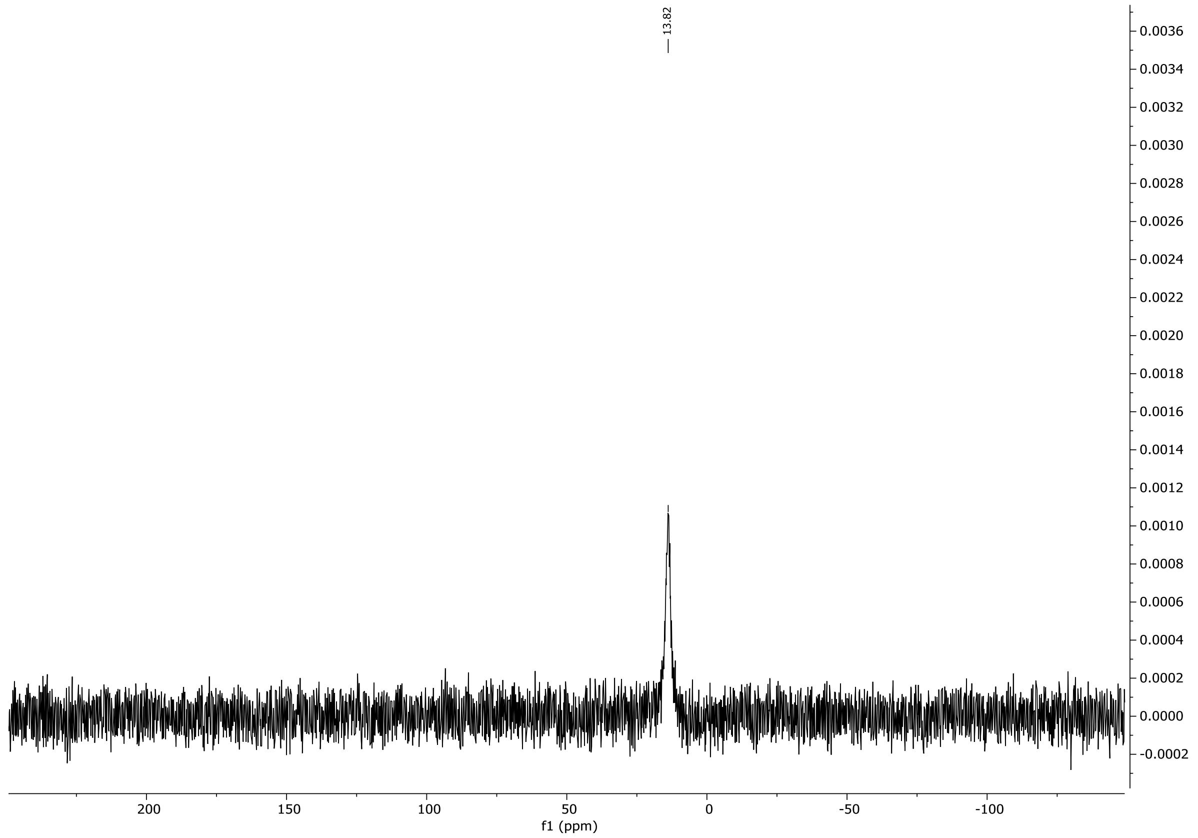

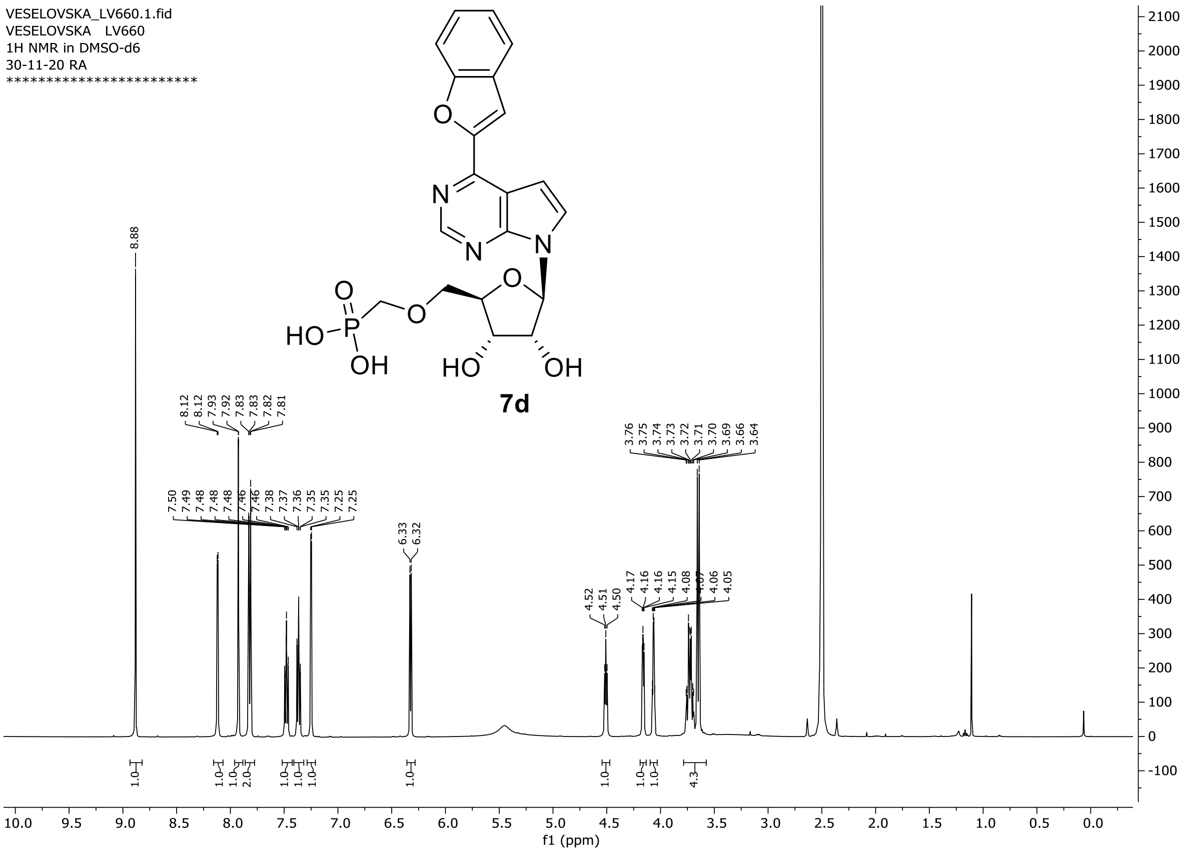
VESELOVSKA_LV660.1.fid
VESELOVSKA LV660
1H NMR in DMSO-d6
30-11-20 RA
7d
f1 (ppm)

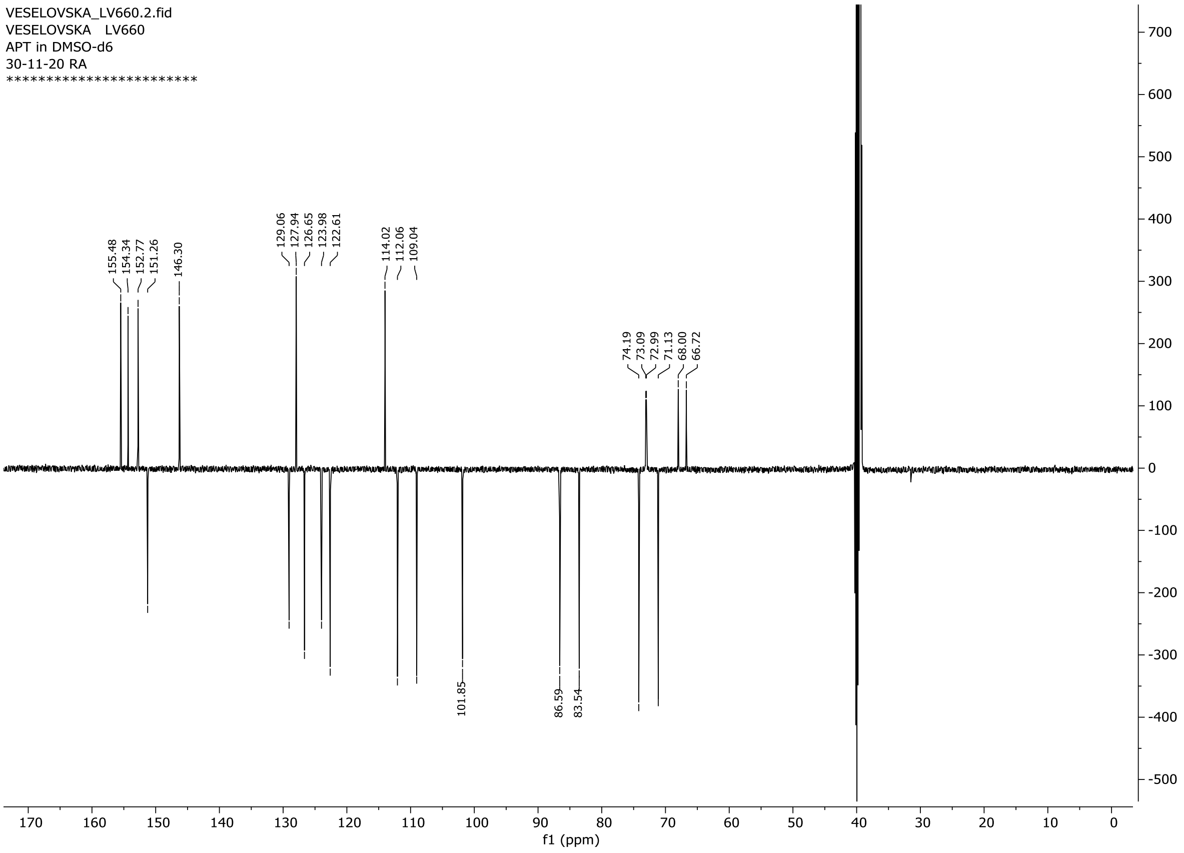
VESELOVSKA_LV660.2.fid
VESELOVSKA LV660
APT in DMSO-d6
30-11-20 RA
f1 (ppm)

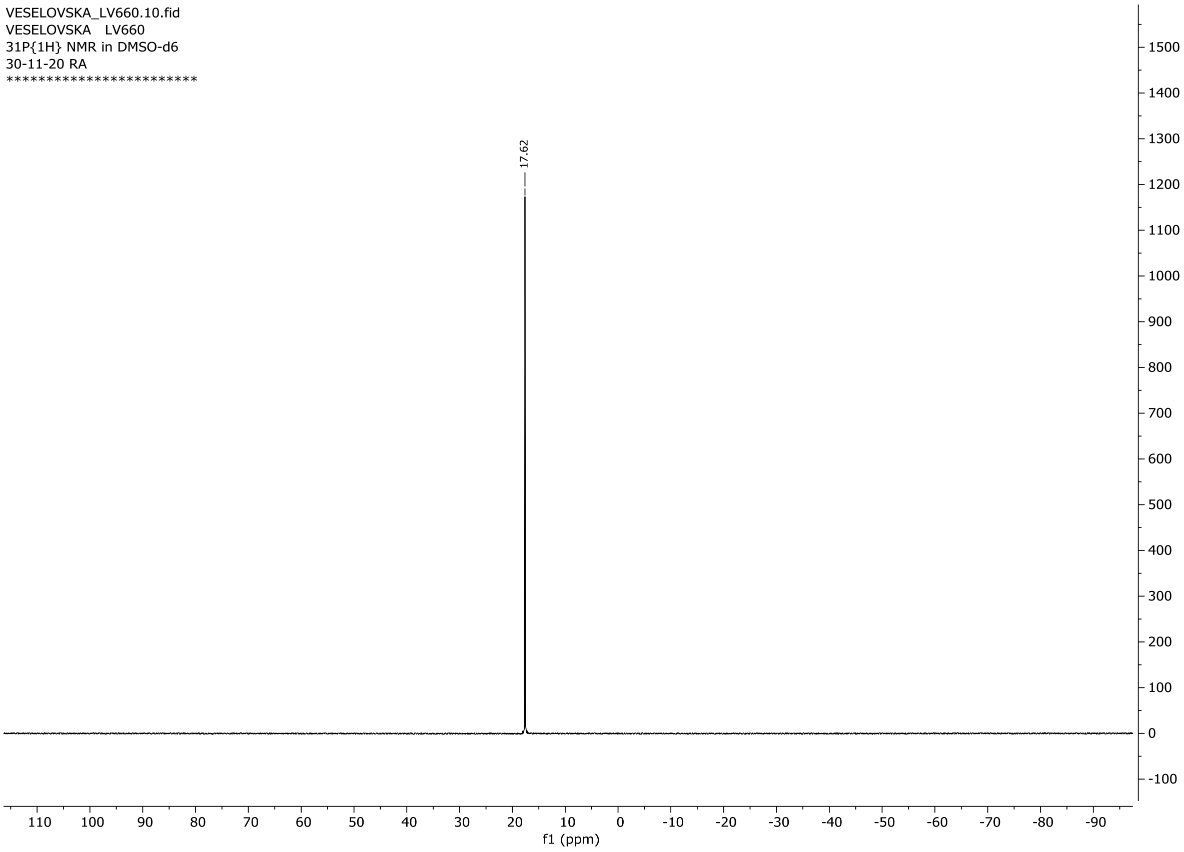
VESELOVSKA_LV660.10.fid
VESELOVSKA LV660
31P{1H} NMR in DMSO-d6
30-11-20 RA
****
17.62
f1 (ppm)

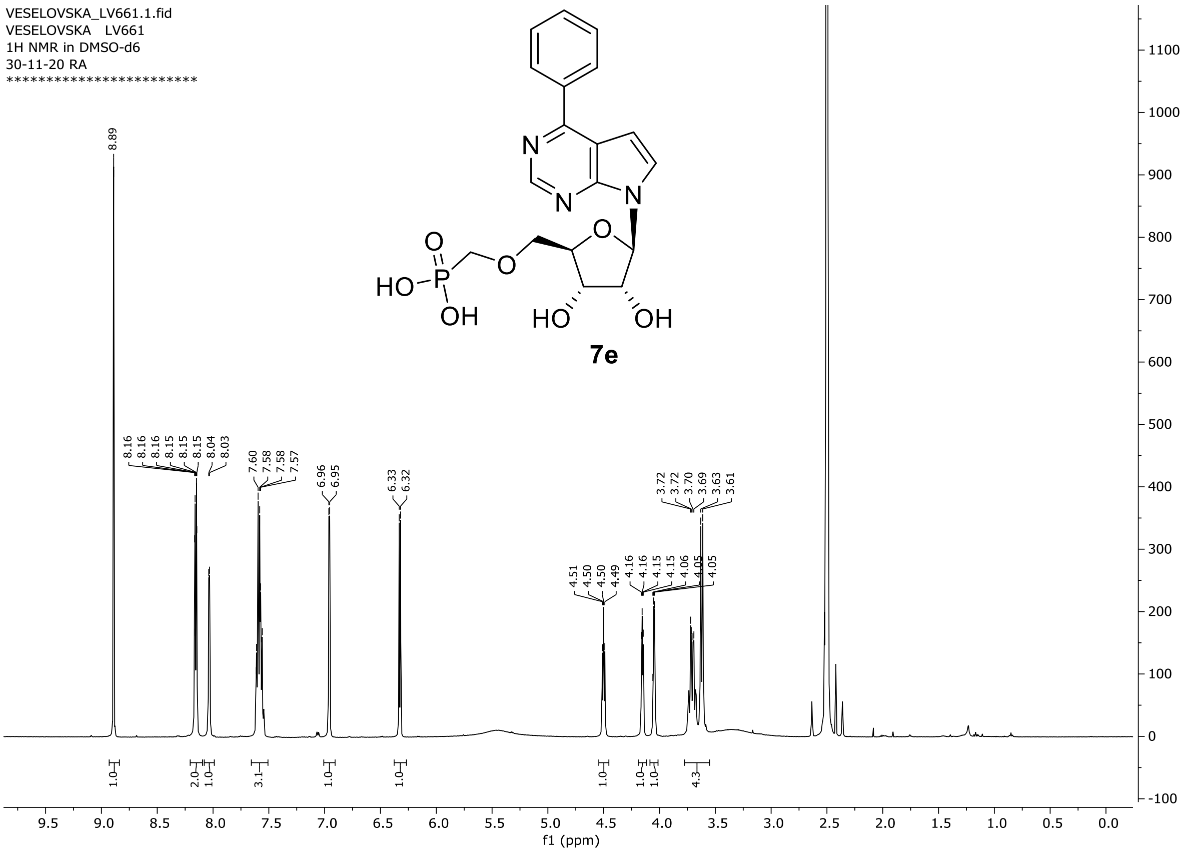
VESELOVSKA_LV661.1.fid
VESELOVSKA LV661
1H NMR in DMSO-d6
30-11-20 RA
****
7e
f1 (ppm)

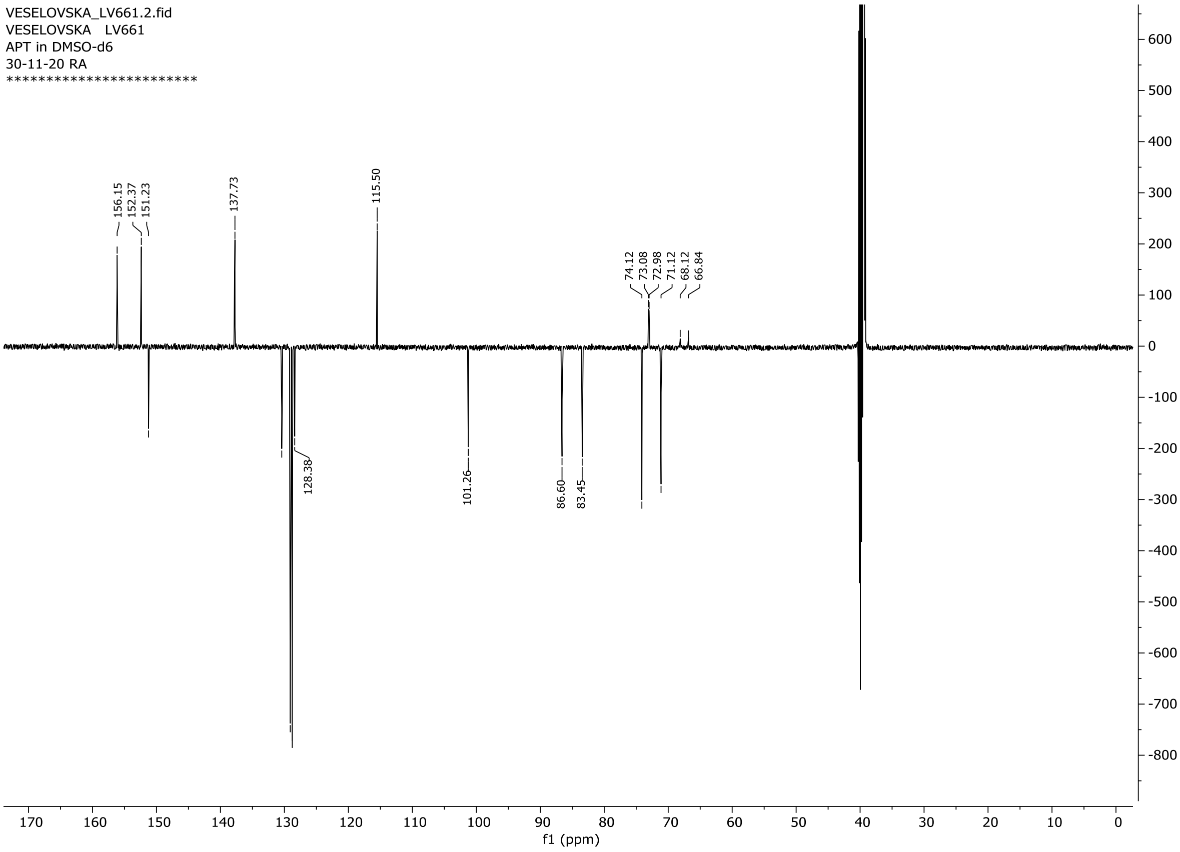
VESELOVSKA_LV661.2.fid
VESELOVSKA LV661
APT in DMSO-d6
30-11-20 RA
****
156.15
152.37
151.23
137.73
115.50
74.12
73.08
72.98
71.12
68.12
66.84
128.38
101.26
86.60
83.45
f1 (ppm)

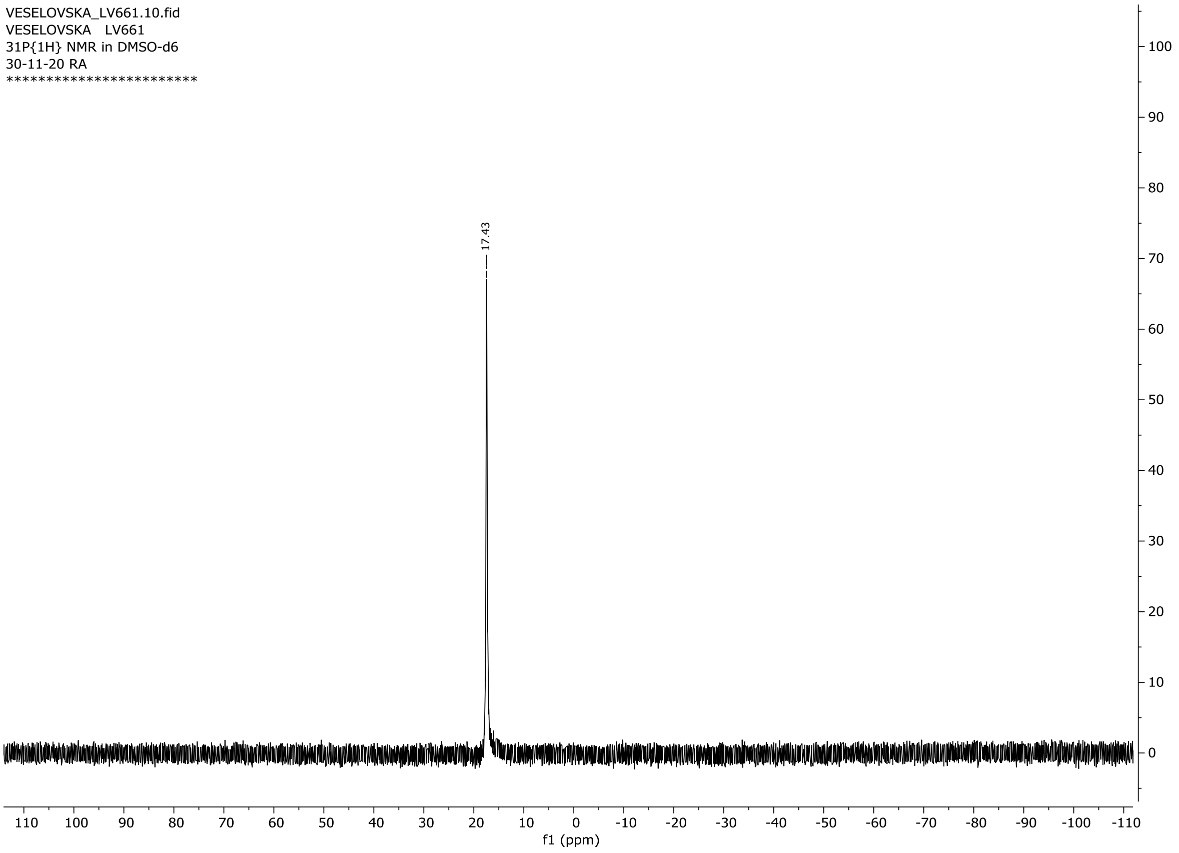
VESELOVSKA_LV661.10.fid
VESELOVSKA LV661
31P{1H} NMR in DMSO-d6
30-11-20 RA
****
17.43
f1 (ppm)

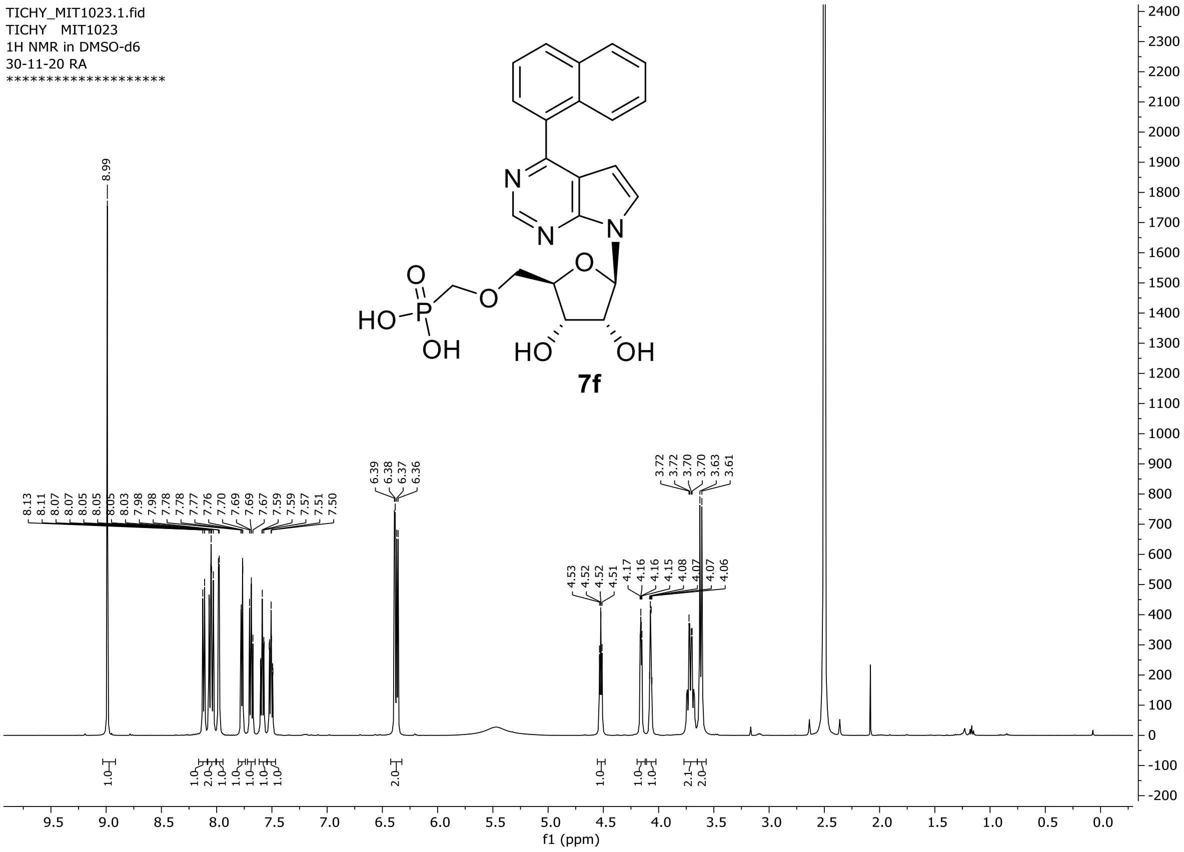
TICHY_MIT1023.1.fid
TICHY MIT1023
1H NMR in DMSO-d6
30-11-20 RA
****
7f
f1 (ppm)

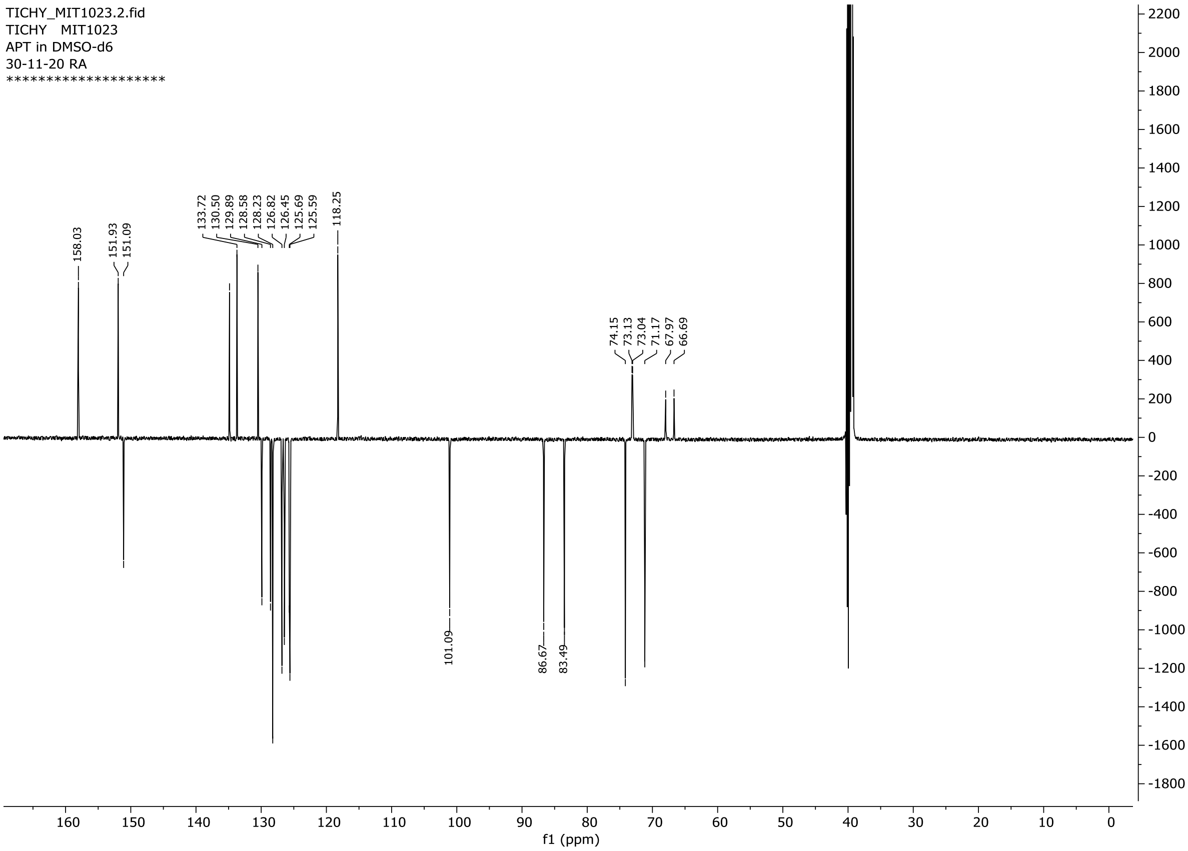
TICHY_MIT1023.2.fid
TICHY MIT1023
APT in DMSO-d6
30-11-20 RA
****
f1 (ppm)

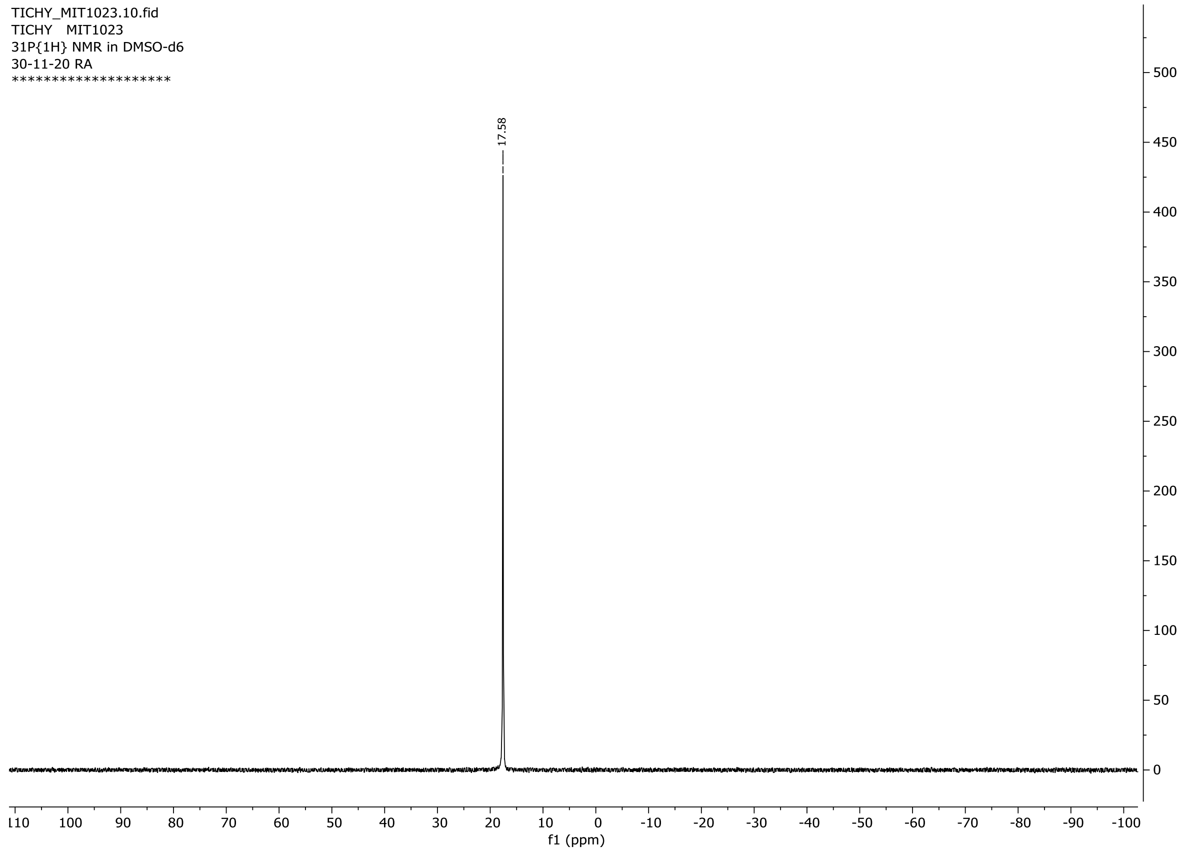
TICHY_MIT1023.10.fid
TICHY MIT1023
31P{1H} NMR in DMSO-d6
30-11-20 RA
17.58
f1 (ppm)

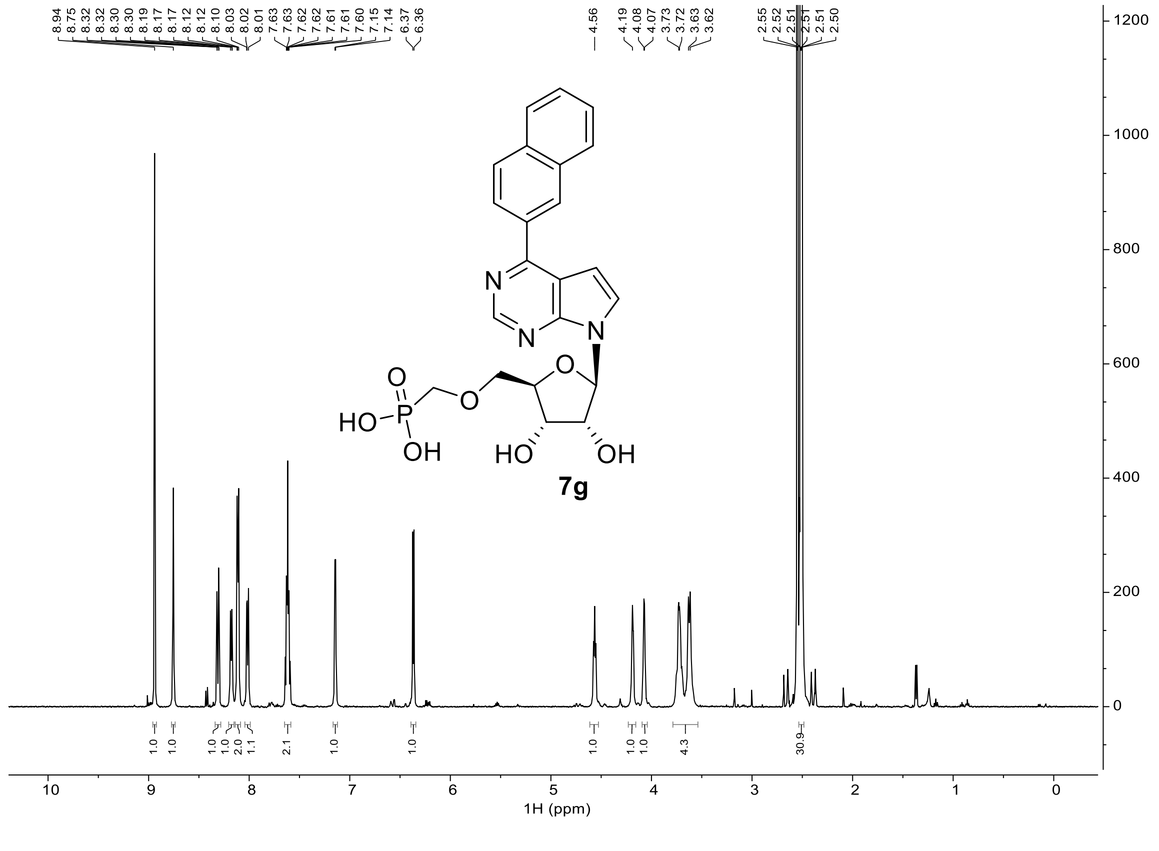
8.94
8.75
8.32
8.32
8.30
8.30
8.19
8.17
8.17
8.12
8.12
8.10
8.03
8.02
8.01
7.63
7.63
7.62
7.62
7.61
7.61
7.60
7.15
7.14
6.37
6.36
4.56
4.19
4.08
4.07
3.73
3.72
3.63
3.62
2.55
2.52
2.51
2.51
2.51
2.50
HO
OH
7g
1H (ppm)

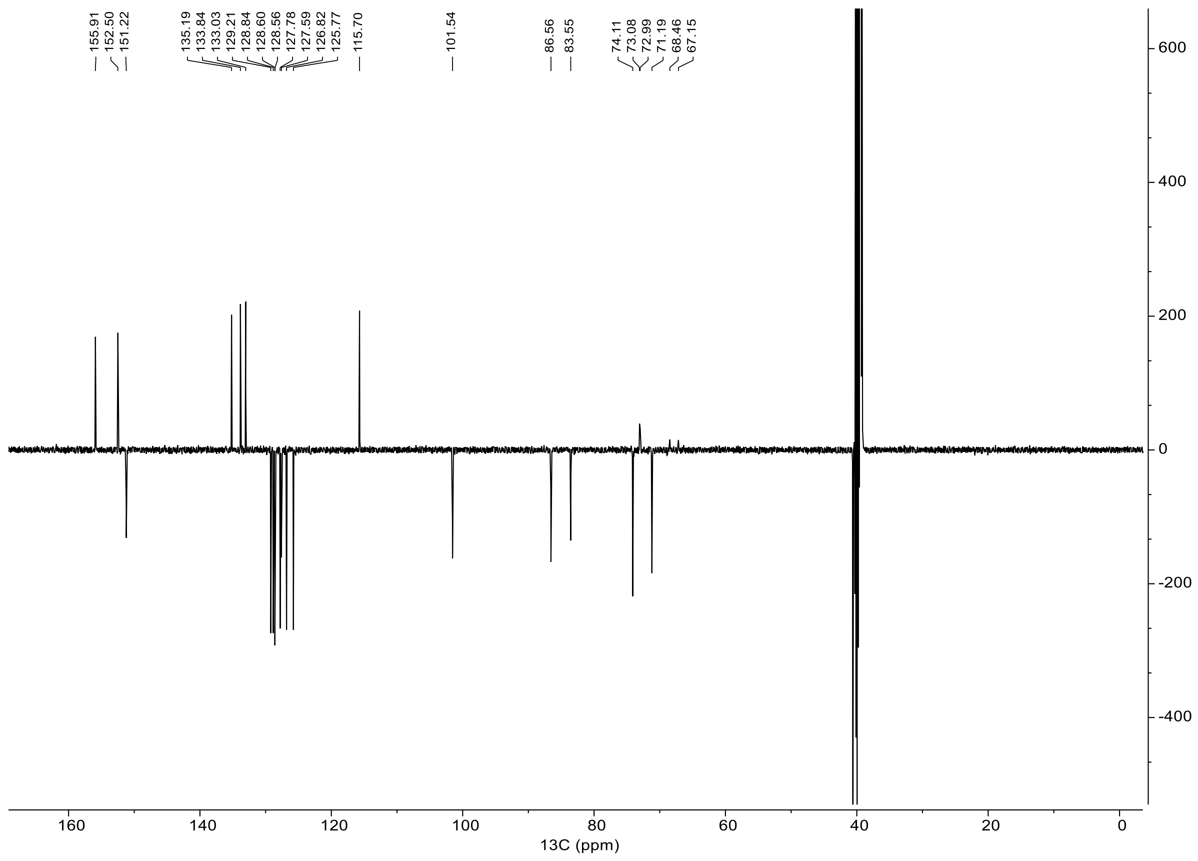
155.91
152.50
151.22
135.19
133.84
133.03
129.21
128.84
128.60
128.56
127.78
127.59
126.82
125.77
115.70
101.54
86.56
83.55
74.11
73.08
72.99
71.19
68.46
67.15
13C (ppm)

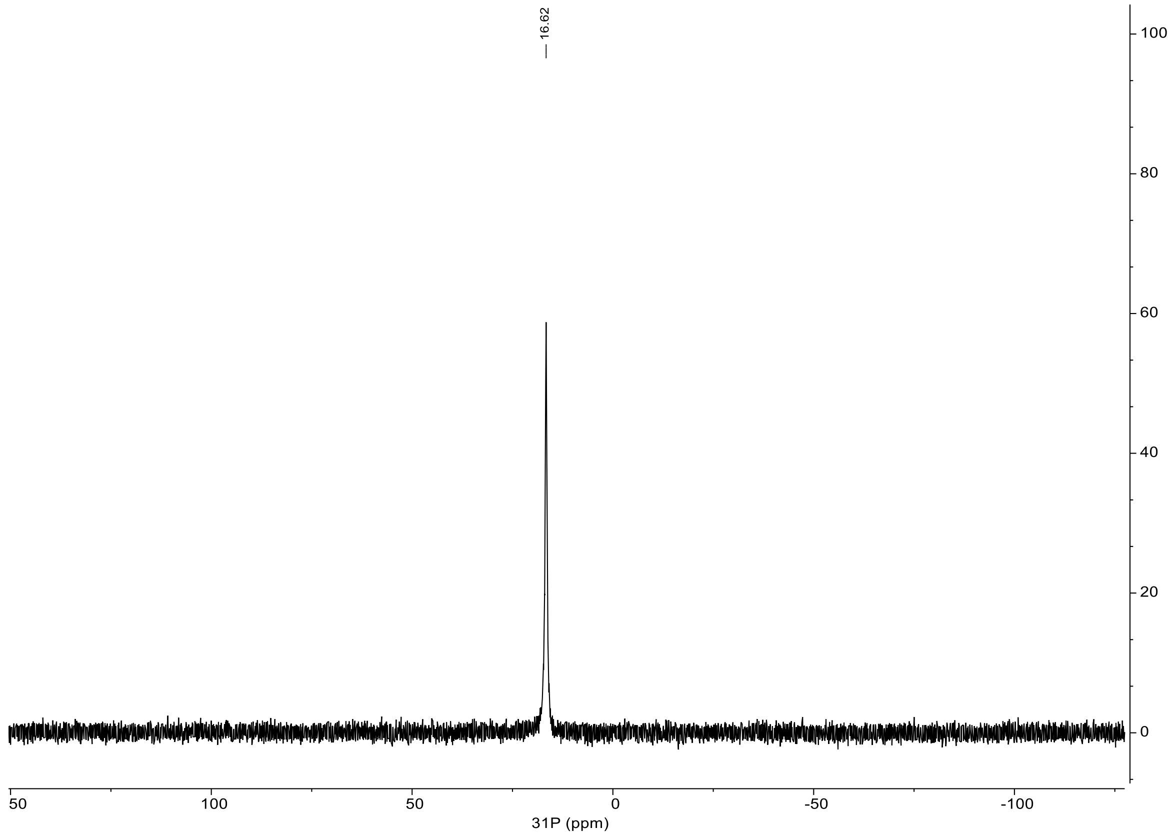
16.62
31P (ppm)

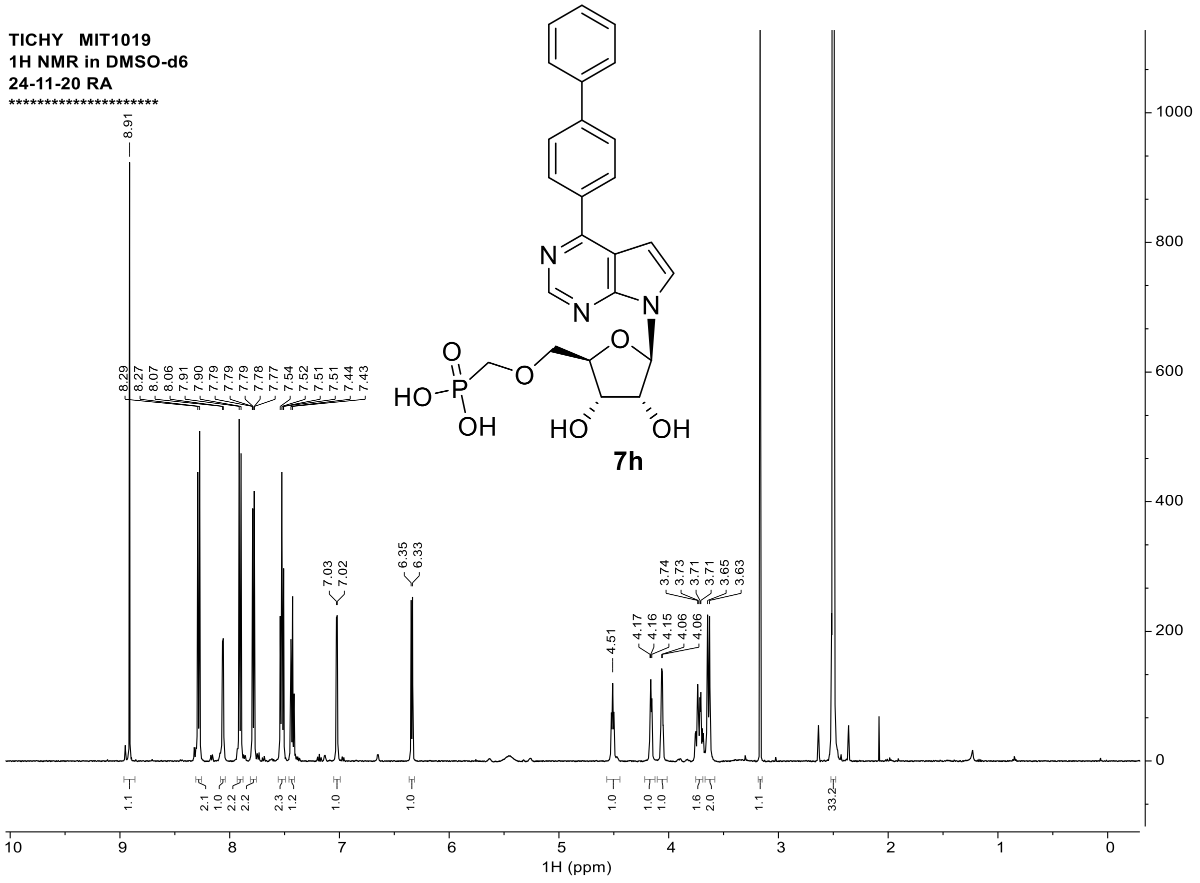
TICHY MIT1019
1H NMR in DMSO-d6
24-11-20 RA
7h
HO
OH
O
N
8.91
8.29
8.27
8.07
8.06
7.91
7.90
7.79
7.79
7.79
7.78
7.77
7.54
7.52
7.51
7.51
7.44
7.43
7.03
7.02
6.35
6.33
4.51
4.17
4.16
4.15
4.06
4.06
3.74
3.73
3.71
3.71
3.65
3.63
1.1
2.1
1.0
2.2
2.2
2.3
1.2
1.0
1.0
1.0
1.0
1.0
1.6
2.0
1.1
33.2
1H (ppm)

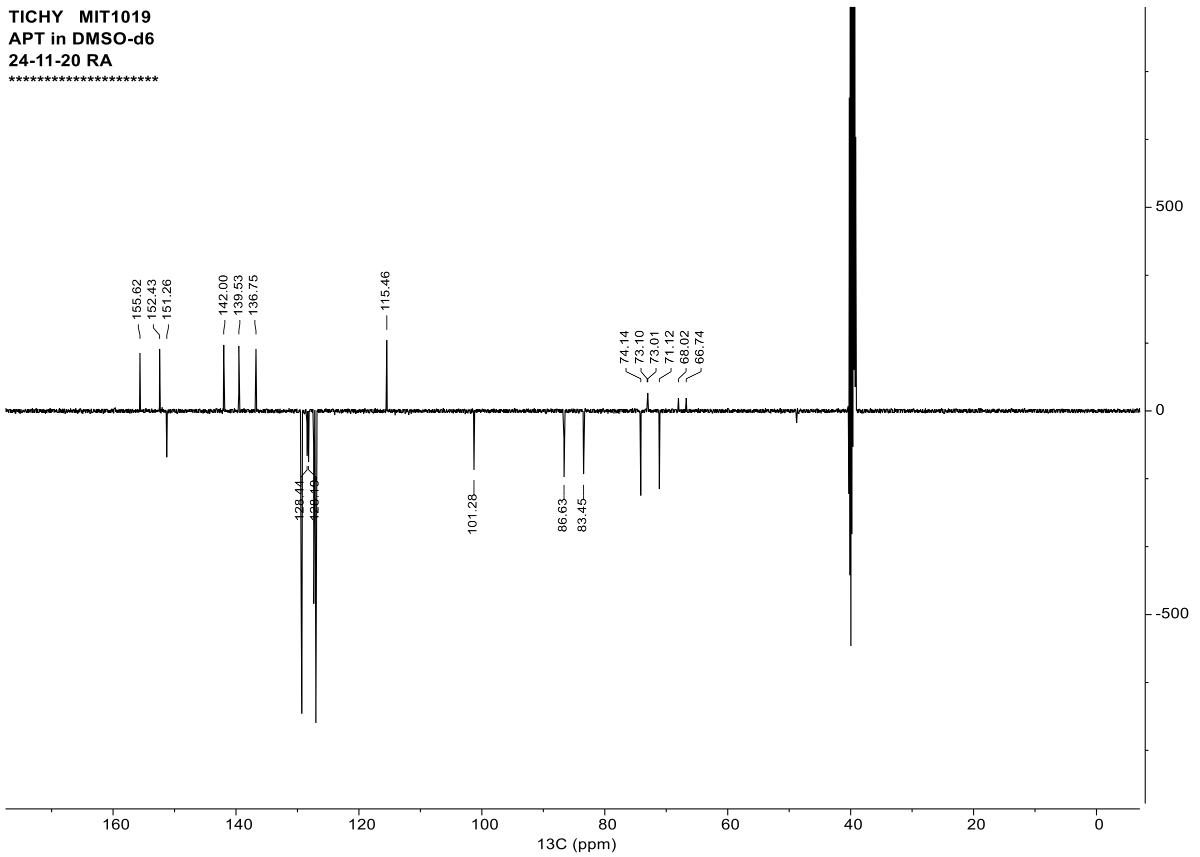
TICHY MIT1019
APT in DMSO-d6
24-11-20 RA
155.62
152.43
151.26
142.00
139.53
136.75
115.46
74.14
73.10
73.01
71.12
68.02
66.74
128.44
101.28
86.63
83.45
13C (ppm)

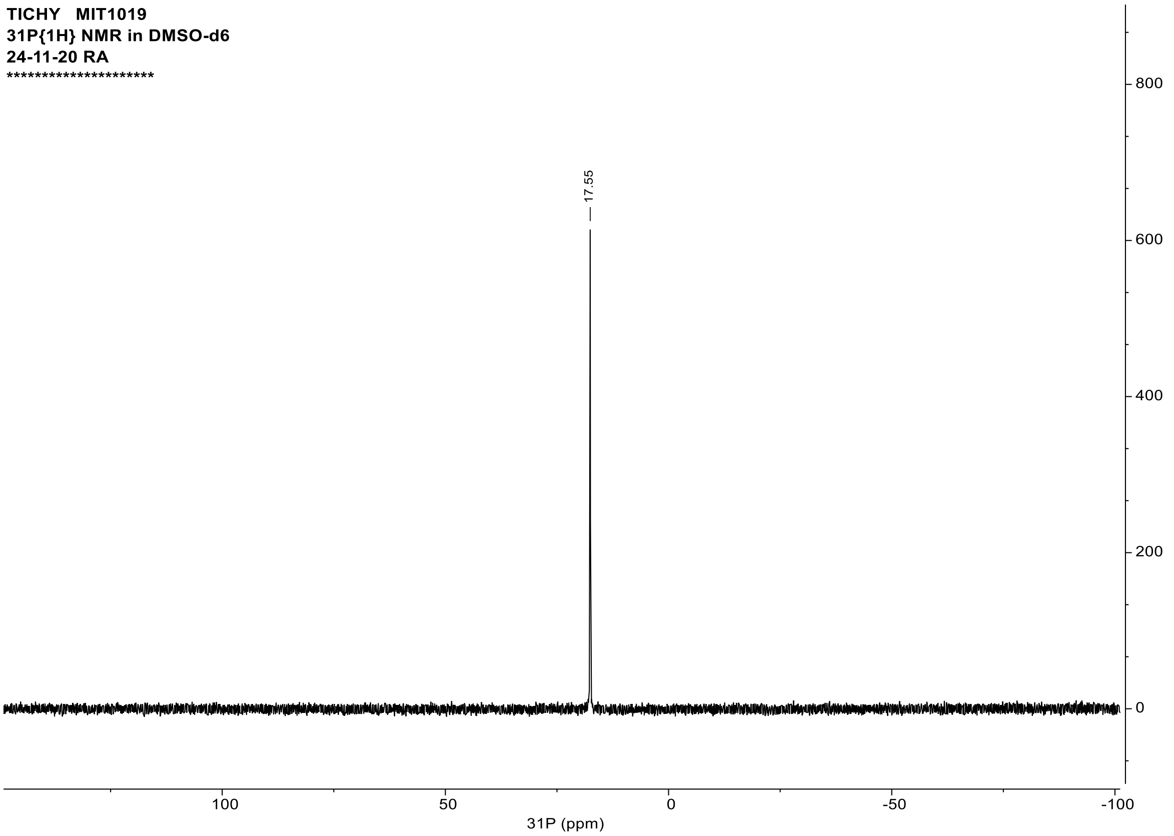
TICHY MIT1019
31P{1H} NMR in DMSO-d6
24-11-20 RA
****
17.55
31P (ppm)

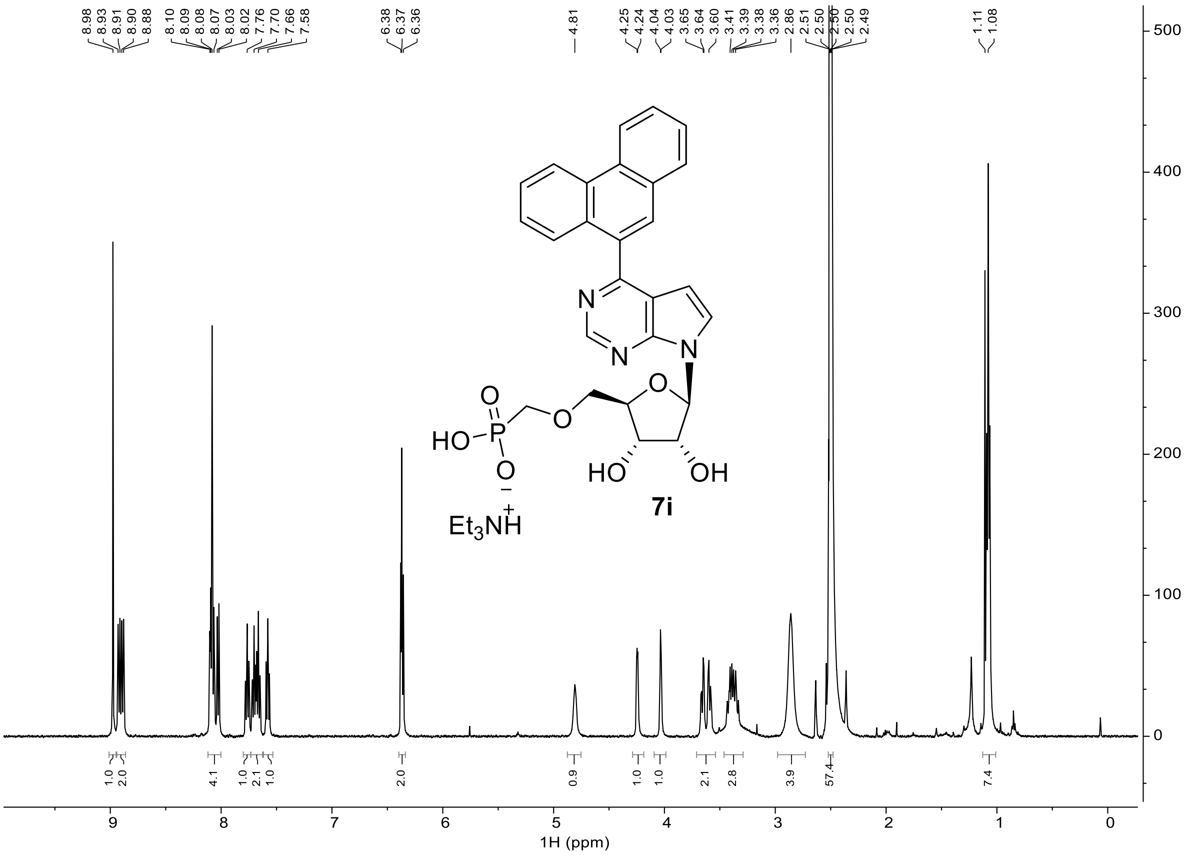
8.98 8.93 8.91 8.90 8.88 8.10 8.09 8.08 8.07 8.03 8.02 7.76 7.70 7.66 7.58 6.38 6.37 6.36 4.81 4.25 4.24 4.04 4.03 3.65 3.64 3.60 3.41 3.39 3.38 3.36 2.86 2.51 2.50 2.50 2.50 2.49 1.11 1.08
1.0 2.0 4.1 1.0 2.1 1.0 2.0 0.9 1.0 1.0 2.1 2.8 3.9 57.4 7.4
HO
O
P
O
N
N
N
HO
OH
Et3NH
7i
1H (ppm)

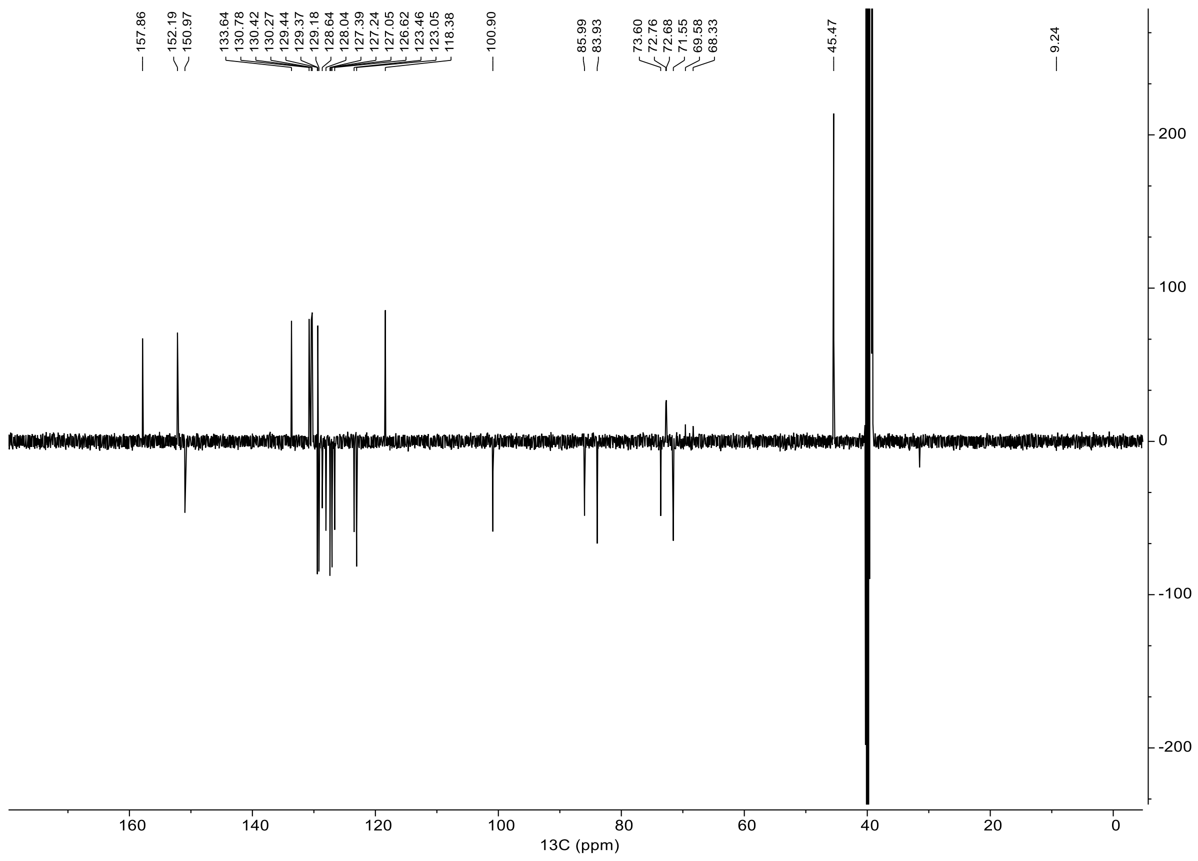
157.86
152.19
150.97
133.64
130.78
130.42
130.27
129.44
129.37
129.18
128.64
128.04
127.39
127.24
127.05
126.62
123.46
123.05
118.38
100.90
85.99
83.93
73.60
72.76
72.68
71.55
69.58
68.33
45.47
9.24
13C (ppm)

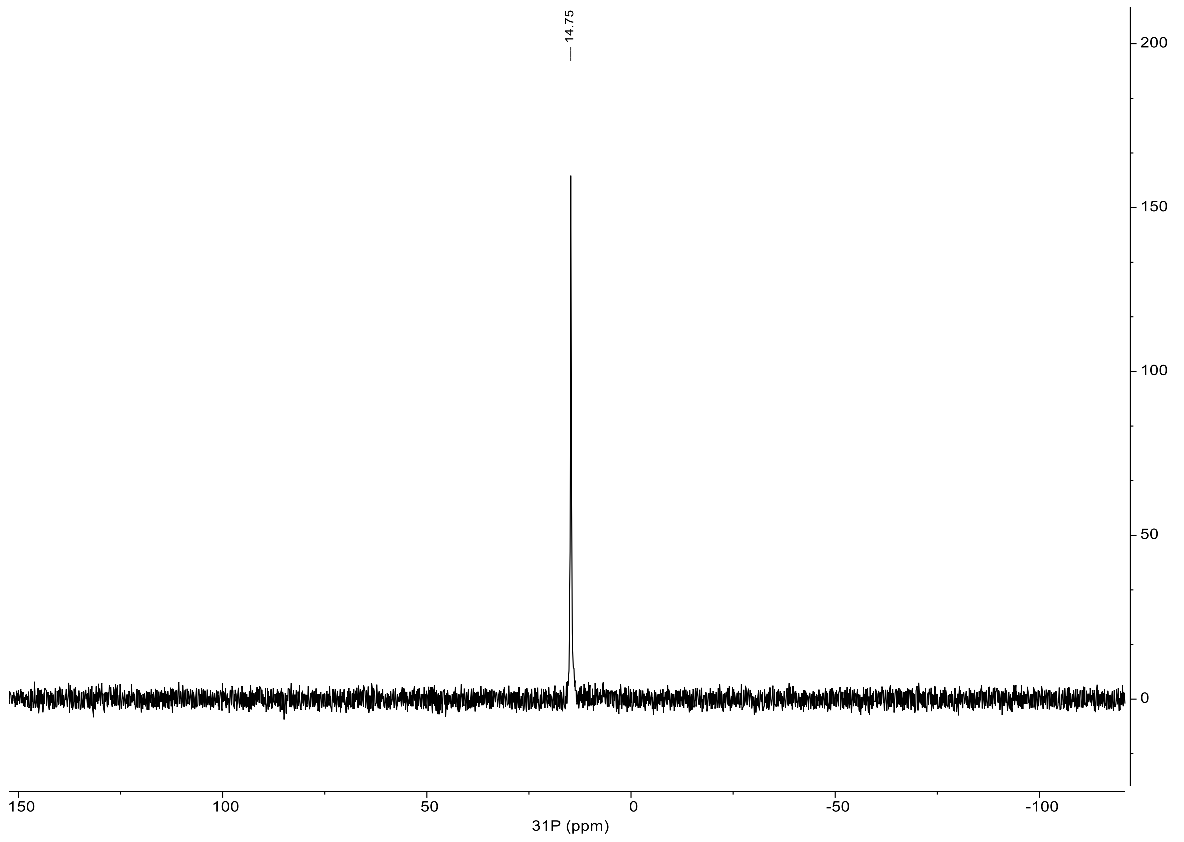
14.75
31P (ppm)

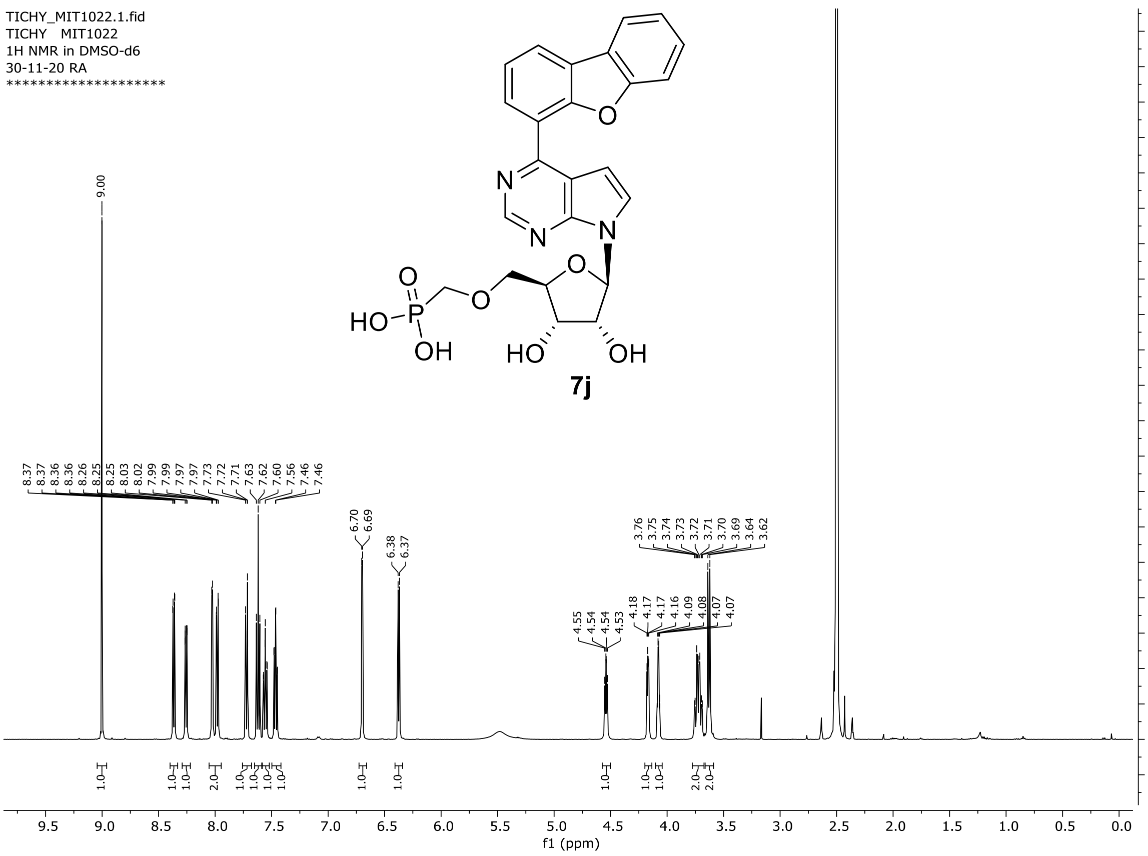
TICHY_MIT1022.1.fid
TICHY MIT1022
1H NMR in DMSO-d6
30-11-20 RA
****
7j
f1 (ppm)

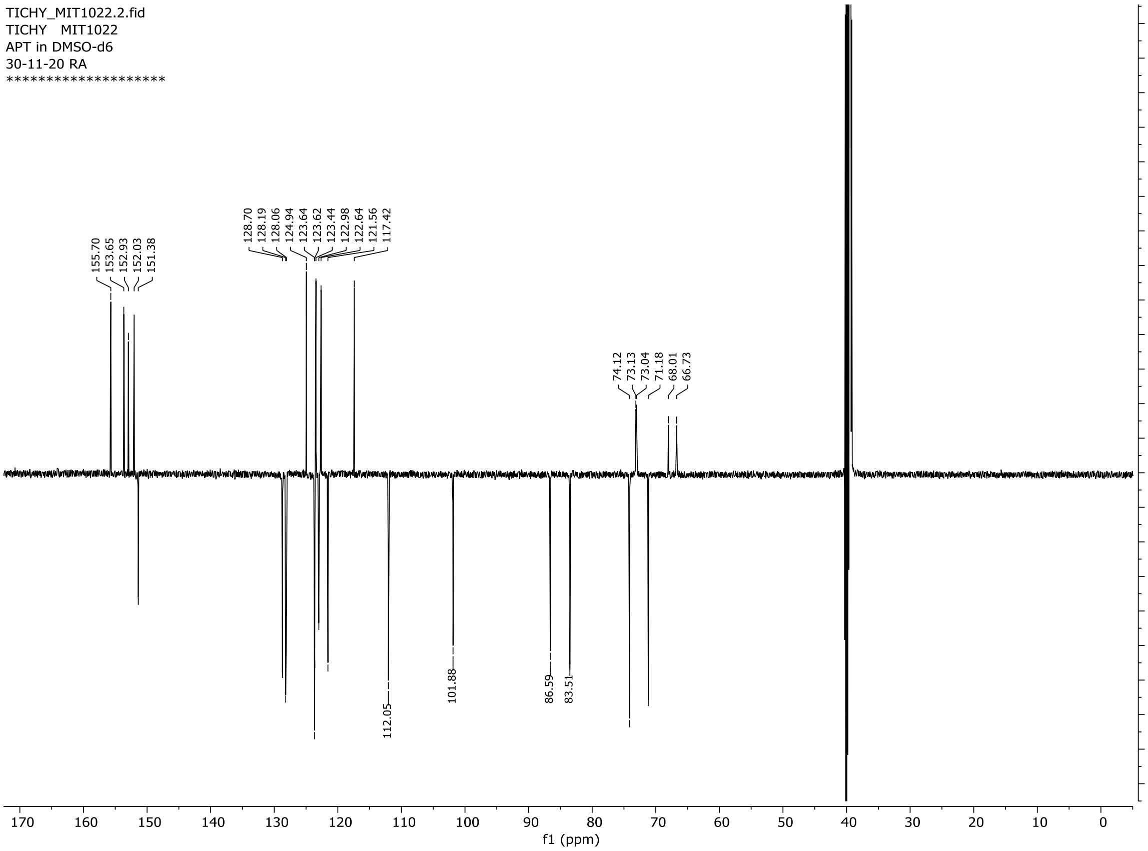
TICHY_MIT1022.2.fid
TICHY MIT1022
APT in DMSO-d6
30-11-20 RA
****
f1 (ppm)

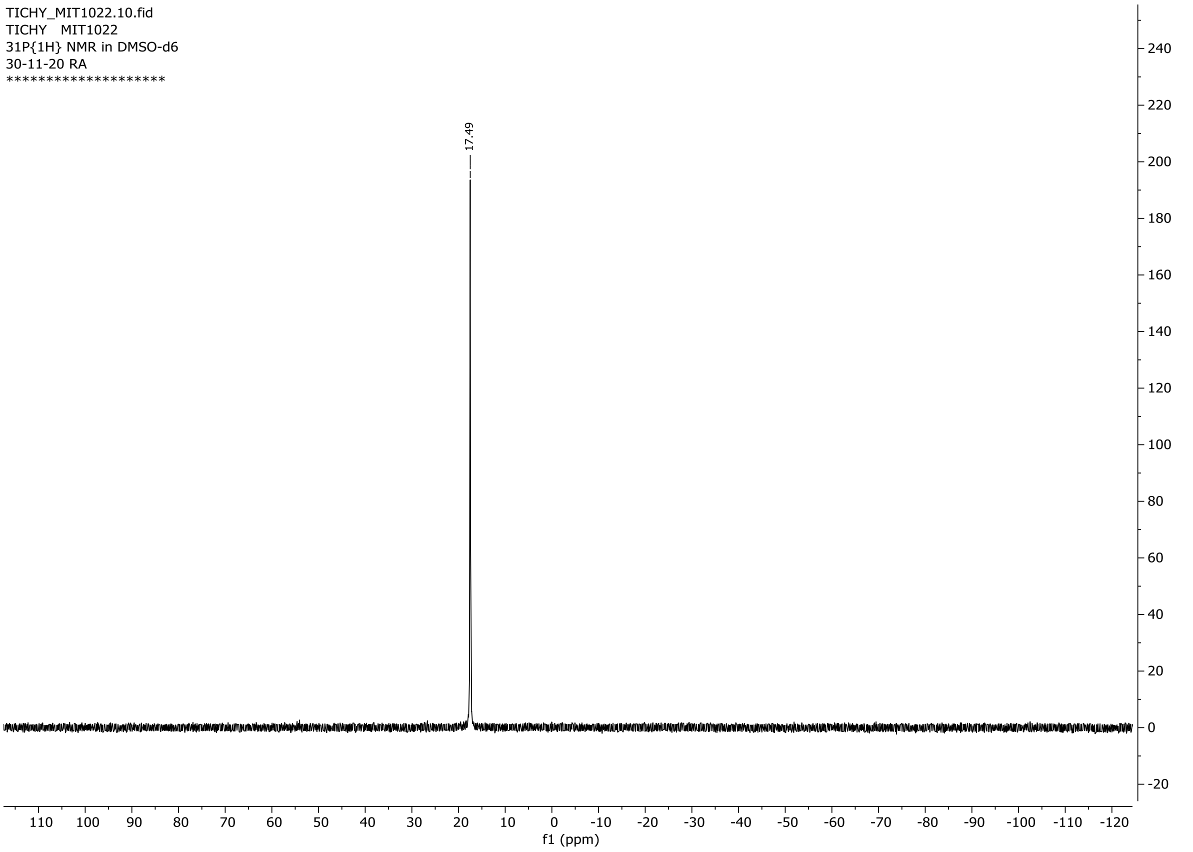

TICHY_MIT1022.10.fid
TICHY MIT1022
31P{1H} NMR in DMSO-d6
30-11-20 RA
****
17.49
f1 (ppm)

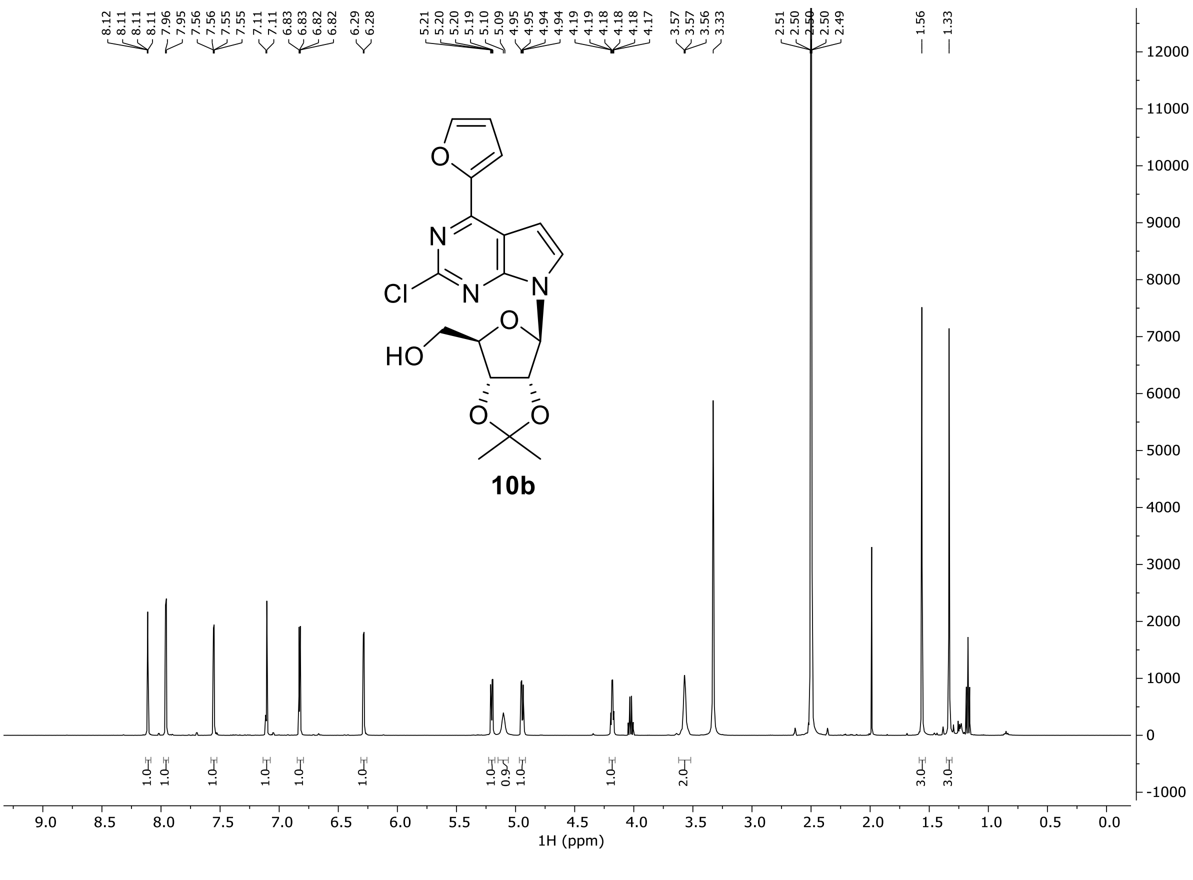

8.12 8.11 8.11 8.11 7.96 7.95 7.56 7.56 7.55 7.55 7.11 7.11 6.83 6.83 6.82 6.82 6.29 6.28
5.21 5.20 5.20 5.19 5.10 5.09 4.95 4.95 4.94 4.94 4.19 4.19 4.18 4.18 4.18 4.17 3.57 3.57 3.56 3.33
2.51 2.50 2.50 2.50 2.49 1.56 1.33
1.0 1.0 1.0 1.0 1.0 1.0 1.0 0.9 1.0 1.0 2.0 3.0 3.0
O
N
N
N
Cl
HO
O
O
O
10b
1H (ppm)

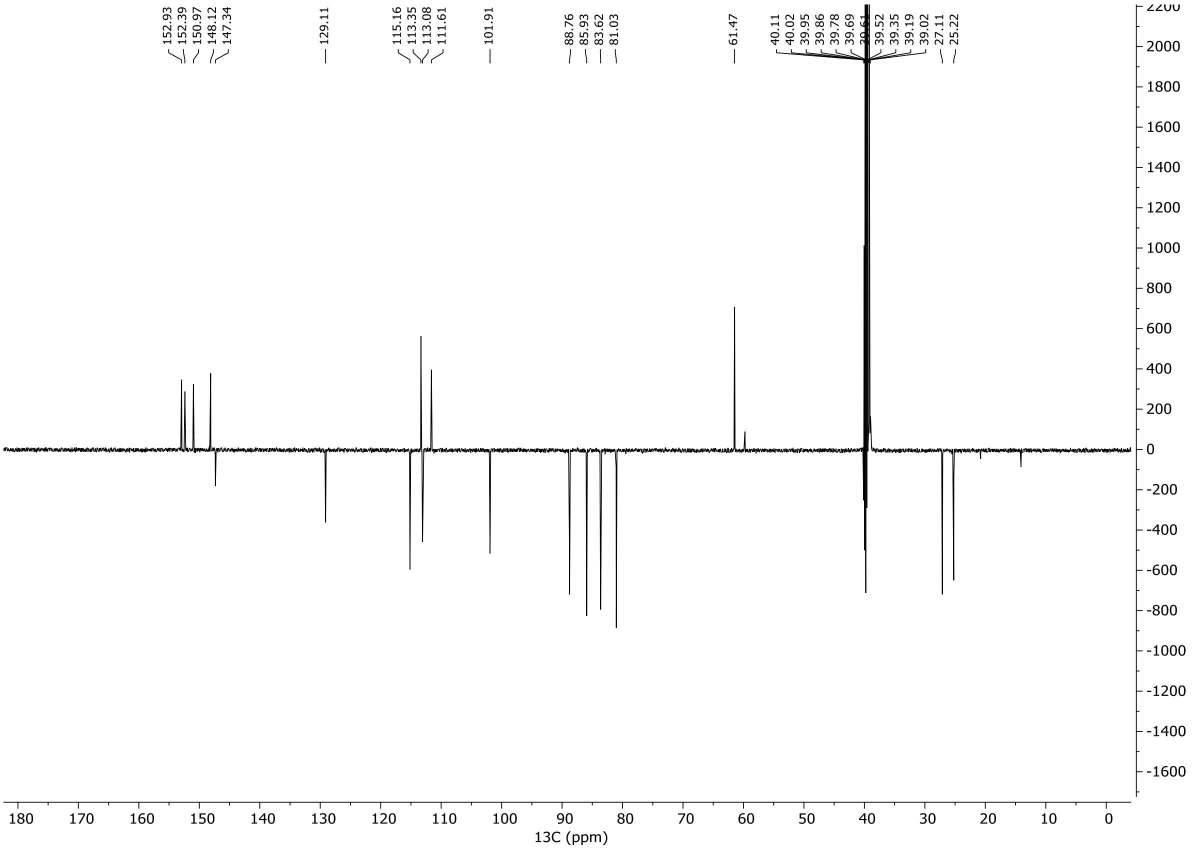
13C (ppm)

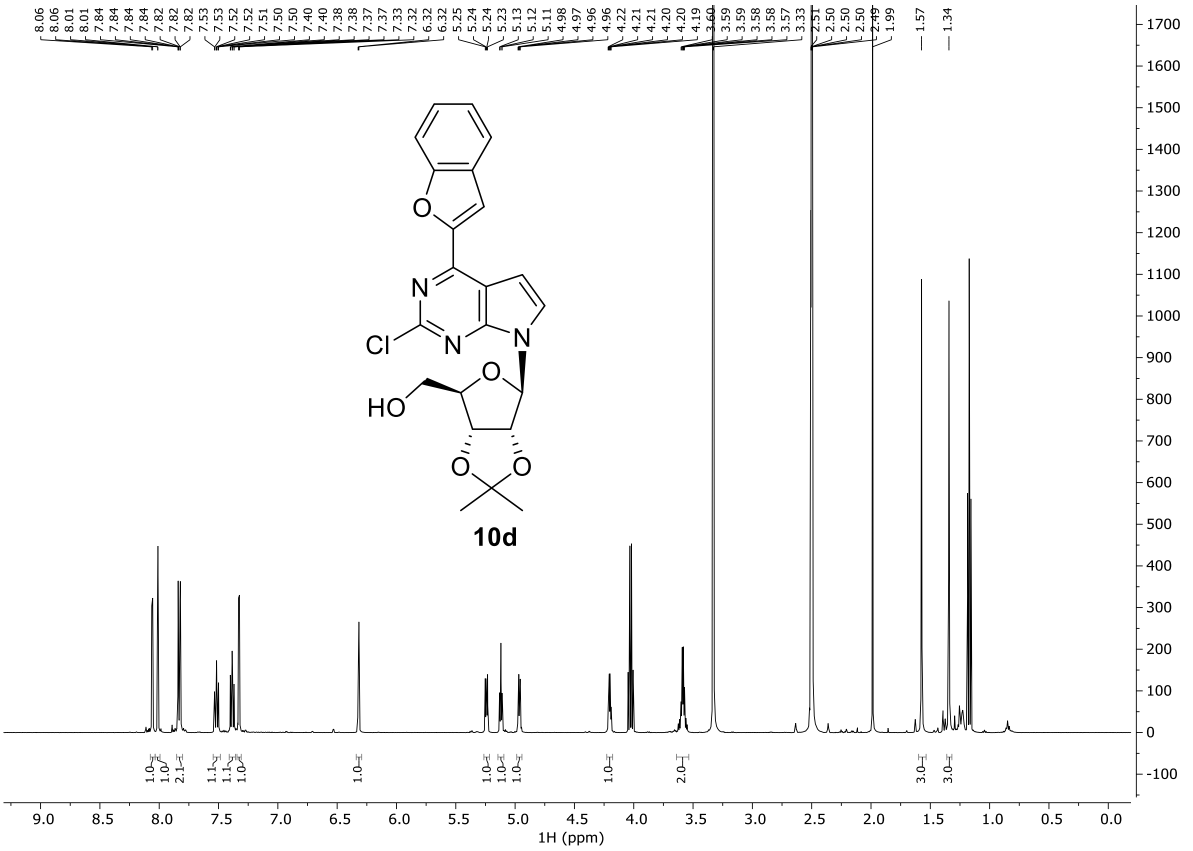
10d
1H (ppm)

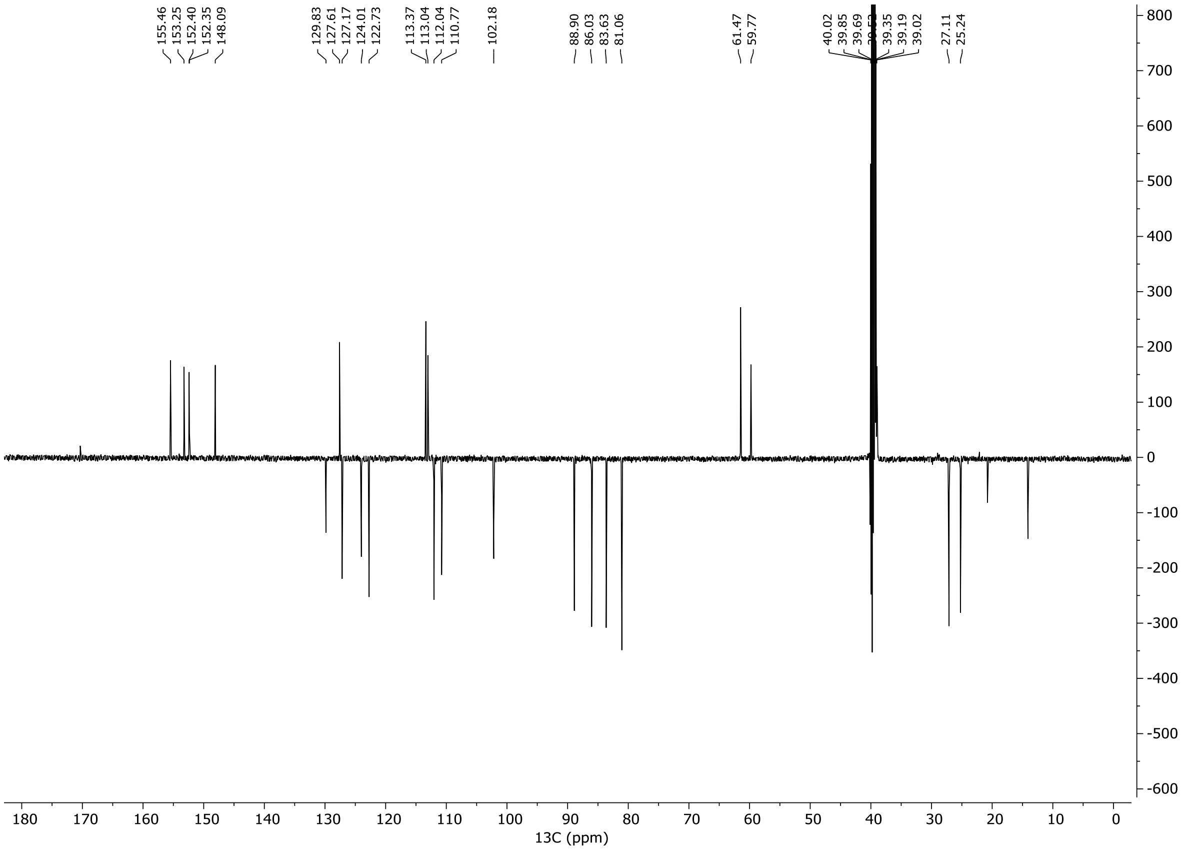
155.46
153.25
152.40
152.35
148.09
129.83
127.61
127.17
124.01
122.73
113.37
113.04
112.04
110.77
102.18
88.90
86.03
83.63
81.06
61.47
59.77
40.02
39.85
39.69
39.35
39.19
39.02
27.11
25.24
13C (ppm)

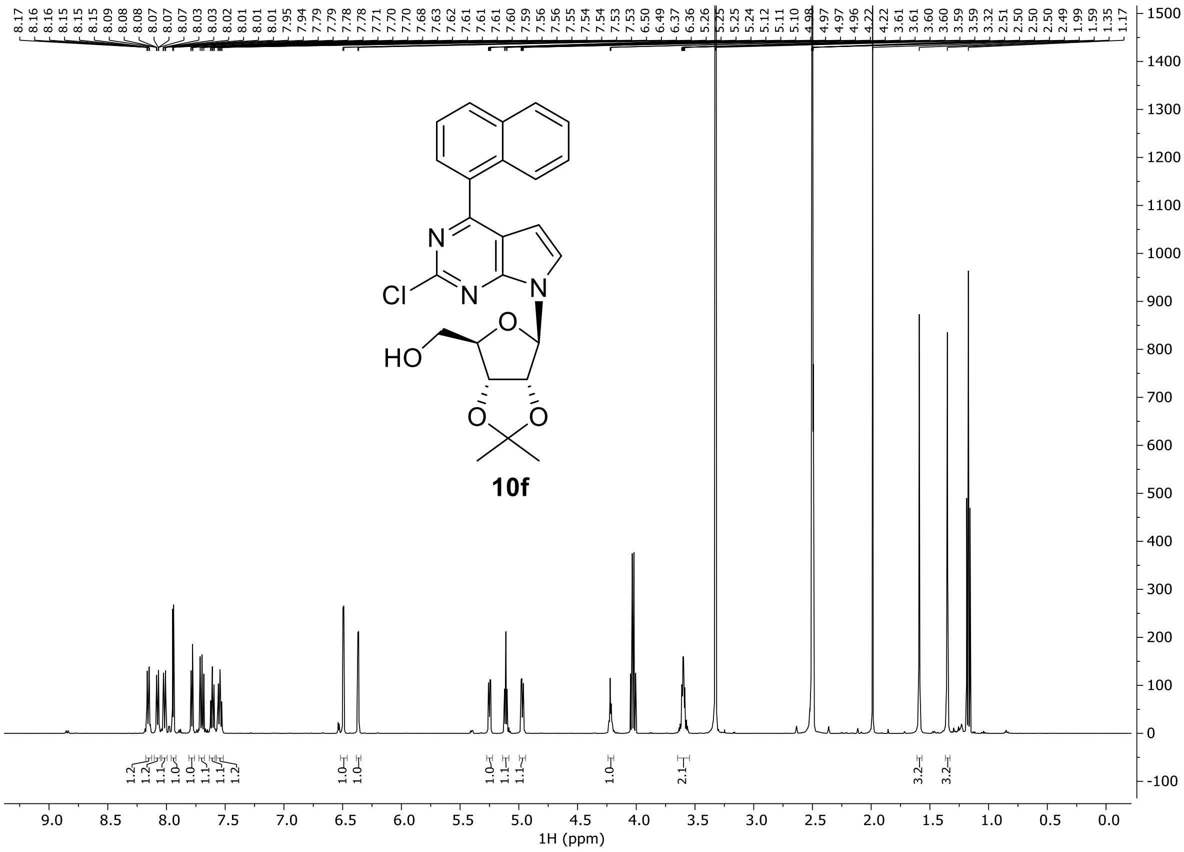
8.17
8.16
8.16
8.15
8.15
8.15
8.09
8.08
8.08
8.07
8.07
8.07
8.03
8.03
8.02
8.01
8.01
7.95
7.94
7.79
7.78
7.78
7.71
7.70
7.70
7.68
7.63
7.62
7.61
7.61
7.61
7.60
7.59
7.56
7.55
7.55
7.54
7.54
7.53
7.53
6.50
6.49
6.37
6.36
5.26
5.25
5.25
5.24
5.12
5.11
5.10
4.98
4.97
4.97
4.96
4.22
4.22
4.22
3.61
3.61
3.60
3.60
3.59
3.59
3.32
2.51
2.50
2.50
2.50
2.49
1.99
1.59
1.35
1.17
N
N
N
Cl
O
HO
O
O
10f
1H (ppm)

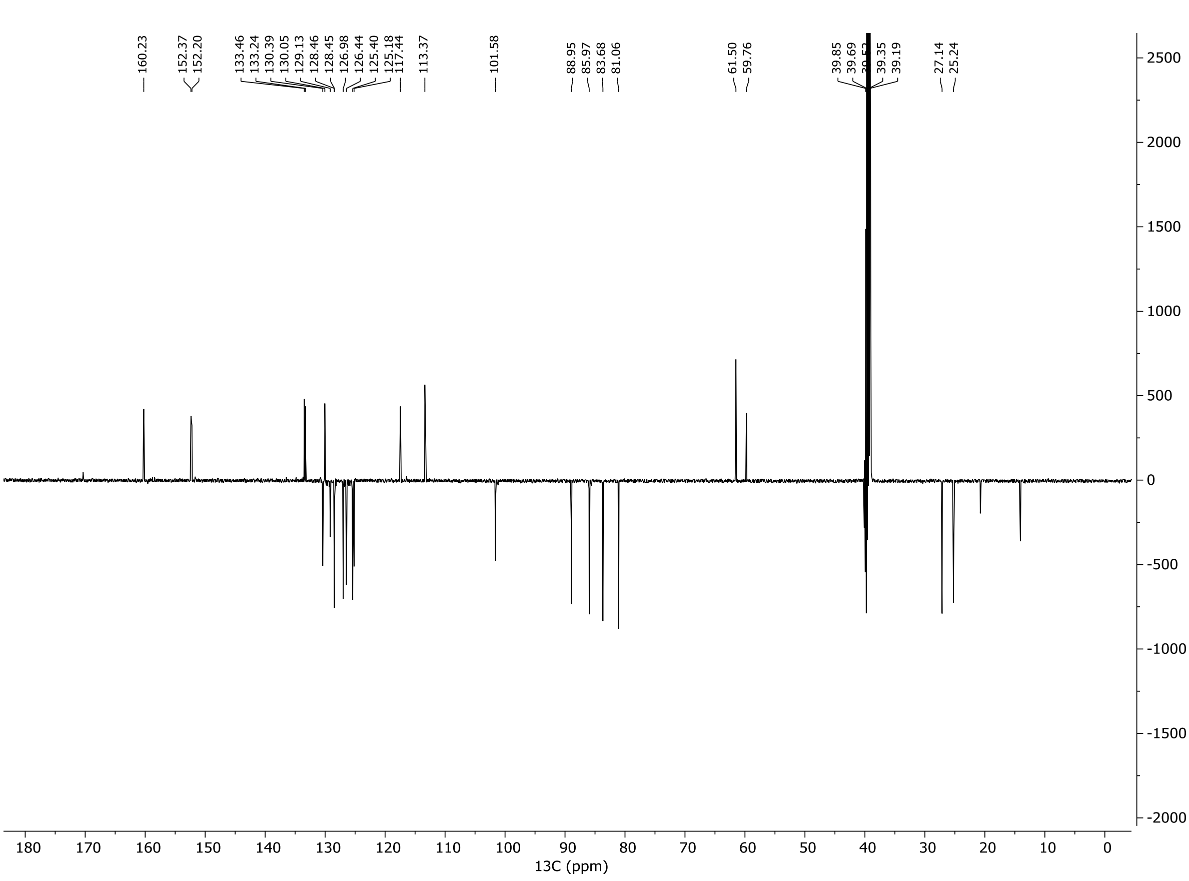
13C (ppm)

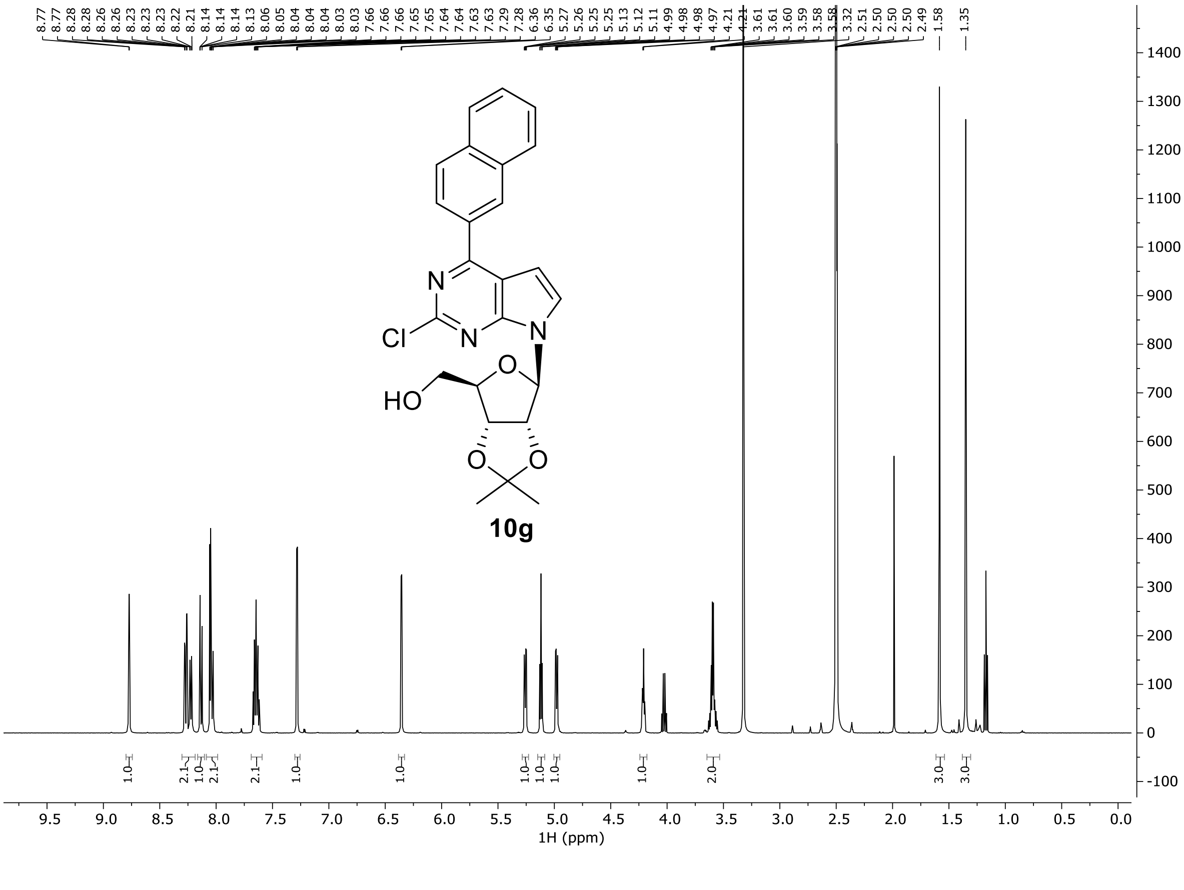
10g
1H (ppm)

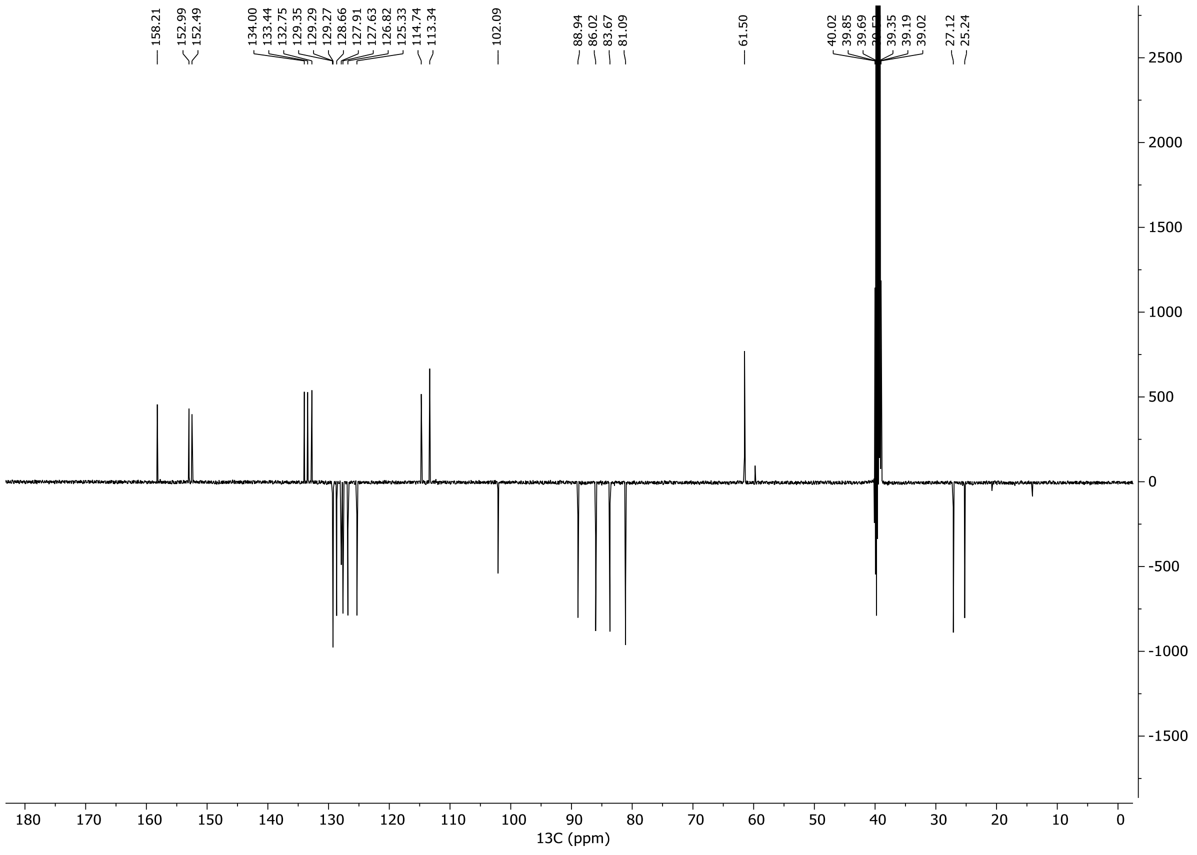
158.21
152.99
152.49
134.00
133.44
132.75
129.35
129.29
129.27
128.66
127.91
127.63
126.82
125.33
114.74
113.34
102.09
88.94
86.02
83.67
81.09
61.50
40.02
39.85
39.69
39.35
39.19
39.02
27.12
25.24
13C (ppm)

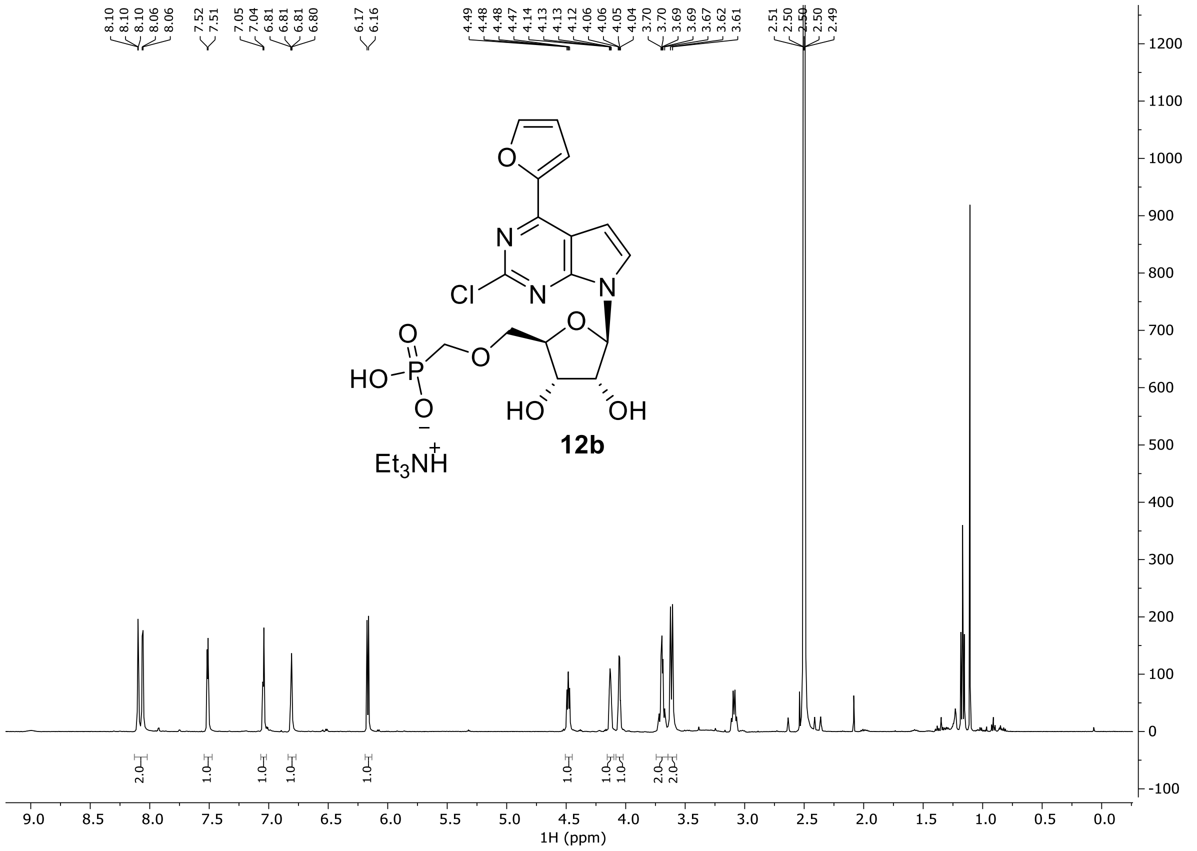
8.10
8.10
8.06
8.06
7.52
7.51
7.05
7.04
6.81
6.81
6.80
6.17
6.16
4.49
4.48
4.48
4.47
4.14
4.13
4.13
4.12
4.06
4.06
4.05
4.04
3.70
3.70
3.69
3.67
3.62
3.61
2.51
2.50
2.50
2.50
2.49
N
N
N
O
Cl
O
O
O
HO
P
O
HO
OH
Et3NH
12b
1H (ppm)

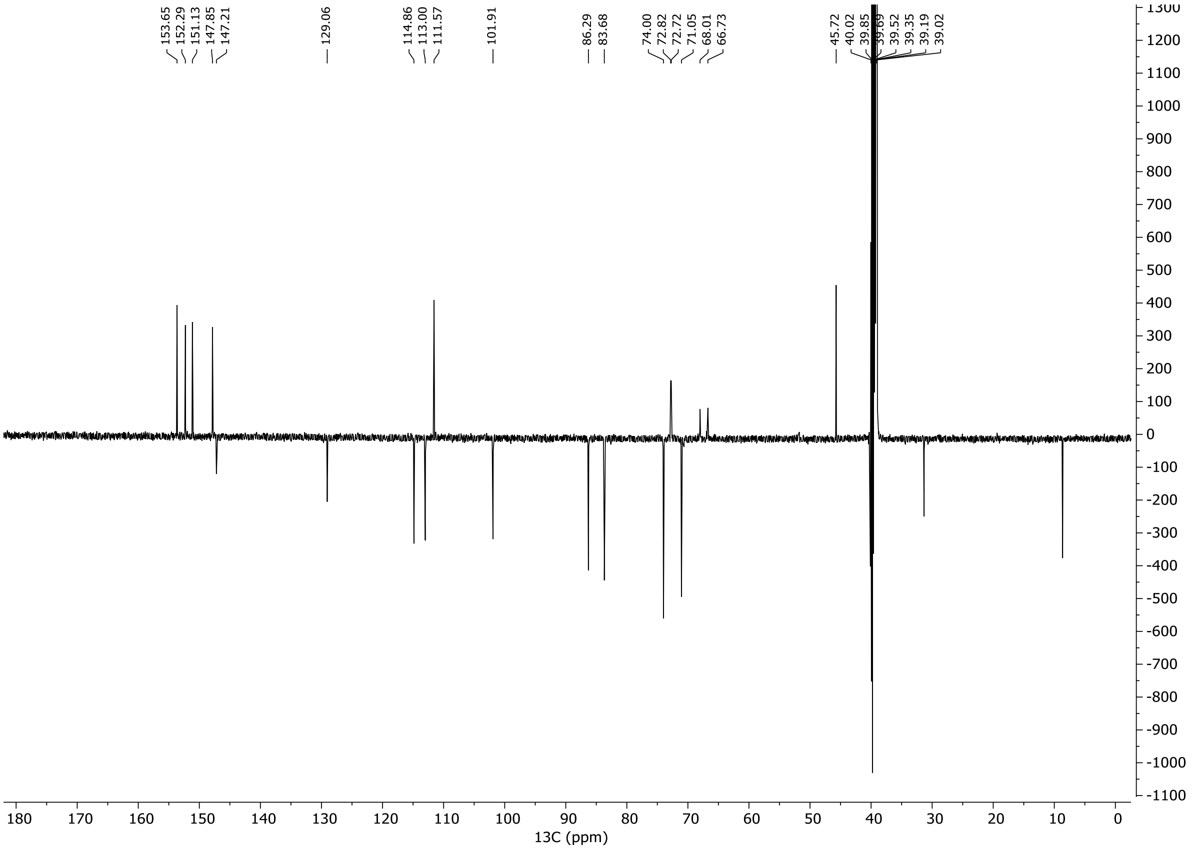
153.65
152.29
151.13
147.85
147.21
129.06
114.86
113.00
111.57
101.91
86.29
83.68
74.00
72.82
72.72
71.05
68.01
66.73
45.72
40.02
39.85
39.69
39.52
39.35
39.19
39.02
13C (ppm)

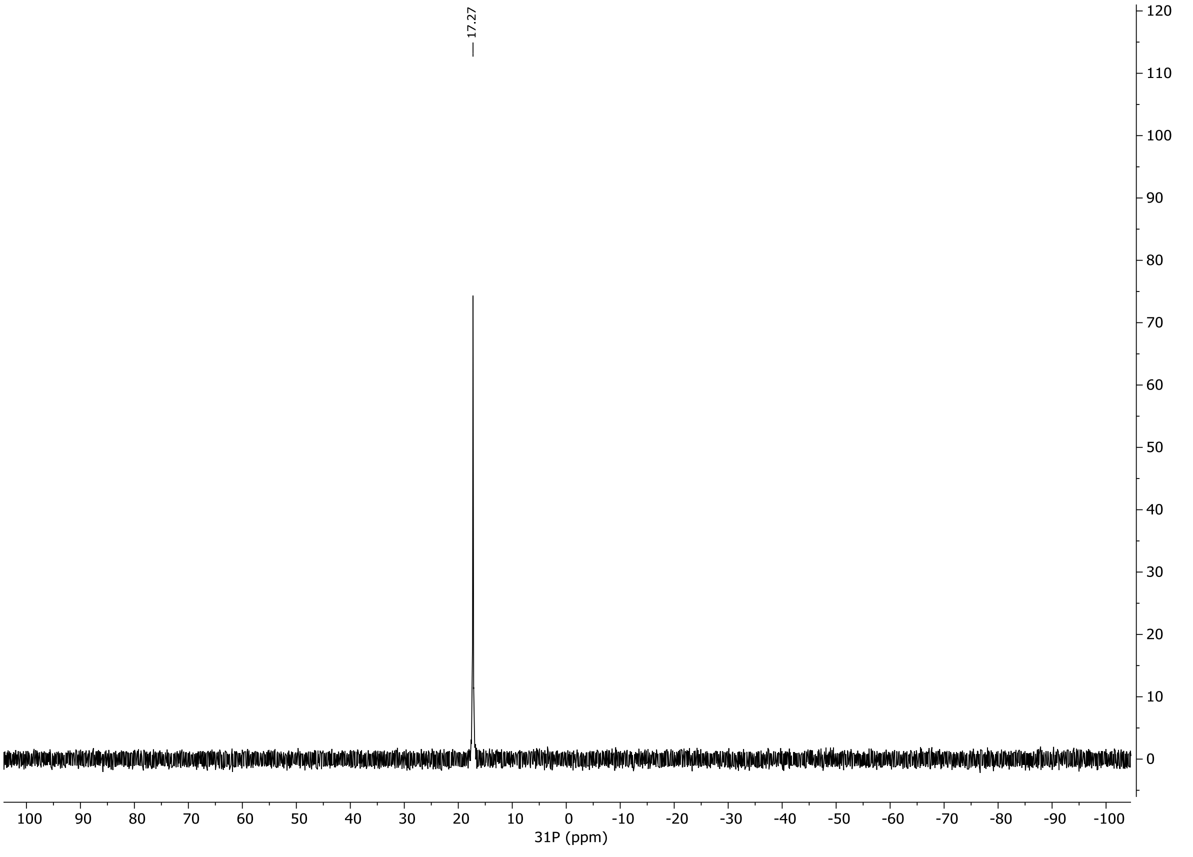
17.27
31P (ppm)

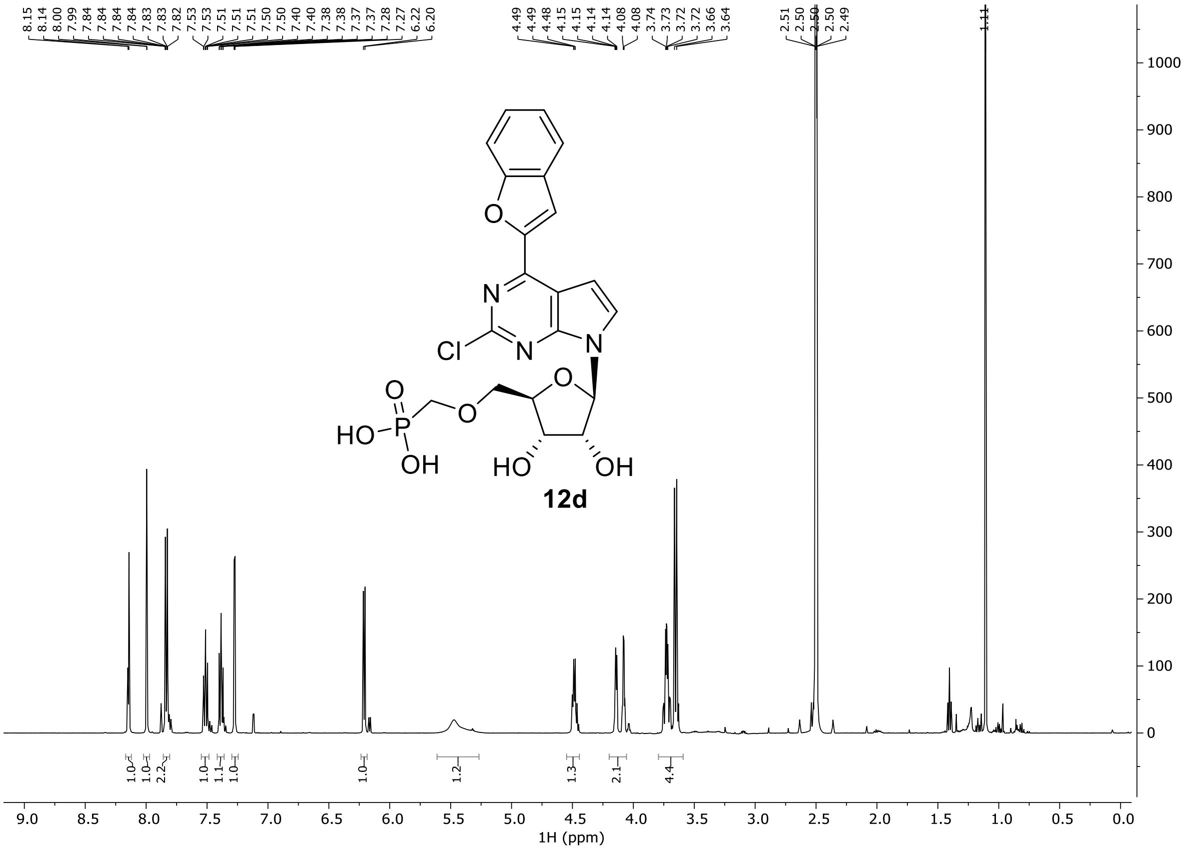
12d
1H (ppm)

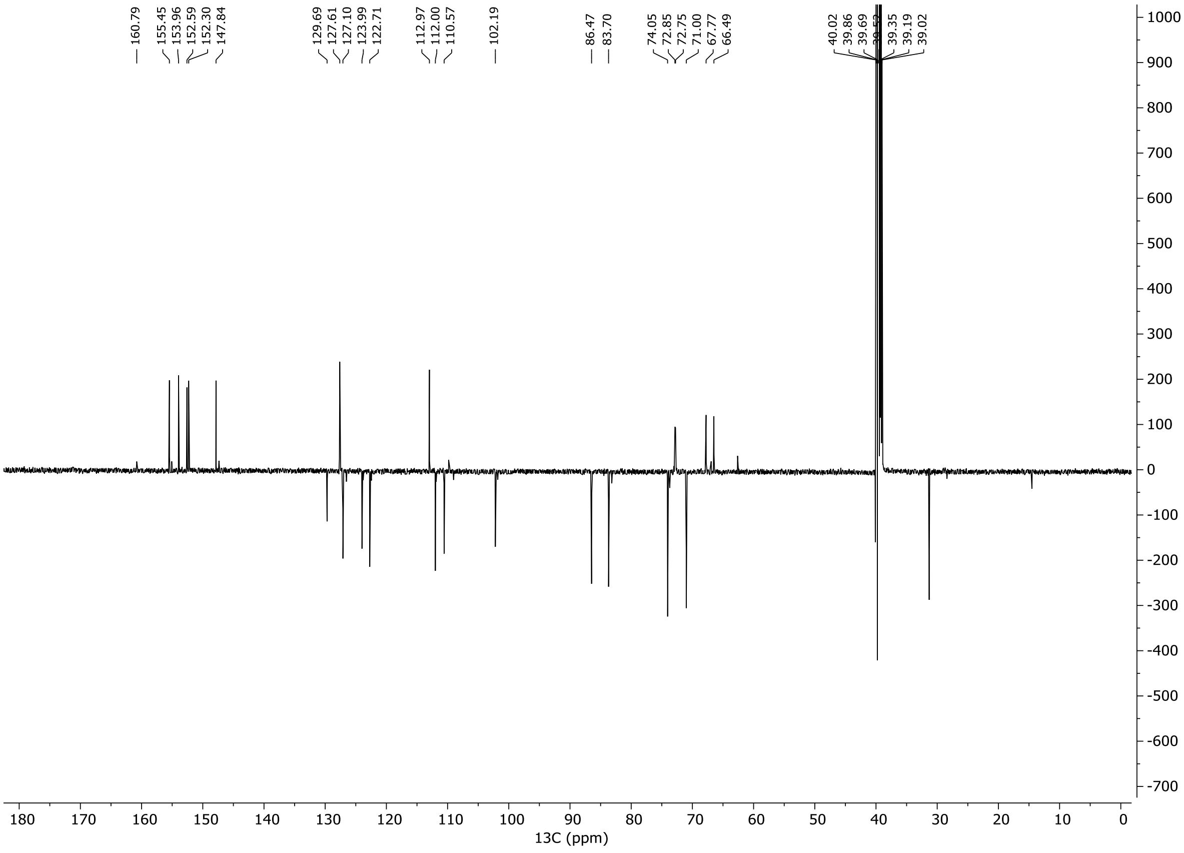
13C (ppm)

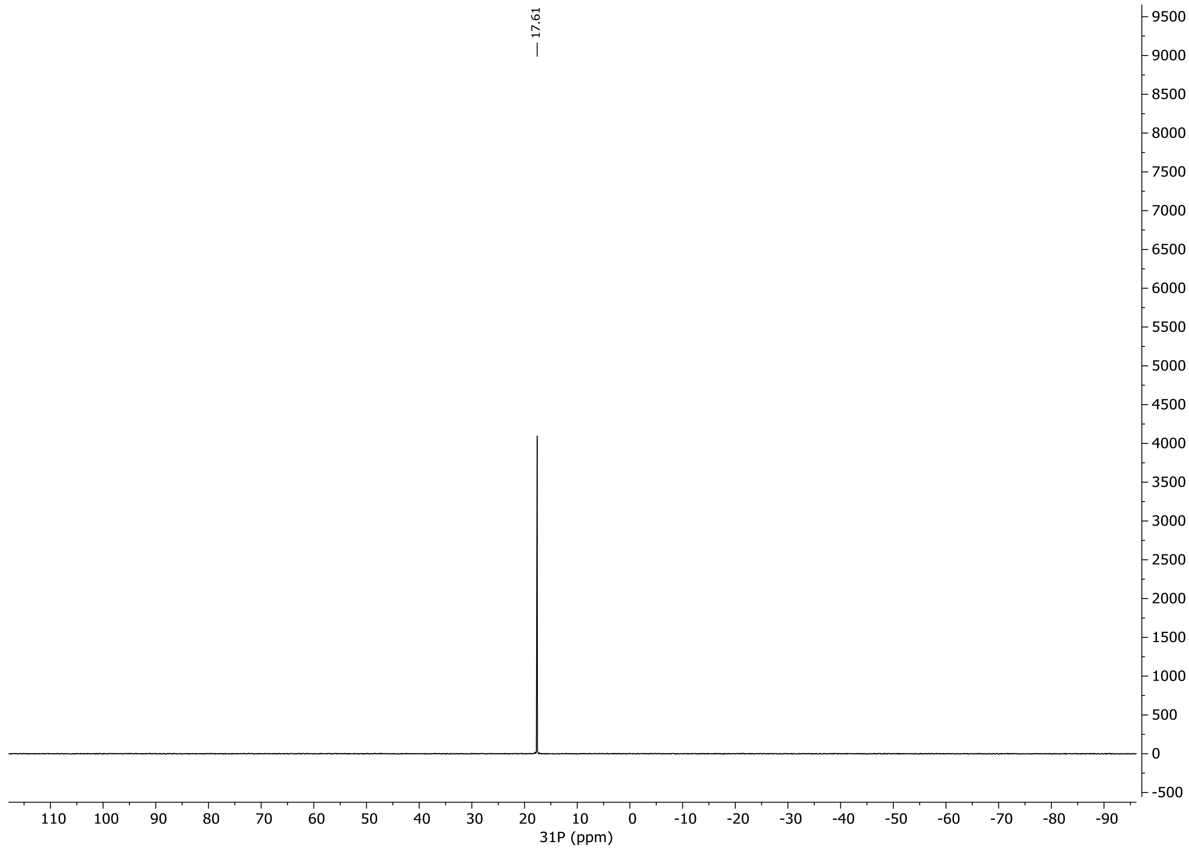

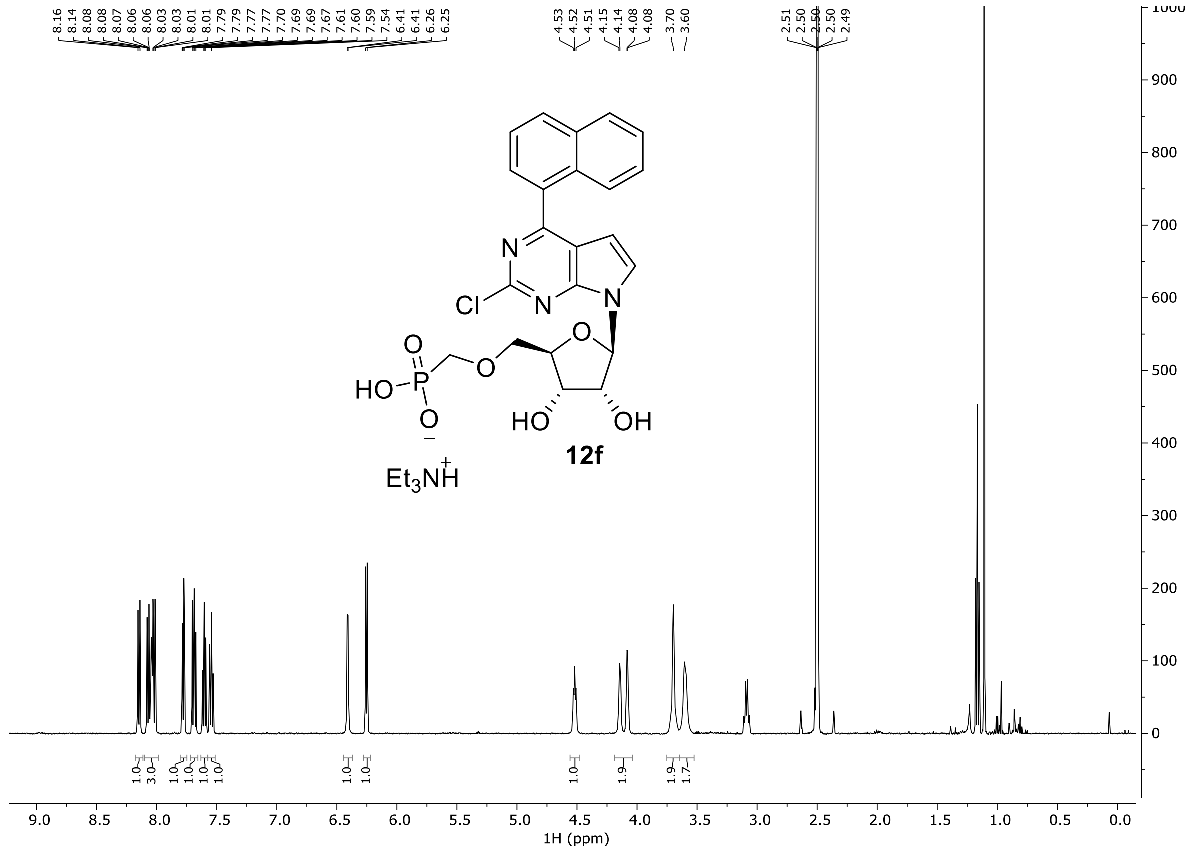

Cl
N
O
HO
OH
P
Et$_3$NH$^+$
12f
1H (ppm)

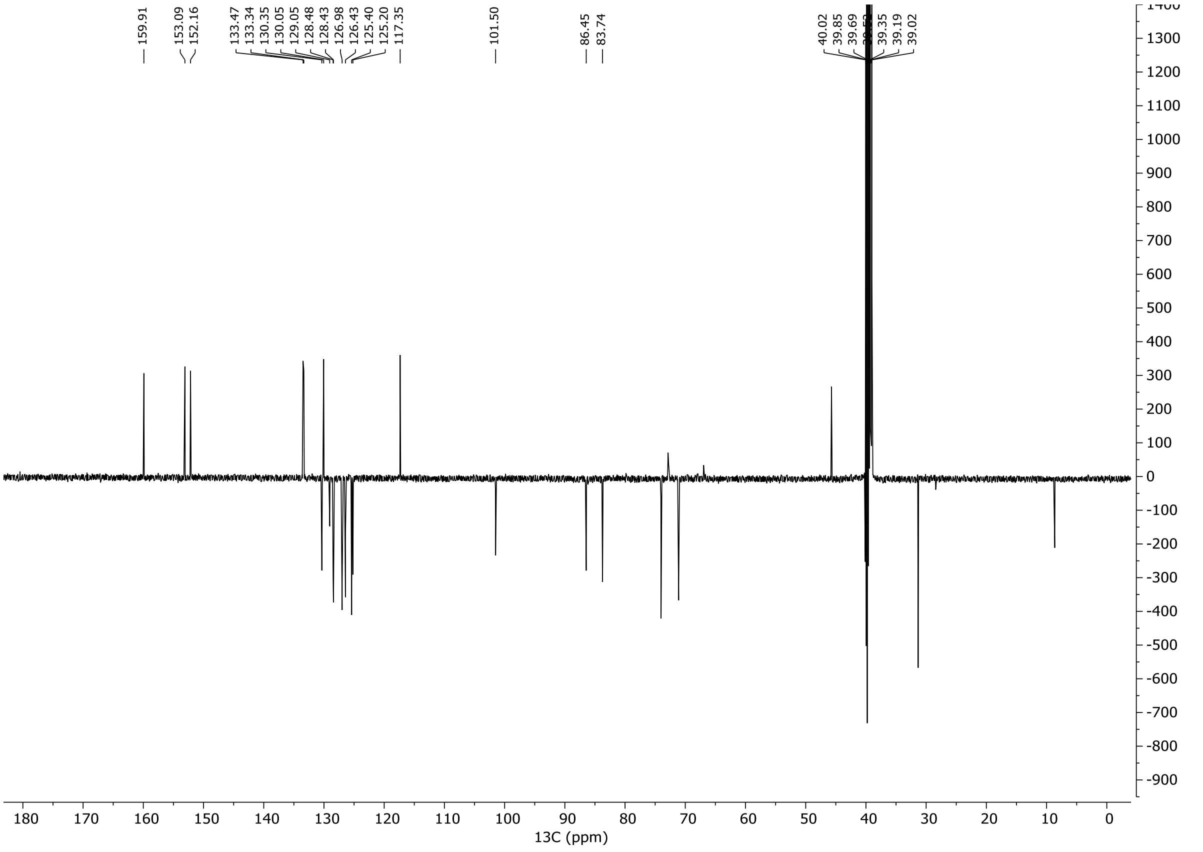
159.91
153.09
152.16
133.47
133.34
130.35
130.05
129.05
128.48
128.43
126.98
126.43
125.40
125.20
117.35
101.50
86.45
83.74
40.02
39.85
39.69
39.35
39.19
39.02
13C (ppm)

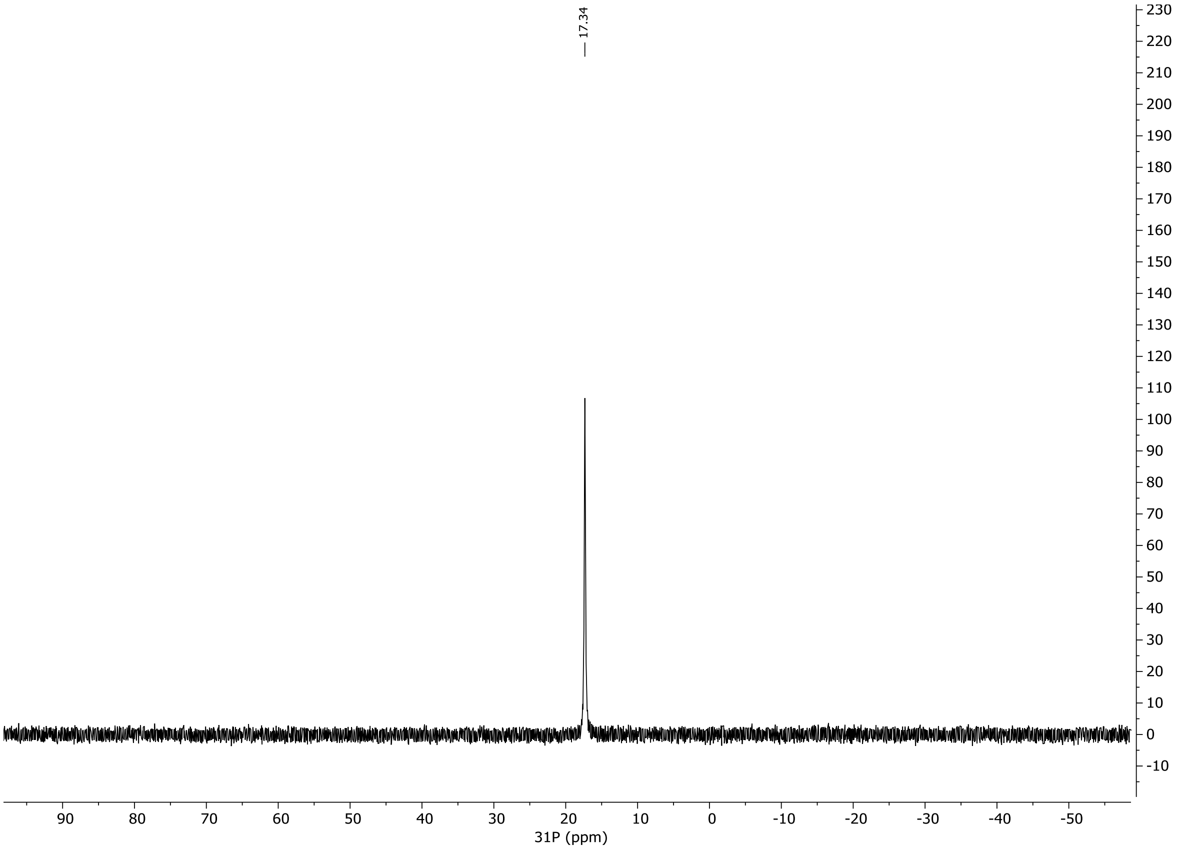
17.34
31P (ppm)

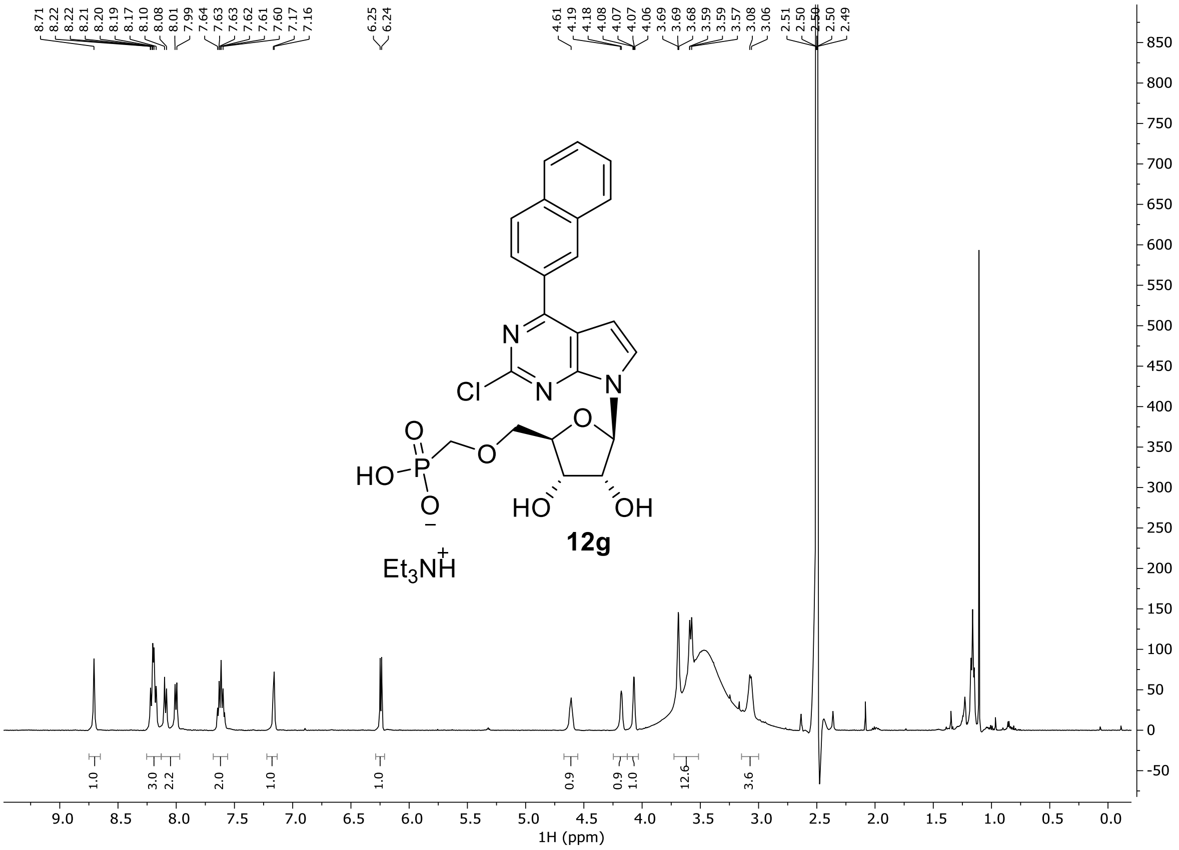
12g
Cl
HO
OH
Et_3NH^+
1H (ppm)

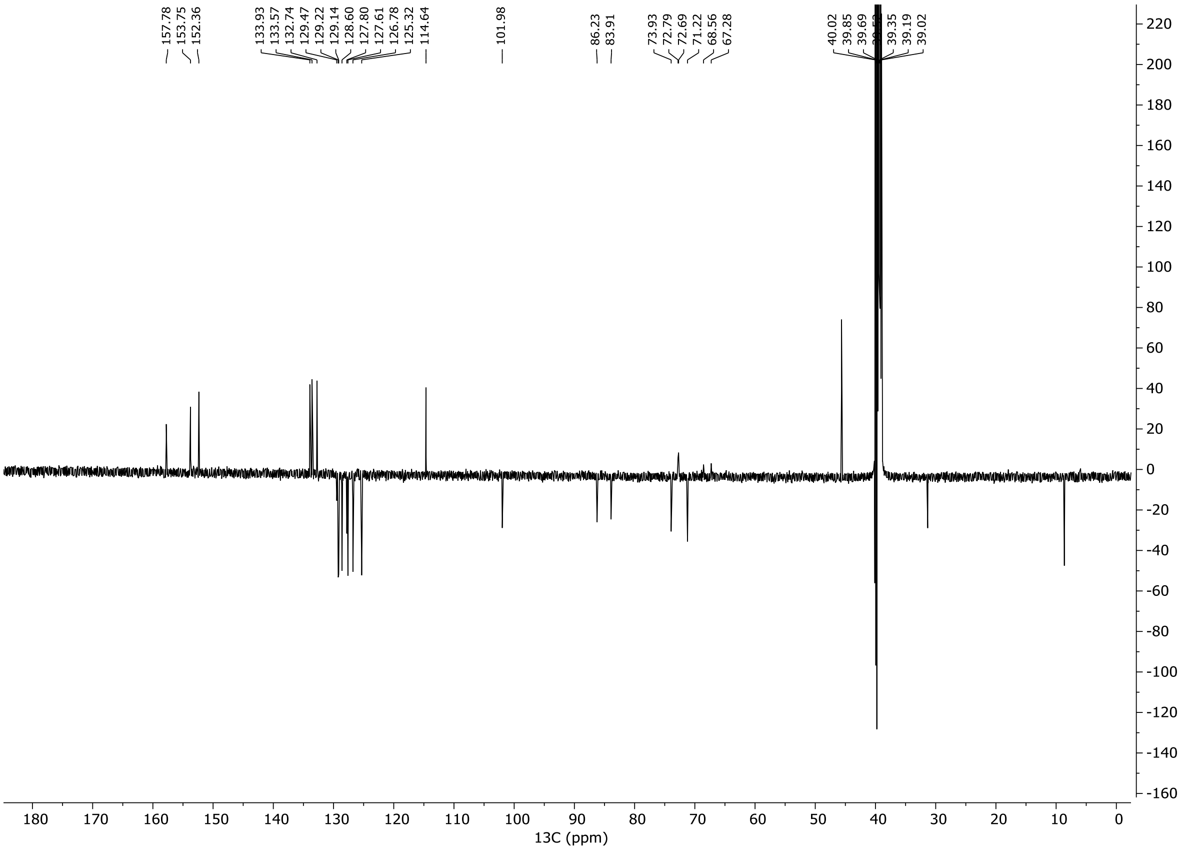
13C (ppm)

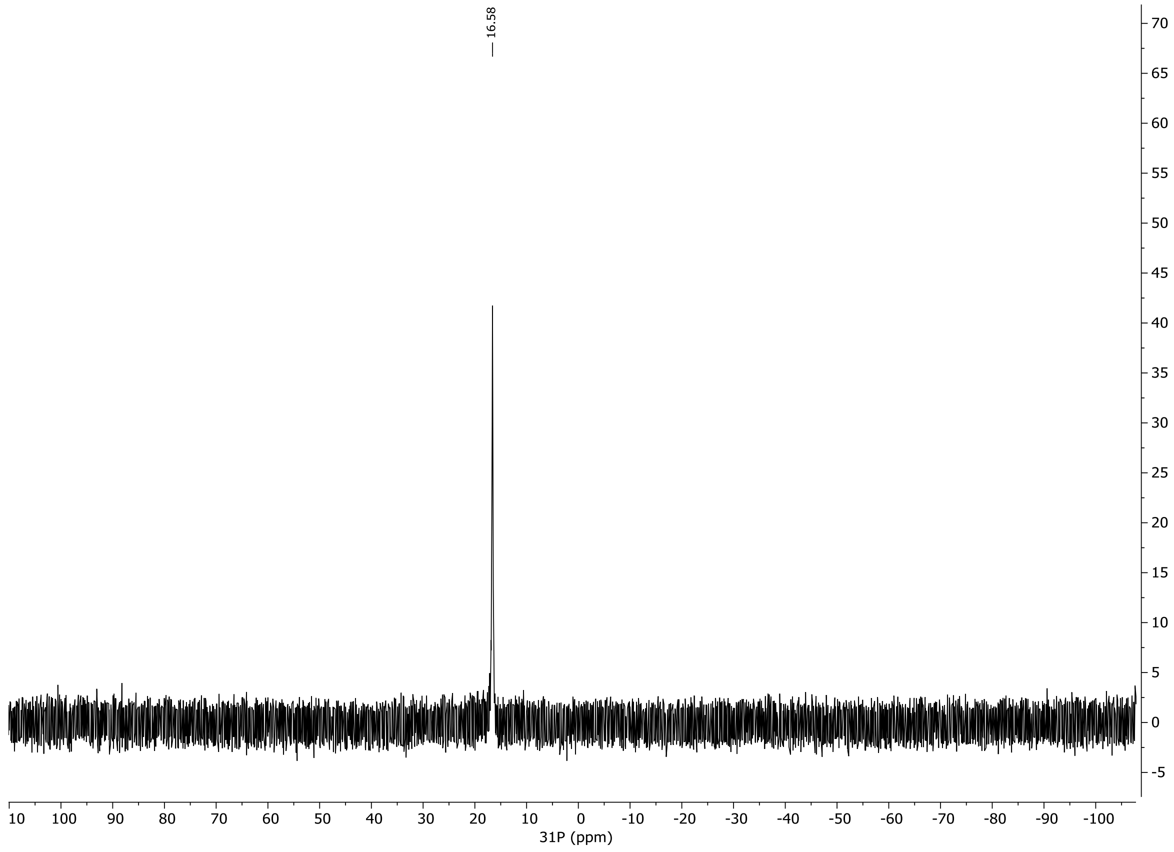

References

(1) An, H.; Statsyuk, A. V. Development of activity-based probes for ubiquitin and ubiquitin-like protein signaling pathways. *J Am Chem Soc* **2013**, *135* (45), 16948-16962. DOI: 10.1021/ja4099643

(2) Malnuit, V.; Smolen, S.; Tichy, M.; Slavetínská, L. P.; Hocek, M. Synthesis of Cyclic and Acyclic Nucleoside Phosphonates and Sulfonamides Derived from 6-(Thiophen-2-yl)-7-fluoro-7-deazapurine. *Eur J Org Chem* **2019**, *2019* (31-32), 5409-5423. DOI: 10.1002/ejoc.201900509

(3) Wu, T.; Brehmer, D.; Beke, L.; Boeckx, A.; Diels, G. S. M.; Gilissen, R. A. H. J.; Lawson, E. C.; Pande, V.; Parade, M. C. B. C.; Schepens, W. B. G.; Thuring, J. W. J. F.; Viellevoye, M.; Sun, W.; Meerpoel, L. Novel 6-6 bicyclic aromatic ring substituted nucleoside analogue for use as PRMT5 inhibitors. WO2017/032840A1, 2017.
